# Rapid and column-free syntheses of acyl fluorides and peptides using *ex situ* generated thionyl fluoride†

**DOI:** 10.1039/d1sc05316g

**Published:** 2021-11-29

**Authors:** Cayo Lee, Brodie J. Thomson, Glenn M. Sammis

**Affiliations:** Department of Chemistry, University of British Columbia 2036 Main Mall Vancouver British Columbia V6T 1Z1 Canada gsammis@chem.ubc.ca

## Abstract

Thionyl fluoride (SOF_2_) was first isolated in 1896, but there have been less than 10 subsequent reports of its use as a reagent for organic synthesis. This is partly due to a lack of facile, lab-scale methods for its generation. Herein we report a novel protocol for the *ex situ* generation of SOF_2_ and subsequent demonstration of its ability to access both aliphatic and aromatic acyl fluorides in 55–98% isolated yields under mild conditions and short reaction times. We further demonstrate its aptitude in amino acid couplings, with a one-pot, column-free strategy that affords the corresponding dipeptides in 65–97% isolated yields with minimal to no epimerization. The broad scope allows for a wide range of protecting groups and both natural and unnatural amino acids. Finally, we demonstrated that this new method can be used in sequential liquid phase peptide synthesis (LPPS) to afford tri-, tetra-, penta-, and decapeptides in 14–88% yields without the need for column chromatography. We also demonstrated that this new method is amenable to solid phase peptide synthesis (SPPS), affording di- and pentapeptides in 80–98% yields.

## Abstract 2

Thionyl fluoride (SOF_2_) was developed as a rapid acid activation peptide coupling reagent for liquid phase peptide synthesis (LPPS) and solid phase peptide synthesis (SPPS).

## Graphical abstract

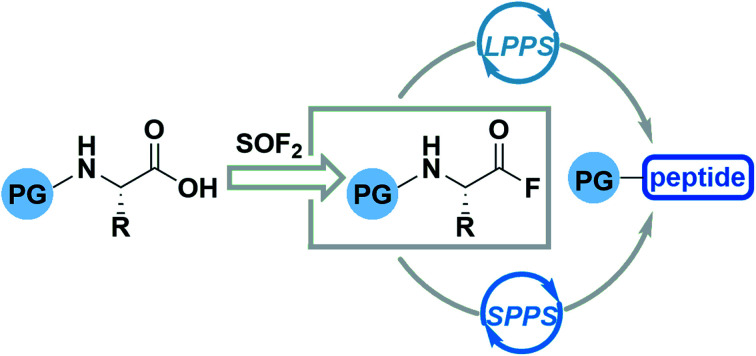

The past decade has witnessed a resurgence in the application of sulfur(vi) fluorides to organic synthesis. The majority of these studies have focused on the commodity chemical sulfuryl fluoride (SO_2_F_2_)^1^ and its derivatives,^2^ which readily react with a wide variety of oxygen-containing functional groups, such as alcohols (1),^3^ oximes (2),^4^ and carboxylates (3),^5^ to form activated fluorosulfate intermediates (5). Fluorosulfates behave like triflate surrogates and have been used in a wide variety of subsequent transformations.^6^ Due to the mild reaction conditions and stable sulfate byproducts, many of these transformations can be carried out in a single reaction vessel and often do not require flash column chromatography for purification.^7^ While fluorosulfate derivatives are powerful for some transformations, they are highly reactive and often undergo undesired side reactions. This problem is exemplified in peptide couplings, where epimerization is observed alpha to the initially formed acyl fluorosulfate (5, R^5^ = R′C(O), Fig. 1).^5*a*^ Despite the importance of a liquid phase, column-free method for peptide coupling, it remains an unsolved challenge for S–F based reagents^8^ and other non-sulfur based deoxyfluorinating agents.^9^

**Fig. 1:**
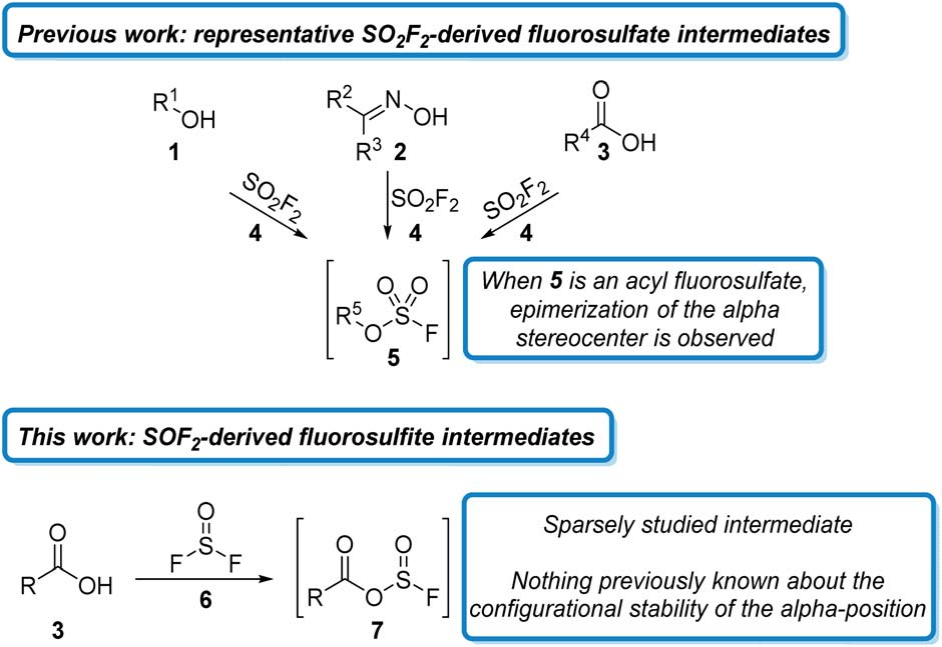
Activation of carboxylic acids for peptide coupling using sulfur fluoride gasses.

An alternative, and largely unexplored strategy for carboxylic acid activation is to access the analogous acylfluorosulfite intermediate (7). These intermediates are less reactive at the acyl carbon than the analogous fluorosulfate derivates and should, therefore, be less susceptible to epimerization.^10^ One reagent that could be used to access these sulfite intermediates is thionyl fluoride (6). Thionyl fluoride is more reactive than sulfuryl fluoride, which should increase the rate of carboxylate activation.^11^ Intriguingly, thionyl fluoride has received very little attention as a reagent. The synthesis of thionyl fluoride was first reported in 1896,^12^ but it was not until 1985 when Shreeve and coworkers reported on its use to react with phosphorous derivatives, amines, and alkanes.^13^ Since then, only four manuscripts and three patents have detailed its use as a reagent.^14^ These studies indicate that thionyl fluoride forms activated fluorosulfite intermediates in the presence of oxygen nucleophiles, but the reactivity of these intermediates has not been extensively studied.

Key to further investigations into the synthetic potential of thionyl fluoride is the development of a facile and direct method for SOF_2_ formation. Thionyl fluoride is typically generated from thionyl chloride and a fluoride salt followed by isolation *via* condensation of the resulting gas.^15^ These methods are effective but isolation of the condensed gas is a significant practical impediment, and has likely limited studies of its reactivity. A more practical strategy is to obviate the need for isolation through *ex situ* generation and direct use of thionyl fluoride.^16^ This has been a powerful strategy for sulfuryl fluoride-based methodologies^16*a*^ but it has not yet been applied to SOF_2_. As thionyl fluoride has a similar safety profile as SO_2_F_2_,^17^ an on-demand generation approach also minimizes the safety risk associated with its handling.

*Ex situ* SOF_2_ gas generation was examined using an analogous set-up as the recent *ex situ* generation of sulfuryl fluoride.^16^ Thionyl chloride and fluoride salts^18^ were added to one reaction vessel and the resulting SOF_2_ gas was bubbled through an organic solvent in a second vessel. Unlike SO_2_F_2_ generation, we found that an imidazole trap inserted between the two reaction chambers was necessary to remove any unwanted SOClF and HCl. Our final optimized conditions^19^ were effective for creating thionyl fluoride solutions using a wide variety of solvents as determined by ^19^F NMR spectroscopy (Table 1, arranged by descending dielectric constants). While dimethyl sulfoxide (DMSO, entry 1) reacts with SOF_2_, acetonitrile (ACN) is a viable solvent and affords comparable concentrations (entry 2) as the analogous reaction with SO_2_F_2_.^20^ Lower concentrations were observed in both *N*,*N*-dimethylformamide (DMF, entry 3)^21^ and dichloromethane (DCM, entry 4). Tetrahydrofuran (THF), 1,2-dimethoxyethane (DME), and ethyl acetate (EtOAc) afforded 0.13 M, 0.11 M, and 0.15 M solutions, respectively (entries 5–7). Chloroform and toluene (Tol) performed equivalently, both yielding 0.10 M solutions (entries 8–9), but SOF_2_ was poorly soluble in the least polar solvent that was screened, petroleum ether (Pet ether, entry 10).

**Table 1:** Solubility of SOF_2_ in organic solvents^a^

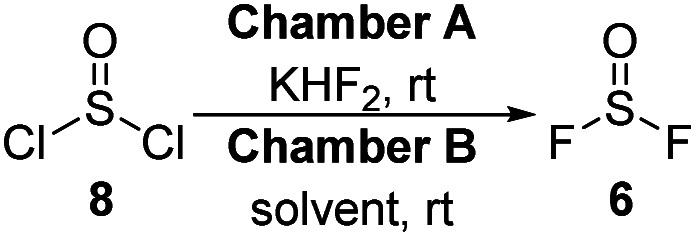		
Entry	Solvent	SOF_2_ (M)
1	DMSO	0
2	ACN	0.14
3	DMF	0.08
4	DCM	0.07
5	THF	0.13
6	DME	0.11
7	EtOAc	0.15
8	Chloroform	0.10
9	Tol	0.10
10	Pet ether	0.03

a Reaction conditions: 8 (3.0 mmol), KHF_2_ (3 equiv), solvent (6 mL), imidazole trap, 30 min. The molarity of SOF_2_ in the solvent was determined by ^19^F NMR spectroscopy using trifluorotoluene as an internal standard.

With a protocol for the *ex situ* generation of thionyl fluoride in hand, we then focused on the syntheses of peptides as it could not be readily accomplished using sulfuryl fluoride. We started our investigations by exploring the first step of this process, the direct conversion of carboxylic acids to the corresponding acyl fluoride. *meta*-Fluorobenzoic acid (1a) was selected as an initial substrate due to its simplicity and because we could use the aryl fluoride as a handle to track the reaction by ^19^F NMR spectroscopy. An initial screen found that acid fluoride 9a can be accessed using the SOF_2_-containing stock solutions from entries 2–10 (Table 1).^22^ DCM was particularly effective; treatment of 1a with a stock solution of thionyl fluoride in DCM afforded the desired product (9a) in 99% conversion after only 30 minutes at room temperature (Table 2), which is more effective than the analogous reaction using SO_2_F_2_.^23^ A direct comparison was performed under the same conditions (Fig. 2), which found that SOF_2_ promotes a higher reaction rate relative to SO_2_F_2_. This enhanced reaction rate compared to SO_2_F_2_ is consistent with literature reports describing the higher reactivity of SOF_2_.^11^ As acyl fluorides can be volatile, DCM was selected for further studies due to its low boiling point. The acyl fluorides can be isolated after extractive work-up by diluting with DCM and washing with 0.1 M NaHCO_3_ solution and brine.

**Table 2:** Formation of acyl fluorides from carboxylic acids using SOF_2_^a^

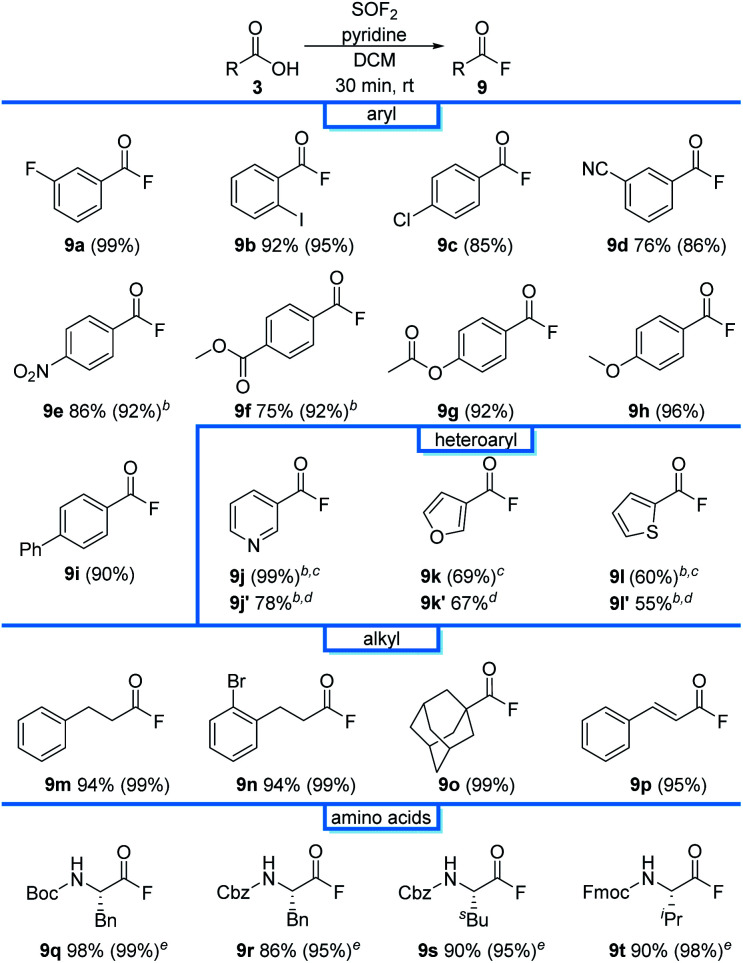

a Reaction conditions: 3 (0.6 mmol), SOF_2_ in DCM (1 equiv, approximately 0.07 M), pyridine (1 equiv), 30 min. Isolated yields for the one-pot reaction are reported, with ^19^F NMR yields using trifluorotoluene as the internal standard provided in parentheses.

b SOF_2_ in ACN was used.

c The reaction time was 1 h.

d Yields of subsequent derivatization to the corresponding *N*-hydroxyphthalimide ester. See ESI for reaction details.

e The reaction time was 20 min.

**Fig. 2:**
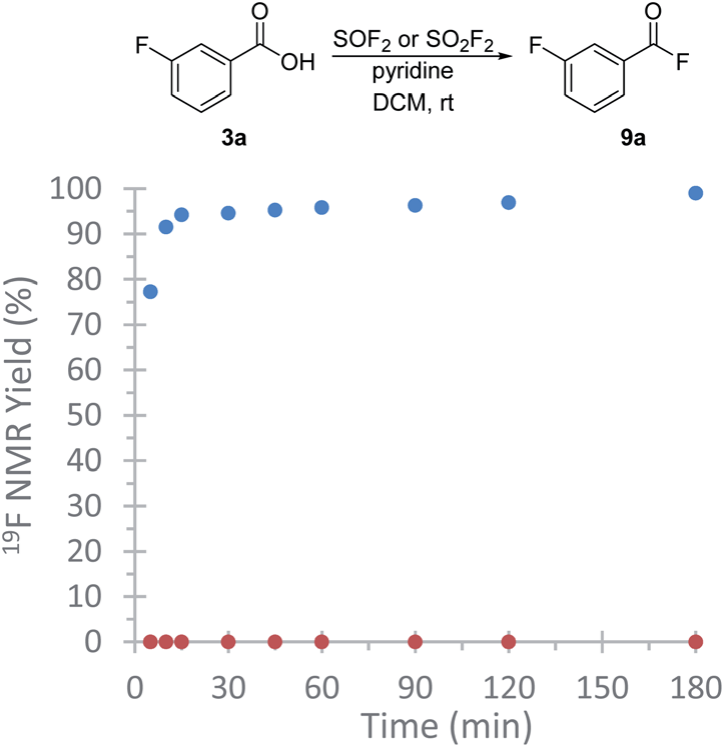
^19^F NMR spectroscopy kinetic study of the formation of 9a from 3-fluorobenzoic acid, with SOF_2_ or SO_2_F_2_. Blue = with SOF_2_; red = with SO_2_F_2_. Reactions were carried out in parallel on a 0.6 mmol scale.

The reaction was effective for a wide range of benzoic acid derivatives, affording 9b–9i in 85–96% NMR yields (Table 2). Investigations next turned to the preparation of heteroaryl acyl fluorides. Pyridine (1j), furan (1k) and thiophene (1l) were effective substrates, affording 9j–9l in 99%, 69%, and 60% NMR yields, respectively. As substrates have low boiling points and have previously been documented to be unstable out of solution,^23^ they were derivatized to the corresponding *N*-hydroxyphthalimide esters 9j′–9l′ in 78%, 67%, and 55% overall isolated yields, respectively. The reaction was also compatible with alkyl carboxylic acids, affording 9m–9p in near quantitative conversion. Boc, Cbz, and Fmoc-protecting amino acids were also viable substrates, affording 9q–9t in excellent isolated yields without the need for flash column chromatography.

Investigations next focused on one-pot peptide couplings directly from Boc protected amino acids (Table 3). Subjecting Boc-protected glycine to our optimized thionyl fluoride reaction conditions, followed by sparging with nitrogen and addition of L-Ala-O^*t*^Bu produced the desired dipeptide (10a) in 87% yield and >99 : 1 er, as determined by HPLC. Notably, the side products are readily removed by extraction and the dipeptide could be isolated pure with no column chromatography required. Alanine (Ala) was compatible with the reaction conditions to deliver 10b in 92% yield with >99 : 1 dr. For comparison, we also conducted experiments using conventional synthetic methodologies including DCC/HOBt, PyBOP, and HBTU. In all cases, the reactions took 4 hours to afford 10b in 42%, 77%, and 76% yield, respectively all with >99 : 1 dr. Notably, column chromatography was required for each of these established coupling methods.

**Table 3:** Representative scope of Boc-protected amino acids serving as electrophilic components^a^

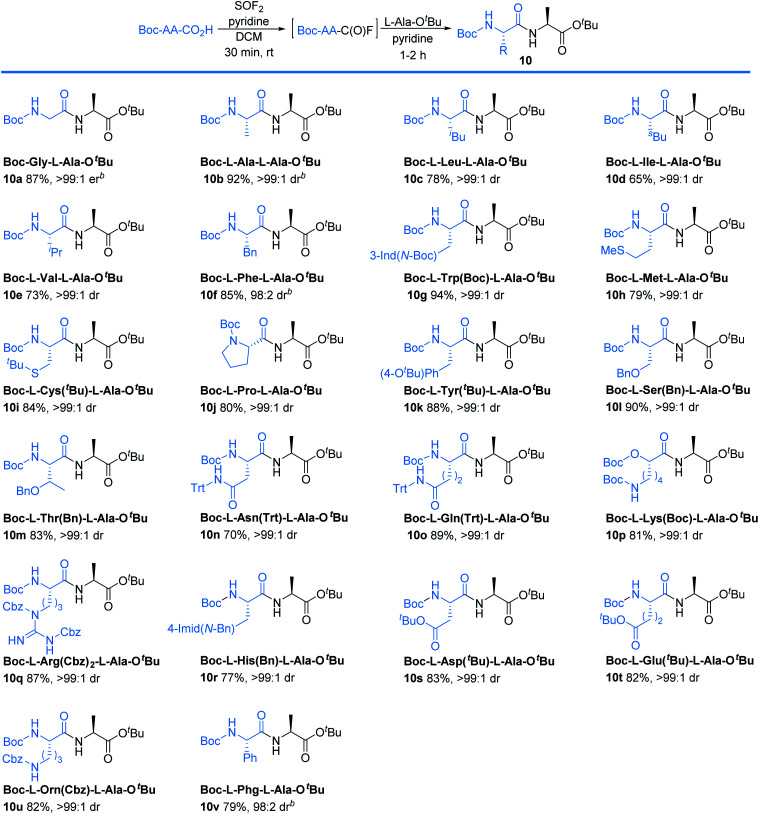

a Reaction conditions: Boc-AA-CO_2_H (0.6 mmol), SOF_2_ in DCM or ACN (1 equiv.), pyridine (1 equiv.), 30 min. Followed by L-Ala-O^*t*^Bu (1 equiv.), pyridine (1 equiv.), 1–2 h. Isolated yields are reported. Unless otherwise noted, the drs were determined by ^1^H NMR.

b The drs and ers were determined by HPLC.

Amino acids with other hydrophobic alkyl and aryl side chains, such as leucine (Leu), isoleucine (Ile), valine (Val), phenylalanine (Phe), tryptophan (Trp), and methionine (Met) were successfully coupled to produce dipeptides 10c–10h in good to excellent yields with minimal to no epimerization. This is in contrast to SO_2_F_2_-mediated amidation of amino acids, where substantial epimerization was observed.^24^ No epimerization was observed when excess SOF_2_ stock solution (1.5 equiv.) was used, suggesting that the issue of epimerization arises from the use of SO_2_F_2_ rather than the number of equivalents of SOF_2_ that were utilized. Coupling with cystine (Cys), proline (Pro), and tyrosine (Tyr) proceeded successfully to afford the desired dipeptides **10i–10k** in 84%, 80%, and 88% yields, respectively. *O*-Protected amino acid serine (Ser) was also effective, and afforded 10l in 90% yield without epimerization. Threonine (Thr) coupling afforded a slightly lower yield (83% for 10m) than serine (Ser), likely due to increased sterics. Asparagine (Asn), glutamine (Gln), lysine (Lys), arginine (Arg), histidine (His), aspartic acid (Asp), and glutamic acid (Glu) were effective in this methodology, giving excellent yields of 10n–10t with >99 : 1 dr. No evidence of cyclization was detected for any of these substrates. Compared to the current state-of-the-art for peptide coupling methodologies, our method provides comparable yields and diastereoselectivities, but with improved reaction times and simpler purification protocols (Table 4).^25^

**Table 4:** Representative examples of examining various protecting groups for peptide bond formation^a^

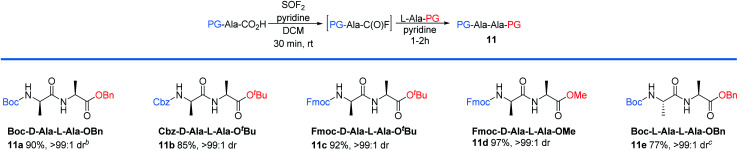

a Reaction conditions: PG-AA-CO_2_H (0.6 mmol), SOF_2_ in DCM or ACN (1 equiv.), pyridine (1 equiv.), 30 min. Followed by L-Ala-PG (1 equiv.), pyridine (1 equiv.), 1–2 h. Isolated yields are reported. Unless otherwise noted, the drs were determined by ^1^H NMR.

b The drs and ers were determined by HPLC.

c 2 gram-scale (8 mmol).

The method can also be applied for unnatural amino acids. Ornithine (Orn) was an efficient substrate, affording 10u in 82% yield. Phenylglycine (Phg) is recognized as one of the most easily racemized amino acids.^26^ Our method successfully coupled phenylglycine (Phg) to give 10v in 79% yield with 98 : 2 dr, in 2 h.

We next examined the protecting group tolerance of this new dipeptide coupling reaction (Table 4).^27^ Amino acids with *N*-Boc, *N*-Cbz, or *N*-Fmoc protecting groups effectively coupled with OBn, O^*t*^Bu, or OMe amino esters to form the corresponding dipeptides 11a–11d in excellent yields with >99 : 1 dr. Our protocol can be performed on a 2 gram-scale to generate 11e safely and with similar efficacy.

To explore the column-free, liquid phase syntheses of tri-, tetra-, penta-, and decapeptides, we designed the protocol outlined in Scheme 1. The protocol begins by first treating an N-terminal amino acid with Boc-protected amino acid fluoride, which was synthesized by our new method (step 1). Boc-protected dipeptides were obtained in 1–2 h after a simple aqueous work up. Subsequent deprotection of the Boc group was achieved with 4.0 M HCl in dioxane or TFA/DCM (1 : 1) in 1 h (step 2).^28^ Concentration *in vacuo* and neutralization afforded N-terminal peptides. Steps 1 and 2 were repeated, as necessary, for subsequent amino acid incorporation. The final coupling with the Boc-protected amino acid fluorides (step 3) afforded the desired polypeptides. This strategy was effective for the synthesis of tripeptides 12a and 12b, which were obtained in 84% and 88% yields over the three-step sequence. Tetrapeptide 12c and pentapeptide 12d were synthesized in 80% and 51% isolated yields using an analogous method as the tripeptides, except the dipeptide *N*-Boc-Leu-Gly-CO_2_H was used. Similary, a decapeptide (12e) was produced in 14% isolated yield over 8 couplings using the dipeptide *N*-Boc-Leu-Gly-CO_2_H. Notably, all of the tri-, tetra-, and pentapeptides could be assembled in a single day without the use of column chromatography.

**Scheme 1:**
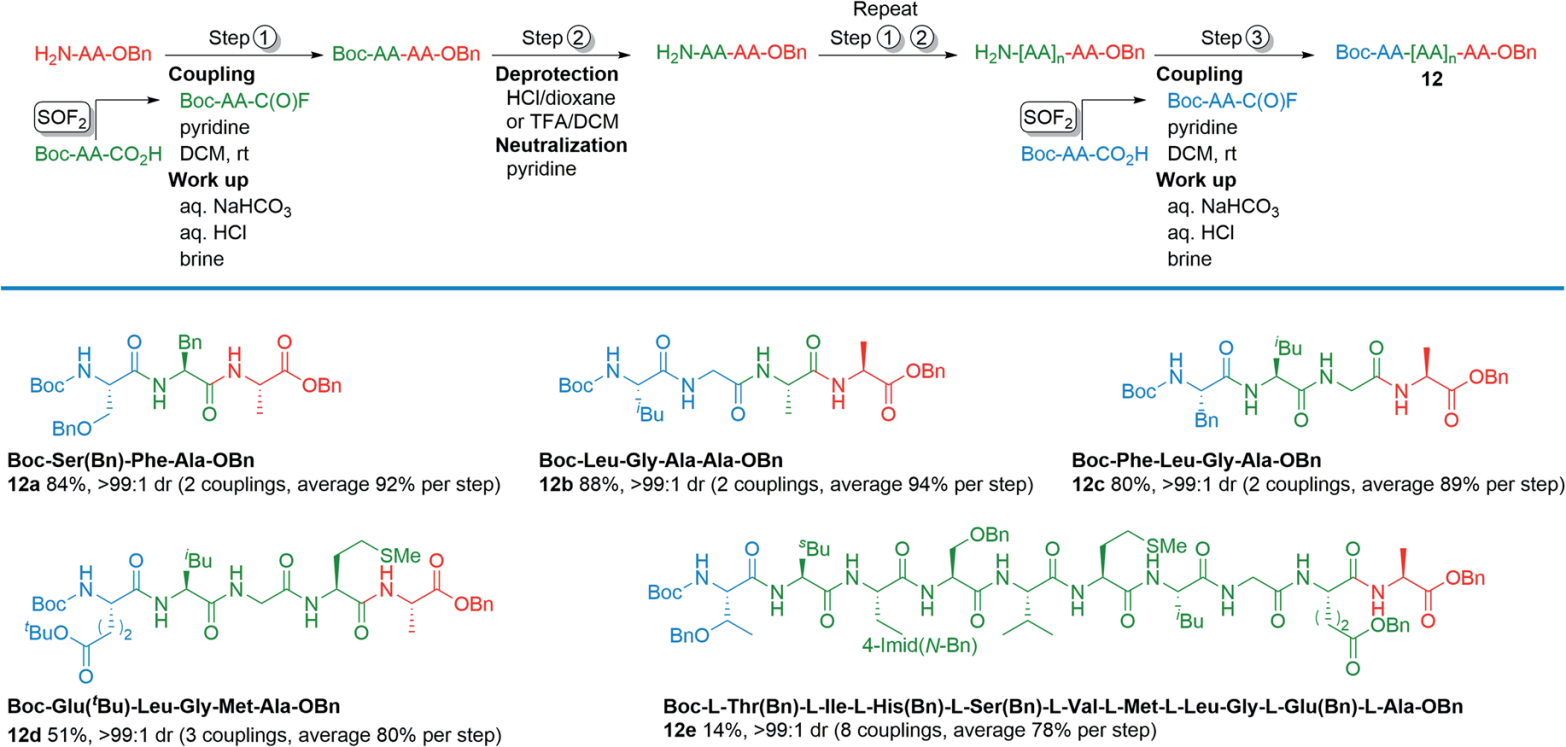
Representative examples of liquid phase peptide synthesis through acyl fluoride intermediates.

**Scheme 2:**
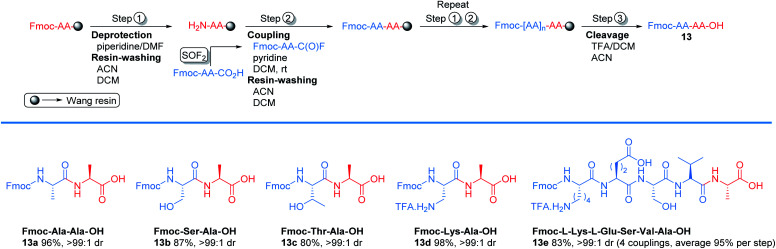
Representative example of solid phase peptide synthesis through acyl fluoride intermediate.

To explore the potential of using SOF_2_ generated amino acid fluoride in solid phase peptide synthesis (SPPS),^29^ we examined the new protocol in couplings with Wang resin (Scheme 2). We started with Fmoc-deprotection of Fmoc-Ala-Wang resin using 20% piperidine in DMF to afford free amine. The free amine was subjected to coupling with Fmoc-Ala-C(O)F to generate Fmoc-Ala-Ala-Wang resin in 1 h. After coupling, the resin was cleaved with TFA/DCM and the target Fmoc protected dipeptide 13a was obtained in 96% yield. Serine (Ser), threonine (Thr), and lysine (Lys) were compatible with this solid phase peptide synthesis and afforded the corresponding products 13b–13c with excellent yields. Pentapeptide 13e was also synthesized in 83% isolated yield.

## Conclusions

In conclusion, we have developed a novel acid activation peptide coupling strategy utilizing SOF_2_ to access acyl fluorides *via* acyl fluorosulfite intermediates. The *ex situ* generation of thionyl fluoride was achieved using inexpensive and readily available commodity chemicals, and displayed an expedited, column-free preparation of alkyl, aryl, and amino acid fluorides. Dipeptides were afforded in a one-pot, column-free protocol, effective across natural and unnatural amino acid substrates with a wide range of protecting groups and retention of optical purity. Our approach was applied to the syntheses of tri-, tetra-, and decapeptides, providing a competitive method for liquid phase, iterative peptide couplings. The new approach was also amenable to solid phase peptide synthesis.

## Data availability

The datasets supporting this article have been uploaded as part of the ESI† material.

## Author contributions

C. L. and G. M. S. conceived the project. C. L. and B. J. T. conducted and analyzed the experiments. C. L., B. J. T., and G. M. S. wrote the manuscript.

## Conflicts of interest

There are no conflicts to declare.

## Supplementary Material

SC-013-D1SC05316G-s001
