# Supplementary material for: Rapid and column-free syntheses of acyl fluorides and peptides using ex situ generated thionyl fluoride
Source: Chem Sci. 2021 Nov 29;13(1):188–94. doi: 10.1039/d1sc05316g (PMC8694322; doi:10.1039/d1sc05316g)

Electronic Supplementary Material (ESI) for Chemical Science.
This journal is © The Royal Society of Chemistry 2021

Supplementary Information for:

Rapid and Column-Free Syntheses of Acyl Fluorides and Peptides using *Ex Situ* Generated Thionyl Fluoride

Cayo Lee, Brodie J. Thomson, and Glenn M. Sammis*

Department of Chemistry, University of British Columbia, 2036 Main Mall, Vancouver, British Columbia V6T 1Z1, Canada

Contents

General methods and instrumentation S3

Optimization study of SOF_2 generation S4

General procedure A: SOF_2 generation without imidazole column S5

General procedure B: SOF_2 generation with imidazole column S6

Optimization of acyl fluoride formation S7

General procedure C: optimization of acyl fluoride formation using SOF_2 .... S8

Preparation of SOF_2 Stock solutions S8

General procedure D: large scale SOF_2 generation in solution S8

Representative example for the ^{19}F NMR yield determination S9

General procedure E: conversion of carboxylic acids to acyl fluorides using SOF_2 .... S10

General procedure F: synthesis of dipeptides through acyl fluorides using SOF_2 .... S10

General procedure G: liquid phase peptide synthesis through acyl fluoride intermediates S11

General procedure H: solid phase peptide synthesis through acyl fluoride intermediates S12

Experimental data S13

Classical synthetic methodologies in peptide synthesis S45

References S47

HPLC data S48

^{1}H, ^{13}C and ^{19}F NMR Spectra S50

General methods and instrumentation

Materials: All chemicals were from commercial sources Sigma-Aldrich, Bachem, AmBeed, AK Scientific, Alfa Aesar, Oxchem, Advanced ChemTech and Fluka. All solvents were purchased from commercial sources Sigma-Aldrich, Fisher Scientific, OmniSolv, and Acros.

All reactions were performed in disposable scintillation 20 mL (28 x 61 mm) glass vials, round-bottom flasks or Econo-Pac® chromatography columns. Screw caps and PTFE/Silicone septa (22 mm x 0.060") were from Chemglass Life Sciences. Polyethylene tubing (I.D. 1.67 mm) was from Becton Dickinson. Disposable 1 mL syringes (I.D. 4.69 mm), 3 mL Syringes (I.D. 9.65 mm), 5 mL Syringes (I.D. 12.45 mm), 10 mL Syringes (I.D. 15.90 mm), and 30 mL Syringes (I.D. 22.90 mm) were from Norm-Ject and Henk-Ject. Disposable needles 16 G x 1 ½ (1.6 mm x 40 mm) and 21 G x 2 (0.8 mm x 5 mm) were from BD PrecisionGlide™. Hypodermic Needles (22 G x 4") were from Air-Tite™. White sleeve stoppers (24 x 40 mm) were from VWR®, white sleeve stoppers (OD 14mm) were from Kimble®, and red Suba-Seal® septa were from Chemglass.

Chromatography: Thin-layer chromatography (TLC) was performed on Merck Silica gel 60 F_{254} TLC aluminium sheets and visualized with 254 nm light and $KMnO_4$ followed by heating.

Infrared spectra (IR) were obtained using a Thermo Nicolet 4700 FT-IR spectrometer or a Perkin Elmer Frontier FT-IR. The spectra are reported in cm^{-1}.

Melting points (m.p.) were determined by the open capillary method using a Mel-Temp II apparatus.

Mass spectrometry: High resolution mass spectra (HRMS) were recorded on a Waters/Micromass LCT spectrometer. Low resolution mass spectra (LRMS) data was obtained using an Agilent 5977A MSD coupled to 7890B GC.

NMR spectra were obtained on a Bruker AV-300 or AV-400 spectrometer. ^{1}H, ^{13}C, and ^{19}F NMR chemical shifts are reported in parts per million (ppm) relative to the residual solvent peak ($CDCl_3$: ^{1}H: δ = 7.26 ppm, ^{13}C: δ = 77.16 ppm). ^{19}F NMR chemical shifts were referenced to $CFCl_3$. NMR yields were determined by ^{19}F NMR using a relaxation time of 40 seconds to complete relaxation of all fluorine nuclei. α, α, α-Trifluorotoluene ($PhCF_3$) was used as an internal standard. Multiplicities are reported as follows: singlet (s), doublet (d), triplet (t), quartet (q), pentet (p), multiplet (m), doublet of doublets (dd), doublet of triplets (dt) and triplet of doublets (td). Assignment of peaks was done based on the chemical shifts, multiplicities, and integrals of the peaks. Coupling constants (J) are reported in Hz.

Optimization study of SOF_2 generation

Table S1. Solvent screen in chamber A

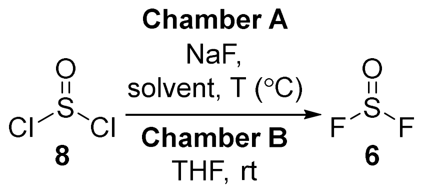

entry	solvent	T (˚C)	SOClF (M)	SOF_2 (M)
1	THF	55	0	0
2	THF	40	0	0
3	THF	RT	0	0
4	TFA	RT	0.22	0.90
5	ACN	RT	0	0.01
6	EtOAc	RT	0	0
7	Dioxane	RT	0	0
8	2-butanone	RT	0	0
9	2-pentanone	RT	0	0
10	Acetone	RT	0	0
11	DCM	RT	0	0
12	-	RT	0	0

Reaction conditions: The reactions were run on 12 mmol scale of thionyl chloride **8** following the general procedure A. The molarity of SOF_2 in THF was determined by ^{19}F NMR spectroscopy using trifluorotoluene as an internal standard.

Table S2. Fluoride source screen in chamber A

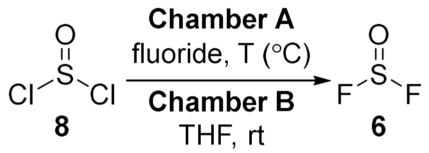

entry	fluoride	T (˚C)	SOClF (M)	SOF_2 (M)
1	NaF	RT	0	0
2	NaF	40	0	0
3	KF	RT	0.06	0.33
4	KF	40	0.10	0.40
5	KHF_2	RT	0.15	0.80
6	KHF_2	40	0.16	0.67
7	KHF_2	55	0.13	0.58

Reaction conditions: The reactions were run on 12 mmol scale of thionyl chloride **8** following the general procedure A. The molarity of SOF_2 in THF was determined by ^{19}F NMR spectroscopy using trifluorotoluene as an internal standard.

Table S3. Optimization of the amount of $SOCl_2$ in chamber A

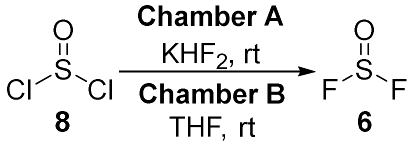

entry	$SOCl_2$ (mmol)	SOClF (M)	SOF_2 (M)
1	12	0.15	0.80
2	6	0.06	0.34
3	3	0.03	0.16

Reaction conditions: The reactions were run on 3 to 12 mmol scale of thionyl chloride **8** following the general procedure A. The molarity of SOF_2 in THF was determined by ^{19}F NMR spectroscopy using trifluorotoluene as an internal standard.

General procedure A: SOF_2 generation without imidazole column

Two 20 mL vials equipped with magnetic stir-bars were capped with septum-fitted vial caps connected by a polyethylene tube. To vial B was added THF (6 mL). To vial A was charged NaF (1.51 g, 36 mmol, 3 equiv.) and solvent (0.90 mL) followed by thionyl chloride (0.87 mL, 12 mmol, 1 equiv.) in one portion (Note: leave the syringe in vial A). The bubbling rate was slow at the beginning, then increased over 30 mins. Vial B was vented with an empty balloon when the bubbling subsided (this triggered more bubbling of SOF_2 through the solution). The tubing was removed when the bubbling subsided again. The mixture was analyzed by ^{19}F NMR spectroscopy with trifluorotoluene as internal standard.

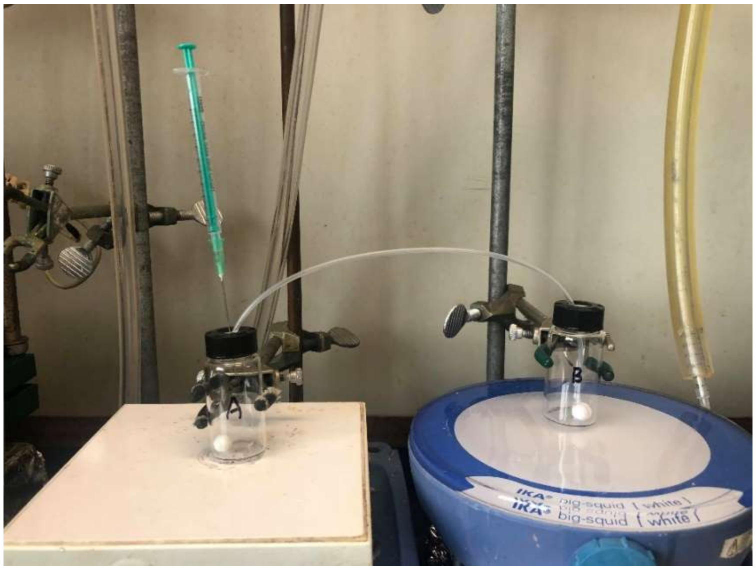

Figure S1. Setup for SOF_2 generation without imidazole column. Note: SOF_2 is a toxic gas and all work should be performed in a fume hood.

Table S4. Optimization of Imidazole column

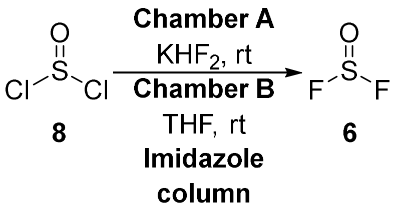

entry	imidazole filter (mmol)	SOClF (M)	SOF_2 (M)
1	-	0.03	0.16
2	10	0.01	0.11
3	40	0	0.11
4	80	0	0.07

Reaction conditions: The reactions were run on 3 mmol scale of thionyl chloride **8** following the general procedure B. The molarity of SOF_2 in THF was determined by ^{19}F NMR spectroscopy using trifluorotoluene as an internal standard.

General procedure B: SOF_2 generation with imidazole column

Two 20 mL vials equipped with magnetic stir-bars were capped with septum-fitted vial caps connected by a 6 mL syringe-filled imidazole column (2.7 g, 40 mmol) (Figure S2). To vial B was added THF (6 mL). To vial A was charged KHF_2 (0.702 g, 9 mmol, 3 equiv.) followed by thionyl chloride (0.22 mL, 3 mmol, 1 equiv.) in one portion (Note: leave the syringe in vial A). The bubbling rate was slow at the beginning, then increased over 30 mins. Vial B was vented with an empty balloon when the bubbling subsided (this triggered more bubbling of SOF_2 through the solution). The tubing was removed when the bubbling subsided again. The mixture was analyzed by ^{19}F NMR spectroscopy with trifluorotoluene as internal standard.

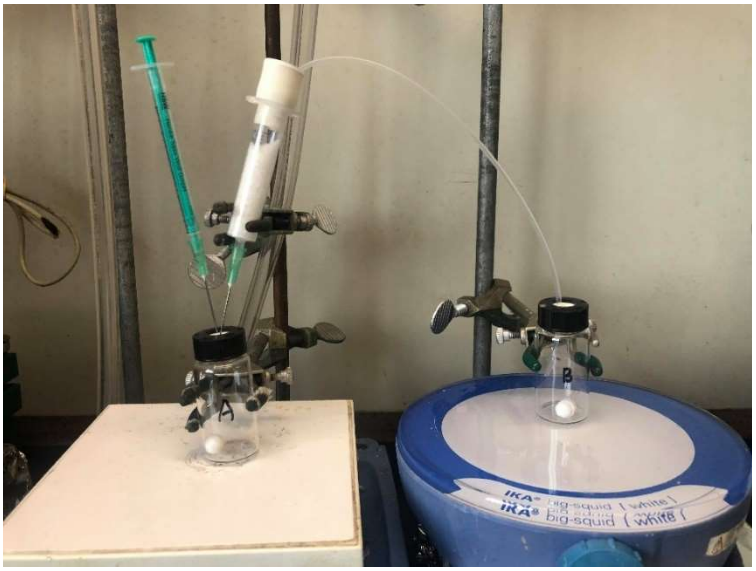

Figure S2. Setup for SOF_2 generation from $SOCl_2$ through an imidazole filter. Note: SOF_2 is a toxic gas and all work should be performed in a fume hood.

Optimization of acyl fluoride formation

Table S5. Optimization of acyl fluorides: solvent screen

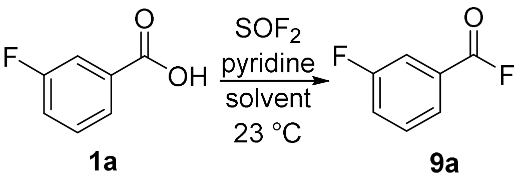

entry	solvent	Conv. (%)	yield (%)
1	DMSO	0 (0)	0 (0)
2	ACN	100 (100)	100 (100)
3	DMF	100 (91)	100 (62)
4	DCM	100 (94)	100 (87)
5	THF	86 (53)	86 (50)
6	DME	88 (65)	88 (60)
7	Ethyl acetate	85 (50)	85 (50)
8	Chloroform	54 (37)	50 (29)
9	Toluene	89 (69)	89 (52)
10	Petroleum ether	45 (21)	40 (13)

Reaction conditions: The reactions were run on 0.6 mmol scale of 3-fluorobenzoic acid **1a** following the general procedure C for 3 h. Conversions/yields after 30 min are indicated in parentheses. The yields were determined by ^{19}F NMR spectroscopy using trifluorotoluene as an internal standard.

Table S6. Optimization of acyl fluorides: base screen

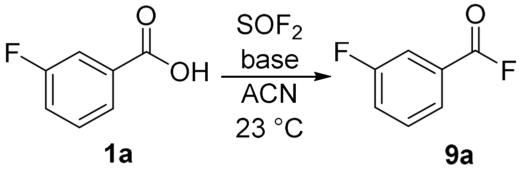

entry	Base	pka	Conv. (%)	yield (%)
1	-	-	0 (0)	0 (0)
2	DBU	12.5	100 (97)	100 (97)
3	K_2CO_3	10.25	45 (25)	13 (8)
4	Et_3N	10.6	100 (100)	100 (100)
5	DIPEA	10.6	100 (100)	100 (100)
6	NMM	7.6	99 (94)	99 (94)
7	Imidazole	6.95	96 (93)	96 (92)
8	Pyridine	5.2	100 (100)	100 (100)

Reaction conditions: The reactions were run on 0.6 mmol scale of 3-fluorobenzoic acid **1a** following the general procedure C for 3 h. Conversions/yields after 30 min are indicated in parentheses. The yields were determined by ^{19}F NMR spectroscopy using trifluorotoluene as an internal standard.

General procedure C: optimization of acyl fluoride formation using SOF_2

Two 20 mL vials equipped with magnetic stir-bars were capped with septum-fitted vial caps connected by a 6 mL syringe-filled imidazole column (Figure S2). To vial B was added 3-fluorobenzoic acid (0.084 g, 0.6 mmol, 1 equiv.), base (0.6 mmol, 1 equiv.) and solvent (6 mL). To vial A was charged KHF_2 (0.702 g, 9 mmol, 3 equiv.) followed by thionyl chloride (0.22 mL, 3 mmol, 1 equiv.) in one portion (Note: leave the syringe in vial A). The bubbling rate was slow at the beginning, then increased over 30 mins. Vial B was vented with an empty balloon when the bubbling subsided (this triggered more bubbling of SOF_2 through the solution). The tubing when the bubbling subsided again. The mixture was analyzed by ^{19}F NMR spectroscopy with trifluorotoluene as internal standard. (Note: SOF_2 is a toxic gas and all work should be performed in a fume hood.)

Preparation of SOF_2 Stock solutions

General procedure D: large scale SOF_2 generation in solution

A 20 mL vial capped with a red Suba-Seal® septum and 100 mL round bottom flask equipped with magnetic stir-bars was capped with a white septum, were connected by a 30 mL syringe-filled imidazole column (imidazole 27 g). To the round bottom flask was added DCM (60 mL). To vial was charged KHF_2 (7.02 g, 90 mmol, 3 equiv.) followed by thionyl chloride (2.2 mL, 30 mmol, 1 equiv.) in one portion (Note: leave the syringe in vial A). The bubbling rate was slow at the beginning, then increased over 30 mins. Vial B was vented with an empty balloon when the bubbling subsided (this triggered more bubbling of SOF_2 through the solution). The tubing was removed when the bubbling subsided again. The mixture was analyzed by ^{19}F NMR spectroscopy with trifluorotoluene as internal standard.

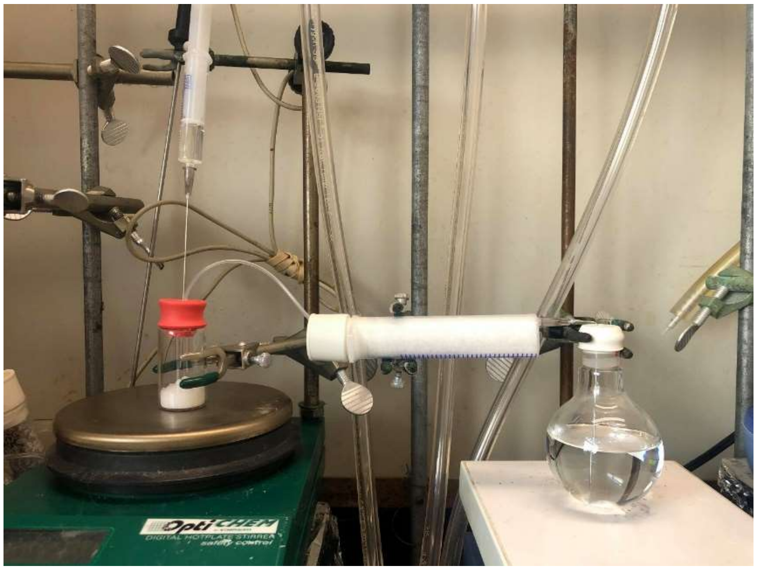

Figure S3. Setup for large scale SOF_2 generation from $SOCl_2$ through an imidazole filter. Note: SOF_2 is a toxic gas and all work should be performed in a fume hood.

Representative example for the ^{19}F NMR yield determination

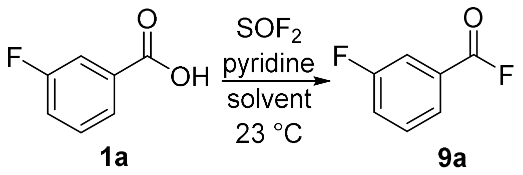

$$\left(\frac{\frac{\text{integration value of product}}{\text{number of nuclei in product}}}{\frac{\text{integration value of internal standard}}{\text{number of nuclei in internal standard}}}\right)\left(\frac{n_{\text{internal standard}}}{n_{\text{substrate}}}\right) \text{x100\%} = {}^{19}\text{F NMR yield}$$

$$\left(\frac{\frac{0.85}{1\ \text{F nuclei}}}{\frac{3.00}{3\ \text{F nuclei}}}\right)\left(\frac{\frac{0.0877\ (\text{g})}{146.11\ (\text{g/mol})}}{\frac{0.0840\ (\text{g})}{140.11\ (\text{g/mol})}}\right) \text{x100} = 85\%$$

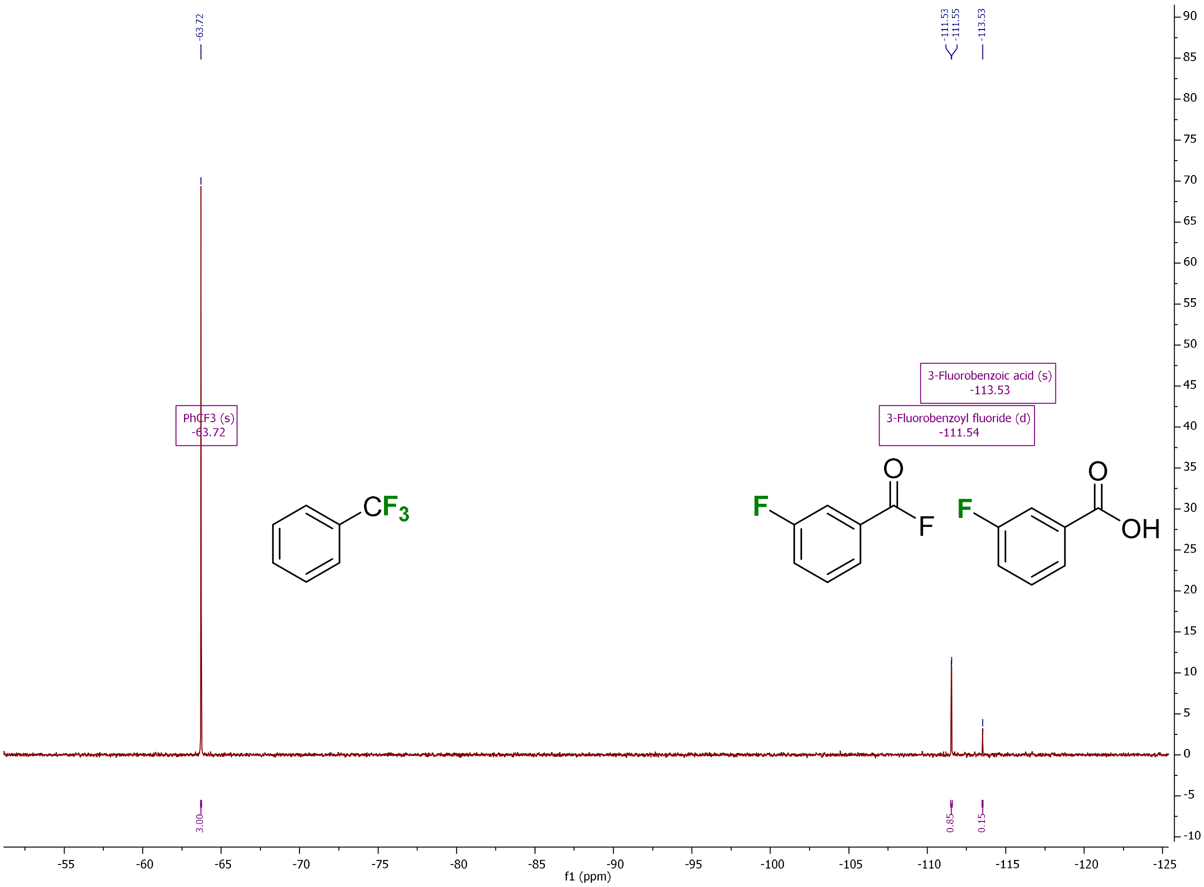

Figure S4. ^{19}F NMR spectrum yield and conversion determination for entry 9 (Table S6).

General procedure E: conversion of carboxylic acids to acyl fluorides using SOF_2

For aryl, alkyl carboxylic acids and amino acids:

A 20 mL vial equipped with a magnetic stir-bar and a septum-fitted vial cap was charged with SOF_2 stock solution in DCM or ACN (0.6 mmol, 1 equiv.), carboxylic acid (0.6 mmol, 1 equiv.) and pyridine (48 µL, 0.6 mmol, 1 equiv.). The mixture was stirred at room temperature for 30 min, unless otherwise specified. The mixture was analyzed by ^{19}F NMR spectroscopy with trifluorotoluene as internal standard. (Note: SOF_2 is a toxic gas and all work should be performed in a fume hood.)

For heteroaryl carboxylic acids (synthesis of carboxylic acid derivatives using *N*-hydroxyphthalimide):

A 20 mL vial equipped with a magnetic stir-bar and a septum-fitted vial cap was charged with SOF_2 stock solution in DCM or ACN (0.6 mmol, 1 equiv.), carboxylic acid (0.6 mmol, 1 equiv.) and pyridine (0.6 mmol, 1 equiv.). The mixture was stirred at room temperature for 1 h. The mixture was analyzed by ^{19}F NMR spectroscopy with trifluorotoluene as internal standard. A vent needle was inserted into the septum, followed by purging N_2 gas to remove remaining SOF_2 in the reaction mixture. *N*-hydroxyphthalimide (98 mg, 0.6 mmol, 1 equiv.) was added with pyridine (48 µL, 0.6 mmol, 1 equiv.) and stirred for 1 hour. (Note: SOF_2 is a toxic gas and all work should be performed in a fume hood.)

Workup: The reaction mixture was diluted with DCM (20 mL), then washed with 0.1 M $NaHCO_3$ solution (20 mL) and brine (20 mL). The organic layers were dried over Na_2SO_4 and concentrated under reduced pressure.

General procedure F: synthesis of dipeptides through acyl fluorides using SOF_2

A 20 mL vial equipped with a magnetic stir-bar and a septum-fitted vial cap was charged with SOF_2 stock solution (0.6 mmol, 1 equiv.), *N*-protected amino acid (0.6 mmol, 1 equiv.) and pyridine (48 µL, 0.6 mmol, 1 equiv.). The mixture was stirred at room temperature for 30 min. A vent needle was inserted into the septum, followed by purging N_2 gas to remove remaining SOF_2 in the reaction mixture. Amino acid ester (0.6 mmol, 1 equiv.) and pyridine (48 µL, 0.6 mmol, 1 equiv.) were added to the reaction mixture and stirred for 1-2 h.

Workup: The reaction mixture was diluted with DCM (20 mL), then washed with 0.1 M $NaHCO_3$ solution (20 mL), 1.0 M HCl (20 mL), and brine (20 mL). The organic layers were dried over Na_2SO_4 and concentrated under reduced pressure.

General procedure G: liquid phase peptide synthesis through acyl fluoride intermediates

Step 1: Peptide bond formation

A 20 mL vial equipped with a magnetic stir-bar and a septum-fitted vial cap was charged with SOF_2 stock solution (0.6 mmol, 1 equiv.), *N*-protected amino acid (0.6 mmol, 1 equiv.) and pyridine (48 µL, 0.6 mmol, 1 equiv.). The mixture was stirred at room temperature for 30 min. A vent needle was inserted into the septum, followed by purging N_2 gas to remove remaining SOF_2 in the reaction mixture. Amino acid ester (0.6 mmol, 1 equiv.) and pyridine (48 µL, 0.6 mmol, 1 equiv.) were added to the reaction mixture and stirred for 1-2 h.

Workup: The reaction mixture was diluted with DCM (20 mL), then washed with 0.1 M $NaHCO_3$ solution (20 mL), 1.0 M HCl (20 mL), and brine (20 mL). The organic layers were dried over Na_2SO_4 and concentrated under reduced pressure.

Step 2: Deprotection of Boc-protected group:

A 25-mL round-bottom flask equipped with a magnetic stir-bar was charged Boc-protected peptide and cooled to 0 ˚C. A solution of 4M HCl/dioxane (12 mL) or TFA/DCM (1:1, 12 mL) was slowly added and stirred at room temperature. After 30-60 min TLC indicated that the reaction was complete, and the reaction mixture was concentrated under reduced pressure. At 0 ˚C, the reaction mixture was neutralized by dropwise addition of pyridine (pH was adjusted to 5 by pH Test Strips, VWR Chemicals BDH®.) to afford free amine.

General procedure H: solid phase peptide synthesis through acyl fluoride intermediates

Step 1: Deprotection of Fmoc-protected group

An Econo-Pac® chromatography column equipped with Fmoc-Ala-Wang resin (0.6 mmol, 1 equiv.) was washed twice with 20% piperidine in DMF (2 × 7 mL, one wash for 5 min and one for 20 min).

Resin-washings: Washings were performed using ACN (7 mL, 2 × 5 min) and DCM (7 mL, 3 × 5 min).

Step 2: Peptide bond formation

A 20 mL vial equipped with a magnetic stir-bar and a septum-fitted vial cap was charged with SOF_2 stock solution (0.6 mmol, 1 equiv.), *N*-protected amino acid (0.6 mmol, 1 equiv.) and pyridine (48 µL, 0.6 mmol, 1 equiv.). The mixture was stirred at room temperature for 30 min. A vent needle was inserted into the septum, followed by purging N_2 gas to remove remaining SOF_2 in the reaction mixture.

Amino acid fluorides in DCM and pyridine (0.6 mmol, 1 equiv.) were transferred to the column and stirred at room temperature for 1 h.

Resin-washings: Washings were performed using ACN (7 mL, 2 × 5 min) and DCM (7 mL, 3 × 5 min).

Step 3: Cleavage

The resin was treated with 5 mL of TFA/DCM 1: 1 (v/v) for 30 min (1-2 h if sidechain deprotection is required). Then, the peptide products were filtered using ACN (5 mL). The filtrate was concentrated under reduced pressure and washed with hexanes (2 × 3 mL) to afford dipeptides.

Recrystallization: The dipeptides were recrystallized from chloroform/hexane, if necessary.

Experimental data

3-Fluorobenzoyl fluoride (9a)

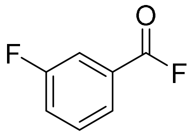

Following the general procedure E using DCM and 3-fluorobenzoic acid, 3-fluorobenzoyl fluoride (**9a**) was obtained. The yield (99 %) was determined by ^{19}F NMR of the reaction mixture. The characterization data matched previously reported data.[1]

^{19}F{^{1}H} NMR (282 MHz, Chloroform-*d*) δ 18.2 (d, *J* = 4.5 Hz), -111.8 (d, *J* = 4.6 Hz).

2-Iodobenzoyl fluoride (9b)

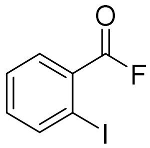

Following the general procedure E using DCM and 2-iodobenzoic acid, 2-iodobenzoyl fluoride (**9b**) was obtained. The yield (99 %) was determined by ^{19}F NMR of the reaction mixture. The product was isolated as a white solid (163 mg, 90 % yield). The characterization data matched previously reported data.[1]

M.p. 42-43 °C

^{1}H NMR (300 MHz, Chloroform-*d*) δ 8.17 – 8.10 (m, 1H), 8.05 – 7.99 (m, 1H), 7.54 – 7.46 (m, 1H), 7.35 – 7.27 (m, 1H).

^{13}C{^{1}H} NMR (75 MHz, Chloroform-*d*) δ 155.4 (d, *J* = 345.1 Hz), 142.8 (d, *J* = 3.9 Hz), 135.3, 133.6 (d, *J* = 1.9 Hz), 128.56 (d, *J* = 60.7 Hz), 128.5, 97.2.

^{19}F{^{1}H} NMR (282 MHz, Chloroform-*d*) δ 28.3.

IR (cm $^{-1}$) 3604, 3089, 1984, 1951, 1799, 1662, 1580, 1562, 1463, 1431, 1283, 1268, 1226, 1171, 1127, 1055, 1027.

LRMS-EI (m/z) 250 $[M]^{+}$.

4-Chlorobenzoyl fluoride (9c)

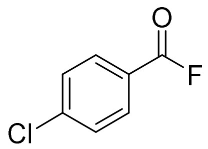

Following the general procedure E using DCM and 4-chlorobenzoic acid, 4-chlorobenzoyl fluoride (**9c**) was obtained. The yield (85 %) was determined by ^{19}F NMR of the reaction mixture. The characterization data matched previously reported data.[2]

^{19}F{^{1}H} NMR (282 MHz, Chloroform-*d*) δ 17.4.

3-Cyanobenzoyl fluoride (9d)

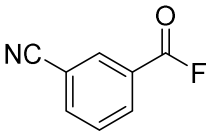

Following the general procedure E using DCM and 3-cyanobenzoic acid, 3-cyanobenzoyl fluoride (**9d**) was obtained. The yield (86 %) was determined by ^{19}F NMR of the reaction mixture. The product was isolated as a white solid (68 mg, 76 % yield). The characterization data matched previously reported data.[2]

M.p. 74-75 °C

^{1}H NMR (300 MHz, Chloroform-*d*) δ 8.35-8.32 (m, 1H), 8.31-8.25 (m, 1H), 8.02-7.96 (m, 1H), 7.75-7.67 (m, 1H).

^{13}C{^{1}H} NMR (75 MHz, Chloroform-*d*) δ 155.5 (d, *J* = 345.1 Hz), 138.3, 135.3 (d, *J* = 3.8 Hz), 134.9 (d, *J* = 3.6 Hz), 130.4 126.6 (d, *J* = 64.2 Hz), 117.1, 114.2.

^{19}F{^{1}H} NMR (282 MHz, Chloroform-*d*) δ 19.0.

IR (cm $^{-1}$) 3077, 3050, 2924, 2235, 1902, 1810, 1699, 1605, 1582, 1480, 1432, 1294, 1260, 1167, 1053, 1028.

LRMS-EI (m/z) 149 $[M]^{+}$.

4-Nitrobenzoyl fluoride (9e)

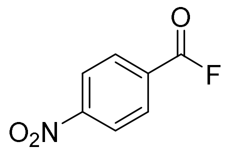

Following the general procedure E using ACN and 4-nitrobenzoic acid, 4-nitrobenzoyl fluoride (**9e**)was obtained. The yield (92 %) was determined by ^{19}F NMR of the reaction mixture. The product was isolated as a white solid (86 mg, 85 % yield). The characterization data matched previously reported data.[2]

M.p. 141-142 °C

^{1}H NMR (300 MHz, Chloroform-*d*) δ 8.42-8.36 (m, 2H), 8.29-8.22 (m, 2H).

^{13}C{^{1}H} NMR (75 MHz, Chloroform-*d*) δ 155.6 (d, *J* = 346.5 Hz), 152.0, 132.8 (d, *J* = 3.6 Hz), 130.4 (d *J* = 63.4 Hz), 124.3.

^{19}F{^{1}H} NMR (282 MHz, Chloroform-*d*) δ 20.9.

IR (cm $^{-1}$) 3115, 1814, 1725, 1609, 1532, 1441, 1347, 1321, 1278, 1233, 1161, 1105, 1030, 1006.

LRMS-EI (m/z) 169 $[M]^{+}$.

Methyl-4-(fluorocarbonyl)benzoate (9f)

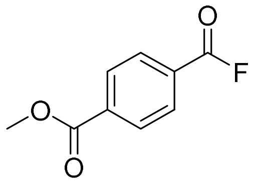

Following the general procedure E using ACN and 4-nitrobenzoic acid, 4-nitrobenzoyl fluoride (**9f**) was obtained. The yield (92 %) was determined by ^{19}F NMR of the reaction mixture. The product was isolated as a white solid (82 mg, 75 % yield). The characterization data matched previously reported data.[3]

M.p. 66-67 °C

^{1}H NMR (300 MHz, Chloroform-*d*) δ 8.20 – 8.14 (m, 2H), 8.13 – 8.08 (m, 2H), 3.96 (s, 3H).

^{13}C{^{1}H} NMR (75 MHz, Chloroform-*d*) 165.8, 156.7 (d, *J* = 346.0 Hz), 136.2, 131.5 (d, *J* = 3.9 Hz), 130.2, 128.7 (d, *J* = 61.8 Hz), 52.9.

^{19}F{^{1}H} NMR (282 MHz, Chloroform-*d*) δ 19.0.

IR (cm $^{-1}$) 3059, 2693, 2855, 1807, 1715, 1579, 1612, 1503, 1441, 1272, 1231, 1104, 1025.

LRMS-EI (m/z) 182 $[M]^{+}$.

4-Acetoxybenzoyl fluoride (9g)

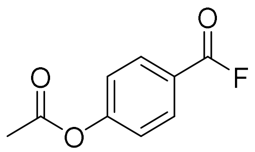

Following the general procedure E using DCM and 4-acetoxybenzoic acid, 4-acetoxybenzoyl fluoride (**9g**) was obtained. The yield (92 %) was determined by ^{19}F NMR of the reaction mixture. The characterization data matched previously reported data.[4]

^{19}F{^{1}H} NMR (282 MHz, Chloroform-*d*) δ 17.2.

4-Methoxybenzoyl fluoride (9h)

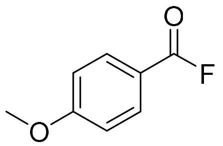

Following the general procedure E using DCM 4-methoxybenzoic acid, 4-methoxybenzoyl fluoride (**9h**) was obtained. The yield (96 %) was determined by ^{19}F NMR of the reaction mixture. The characterization data matched previously reported data.[1]

^{19}F{^{1}H} NMR (282 MHz, Chloroform-*d*) δ 14.9.

[1,1'-biphenyl]-4-carbanoyl fluoride (9i)

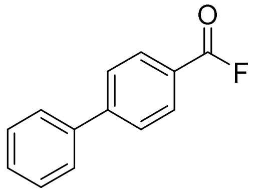

Following the general procedure E using DCM and [1,1'-biphenyl]-4-carboxylic acid, [1,1'-biphenyl]-4-carbonyl fluoride (**9i**) was obtained. The yield (90 %) was determined by ^{19}F NMR of the reaction mixture. The characterization data matched previously reported data.[5]

^{19}F{^{1}H} NMR (282 MHz, Chloroform-*d*) δ 17.0.

Nicotinoyl fluoride (9j)

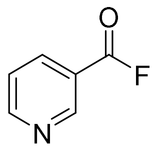

Following the general procedure E using ACN and nicotinic acid, nicotinoyl fluoride (**9j**) was obtained. The yield of nicotinoyl fluoride (99 %) was determined by ^{19}F NMR of the reaction mixture. The characterization data matched previously reported data.[1]

^{19}F{^{1}H} NMR (282 MHz, Chloroform-*d*) δ 19.6.

1,3-Dioxoisoindolin-2-yl nicotinate (9j')

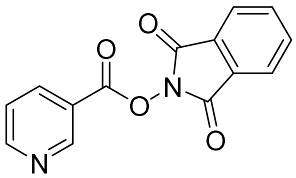

Following the general procedure E using ACN and nicotinic acid, 1,3-dioxoisoindolin-2-yl nicotinate (**9j'**) was obtained as a white solid (126 mg, 78 %). The characterization data matched previously reported data.[6,7]

M.p. 142-143 °C

^{1}H NMR (300 MHz, Chloroform-*d*) δ 9.39 (s, 1H), 9.00 – 8.82 (m, 1H), 8.62 – 8.33 (m, 1H), 8.06 – 7.89 (m, 2H), 7.89 – 7.78 (m, 2H), 7.61 – 7.42 (m, 1H).

^{13}C{^{1}H} NMR (75 MHz, Chloroform-*d*) δ 161.9, 155.2, 151.6, 138.1, 135.1, 129.0, 124.3, 123.9, 122.0.

IR (cm $^{-1}$) 3020, 1735, 1586, 1467, 1420, 1359, 1249, 1188, 1047, 1031.

LRMS-ESI (m/z) 269 $[M+H]^{+}$.

Furan-3-carbonyl fluoride (9k)

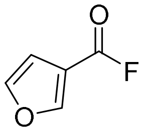

Following the general procedure E using DCM and 3-furoic acid, furan-3-carbonyl fluoride (**9k**) was obtained. The yield (69 %) of furan-3-carbonyl fluoride was determined by ^{19}F NMR of the reaction mixture. The characterization data matched previously reported data.[8]

^{19}F{^{1}H} NMR (282 MHz, Chloroform-*d*) δ 26.5.

1,3-dioxoisoindolin-2-yl furan-2-carboxylate (9k')

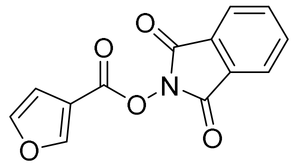

Following the general procedure E using DCM and 3-furoic acid, 1,3-dioxoisoindolin-2-yl furan-2-carboxylate (**9k'**) was obtained as a white solid (104 mg, 67 %). The characterization data matched previously reported data.[9]

M.p. 129-130 °C

^{1}H NMR (300 MHz, Chloroform-*d*) δ 8.32 – 8.29 (m, 1H), 7.96 – 7.89 (m, 2H), 7.85 – 7.78 (m, 2H), 7.64 – 7.48 (m, 1H), 7.00 – 6.81 (m, 1H).

^{13}C{^{1}H} NMR (75 MHz, Chloroform-*d*) δ 162.1, 159.0, 150.0, 144.7, 135.0, 129.1, 124.2, 114.2, 110.0.

IR (cm $^{-1}$) 3140, 1737, 1563, 1508, 1465, 1296, 1082, 1053, 1041.

LRMS-ESI (m/z) 280 [M+Na]$^+$.

Thiophene-2-carbonyl fluoride (9l)

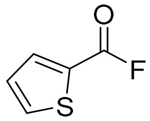

Following the general procedure E using ACN and thiophene-2-carboxylic acid, thiophene-2-carbonyl fluoride (**9l**) was obtained. The yield (60 %) of thiophene-2-carbonyl fluoride was determined by ^{19}F NMR of the reaction mixture. The characterization data matched previously reported data.[10]

^{19}F{^{1}H} NMR (282 MHz, Chloroform-*d*) δ 23.0.

1,3-Dioxoisoindolin-2-yl thiophene-2-carboxylate (9l')

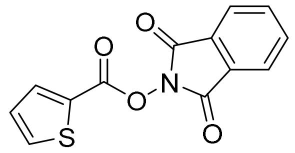

Following the general procedure E using ACN and thiophene-2-carboxylic acid, 1,3-dioxoisoindolin-2-yl thiophene-2-carboxylate (**9l'**) was obtained as a white solid (90 mg, 55 %). The characterization data matched previously reported data.[9]

M.p. 144-145 °C

^{1}H NMR (300 MHz, Chloroform-*d*) δ 8.07 (dd, *J* = 3.8, 1.3 Hz, 1H), 7.93-7.88 (m, 2H), 7.81-7.76 (m, 3H), 7.22 (dd, *J* = 5.0, 3.8 Hz, 1H).

^{13}C{^{1}H} NMR (75 MHz, Chloroform-*d*) δ 162.1, 158.4, 136.8, 135.8, 135.0, 129.0, 128.5, 127.2, 124.2.

IR (cm $^{-1}$) 3100, 1757, 1731, 1517, 1466, 1409, 1355, 1244.

LRMS-EI (m/z) 273 $[M]^{+}$.

3-Phenylpropanoyl fluoride (9m)

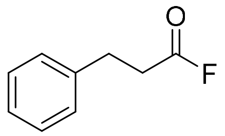

Following the general procedure E using DCM and 3-phenylpropionic acid, 3-phenylpropanoyl fluoride (**9m**) was obtained. The yield (99 %) was determined by ^{19}F NMR of the reaction mixture. The product was isolated as a colorless oil (86 mg, 94 % yield). The characterization data matched previously reported data.[11]

^{1}H NMR (300 MHz, Chloroform-*d*) δ 7.36-7.21 (m, 5H), 3.0 (t, *J* = 7.6 Hz, 2H), 2.8 (t, *J* = 7.4 Hz, 2H).

^{13}C{^{1}H} NMR (75 MHz, Chloroform-*d*) δ 162.9 (d, *J* = 360.6 Hz), 139.0, 128.9, 128.4, 127.0, 34.0 (d, *J* = 50.6 Hz), 30.1 (d, *J* = 2.3 Hz).

^{19}F{^{1}H} NMR (282 MHz, Chloroform-*d*) δ 44.9.

IR (cm $^{-1}$) 3032, 2933, 1838, 1605, 1497, 1455, 1411, 1363, 1289, 1089, 1074, 1036.

LRMS-EI (m/z) 152 $[M]^{+}$.

3-(2-Bromophenyl)propanoyl fluoride (9n)

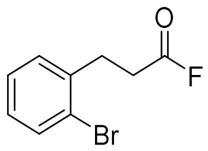

Following the general procedure E using DCM and 3-(2-bromophenyl)propionic acid, 3-(2-bromophenyl)propanoyl fluoride (**9n**) was obtained. The yield (99 %) was determined by ^{19}F NMR of the reaction mixture. The product was isolated as a colorless oil (131 mg, 94 % yield). The characterization data matched previously reported data.[2]

^{1}H NMR (300 MHz, Chloroform-*d*) δ 7.57 – 7.54 (m, 1H), 7.31 – 7.23 (m, 2H), 7.16 – 7.08 (m, 1H), 3.12 (t, *J* = 7.5 Hz, 2H), 2.86 (t, *J* = 7.5 Hz, 2H).

^{13}C{^{1}H} NMR (75 MHz, Chloroform-*d*) δ 162.8 (d, *J* = 360.3 Hz), 138.3, 133.2, 130.8, 128.8, 128.0, 124.3, 32.2 (d, *J* = 51.2 Hz), 30.7 (d, *J* = 30.7 Hz).

^{19}F{^{1}H} NMR (282 MHz, Chloroform-*d*) δ 44.9.

IR (cm $^{-1}$) 2941, 1838, 1569, 1473, 1442, 1410, 1369, 1293, 1205, 1121, 1085, 1027.

LRMS-EI (m/z) 230 [M]$^{+}$.

Adamantane-1-carbonyl fluoride (9o)

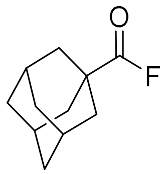

Following the general procedure E using DCM and 1-adamantane carboxylic acid, adamantane-1-carbonyl fluoride (**9o**) was obtained. The yield (99 %) was determined by ^{19}F NMR of the reaction mixture. The characterization data matched previously reported data.[1]

^{19}F{^{1}H} NMR (282 MHz, Chloroform-*d*) δ 22.9.

Cinnamoyl fluoride (9p)

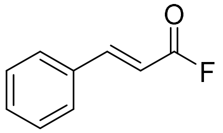

Following the general procedure E using DCM and *trans*-cinnamic acid, cinnamoyl fluoride (**9p**) was obtained. The yield (95 %) was determined by ^{19}F NMR of the reaction mixture. The characterization data matched previously reported data.[1]

^{19}F{^{1}H} NMR (282 MHz, Chloroform-*d*) δ 24.2.

Boc-L-Phe-F (9q)

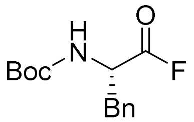

Following the general procedure E using DCM and Boc-L-Phe-OH, Boc-L-Phe-F (**9q**) was obtained as a white solid (158 mg, 98% yield). The characterization data matched previously reported data.[12,13]

M.p. 68–69 ˚C

$[\alpha]_D^{24}$ = –28.1 (*c* 1.00, EtOAc)

^{1}H NMR (300 MHz, Chloroform-*d*) δ 7.39 – 7.28 (m, 3H, BocNHCHCH$_2$C$_6$***H***$_5$), 7.21 – 7.15 (m, 2H, BocNHCHCH$_2$C$_6$***H***$_5$), 4.83 (s, 1H, BocN***H***), 4.75 (s, 1H, BocNHC***H***), 3.17 (d, *J* = 5.2 Hz, 2H, BocNHCHC***H***$_2$Ph), 1.42 (s, 9H, (C***H***$_3$)$_3$COCONH).

^{13}C{^{1}H} NMR (75 MHz, Chloroform-*d*) δ 162.3 (d, *J* = 371.4 Hz), 155.0, 134.6, 129.3, 129.2, 127.8, 81.0, 53.5 (d, *J* = 59.4 Hz), 37.1, 28.3.

^{19}F{^{1}H} NMR (282 MHz, Chloroform-*d*) δ 30.3.

IR (cm^{-1}): 3381, 2993, 2976, 2933, 1835, 1783, 1681, 1512, 1445, 1367, 1287, 1251, 1160, 1118.

LRMS-EI (m/z) calculated for $C_{14}H_{18}FNO_3$ $[M]^+$ 267.1, found 267.3.

Cbz-L-Phe-F (9r)

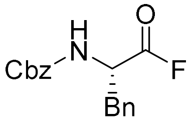

Following the general procedure E using DCM and Cbz-L-Phe-OH, Cbz-L-Phe-F (**9r**) was obtained as a white solid (156 mg, 86% yield). The characterization data matched previously reported data.[12]

M.p. 85–86 ˚C

$[\alpha]_D^{24}$ = –33.9 (*c* 1.00,)

^{1}H NMR (300 MHz, Chloroform-*d*) δ 7.42 – 7.10 (m, 11H, CbzNHCHCH$_2$C$_6$***H***$_5$, C$_6$***H***$_5$CH$_2$OCONH, and CbzN***H***), 5.11 (s, 2H, PhC***H***$_2$OCO), 4.90 – 4.76 (m, 1H, CbzNHC***H***), 3.19 (d, *J* = 5.9 Hz, 2H, CbzNHCHC***H***$_2$Ph).

^{13}C{^{1}H} NMR (75 MHz, Chloroform-*d*) δ 161.9 (d, *J* = 370.1 Hz), 155.6, 135.9, 134.2, 129.3, 129.2, 128.7, 128.6, 128.3, 128.0, 67.6, 53.9 (d, *J* = 60.3 Hz), 37.0.

^{19}F{^{1}H} NMR (282 MHz, Chloroform-*d*) δ 30.3.

IR (cm^{-1}): 3412, 3324, 3064, 3032, 2954, 1843, 1702, 1519, 1452, 1343, 1258, 1114, 1079, 1051.

LRMS-EI (m/z) calculated for $C_{17}H_{16}FNO_3$ $[M]^+$ 301.1, found 301.1.

Cbz-L-Ile-F (9s)

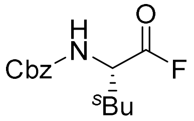

Following the general procedure E using DCM and Cbz-L-Ile-OH, Cbz-L-Ile-F (**9s**) was obtained as a colorless oil (144 mg, 90% yield). The characterization data matched previously reported data.[14]

$[\alpha]_D^{26}$ = +11.7 (*c* 1.00, EtOAc)

^{1}H NMR (300 MHz, Chloroform-*d*) δ 7.35 (d, *J* = 3.1 Hz, 5H, C$_6$***H***$_5$CH$_2$OCONH), 5.27 – 5.06 (m, 3H, C$_6$H$_5$C***H$_2$***OCONH and CbzN***H***), 4.52 (dd, *J* = 8.6, 4.6 Hz, 1H, CbzNHC***H***), 2.06 – 1.88 (m, 1H, CHC***H***(CH$_3$)CH$_2$CH$_3$), 1.55 – 1.40 (m, 1H, CHCH(CH$_3$)C***H***$_2$CH$_3$), 1.32 – 1.16 (m, 1H, CHCH(CH$_3$)C***H***$_2$CH$_3$), 1.02 (d, *J* = 6.8 Hz, 3H, CHCH(C***H***$_3$)CH$_2$CH$_3$), 0.95 (t, *J* = 7.4 Hz, 3H, CHCH(CH$_3$)CH$_2$C***H***$_3$).

^{13}C{^{1}H} NMR (75 MHz, Chloroform-*d*) δ 162.5 (d, *J* = 372.5 Hz), 156.1, 135.9, 128.8, 128.5, 128.4, 67.7, 57.7 (d, *J* = 57.5 Hz), 37.3, 25.1, 15.6, 11.6.

^{19}F{^{1}H} NMR (282 MHz, Chloroform-*d*) δ 34.6.

IR (cm^{-1}): 3324, 3032, 2969, 2937, 2881, 1843, 1702, 1526, 1455, 1255, 1082, 1040.

LRMS-EI (m/z) calculated for $C_{14}H_{18}FNO_3$ $[M]^+$ 267.1, found 267.2.

Fmoc-L-Val-F (9t)

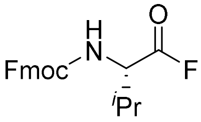

Following the general procedure E using DCM and Fmoc-L-Val-OH, Fmoc-L-Val-F (**9t**) was obtained as a white solid (185 mg, 90% yield). The characterization data matched previously reported data.[15,16]

M.p. 111–112 ˚C

[α]$_D$^{25} = +10.7 (*c* 1.00, CH_2Cl_2)

^{1}H NMR (300 MHz, Chloroform-*d*) δ 7.77 (d, *J* = 7.6 Hz, 2H, $C_{12}\underline{\boldsymbol{H}}_8CHCH_2OCONH$), 7.59 (d, *J* = 7.4 Hz, 2H, $C_{12}\underline{\boldsymbol{H}}_8CHCH_2OCONH$), 7.41 (t, *J* = 7.4 Hz, 2H, $C_{12}\underline{\boldsymbol{H}}_8CHCH_2OCONH$), 7.32 (t, *J* = 7.4 Hz, 2H, $C_{12}\underline{\boldsymbol{H}}_8CHCH_2OCONH$), 5.16 (d, *J* = 9.0 Hz, 1H, FmocN$\underline{\boldsymbol{H}}$), 4.47 (d, *J* = 6.9 Hz, 3H, $C_{12}H_8CHC\underline{\boldsymbol{H}}_2OCONH$, FmocNHC$\underline{\boldsymbol{H}}$), 4.23 (t, *J* = 6.7 Hz, 1H, $C_{12}H_8C\underline{\boldsymbol{H}}$), 2.27 (dq, *J* = 12.8, 6.5 Hz, 1H, FmocNHCHC$\underline{\boldsymbol{H}}(CH_3)_2$), 1.04 (d, *J* = 6.9 Hz, 3H, FmocNHCHCH$(C\underline{\boldsymbol{H}}_3)_2$), 1.00 (d, *J* = 6.9 Hz, 3H, FmocNHCHCH$(C\underline{\boldsymbol{H}}_3)_2$).

^{13}C{^{1}H} NMR (75 MHz, Chloroform-*d*) δ 162.49 (d, *J* = 371.4 Hz), 156.14, 143.78, 143.67, 141.48, 127.95, 127.26, 125.09, 120.19, 67.45, 58.29 (d, *J* = 57.6 Hz), 47.27, 30.55 (d, *J* = 1.9 Hz), 18.94, 17.71.

^{19}F{^{1}H} NMR (282 MHz, Chloroform-*d*) δ 33.5.

IR (cm^{-1}): 3413, 3329, 3069, 2968, 1840, 1706, 1517, 1446, 1313, 1266, 1232, 1081, 1027.

LRMS-EI (m/z) calculated for $C_{20}H_{20}FNO_3$ $[M]^+$ 341.1, found 341.1.

Boc-Gly-L-Ala-O^{t}Bu (10a)

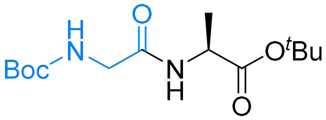

Following the general procedure (Method F) using Boc-Gly-OH and L-Ala-O^{t}Bu, Boc-Gly-L-Ala-O^{t}Bu (**10a**) was obtained as a colorless oil (157 mg, 87% yield) with 99:1 er. The characterization data matched previously reported data.[17]

[α]$_D$^{23} = +11.2 (*c* 1.00, $CHCl_3$)

^{1}H NMR (300 MHz, Chloroform-*d*) δ 6.58 (d, *J* = 7.4 Hz, 1H, N$\underline{\boldsymbol{H}}$), 5.13 (s, 1H, BocN$\underline{\boldsymbol{H}}CH_2$), 4.46 (p, *J* = 7.2 Hz, 1H, C$\underline{\boldsymbol{H}}CH_3$), 3.80 (s, 2H, BocNHC$\underline{\boldsymbol{H}}_2$), 1.46 (s, 9H, $CO_2C(C\underline{\boldsymbol{H}}_3)_3$), 1.45 (s, 9H, $(C\underline{\boldsymbol{H}}_3)_3COCONHCH_2$), 1.37 (d, *J* = 7.1 Hz, 3H, CHC$\underline{\boldsymbol{H}}_3$).

^{13}C{^{1}H} NMR (75 MHz, Chloroform-*d*) δ 172.1, 168.9, 156.0, 82.3, 80.4, 48.7, 44.4, 28.4, 28.1, 18.8.

IR (cm^{-1}): 3313, 2979, 2933, 1723, 1667, 1519, 1455, 1367, 1248, 1149.

HRMS-ESI (m/z) calculated for $C_{14}H_{26}N_2O_5Na$ $[M+Na]^+$ 325.1739, found 325.1730.

Boc-L-Ala-L-Ala-O^{t}Bu (10b)

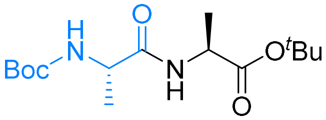

Following the general procedure (Method F) using Boc-L-Ala-OH and L-Ala-O^{t}Bu, Boc-L-Ala-L-Ala-O^{t}Bu (**10b**) was obtained as a white solid (175 mg, 92% yield) with 99:1 dr. The characterization data matched previously reported data.[17,18]

M.p. 49–50 ˚C

[α]$_D$^{24} = –16.1 (*c* 1.00, $CHCl_3$)

^{1}H NMR (300 MHz, Chloroform-*d*) δ 6.57 (d, *J* = 7.4 Hz, 1H, N***H***), 5.02 (s, 1H, BocN***H***$CHCH_3$), 4.42 (p, *J* = 7.2 Hz, 1H, C***H***CH_3), 4.15 (s, 1H, BocNHC***H***CH_3), 1.45 (s, 9H, $CO_2C(C\boldsymbol{H}_3)_3$), 1.44 (s, 9H, $(C\boldsymbol{H}_3)_3COCONHCHCH_3$), 1.37 (d, *J* = 7.1 Hz, 3H, $BocNHCHC\boldsymbol{H}_3$), 1.34 (d, *J* = 7.1 Hz, 3H, $CHC\boldsymbol{H}_3$).

^{13}C{^{1}H} NMR (75 MHz, Chloroform-*d*) δ 172.1, 172.1, 155.5, 82.1, 80.2, 50.2, 48.8, 28.4, 28.1, 18.7, 18.7.

IR (cm^{-1}): 3296, 2979, 2933, 1716, 1663, 1529, 1452, 1364, 1244, 1156, 1023.

HRMS-ESI (m/z) calculated for $C_{15}H_{28}N_2O_5Na$ $[M+Na]^+$ 339.1896, found 339.1893.

Boc-L-Leu-L-Ala-O^{t}Bu (10c)

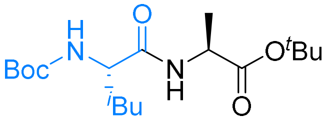

Following the general procedure (Method F) using Boc-L-Leu-OH and L-Ala-O^{t}Bu, Boc-L-Leu-L-Ala-O^{t}Bu (**10c**) was obtained as a white solid (169 mg, 78% yield) with 99:1 dr. The characterization data matched previously reported data.[17]

M.p. 87–89 ˚C

[α]$_D$^{24} = –22.6 (*c* 1.00, $CHCl_3$)

^{1}H NMR (300 MHz, Chloroform-*d*) δ 6.57 (d, *J* = 7.4 Hz, 1H, N***H***), 4.92 (d, *J* = 7.6 Hz, 1H, BocN***H***$CHCH_2CH(CH_3)_2$), 4.42 (p, *J* = 7.2 Hz, 1H, C***H***CH_3), 4.20 – 4.02 (m, 1H, BocNHC***H***$CH_2CH(CH_3)_2$), 1.87 – 1.56 (m, 3H, $BocNHCHC\boldsymbol{H}_2C\boldsymbol{H}(CH_3)_2$), 1.45 (s, 9H, $CO_2C(C\boldsymbol{H}_3)_3$), 1.43 (s, 9H, $(C\boldsymbol{H}_3)_3COCONHCHCH_2CH(CH_3)_2$), 1.35 (d, *J* = 7.1 Hz, 3H, $CHC\boldsymbol{H}_3$), 0.94 (d, *J* = 6.3 Hz, 3H $BocNHCHCH_2CH(C\boldsymbol{H}_3)_2$), 0.92 (d, *J* = 6.2 Hz, 3H, $BocNHCHCH_2CH(C\boldsymbol{H}_3)_2$).

^{13}C{^{1}H} NMR (75 MHz, Chloroform-*d*) δ 172.0, 172.0, 155.7, 82.1, 80.1, 53.2, 48.8, 41.6, 28.4, 28.1, 24.8, 23.1, 22.1, 18.7.

IR (cm^{-1}): 3299, 2979, 2933, 1740, 1684, 1660, 1533, 1364, 1290, 1251, 1160.

HRMS-ESI (m/z) calculated for $C_{18}H_{34}N_2O_5Na$ $[M+Na]^+$ 381.2365, found 381.2356.

Boc-L-Ile-L-Ala-O^{t}Bu (10d)

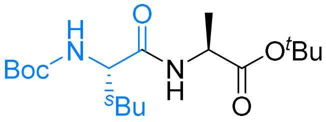

Following the general procedure (Method F) using Boc-L-Ile-OH and L-Ala-O^{t}Bu, Boc-L-Ile-L-Ala-O^{t}Bu (**10d**) was obtained as a white solid (139 mg, 65% yield) with 99:1 dr. The characterization data matched previously reported data.[18]

M.p. 112–113 ˚C

[α]$_D$^{19} = –10.4 (*c* 0.80, $CHCl_3$)

^{1}H NMR (300 MHz, Chloroform-*d*) δ 6.40 (d, *J* = 6.6 Hz, 1H, N***H***), 5.05 (d, *J* = 8.9 Hz, 1H, BocN***H***CHCH(CH$_3$)CH$_2$CH$_3$), 4.44 (p, *J* = 7.2 Hz, 1H, C***H***CH$_3$), 3.94 (t, *J* = 7.4 Hz, 1H, BocNHC***H***CH(CH$_3$)CH$_2$CH$_3$), 1.95 – 1.74 (m, 1H, BocNHCHC***H***(CH$_3$)CH$_2$CH$_3$), 1.57 – 1.47 (m, 1H, BocNHCHCH(CH$_3$)C***H***$_2$CH$_3$), 1.45 (s, 9H, CO$_2$C(C***H***$_3$)$_3$), 1.43 (s, 9H, (C***H***$_3$)$_3$COCONHCHCH(CH$_3$)CH$_2$CH$_3$), 1.36 (d, *J* = 7.1 Hz, 3H, CHC***H***$_3$), 1.24 – 1.05 (m, 1H, BocNHCHCH(CH$_3$)C***H***$_2$CH$_3$), 0.93 (d, J = 6.9 Hz, 3H, BocNHCHCH(C***H***$_3$)CH$_2$CH$_3$), 0.90 (t, J = 7.3 Hz, 3H, BocNHCHCH(CH$_3$)CH$_2$C***H***$_3$).

^{13}C{^{1}H} NMR (75 MHz, Chloroform-*d*) δ 172.0, 170.9, 155.8, 82.1, 80.0, 59.4, 48.8, 37.6, 28.4, 28.1, 24.8, 18.7, 15.7, 11.6.

IR (cm^{-1}): 3317, 2976, 2934, 1698, 1656, 1502, 1455, 1367, 1248, 1149.

HRMS-ESI (m/z) calculated for $C_{18}H_{34}N_2O_5Na$ $[M+Na]^+$ 381.2365, found 381.2362.

Boc-L-Val-L-Ala-O^{t}Bu (10e)

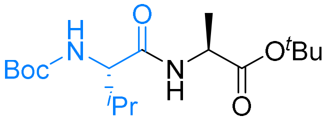

Following the general procedure (Method F) using Boc-L-Val-OH and L-Ala-O^{t}Bu, Boc-L-Val-L-Ala-O^{t}Bu (**10e**) was obtained as a white solid (151 mg, 73% yield) with 99:1 dr. The characterization data matched previously reported data. [17]

M.p. 97–98 ˚C

[α]$_D$^{24} = –6.7 (*c* 1.17, $CHCl_3$)

^{1}H NMR (300 MHz, Chloroform-*d*) δ 6.37 (d, *J* = 6.2 Hz, 1H, N***H***), 5.05 (d, *J* = 8.6 Hz, 1H, BocN***H***CHCH(CH$_3$)$_2$), 4.44 (p, *J* = 7.2 Hz, 1H, C***H***CH$_3$), 3.92 (t, *J* = 7.6 Hz, 1H, BocNHC***H***CH(CH$_3$)$_2$), 2.21 – 2.06 (m, 1H, , BocNHCHC***H***(CH$_3$)$_2$), 1.46 (s, 9H, CO$_2$C(C***H***$_3$)$_3$), 1.44 (s, 9H, (C***H***$_3$)$_3$COCONHCHCH(CH$_3$)$_2$), 1.37 (d, *J* = 7.1 Hz, 3H, CHC***H***$_3$), 0.96 (d, *J* = 6.8 Hz, 3H, BocNHCHCH(C***H***$_3$)$_2$), 0.91 (d, *J* = 6.8 Hz, 3H, BocNHCHCH(C***H***$_3$)$_2$).

^{13}C{^{1}H} NMR (75 MHz, Chloroform-*d*) δ 172.0, 171.0, 155.9, 82.2, 80.0, 59.9, 48.8, 31.2, 28.4, 28.1, 19.4, 18.7, 17.8.

IR (cm^{-1}): 3310, 2976, 2933, 1737, 1680, 1653, 1523, 1368, 1298, 1244, 1149.

HRMS-ESI (m/z) calculated for $C_{17}H_{32}N_2O_5Na$ $[M+Na]^+$ 367.2209, found 367.2201.

Boc-L-Phe-L-Ala-O*t*Bu (10f)

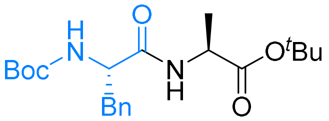

Following the general procedure (Method F) using Boc-L-Phe-OH and L-Ala-O*t*Bu, Boc-L-Phe-L-Ala-O*t*Bu (**10f**) was obtained as a white solid (199 mg, 85% yield) with 98:2 dr. The characterization data matched previously reported data.[17,19]

M.p. 84–86 ˚C

$[\alpha]_D^{24}$ = –18.3 (*c* 1.00, MeOH)

^{1}H NMR (300 MHz, Chloroform-*d*) δ 7.36 – 7.15 (m, 5H, BocNHCHCH$_2$C$_6$***H***$_5$), 6.44 (d, *J* = 7.1 Hz, 1H, N***H***), 4.99 (s, 1H, BocN***H***CHCH$_2$C$_6$H$_5$), 4.47 – 4.22 (m, 2H, BocNHC***H***CH$_2$C$_6$H$_5$ and C***H***CH$_3$), 3.10 – 3.02 (m, 2H, BocNHCHC***H***$_2$C$_6$H$_5$), 1.44 (s, 9H, CO$_2$C(C***H***$_3$)$_3$), 1.40 (s, 9H, (C***H***$_3$)$_3$COCONHCHCH$_2$Ph), 1.31 (d, *J* = 7.1 Hz, 3H, CHC***H***$_3$).

^{13}C{^{1}H} NMR (75 MHz, Chloroform-*d*) δ 171.8, 170.6, 155.4, 136.6, 129.5, 128.7, 127.1, 82.1, 80.3, 55.8, 48.9, 38.6, 28.4, 28.0, 18.7.

IR (cm^{-1}): 3296, 2976, 2930, 1737, 1681, 1660, 1529, 1366, 1248, 1153, 1047.

HRMS-ESI (m/z) calculated for $C_{21}H_{32}N_2O_5Na$ $[M+Na]^+$ 415.2209, found 415.2201.

Boc-L-Trp(Boc)-L-Ala-O*t*Bu (10g)

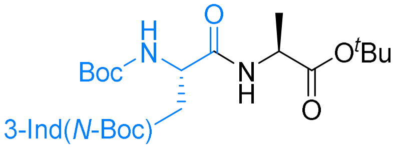

Following the general procedure (Method F) using Boc-L-Trp(Boc)-OH and L-Ala-O*t*Bu, Boc-L-Trp(Boc)-L-Ala-O*t*Bu (**10g**) was obtained as a white solid (300 mg, 94% yield) with 99:1 dr.

M.p. 78–80 ˚C

$[\alpha]_D^{24}$ = +6.8 (*c* 1.00, CHCl$_3$)

^{1}H NMR (300 MHz, Chloroform-*d*) δ 8.12 (d, *J* = 8.2 Hz, 1H, BocNHCHCH$_2$***IndH***), 7.59 (d, *J* = 7.7 Hz, 1H, BocNHCHCH$_2$***IndH***), 7.45 (s, 1H, BocNHCHCH$_2$***IndH***), 7.34 – 7.28 (m, 1H, BocNHCHCH$_2$***IndH***), 7.25 – 7.19 (m, 1H, BocNHCHCH$_2$***IndH***), 6.46 (d, *J* = 7.0 Hz, 1H, N***H***)), 5.08 (s, 1H, BocN***H***CHCH$_2$Ind), 4.52 – 4.37 (m, 1H, BocNHC***H***CH$_2$Ind), 4.31 (p, *J* = 7.1 Hz, 1H, C***H***CH$_3$), 3.32 – 3.18 (m, 1H, BocNHCHC***H***$_2$Ind), 3.13 (dd, *J* = 14.4,

6.6 Hz, 1H, BocNHCHC***H***$_2$Ind), 1.65 (s, 9H, IndCO$_2$C(C***H***$_3$)$_3$), 1.41 (s, 18H, (C***H***$_3$)$_3$COCONHCHCH$_2$Ind and CO$_2$C(C***H***$_3$)$_3$), 1.27 (d, *J* = 7.1 Hz, 3H, CHC***H***$_3$).

^{13}C{^{1}H} NMR (75 MHz, Chloroform-*d*) δ 171.6, 170.6, 155.4, 149.7, 135.6, 130.5, 124.7, 124.5, 122.8, 119.2, 115.5, 115.4, 83.7, 82.1, 80.3, 54.6, 48.9, 28.4, 28.3, 28.1, 28.0, 18.7.

IR (cm^{-1}): 3303, 2979, 2933, 1734, 1695, 1656, 1533, 1452, 1368, 1255, 1153.

HRMS-ESI (m/z) calculated for $C_{28}H_{41}N_3O_7Na$ [M+Na]$^+$ 554.2842, found 554.2840.

Boc-L-Met-L-Ala-O*t*Bu (10h)

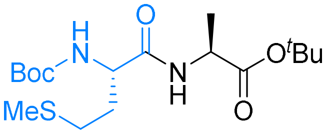

Following the general procedure (Method F) using Boc-L-Met-OH and L-Ala-O*t*Bu, Boc-L-Met-L-Ala-O*t*Bu (**10h**) was obtained as a colorless liquid (179 mg, 79% yield) with 99:1 dr. The characterization data matched previously reported data. The characterization data matched previously reported data.[18]

[α]$_D^{24}$ = +0.10 (*c* 0.50, CHCl$_3$)

^{1}H NMR (300 MHz, Chloroform-*d*) δ 6.86 (d, *J* = 7.3 Hz, 1H, N***H***), 5.43 (d, *J* = 7.2 Hz, 1H, BocN***H***CHCH$_2$CH$_2$SCH$_3$), 4.36 (p, *J* = 7.2 Hz, 1H, C***H***CH$_3$), 4.26 (q, *J* = 7.5 Hz, 1H, BocNHC***H***CH$_2$CH$_2$SCH$_3$), 2.52 (t, *J* = 7.3 Hz, 2H, BocNHCHCH$_2$C***H***$_2$SCH$_3$), 2.04 (s, 3H, BocNHCHCH$_2$CH$_2$SC***H***$_3$), 2.03 – 1.95 (m, 1H, BocNHCHC***H***$_2$CH$_2$SCH$_3$), 1.93 – 1.80 (m, 1H, BocNHCHC***H***$_2$CH$_2$SCH$_3$), 1.39 (s, 9H, CO$_2$C(C***H***$_3$)$_3$), 1.37 (s, 9H, (C***H***$_3$)$_3$COCONHCHCH$_2$CH$_2$SCH$_3$), 1.30 (d, *J* = 7.2 Hz, 3H, CHC***H***$_3$).

^{13}C{^{1}H} NMR (75 MHz, Chloroform-*d*) δ 171.8, 171.1, 155.6, 81.9, 79.9, 53.3, 48.7, 32.1, 30.1, 28.3, 28.0, 18.3, 15.2.

IR (cm^{-1}): 3296, 2979, 2933, 1737, 1660, 1540, 1452, 1368, 1301, 1248, 1153.

HRMS-ESI (m/z) calculated for $C_{17}H_{32}N_2O_5SNa$ [M+Na]$^+$ 399.1930, found 399.1926.

Boc-L-Cys(*t*Bu)-L-Ala-O*t*Bu (10i)

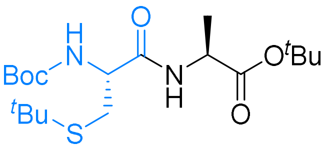

Following the general procedure (Method F) using Boc-L-Cys(*t*Bu)-OH and L-Ala-O*t*Bu, Boc-L-Cys(*t*Bu)-L-Ala-O*t*Bu (**10i**) was obtained as a colorless liquid (203 mg, 84% yield) with 99:1 dr. The characterization data matched previously reported data.[17,18]

[α]$_D^{22}$ = –2.5 (*c* 1.00, CHCl$_3$)

^{1}H NMR (300 MHz, Chloroform-*d*) δ 6.90 (d, *J* = 7.2 Hz, 1H, N***H***), 5.36 (s, 1H, BocN***H***CHCH$_2$SC(CH$_3$)$_3$), 4.42 (p, *J* = 7.1 Hz, 1H, C***H***CH$_3$), 4.32 – 4.17 (m, 1H, BocNHC***H***CH$_2$SC(CH$_3$)$_3$), 3.01 (dd, *J* = 13.0, 5.7 Hz, 1H, BocNHCHC***H***$_2$SC(CH$_3$)$_3$), 2.81 (dd, *J* = 13.0, 6.6 Hz, 1H, BocNHCHC***H***$_2$SC(CH$_3$)$_3$), 1.46 (s, 9H, CO$_2$C(C***H***$_3$)$_3$), 1.45 (s, 9H, (C***H***$_3$)$_3$OCONHCHCH$_2$SC(CH$_3$)$_3$), 1.37 (d, *J* = 7.1 Hz, 3H, CHC***H***$_3$), 1.33 (s, 9H, BocCHCH$_2$SC(C***H***$_3$)$_3$).

^{13}C{^{1}H} NMR (75 MHz, Chloroform-*d*) δ 171.8, 170.0, 155.4, 82.2, 80.4, 54.4, 49.1, 43.0, 31.0, 30.9, 28.4, 28.1, 18.7.

IR (cm^{-1}): 3310, 2979, 2937, 1733, 1660, 1515, 1455, 1367, 1248, 1160, 1051.

HRMS-ESI (m/z) calculated for $C_{19}H_{36}N_2O_5SNa$ [M+Na]$^+$ 427.2243, found 427.2234.

Boc-L-Pro-L-Ala-O^{t}Bu (10j)

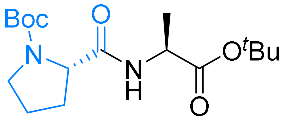

Following the general procedure (Method F) using Boc-L-Pro-OH and L-Ala-O^{t}Bu, Boc-L-Pro-L-Ala-O^{t}Bu (**10j**) was obtained as a white solid (151 mg, 73% yield) with 99:1 dr (ratio of rotamers = 1:1). The characterization data matched previously reported data.[17,18]

M.p. 71–73 ˚C

[α]$_D^{24}$ = –83.2 (*c* 1.00, CHCl$_3$)

^{1}H NMR (300 MHz, Chloroform-*d*) δ 7.18 (s, 1H x ½, N***H***), 6.66 (s, 1H x ½, N***H***), 4.61 – 4.03 (m, 2H, BocNCH$_2$CH$_2$CH$_2$C***H*** and C***H***CH$_3$), 3.52 – 3.31 (m, 2H, BocNC***H***$_2$CH$_2$CH$_2$CH), 2.31 – 1.81 (m, 4H, BocNCH$_2$C***H***$_2$C***H***$_2$CH), 1.43 (s, 18H, (C***H***$_3$)$_3$COCONCH$_2$CH$_2$CH$_2$CH and CO$_2$C(C***H***$_3$)$_3$), 1.33 (d, *J* = 7.1 Hz, 3H, CHC***H***$_3$).

^{13}C{^{1}H} NMR (75 MHz, Chloroform-*d*) δ 172.0 (1C x ½), 172.0, 171.9 (1C x ½), 155.6 (1C x ½), 154.7 (1C x ½), 82.0 (1C x ½), 81.9 (1C x ½), 80.7 (1C x ½), 80.4 (1C x ½), 61.2 (1C x ½), 60.1 (1C x ½), 48.7 (1C x ½), 48.6 (1C x ½), 47.1, 31.1 (1C x ½), 31.0 (1C x ½), 28.4 (3C), 28.0 (3C), 24.4 (1C x ½), 23.8 (1C x ½), 18.9 (1C x ½), 18.6 (1C x ½).

IR (cm^{-1}): 3310, 2976, 2933, 1737, 1699, 1674, 1543, 1393, 1368, 1248, 1160.

HRMS-ESI (m/z) calculated for $C_{17}H_{30}N_2O_5SNa$ [M+Na]$^+$ 365.2052, found 365.2050.

Boc-L-Tyr(^{t}Bu)-L-Ala-O^{t}Bu (10k)

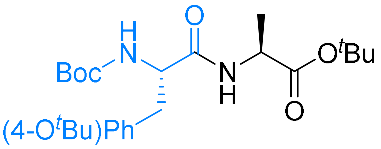

Following the general procedure (Method F) using Boc-L-Tyr(^{t}Bu)-OH and L-Ala-O^{t}Bu, Boc-L-Tyr(^{t}Bu)-L-Ala-O^{t}Bu (**10k**) was obtained as a white solid (247 mg, 88% yield) with 99:1 dr. The characterization data matched previously reported data.[17]

M.p. 54–56 ˚C

[α]$_D^{22}$ = +7.9 (*c* 1.00, $CHCl_3$)

^{1}H NMR (300 MHz, Chloroform-*d*) δ 7.08 (d, *J* = 8.4 Hz, 2H, $BocNHCHCH_2C_6\underline{\boldsymbol{H}}_4OC(CH_3)_3$), 6.93 – 6.84 (m, 2H, $BocNHCHCH_2C_6\underline{\boldsymbol{H}}_4OC(CH_3)_3$), 6.46 (d, *J* = 7.1 Hz, 1H, N$\underline{\boldsymbol{H}}$), 4.96 (s, 1H, $BocN\underline{\boldsymbol{H}}CHCH_2C_6H_4OC(CH_3)_3$), 4.46 – 4.15 (m, 2H, $BocNHC\underline{\boldsymbol{H}}CH_2C_6H_4OC(CH_3)_3$ and $C\underline{\boldsymbol{H}}CH_3$)), 3.01 (d, *J* = 6.6 Hz, 2H, $BocNHCHC\underline{\boldsymbol{H}}_2C_6H_4OC(CH_3)_3$), 1.43 (s, 9H, $CO_2C(C\underline{\boldsymbol{H}}_3)_3$), 1.40 (s, 9H, $(C\underline{\boldsymbol{H}}_3)_3COCONHCHCH_2C_6H_4OC(CH_3)_3$), 1.31 (s, 9H, $BocNHCHCH_2C_6H_4OC(C\underline{\boldsymbol{H}}_3)_3$), 1.29 (s, 3H, $CHC\underline{\boldsymbol{H}}_3$).

^{13}C{^{1}H} NMR (75 MHz, Chloroform-*d*) δ 171.8, 170.7, 155.4, 154.5, 131.4, 129.9, 124.4, 82.1, 80.3, 78.5, 55.7, 48.9, 37.8, 29.0, 28.4, 28.0, 18.7.

IR (cm^{-1}): 3296, 2979, 2933, 1737, 1685, 1656, 1529, 1364, 1237, 1160, 1051.

HRMS-ESI (m/z) calculated for $C_{25}H_{40}N_2O_6Na$ $[M+Na]^+$ 487.2784, found 487.2775.

Boc-L-Ser(Bn)-L-Ala-O^{t}Bu (10l)

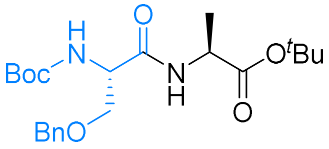

Following the general procedure (Method F) using Boc-L-Ser(Bn)-OH and L-Ala-O^{t}Bu, Boc-L-Ser(Bn)-L-Ala-O^{t}Bu (**10l**) was obtained as a colorless liquid (227 mg, 90% yield) with 99:1 dr.

[α]$_D^{19}$ = +25.5 (*c* 1.00, $CHCl_3$)

^{1}H NMR (300 MHz, Chloroform-*d*) δ 7.29 (d, *J* = 2.9 Hz, 5H, $BocNHCHCH_2OCH_2C_6\underline{\boldsymbol{H}}_5$), 7.12 (d, *J* = 6.5 Hz, 1H, N$\underline{\boldsymbol{H}}$), 5.47 (d, *J* = 7.0 Hz, $BocN\underline{\boldsymbol{H}}CHCH_2OCH_2C_6H_5$), 4.52 (s, 2H, $BocNHCHCH_2OC\underline{\boldsymbol{H}}_2C_6H_5$), 4.41 (p, *J* = 7.1 Hz, 1H, $C\underline{\boldsymbol{H}}CH_3$), 4.28 (s, 1H, $BocNHC\underline{\boldsymbol{H}}CH_2OCH_2C_6H_5$), 3.86 (dd, *J* = 9.4, 4.1 Hz, 1H, $BocNHCHC\underline{\boldsymbol{H}}_2OCH_2C_6H_5$), 3.57 (dd, *J* = 9.3, 6.3 Hz, 1H, $BocNHCHC\underline{\boldsymbol{H}}_2OCH_2C_6H_5$), 1.43 (s, 9H, $CO_2C(C\underline{\boldsymbol{H}}_3)_3$), 1.42 (s, 9H, $(C\underline{\boldsymbol{H}}_3)_3COCONHCHCH_2OCH_2C_6H_5$), 1.32 (d, *J* = 7.1 Hz, 3H, $CHC\underline{\boldsymbol{H}}_3$).

^{13}C{^{1}H} NMR (75 MHz, Chloroform-*d*) δ 171.8, 169.9, 155.6, 137.7, 128.5, 128.1 – 127.9 (m), 127.9, 81.9, 80.2, 73.5, 70.1, 54.8 – 53.4 (m), 49.0, 28.4, 28.0, 18.6.

IR (cm^{-1}): 3321, 2979, 1719, 1681, 1663, 1512, 1367, 1248, 1160, 1051.

HRMS-ESI (m/z) calculated for $C_{22}H_{34}N_2O_6Na$ $[M+Na]^+$ 445.2315, found 445.2309.

Boc-L-Thr(Bn)-L-Ala-O^{t}Bu (10m)

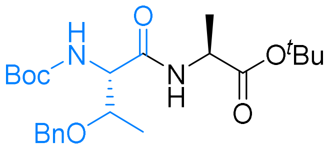

Following the general procedure (Method F) using Boc-L-Thr(Bn)-OH and L-Ala-O^{t}Bu, Boc-L-Thr(Bn)-L-Ala-O^{t}Bu (**10m**) was obtained as a colorless liquid (216 mg, 83% yield) with 99:1 dr.

[α]$_D^{25}$ = +16.0 (*c* 1.00, CHCl$_3$)

^{1}H NMR (300 MHz, Chloroform-*d*) δ 7.36 – 7.26 (m, 5H, BocNHCHCH(CH$_3$)OCH$_2$C$_6$***H***$_5$), 7.14 (d, *J* = 7.2 Hz, 1H, N***H***), 5.47 (d, *J* = 7.3 Hz, 1H, BocN***H***CHCH(CH$_3$)OCH$_2$C$_6$H$_5$), 4.67 – 4.50 (m, 2H, BocNHCHCH(CH$_3$)OC***H***$_2$C$_6$H$_5$), 4.42 (p, *J* = 7.0 Hz, 1H, C***H***CH$_3$), 4.31 – 4.23 (m, 1H, BocNHC***H***CH(CH$_3$)OCH$_2$C$_6$H$_5$), 4.23 – 4.14 (m, 1H, BocNHCHC***H***(CH$_3$)OCH$_2$C$_6$H$_5$), 1.45 (s, 9H, CO$_2$C(C***H***$_3$)$_3$), 1.44 (s, 9H, (C***H***$_3$)$_3$COCONHCHCH(CH$_3$)OCH$_2$C$_6$H$_5$), 1.33 (d, *J* = 7.1 Hz, 3H, CHC***H***$_3$), 1.21 (d, *J* = 6.4 Hz, 3H, BocNHCHCH(C***H***$_3$)OCH$_2$C$_6$H$_5$).

^{13}C{^{1}H} NMR (75 MHz, Chloroform-*d*) δ 171.8, 169.6, 156.0, 138.2, 128.5, 128.3, 127.9, 82.0, 80.2, 75.0, 71.7, 57.8, 49.0, 28.4, 28.1, 18.6, 15.6.

IR (cm^{-1}): 3327, 3053, 2979, 1719, 1681, 1663, 1498, 1367, 1244, 1146, 1058.

HRMS-ESI (m/z) calculated for $C_{23}H_{36}N_2O_6Na$ [M+Na]$^+$ 459.2471, found 459.2463.

Boc-L-Asn(Trt)-L-Ala-O^{t}Bu (10n)

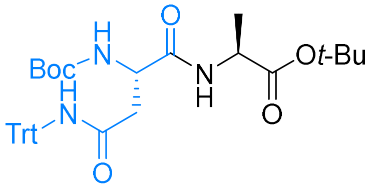

Following the general procedure (Method F) using Boc-L-Asn(Trt)-OH and L-Ala-O^{t}Bu, Boc-L-Asn(Trt)-L-Ala-O^{t}Bu (**10n**) was obtained as a white solid (252 mg, 70% yield) with 99:1 dr. The characterization data matched previously reported data.[17]

M.p. 169–171 ˚C

[α]$_D^{25}$ = –3.6 (*c* 1.00, CHCl$_3$)

^{1}H NMR (300 MHz, Chloroform-*d*) δ 7.32 – 7.14 (m, 15H, BocNHCHCH$_2$CONHC(C$_6$***H***$_5$)$_3$), 6.96 (s, 1H, BocN***H***CHCH$_2$CONHTrt), 6.20 (d, *J* = 8.3 Hz, 1H, N***H***), 4.51 – 4.39 (m, 1H, BocNHC***H***CH$_2$CONHTrt), 4.33 (p, *J* = 7.1 Hz, 1H, C***H***CH$_3$), 3.09 (d, *J* = 14.6 Hz, 1H, BocNHCHC***H***$_2$CONHTrt), 2.58 (dd, *J* = 15.2, 5.0 Hz, 1H, BocNHCHC***H***$_2$CONHTrt), 1.45 (s, 9H, CO$_2$C(C***H***$_3$)$_3$), 1.43 (s, 9H, (C***H***$_3$)$_3$NHCHCH$_2$CONHTrt), 1.28 (d, *J* = 7.1 Hz, 3H, CHC***H***$_3$).

^{13}C{^{1}H} NMR (75 MHz, Chloroform-*d*) δ 171.6, 170.9, 170.7, 155.9, 144.5, 128.8, 128.1, 127.2, 81.9, 80.3, 70.9, 51.3, 49.2, 38.1, 28.4, 28.1, 18.3.

IR (cm^{-1}): 3313, 2979, 1716, 1663, 1494, 1448, 1367, 1248, 1156, 1051.

HRMS-ESI (m/z) calculated for $C_{35}H_{44}N_3O_6Na$ [M+H]$^+$ 602.3230, found 602.3228.

Boc-L-Gln(Trt)-L-Ala-O*t*Bu (10o)

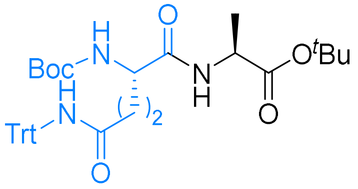

Following the general procedure (Method F) using Boc-L-Gln(Trt)-OH and L-Ala-O*t*Bu, Boc-L-Gln(Trt)-L-Ala-O*t*Bu (**10o**) was obtained as a white solid (328 mg, 89% yield) with 99:1 dr. The characterization data matched previously reported data.[17]

M.p. 88–90 ˚C

[α]$_D$^{24} = +7.2 (*c* 1.00, $CHCl_3$)

^{1}H NMR (300 MHz, Chloroform-*d*) δ 7.30 – 7.23 (m, 15H, BocNHCHCH$_2$CH$_2$CO$_2$C(C$_6$***H***$_5$)$_3$), 6.64 (d, *J* = 7.3 Hz, 1H, N***H***), 5.46 (d, *J* = 7.3 Hz, 1H, BocN***H***CHCH$_2$CH$_2$CO$_2$Trt), 4.34 (p, *J* = 7.2 Hz, 1H, C***H***CH$_3$), 4.14 – 3.97 (m, 1H, BocNHC***H***CH$_2$CH$_2$CO$_2$Trt), 2.47 (t, *J* = 6.6 Hz, 2H, BocNHCHCH$_2$C***H***$_2$CO$_2$Trt), 2.09 – 1.84 (m, 2H, BocNHCHC***H***$_2$CH$_2$CO$_2$Trt), 1.43 (s, 18H, (C***H***$_3$)$_3$COCONHCHCH$_2$CH$_2$CO$_2$Trt and CO$_2$C(C***H***$_3$)$_3$), 1.28 (d, *J* = 7.2 Hz, 3H, CHC***H***$_3$).

^{13}C{^{1}H} NMR (75 MHz, Chloroform-*d*) δ 172.0, 171.9, 171.3, 155.9, 144.8, 128.8, 128.1, 127.1, 82.0, 80.0, 70.7, 53.5, 48.9, 33.6, 29.8, 28.5, 28.1, 18.1.

IR (cm^{-1}): 3310, 3060, 2976, 2933, 1663, 1494, 1445, 1368, 1248, 1156, 1051.

HRMS-ESI (m/z) calculated for $C_{36}H_{45}N_3O_6Na$ [M+Na]$^+$ 638.3206, found 638.3205.

Boc-L-Lys(Boc)-L-Ala-O*t*Bu (10p)

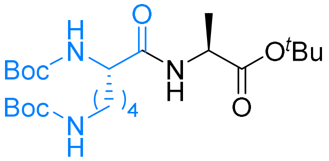

Following the general procedure (Method F) using Boc-L-Lys(Boc)-OH and L-Ala-O*t*Bu, Boc-L-Lys(Boc)-L-Ala-O*t*Bu (**10p**) was obtained as a white solid (231 mg, 81% yield) with 99:1 dr. The characterization data matched previously reported data.[17]

M.p. 54–56 ˚C

[α]$_D^{24}$ = –8.6 (*c* 1.00, $CHCl_3$)

^{1}H NMR (300 MHz, Chloroform-*d*) δ 6.66 (d, *J* = 7.3 Hz, 1H, N**_H_**), 5.19 (d, *J* = 6.1 Hz, 1H, BocN**_H_**CHCH$_2$CH$_2$CH$_2$CH$_2$NHBoc), 4.69 (s, 1H, BocNHCHCH$_2$CH$_2$CH$_2$CH$_2$N**_H_**Boc), 4.40 (p, *J* = 7.2 Hz, 1H,C**_H_**CH$_3$), 4.15 – 3.97 (m, 1H, BocNHC**_H_**CH$_2$CH$_2$CH$_2$CH$_2$NHBoc), 3.22 – 2.96 (m, 2H, BocNHCHCH$_2$CH$_2$CH$_2$C**_H_**$_2$NHBoc), 1.89 – 1.74 (m, 1H, BocNHCHC**_H_**$_2$CH$_2$CH$_2$CH$_2$NHBoc), 1.68 – 1.56 (m, 1H, BocNHCHC**_H_**$_2$CH$_2$CH$_2$CH$_2$NHBoc), 1.56 – 1.44 (m, 4H, BocNHCHCH$_2$C**_H_**$_2$C**_H_**$_2$CH$_2$NHBoc), 1.44 (s, 9H, CO$_2$C(C**_H_**$_3$)$_3$), 1.42 (s, 18H, (C**_H_**$_3$)$_3$COCONHCHCH$_2$CH$_2$CH$_2$CH$_2$NHCO$_2$C(C**_H_**$_3$)$_3$), 1.34 (d, *J* = 7.1 Hz, 3H, CHC**_H_**$_3$).

^{13}C{^{1}H} NMR (75 MHz, Chloroform-*d*) δ 172.0, 171.6, 156.3, 155.8, 82.1, 80.1, 79.2, 54.4, 48.8, 40.1, 32.3, 29.7, 28.6, 28.4, 28.1, 22.6, 18.5.

IR (cm^{-1}): 3324, 2976, 2933, 1779, 1691, 1660, 1515, 1452, 1364, 1248, 1153.

HRMS-ESI (m/z) calculated for $C_{23}H_{44}N_3O_7$ [M+H]$^+$ 474.3179, found 474.3179.

Boc-L-Arg(Cbz)$_2$-L-Ala-O^{t}Bu (10q)

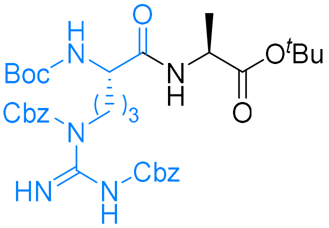

Following the general procedure (Method F) using Boc-L-Arg(Cbz)$_2$-OH and L-Ala-O^{t}Bu, Boc-L- Arg(Cbz)$_2$-L-Ala-O^{t}Bu (**10q**) was obtained as a white solid (348 mg, 87% yield) with 99:1 dr. The characterization data matched previously reported data.[18]

M.p. 43–45 ˚C

[α]$_D^{22}$ = +3.9 (*c* 1.00, $CHCl_3$)

^{1}H NMR (300 MHz, Chloroform-*d*) δ 9.44 (s, 1H, BocNHCH(CH$_2$)$_3$N(Cbz)CNHN**_H_**Cbz), 9.30 (s, 1H, BocNHCH(CH$_2$)$_3$N(Cbz)CN**_H_**NHCbz), 7.56 – 7.26 (m, 10H, BocNHCH(CH$_2$)$_3$N(CO$_2$CH$_2$C$_6$**_H_**$_5$)CNHNH(CO$_2$CH$_2$C$_6$**_H_**$_5$)), 6.79 (d, *J* = 7.4 Hz, 1H, N**_H_**), 5.51 (d, *J* = 7.6 Hz, 1H, BocN**_H_**CH(CH$_2$)$_3$N(Cbz)CNHNH(Cbz)), 5.25 (s, 2H, BocNHCH(CH$_2$)$_3$N(CO$_2$CH$_2$C$_6$H$_5$)CNHNH(CO$_2$C**_H_**$_2$C$_6$H$_5$)), 5.22 – 5.10 (m, 2H, BocNHCH(CH$_2$)$_3$N(CO$_2$C**_H_**$_2$C$_6$H$_5$)CNHNH(CO$_2$CH$_2$C$_6$H$_5$)), 4.35 (p, *J* = 7.2 Hz, 1H, CHC**_H_**$_3$), 4.30 – 4.15 (m, 1H, BocNHC**_H_**(CH$_2$)$_3$N(Cbz)CNHNH(Cbz)), 4.13 – 4.01 (m, 1H, BocNHCH(CH$_2$)$_2$C**_H_**$_2$N(Cbz)CNHNH(Cbz)), 4.01 – 3.87 (m, 1H, BocNHCH(CH$_2$)$_2$C**_H_**$_2$N(Cbz)CNHNH(Cbz)), 1.86 – 1.63 (m, 4H, BocNHCH(C**_H_**$_2$)$_2$CH$_2$N(Cbz)CNHNH(Cbz)), 1.43 (s, 18H, (CH$_3$)$_3$OCONHCH(CH$_2$)$_3$N(Cbz)CNHNH(Cbz) and CO$_2$C(CH$_3$)$_3$), 1.20 (d, *J* = 7.2 Hz, 3H, CHC**_H_**$_3$).

^{13}C{^{1}H} NMR (75 MHz, Chloroform-*d*) δ 171.8, 171.5, 163.8, 160.9, 156.0, 155.7, 136.9, 134.8, 129.0, 129.0, 128.6, 128.5, 128.0, 127.9, 81.8, 79.9, 69.1, 67.1, 54.0, 48.8, 44.2, 29.0, 28.5, 28.1, 24.9, 18.2.

IR (cm^{-1}): 3387, 2979, 2941, 1718, 1660, 1609, 1510, 1369, 1248, 11461, 1098.

HRMS-ESI (m/z) calculated for $C_{34}H_{48}N_5O_9$ $[M+H]^+$ 670.3452, found 670.3450.

Boc-L-His(Bn)-L-Ala-O*t*Bu (10r)

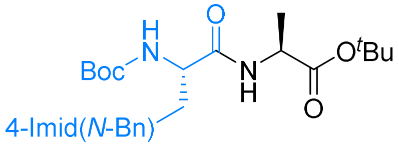

Following the general procedure (Method F) using Boc-L-His(Bn)-OH and L-Ala-O*t*Bu, Boc-L-His(Bn)-L-Ala-O*t*Bu (**10r**) was obtained as a white solid (219 mg, 77% yield) with 99:1 dr. [17]

M.p. 98–100 ˚C

$[\alpha]_D^{24}$ = +25.5 (*c* 1.00, $CHCl_3$)

^{1}H NMR (300 MHz, Chloroform-*d*) δ 7.48 (s, 1H, N***H***), 7.34 – 7.28 (m, 3H, BocNHCHCH$_2$Imid***H***(CH$_2$C$_6$***H***$_5$)), 7.18 – 7.09 (m, 2H, BocNHCHCH$_2$Imid(CH$_2$C$_6$***H***$_5$)), 6.71 (s, 1H, BocNHCHCH$_2$Imid***H***(CH$_2$C$_6$H$_5$)), 6.33 (s, 1H, BocN***H***CHCH$_2$Imid(CH$_2$C$_6$H$_5$)), 5.01 (s, 2H, BocNHCHCH$_2$Imid(C***H***$_2$C$_6$H$_5$)), 4.42 – 4.33 (m, 1H, BocNHC***H***CH$_2$Imid(CH$_2$C$_6$H$_5$)), 4.30 (q, *J* = 7.1 Hz, 1H, CHC***H***$_3$), 3.19 – 3.02 (m, 1H, BocNHCHC***H***$_2$Imid(CH$_2$C$_6$H$_5$)), 2.92 (dd, *J* = 14.7, 5.6 Hz, 1H, BocNHCHC***H***$_2$Imid(CH$_2$C$_6$H$_5$)), 1.42 (s, 9H, CO$_2$C(C***H***$_3$)$_3$), 1.41 (s, 9H, (C***H***$_3$)$_3$COCONHCHCH$_2$Imid(CH$_2$C$_6$H$_5$)), 1.13 (d, *J* = 7.1 Hz, 3H, CHC***H***$_3$).

^{13}C{^{1}H} NMR (75 MHz, Chloroform-*d*) δ 171.9, 171.1, 155.8, 138.4, 136.7, 135.9, 129.1, 128.5, 127.6, 117.4, 81.8, 79.9, 54.7, 51.1, 48.8, 30.4, 28.4, 28.0, 18.5.

IR (cm^{-1}): 3303, 2979, 2930, 1713, 1670, 1498, 1452, 1364, 1248, 1157.

HRMS-ESI (m/z) calculated for $C_{25}H_{36}N_4O_5Na$ $[M+Na]^+$ 495.2583, found 495.2586.

Boc-L-Asp(*t*Bu)-L-Ala-O*t*Bu (10s)

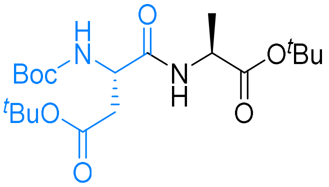

Following the general procedure (Method F) using Boc-L-Asp(*t*Bu)-OH and L-Ala-O*t*Bu, Boc-L-Asp(*t*Bu)-L-Ala-O*t*Bu (**10s**) was obtained as a colorless liquid (207 mg, 83% yield) with 99:1 dr. The characterization data matched previously reported data.[17]

$[\alpha]_D^{24}$ = +22.4 (*c* 1.10, $CHCl_3$)

^{1}H NMR (300 MHz, Chloroform-*d*) δ 7.03 (d, *J* = 7.3 Hz, 1H, N***H***), 5.68 (d, *J* = 7.6 Hz, 1H, BocN***H***CHCH$_2$CO$_2$C(CH$_3$)$_3$), 4.51 – 4.41 (m, 1H, BocNHC***H***CH$_2$CO$_2$C(CH$_3$)$_3$), 4.38 (p, *J* = 7.1 Hz, 1H, C***H***CH$_3$), 2.87 (dd, *J* = 16.9, 4.7 Hz, 1H, BocNHCHC***H***$_2$CO$_2$C(CH$_3$)$_3$), 2.58 (dd, *J* = 16.9, 6.3 Hz, 1H,

BocNHCHC***H***$_2$CO$_2$C(CH$_3$)$_3$), 1.44 (s, 9H, CO$_2$C(C***H***$_3$)$_3$), 1.44 (s, 9H, (C***H***$_3$)$_3$COCONHCHCH$_2$CO$_2$C(CH$_3$)$_3$), 1.43 (s, 9H, BocNHCHCH$_2$CO$_2$C(C***H***$_3$)$_3$), 1.34 (d, J = 7.1 Hz, 3H, CHC***H***$_3$).

^{13}C{^{1}H} NMR (75 MHz, Chloroform-*d*) δ 171.7, 171.3, 170.4, 155.5, 82.0, 81.8, 80.4, 50.8, 49.0, 37.6, 28.4, 28.1, 28.1, 18.5.

IR (cm^{-1}): 3314, 2979, 2934, 1730, 1667, 1519, 1368, 1301, 1248, 1149, 1048.

HRMS-ESI (m/z) calculated for $C_{20}H_{36}N_2O_7Na$ [M+Na]$^+$ 439.2420, found 474.2420.

Boc-L-Glu(^{t}Bu)-L-Ala-O^{t}Bu (10t)

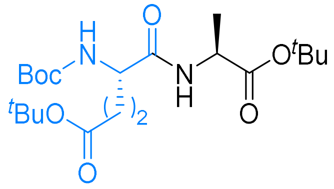

Following the general procedure (Method F) using Boc-L-Glu(^{t}Bu)-OH and L-Ala-O^{t}Bu, Boc-L-Glu(^{t}Bu)-L-Ala-O^{t}Bu (**10t**) was obtained as a colorless liquid (212 mg, 82% yield) with 99:1 dr. The characterization data matched previously reported data.[17]

M.p. 98–100 ˚C

[α]$_D$^{24} = –8.1 (*c* 1.00, CHCl$_3$)

^{1}H NMR (300 MHz, Chloroform-*d*) δ 6.73 (d, J = 7.4 Hz, 1H, N***H***), 5.27 (d, J = 7.2 Hz, 1H, BocN***H***CHCH$_2$CH$_2$CO$_2$C(CH$_3$)$_3$), 4.41 (p, J = 7.1 Hz, 1H, C***H***CH$_3$), 4.25 – 3.99 (m, 1H, BocNHC***H***CH$_2$CH$_2$CO$_2$C(CH$_3$)$_3$), 2.46 – 2.27 (m, 2H, BocNHCHCH$_2$C***H***$_2$CO$_2$C(CH$_3$)$_3$), 2.15 – 1.98 (m, 1H, BocNHCHC***H***$_2$CH$_2$CO$_2$C(CH$_3$)$_3$), 1.95 – 1.81 (m, 1H, BocNHCHC***H***$_2$CH$_2$CO$_2$C(CH$_3$)$_3$), 1.45 (s, 9H, CO$_2$C(C***H***$_3$)$_3$), 1.44 (s, 9H, (C***H***$_3$)$_3$COCONHCH), 1.43 (s, 9H, BocNHCHCH$_2$CH$_2$CO$_2$C(C***H***$_3$)$_3$), 1.36 (d, J = 7.1 Hz, 3H, CHC***H***$_3$).

^{13}C{^{1}H} NMR (75 MHz, Chloroform-*d*) δ 172.9, 171.9, 171.1, 155.7, 82.1, 81.0, 80.1, 54.0, 48.9, 31.9, 28.4, 28.2, 28.1, 18.6.

IR (cm^{-1}): 3313, 2979, 2933, 1726, 1660, 1526, 1452, 1364, 1245, 1150, 1048.

HRMS-ESI (m/z) calculated for $C_{21}H_{39}N_2O_7$ [M+H]$^+$ 431.2757, found 431.2754.

Boc-L-Orn(Cbz)-L-Ala-O^{t}Bu (10u)

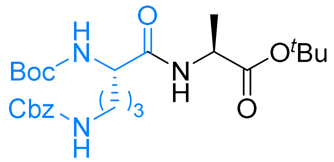

Following the general procedure (Method F) using Boc-L-Orn(Cbz)-OH and L-Ala-O^{t}Bu, Boc-L-Orn(Cbz)-L-Ala-O^{t}Bu (**10u**) was obtained as a white solid (242 mg, 82% yield) with 99:1 dr.

M.p. 70–72 ˚C

$[\alpha]_D^{24}$ = +1.9 (*c* 1.25, $CHCl_3$)

^{1}H NMR (300 MHz, Chloroform-*d*) δ 7.39 – 7.25 (m, 5H, $BocNHCHCH_2CH_2CH_2NHCO_2CH_2C_6\underline{\boldsymbol{H}}_5$), 6.79 (d, *J* = 7.6 Hz, 1H, $BocN\underline{\boldsymbol{H}}CHCH_2CH_2CH_2NHCO_2CH_2C_6H_5$), 5.25 – 5.13 (m, 1H, N***H***), 5.10 (d, *J* = 3.9 Hz, 2H, $BocNHCHCH_2CH_2CH_2NHCO_2C\underline{\boldsymbol{H}}_2C_6H_5$), 5.06 – 4.93 (m, 1H, $BocNHCHCH_2CH_2CH_2N\underline{\boldsymbol{H}}CO_2CH_2C_6H_5$), 4.41 (p, *J* = 7.2 Hz, 1H, $C\underline{\boldsymbol{H}}CH_3$), 4.35 – 4.06 (m, 1H, $BocNHC\underline{\boldsymbol{H}}CH_2CH_2CH_2NHCO_2CH_2C_6H_5$), 3.55 – 3.28 (m, 1H, $BocNHCHCH_2CH_2C\underline{\boldsymbol{H}}_2NHCO_2CH_2C_6H_5$), 3.22 – 3.10 (m, 1H, $BocNHCHCH_2CH_2C\underline{\boldsymbol{H}}_2NHCO_2CH_2C_6H_5$), 1.90 – 1.79 (m, 1H, $BocNHCHC\underline{\boldsymbol{H}}_2CH_2CH_2NHCO_2CH_2C_6H_5$), 1.67 – 1.52 (m, 3H, $BocNHCHC\underline{\boldsymbol{H}}_2C\underline{\boldsymbol{H}}_2CH_2NHCO_2CH_2C_6H_5$), 1.44 (s, 9H, $CO_2C(C\underline{\boldsymbol{H}}_3)_3$), 1.43 (s, 9H, $(C\underline{\boldsymbol{H}}_3)_3COCONHCHCH_2CH_2CH_2NHCO_2CH_2C_6H_5$), 1.36 (d, *J* = 7.2 Hz, 3H, $CHC\underline{\boldsymbol{H}}_3$).

^{13}C{^{1}H} NMR (75 MHz, Chloroform-*d*) δ 172.1, 171.7, 157.0, 155.8, 136.7, 128.6, 128.2, 82.0, 80.0, 66.9, 53.3, 48.8, 40.0, 30.4, 28.4, 28.1, 26.2, 18.4.

IR (cm^{-1}): 3321, 2976, 2934, 1702, 1660, 1523, 1455, 1364, 1248, 1156.

HRMS-ESI (m/z) calculated for C25H40N3O7 $[M+H]^+$ 494.2866, found 494.2866.

Boc-L-Phg-L-Ala-O^{t}Bu (10v)

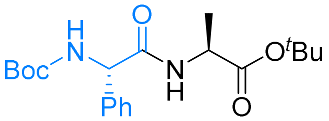

Following the general procedure (Method F) using Boc-L-Phg-OH and L-Ala-O^{t}Bu, Boc-L-Phg-L-Ala-O^{t}Bu (**10v**) was obtained as a white solid (179 mg, 79% yield) with 98:2 dr. The characterization data matched previously reported data.[17,18]

M.p. 110–112 ˚C

$[\alpha]_D^{24}$ = +73.11 (*c* 1.00, $CHCl_3$)

^{1}H NMR (300 MHz, Chloroform-*d*) δ 7.42 – 7.26 (m, 5H, $BocNHCHC_6\underline{\boldsymbol{H}}_5$), 6.36 (d, *J* = 7.1 Hz, 1H, $BocN\underline{\boldsymbol{H}}CHC_6H_5$), 5.75 (s, 1H, N***H***), 5.13 (s, 1H, $BocNHC\underline{\boldsymbol{H}}C_6H_5$), 4.38 (p, *J* = 7.1 Hz, 1H, $C\underline{\boldsymbol{H}}CH_3$), 1.40 (s, 9H, $CO_2C(C\underline{\boldsymbol{H}}_3)_3$), 1.37 (s, 9H, $(C\underline{\boldsymbol{H}}_3)_3COCONHCHC_6H_5$), 1.36 (d, J = 7.5 Hz, 3H, $CHC\underline{\boldsymbol{H}}_3$).

^{13}C{^{1}H} NMR (75 MHz, Chloroform-*d*) δ 171.6, 169.5, 155.2, 138.3, 129.1, 128.5, 127.4, 82.2, 80.2, 58.8, 49.2, 28.4, 28.0, 18.7.

IR (cm^{-1}): 3310, 2979, 2934, 1723, 1660, 1519, 1498, 1368, 1248, 1157.

HRMS-ESI (m/z) calculated for $C_{20}H_{30}N_2O_5Na$ $[M+Na]^+$ 401.2052, found 401.2051.

Boc-D-Ala-L-Ala-OBn (11a)

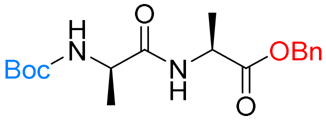

Following the general procedure (Method F) using Boc-D-Ala-OH and L-Ala-OBn, Boc-D-Ala-L-Ala-OBn (**11a**) was obtained as a white solid (190 mg, 90% yield) with 99:1 dr. The characterization data matched previously reported data.[20]

M.p. 72–74 ˚C

[α]$_D$^{25} = +24.5 (*c* 1.00, $CHCl_3$)

^{1}H NMR (300 MHz, Chloroform-*d*) δ 7.42 – 7.27 (m, 5H, $CO_2CH_2C_6$***H***$_5$), 6.71 (s, 1H, N***H***), 5.19 (d, *J* = 12.3 Hz, 1H, CO_2C***H***$_2$Ph), 5.13 (d, *J* = 12.3 Hz, 1H, CO_2C***H***$_2$Ph), 4.91 (s, 1H, BocN***H***$CHCH_3$), 4.60 (p, *J* = 7.2 Hz, 1H, C***H***CH_3), 4.33 – 4.03 (m, 1H, BocNHC***H***CH_3), 1.44 (s, 9H, (C***H***$_3$)$_3$COCONHCHCH$_3$), 1.41 (d, *J* = 7.2 Hz, 3H, BocNHCHC***H***$_3$), 1.34 (d, *J* = 7.1 Hz, 3H, CHC***H***$_3$).

^{13}C{^{1}H} NMR (75 MHz, Chloroform-*d*) δ 172.8, 172.3, 155.6, 135.5, 128.8, 128.6, 128.3, 80.4, 67.3, 50.1, 48.2, 28.4, 18.4, 18.3.

IR (cm^{-1}): 3320, 2980, 2933, 1740, 1663, 1512, 1248, 1163, 1054.

HRMS-ESI (m/z) calculated for $C_{18}H_{26}N_2O_5Na$ $[M+Na]^+$ 373.1739, found 351.1733.

Cbz-D-Ala-L-Ala-O^{t}Bu (11b)

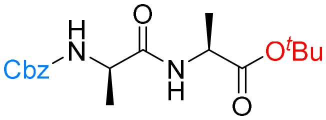

Following the general procedure (Method F) using Cbz-D-Ala-OH and L-Ala-O^{t}Bu, Cbz-D-Ala-L-Ala-O^{t}Bu (**11b**) was obtained as a colorless oil (180 mg, 85% yield) with 99:1 dr.

[α]$_D$^{25} = +14.0 (*c* 1.00, $CHCl_3$)

^{1}H NMR (300 MHz, Chloroform-*d*) δ 7.37 – 7.30 (m, 5H, C_6***H***$_5CH_2$OCONH), 6.62 (s, 1H, N***H***), 5.39 (s, 1H, CbzN***H***), 5.12 (s, 2H, PhC***H***$_2$OCONH), 4.42 (p, *J* = 7.1 Hz, 1H, CHC***H***$_3$), 4.33 – 4.16 (m, 1H, CbzNHC***H***CH_3), 1.45 (s, 9H, CO_2C(C***H***$_3$)$_3$), 1.40 – 1.32 (m, 6H, CHC***H***$_3$, CbzNHCHC***H***$_3$).

^{13}C{^{1}H} NMR (75 MHz, Chloroform-*d*) δ 172.1, 171.7, 156.0, 136.3, 128.7, 128.3, 128.2, 82.3, 67.2, 50.7, 48.8, 28.1, 18.8, 18.6.

IR (cm^{-1}): 3320, 3035, 2979, 2940, 1726, 1660, 1522, 1452, 1367, 1241, 1149, 1061.

HRMS-ESI (m/z) calculated for $C_{18}H_{26}N_2O_5Na$ $[M+Na]^+$ 373.1739, found 373.1733.

Fmoc-D-Ala-L-Ala-O^{t}Bu (11c)

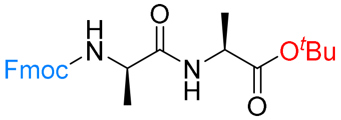

Following the general procedure (Method F) using Fmoc-D-Ala-OH and L-Ala-O^{t}Bu, Fmoc-D-Ala-L-Ala-O^{t}Bu (**11c**) was obtained as a white solid (242 mg, 92% yield) with 99:1 dr.

M.p. 108–109 ˚C

[α]$_D^{24}$ = +12.8 (*c* 1.00, $CHCl_3$)

^{1}H NMR (300 MHz, Chloroform-*d*) δ 7.76 (d, *J* = 7.4 Hz, 2H, $C_{12}\underline{\boldsymbol{H}}_8CHCH_2OCONHCHCH_3$), 7.59 (d, *J* = 7.4 Hz, 2H, $C_{12}\underline{\boldsymbol{H}}_8CHCH_2OCONHCHCH_3$), 7.40 (t, *J* = 7.3 Hz, 2H, $C_{12}\underline{\boldsymbol{H}}_8CHCH_2OCONHCHCH_3$), 7.31 (t, *J* = 7.4 Hz, 2H, $C_{12}\underline{\boldsymbol{H}}_8CHCH_2OCONHCHCH_3$), 6.62 (d, *J* = 7.1 Hz, 1H, N$\underline{\boldsymbol{H}}$), 5.45 (d, *J* = 7.4 Hz, 1H, FmocN$\underline{\boldsymbol{H}}$$CHCH_3$), 4.52 – 4.36 (m, 3H, , $C_{12}H_8CHC\underline{\boldsymbol{H}}_2OCONHCHCH_3$, C$\underline{\boldsymbol{H}}$$CH_3$), 4.29 (t, *J* = 7.0 Hz, 1H, FmocNHC$\underline{\boldsymbol{H}}$$CH_3$), 4.22 (t, *J* = 7.0 Hz, 1H, , $C_{12}H_8C\underline{\boldsymbol{H}}CH_2OCONHCHCH_3$), 1.45 (s, 9H, , $CO_2C(C\underline{\boldsymbol{H}}_3)_3$), 1.43 – 1.33 (m, 6H, FmocNHCHC$\underline{\boldsymbol{H}}_3$, CHC$\underline{\boldsymbol{H}}_3$).

^{13}C{^{1}H} NMR (75 MHz, Chloroform-*d*) δ 172.1, 171.7, 156.1, 144.0, 143.9, 141.4, 127.9, 127.2, 125.2, 120.1, 82.3, 67.3, 50.6, 48.8, 47.3, 28.1, 19.0, 18.6.

IR (cm^{-1}): 3306, 2979, 2937, 1726, 1663, 1522, 1448, 1367, 1244, 1153.

HRMS-ESI (m/z) calculated for $C_{25}H_{30}N_2O_5Na$ $[M+Na]^+$ 461.2052, found 461.2052.

Fmoc-D-Ala-L-Ala-OMe (11d)

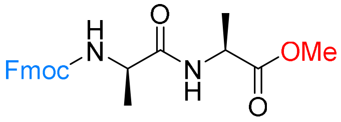

Following the general procedure (Method F) using Fmoc-D-Ala-OH and L-Ala-OMe, Fmoc-D-Ala-L-Ala-OMe (**11d**) was obtained as a white solid (230 mg, 97% yield) with 99:1 dr.

M.p. 184–186 ˚C

[α]$_D^{23}$ = +12.4 (*c* 1.00, $CHCl_3$)

^{1}H NMR (300 MHz, Chloroform-*d*) δ 7.76 (d, *J* = 7.4 Hz, 2H, $C_{12}\underline{\boldsymbol{H}}_8CHCH_2OCONHCHCH_3$), 7.59 (d, *J* = 7.4 Hz, 2H, $C_{12}\underline{\boldsymbol{H}}_8CHCH_2OCONHCHCH_3$), 7.40 (t, *J* = 7.3 Hz, 2H, $C_{12}\underline{\boldsymbol{H}}_8CHCH_2OCONHCHCH_3$), 7.31 (t, *J* = 7.4 Hz, 2H, $C_{12}\underline{\boldsymbol{H}}_8CHCH_2OCONHCHCH_3$), 6.63 (s, 1H, N$\underline{\boldsymbol{H}}$), 5.39 (d, *J* = 7.6 Hz, 1H, FmocN$\underline{\boldsymbol{H}}$$CHCH_3$), 4.57 (p, *J* = 7.2 Hz, 1H, C$\underline{\boldsymbol{H}}$$CH_3$), 4.41 (d, *J* = 6.7 Hz, 2H, $C_{12}H_8CHC\underline{\boldsymbol{H}}_2OCONHCHCH_3$), 4.35 – 4.26 (m, 1H, FmocNHC$\underline{\boldsymbol{H}}$$CH_3$), 4.22 (t, *J* = 7.0 Hz, 1H, $C_{12}H_8C\underline{\boldsymbol{H}}CH_2OCONHCHCH_3$), 3.73 (s, 3H, $CO_2C\underline{\boldsymbol{H}}_3$), 1.42 (s, 3H, FmocNHCHC$\underline{\boldsymbol{H}}_3$), 1.39 (s, 3H, CHC$\underline{\boldsymbol{H}}_3$).

^{13}C{^{1}H} NMR (75 MHz, Chloroform-*d*) δ 173.3, 171.9, 156.1, 143.9, 143.9, 141.4, 127.9, 127.2, 125.2, 120.1, 67.3, 52.7, 50.6, 48.2, 47.2, 18.8, 18.4.

IR (cm^{-1}): 3310, 3064, 2986, 2951, 1730, 1663, 1526, 1448, 1325, 1248, 1216, 1153.

HRMS-ESI (m/z) calculated for $C_{22}H_{24}N_2O_5Na$ $[M+Na]^+$ 419.1583, found 419.1580.

Boc-L-Ala-L-Ala-OBn (11e)

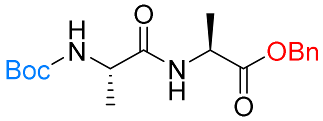

Following the general procedure (Method F, 8 mmol scale) using Boc-L-Ala-OH and L-Ala-OBn, Boc-L-Ala-L-Ala-OBn (11e) was obtained as a white solid (2.163 g, 77% yield) with 99:1 dr.

M.p. 64–66 ˚C

$[\alpha]_D^{24}$ = –33.6 (*c* 1.0, $CHCl_3$)

^{1}H NMR (300 MHz, Chloroform-*d*) δ 7.41 – 7.29 (m, 5H, $CO_2CH_2C_6\underline{H}_5$), 6.64 (d, *J* = 6.1 Hz, 1H, N$\underline{H}$), 5.19 (d, *J* = 12.3 Hz, 1H, $CO_2C\underline{H}_2Ph$), 5.16 (d, *J* = 12.3 Hz, 1H, $CO_2C\underline{H}_2Ph$), 5.01 (s, 1H, BocN$\underline{H}$CHCH$_3$), 4.60 (p, *J* = 7.2 Hz, 1H, C$\underline{H}$CH$_3$), 4.29 – 4.05 (m, 1H), 1.43 (s, 9H, $(C\underline{H}_3)_3COCONHCHCH_3$), 1.40 (d, *J* = 7.2 Hz, 3H, BocNHCHC$\underline{H}_3$), 1.33 (d, *J* = 7.0 Hz, 3H, CHC$\underline{H}_3$).

^{13}C{^{1}H} NMR (75 MHz, Chloroform-*d*) δ 172.7, 172.3, 155.5, 135.4, 128.8, 128.6, 128.3, 80.3, 67.3, 50.1, 48.3, 28.4, 18.5.

IR (cm^{-1}): 3306, 2979, 2940, 1740, 1663, 1506, 1247, 1159, 1052.

HRMS-ESI (m/z) calculated for $C_{18}H_{26}N_2O_5Na$ $[M+Na]^+$ 373.1739, found 373.1736.

Boc-L-Ser(Bn)-L-Phe-L-Ala-OBn (12a)

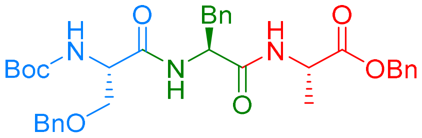

Following the general procedure (Method G) using Boc-L-Ser(Bn)-OH, Boc-L-Phe-OH and L-Ala-OBn, Boc-L-Ser(Bn)-L-Phe-L-Ala-OBn (**12a**) was obtained as a colorless liquid (303 mg, 84% yield) with 99:1 dr.

$[\alpha]_D^{23}$ = –5.7 (*c* 1.00, $CHCl_3$)

^{1}H NMR (300 MHz, Chloroform-*d*) δ 7.43 – 7.08 (m, 15H, $BocNHCHCH_2OCH_2C_6\underline{H}_5$, $CONHCHC_6\underline{H}_5$, and $CO_2CH_2C_6\underline{H}_5$), 6.78 (d, *J* = 8.2 Hz, 1H, N$\underline{H}$), 6.61 (s, 1H, N$\underline{H}$), 5.30 (s, 1H, N$\underline{H}$), 5.14 (s, 2H, $CO_2C\underline{H}_2C_6H_5$), 4.77 – 4.64 (m, 1H, $C\underline{H}CH_2C_6H_5$), 4.58 – 4.38 (m, 3H, $BocNHC\underline{H}CH_2OC\underline{H}_2C_6H_5$), 4.28 – 4.12 (m, 1H, C$\underline{H}CH_3$), 3.82 (dd, *J* = 9.4, 4.3 Hz, 1H, $BocNHCHC\underline{H}_2OCH_2C_6H_5$), 3.56 (dd, *J* = 9.4, 6.4 Hz, 1H, $BocNHCHC\underline{H}_2OCH_2C_6H_5$), 3.17 (dd, *J* = 13.9, 6.1 Hz, 1H, $CHC\underline{H}_2C_6H_5$), 3.00 (dd, *J* = 13.9, 6.4 Hz, 1H, $CHC\underline{H}_2C_6H_5$), 1.39 (s, 9H, $(C\underline{H}_3)_3COCONHCHCH_2OCH_2C_6H_5$), 1.26 (d, *J* = 7.2 Hz, 3H, CHC$\underline{H}_3$), .

$^{13}C\{^1H\}$ NMR (75 MHz, Chloroform-*d*) δ 172.2, 170.1, 155.7, 137.4, 136.3, 135.5, 129.5, 128.8, 128.7, 128.7, 128.5, 128.3, 128.1, 127.9, 127.2, 80.6, 73.6, 69.7, 67.2, 54.5, 54.1, 48.4, 37.7, 28.4, 18.1.

IR (cm^{-1}): 3289, 3028, 2978, 1743, 1688, 1646, 1524, 1366, 1246, 1164, 1051.

HRMS-ESI (m/z) calculated for $C_{34}H_{42}N_3O_7$ $[M+H]^+$ 604.3023, found 604.3019.

Boc-L-Leu-Gly-L-Ala-L-Ala-OBn (12b)

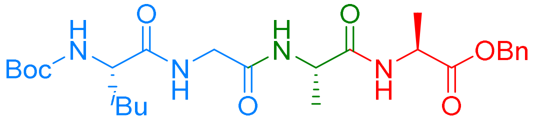

Following the general procedure (Method G) using Boc-L-Leu-Gly-OH, Boc-L-Ala-OH and L-Ala-OBn, Boc-L-Leu-Gly-L-Ala-L-Ala-OBn (**12b**) was obtained as a white solid (276 mg, 88% yield) with 99:1 dr.

M.p. 63–65 ˚C

[α]$_D^{22}$ = –8.3 (*c* 1.00, $CHCl_3$)

^1H NMR (300 MHz, Chloroform-*d*) δ 7.41 – 7.26 (m, 5H, $CO_2CH_2C_6\underline{H}_5$), 7.25 – 7.16 (m, 2H, 2 x N**H**), 7.13 (d, *J* = 7.5 Hz, 1H, N**H**), 5.29 (d, *J* = 6.8 Hz, 1H, N**H**), 5.18 (d, *J* = 12.3 Hz, 1H, $CO_2C\underline{H}_2C_6H_5$), 5.11 (d, *J* = 12.3 Hz, 1H, $CO_2C\underline{H}_2C_6H_5$), 4.70 – 4.48 (m, 2H, 2 x $C\underline{H}CH_3$), 4.21 – 4.05 (m, 1H, $BocNHC\underline{H}CH_2CH(CH_3)_2$), 4.04 – 3.85 (m, 2H, $BocNHCHCONHC\underline{H}_2CONH$), 1.75 – 1.55 (m, 2H, $BocNHCHC\underline{H}_2CH(CH_3)_2$), 1.54 – 1.45 (m, 1H, $BocNHCHCH_2C\underline{H}(CH_3)_2$), 1.45 – 1.38 (m, 12H, $(C\underline{H}_3)_3COCONHCHCONH$ and $CHC\underline{H}_3$), 1.36 (d, *J* = 7.1 Hz, 3H, $CHC\underline{H}_3$), 0.93 (d, *J* = 4.4 Hz, 3H, $BocNHCHCH_2CH(C\underline{H}_3)_2$), 0.91 (d, *J* = 4.3 Hz, 3H, $BocNHCHCH_2CH(C\underline{H}_3)_2$).

$^{13}C\{^1H\}$ NMR (75 MHz, Chloroform-*d*) δ 173.8, 172.8, 172.1, 169.0, 156.2, 135.5, 128.7, 128.5, 128.2, 80.4, 67.2, 53.8, 49.1, 48.3, 43.3, 41.3, 28.4, 24.9, 23.1, 22.0, 18.1.

IR (cm^{-1}): 3297, 3036, 2958, 1743, 1647, 1530, 1455, 1367, 1250, 1167, 1047.

HRMS-ESI (m/z) calculated for $C_{26}H_{41}N_4O_7$ $[M+H]^+$ 521.2975, found 521.2977.

Boc-L-Phe-L-Leu-Gly-L-Ala-OBn (12c)

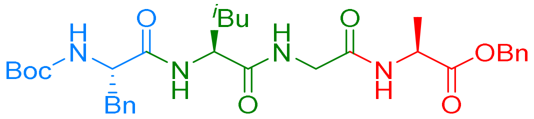

Following the general procedure (Method G) using Boc-L-Phe-OH, Boc-L-Leu-Gly-OH and L-Ala-OBn, Boc-L-Phe-L-Leu-Gly-L-Ala-OBn (**12c**) was obtained as a white solid (286 mg, 80% yield) with 99:1 dr.

M.p. 53–55 ˚C

[α]$_D^{22}$ = –11.9 (*c* 1.00, $CHCl_3$)

^{1}H NMR (300 MHz, Chloroform-*d*) δ 7.40 – 7.19 (m, 10H, BocNHCHCH$_2$C$_6$***H***$_5$ and CO$_2$CH$_2$C$_6$***H***$_5$), 7.11 (d, *J* = 7.4 Hz, 1H, N***H***), 7.05 – 6.94 (m, 1H, N***H***), 6.65 (d, *J* = 7.3 Hz, 1H,N***H***), 5.23 (d, *J* = 12.4 Hz, 1H, CO$_2$C***H***$_2$C$_6$H$_5$), 5.17 (d, *J* = 12.4 Hz, 1H, CO$_2$C***H***$_2$C$_6$H$_5$), 5.14 (d, *J* = 6.2 Hz, 1H, N***H***), 4.64 (p, *J* = 7.2 Hz, 1H, C***H***CH$_3$), 4.54 – 4.21 (m, 2H, BocNHC***H***CH$_2$C$_6$H$_5$), 4.11 – 3.76 (m, 2H, C***H***CH$_2$CH(CH$_3$)$_2$), 3.16 – 2.99 (m, 2H, BocNHCHC***H***$_2$C$_6$H$_5$), 1.75 – 1.49 (m, 3H, CHC***H***$_2$C***H***(CH$_3$)$_2$), 1.45 (d, *J* = 7.2 Hz, 3H, CHC***H***$_3$), 1.43 (s, 9H, (C***H***$_3$)$_3$COCONHCHCONH), 0.94 (d, *J* = 4.0 Hz, 3H, BocNHCHCH$_2$CH(C***H***$_3$)$_2$), 0.92 (d, *J* = 4.0 Hz, 3H, BocNHCHCH$_2$CH(C***H***$_3$)$_2$).

^{13}C{^{1}H} NMR (75 MHz, Chloroform-*d*) δ 173.3, 172.9, 172.6, 169.2, 156.5, 137.0, 136.2, 130.0, 129.5, 129.3, 129.1, 128.8, 127.8, 81.4, 67.8, 56.7, 53.0, 48.9, 43.8, 41.2, 38.5, 29.0, 25.3, 23.7, 22.5, 18.7.

IR (cm^{-1}): 3291, 3064, 2958, 1744, 1642, 1527, 1455, 1367, 1250, 1164, 1050.

HRMS-ESI (m/z) calculated for $C_{32}H_{44}N_4O_7Na$ [M+Na]$^+$ 619.3108, found 619.3108.

Boc-L-Glu(^{t}Bu)-L-Leu-Gly-L-Met-L-Ala-OBn (12d)

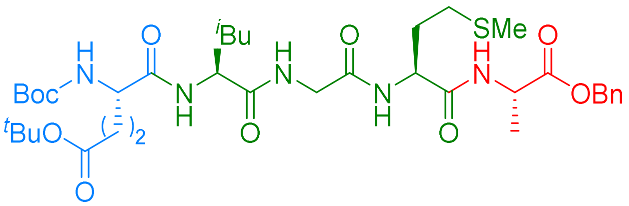

Following the general procedure (Method G) using Boc-L-Glu(^{t}Bu)-OH, Boc-L-Leu-Gly-OH, Boc-L-Met-OH and L-Ala-OBn, Boc-L-Glu(^{t}Bu)-L-Leu-Gly-L-Met-L-Ala-OBn (**12d**) was obtained as a white solid (233 mg, 51% yield) with 99:1 dr.

M.p. 162–164 ˚C

[α]$_D^{22}$ = –15.0 (*c* 1.00, CHCl$_3$)

^{1}H NMR (300 MHz, Chloroform-*d*) δ 7.85 (s, 1H, N***H***), 7.52 (s, 1H, N***H***), 7.36 – 7.31 (m, 6H, CO$_2$CH$_2$C$_6$***H***$_5$ and N***H***), 7.20 (s, 1H, N***H***), 6.04 (s, 1H, N***H***), 5.22 (d, *J* = 12.4 Hz, 1H, CO$_2$C***H***$_2$C$_6$H$_5$), 5.13 (d, *J* = 12.4 Hz, 1H, CO$_2$C***H***$_2$C$_6$H$_5$), 4.78 – 3.59 (m, 6H, BocNHC***H***CH$_2$CH$_2$CO$_2$C(CH$_3$)$_3$, C***H***CH$_2$CH(CH$_3$)$_2$, NHC***H***$_2$CONH, C***H***CH$_2$CH$_2$SCH$_3$ and C***H***CH$_3$), 2.53 (t, *J* = 7.7 Hz, 2H, CHCH$_2$C***H***$_2$SCH$_3$), 2.41 – 2.31 (m, 2H, BocNHCHCH$_2$C***H***$_2$CO$_2$C(CH$_3$)$_3$), 2.08 – 1.91 (m, 7H, BocNHCHC***H***$_2$CH$_2$CO$_2$C(CH$_3$)$_3$ and CHC***H***$_2$CH$_2$SC***H***$_3$), 1.74 – 1.61 (m, 2H, CHC***H***$_2$CH(CH$_3$)$_2$), 1.46 – 1.43 (m, 4H, CHCH$_2$C***H***(CH$_3$)$_2$ and CHC***H***$_3$), 1.42 (s, 9H, (C***H***$_3$)$_3$COCONHCHCH$_2$CH$_2$CO$_2$C(CH$_3$)$_3$), 1.40 (s, 9H, BocNHCHCH$_2$CH$_2$CO$_2$C(C***H***$_3$)$_3$), 0.93 (d, *J* = 6.1 Hz, 3H, CHCH$_2$CH(C***H***$_3$)$_2$), 0.91 (d, *J* = 5.9 Hz, 3H, CHCH$_2$CH(C***H***$_3$)$_2$).

^{13}C{^{1}H} NMR (75 MHz, Chloroform-*d*) δ 173.2, 172.8, 172.7, 171.8, 171.0, 169.2, 156.5, 135.6, 128.7, 128.4, 128.2, 81.3, 80.6, 67.2, 55.3, 52.7, 52.4, 48.3, 43.3, 41.2, 32.2, 32.0, 30.1, 28.4, 28.2, 27.3, 25.0, 23.1, 22.2, 18.1, 15.3.

IR (cm^{-1}): 3282, 3078, 2977, 1730, 1632, 1525, 1367, 1249, 1155.

HRMS-ESI (m/z) calculated for $C_{37}H_{59}N_5O_{10}SNa$ [M+Na]$^+$ 788.3880, found 788.3878.

Boc-L-Thr(Bn)-L-Ile-L-His(Bn)-L-Ser(Bn)-L-Val-L-Met-L-Leu-Gly-L-Glu(Bn)-L-Ala-OBn (12e)

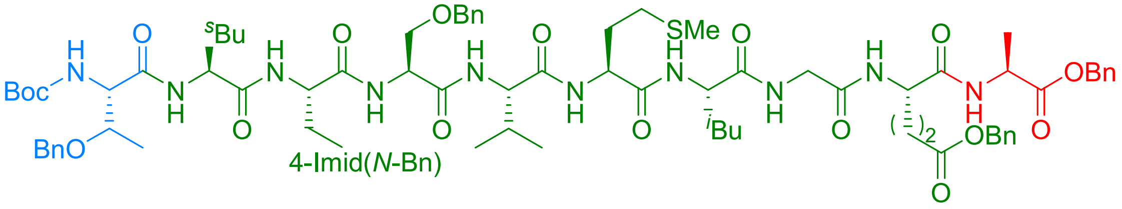

Following the general procedure (Method G) using Boc-L-Thr(Bn)-OH, Boc-L-Ile-OH, Boc-His(Bn)-OH, Boc-L-Ser(Bn)-OH, Boc-L-Val-OH, Boc-Met-OH, Boc-L-Leu-Gly-OH, L-Glu(Bn)-OBn and L-Ala-OBn, Boc-L-Thr(Bn)-L-Ile-L-His(Bn)-L-Ser(Bn)-L-Val-L-Met-L-Leu-Gly-L-Glu(Bn)-L-Ala-OBn (**12e**) was obtained as a white solid (131 mg, 14% yield) with 99:1 dr.

M.p. 178–180 ˚C

[α]$_D$^{22} = –2.2 (*c* 1.00, $CHCl_3$)

^{1}H NMR (300 MHz, Chloroform-*d*) δ 7.73 – 7.49 (m, 1H, N***H***), 7.48 – 6.71 (m, 34H, $CHCH_2$<u>Imid***H***$_2$</u>($CH_2C_6H_5$), 5 x CH_2C_6***H***$_5$ and 7 x N***H***), 5.58 – 5.31 (m, 1H, N***H***), 5.29 – 4.75 (m, 8H, N***H***, C***H***CH_2Imid(C***H***$_2C_6H_5$) and 2 x CO_2C***H***$_2C_6H_5$), 4.75 – 4.26 (m, 10H, C***H***CH_2OC***H***$_2C_6H_5$, C***H***$CH(CH_3)OC$***H***$_2C_6H_5$, C***H***$CH_2CH_2SCH_3$, C***H***$CH_2CH(CH_3)_2$ and C***H***$CH_2CH_2CO_2CH_2C_6H_5$ and C***H***CH_3), 4.26 – 4.07 (m, 3H, C***H***$CH(CH_3)CH_2CH_3$, C***H***$CH(CH_3)_2$ and CHC***H***$(CH_3)OCH_2C_6H_5$), 4.04 – 3.76 (m, 3H, NHC***H***$_2$CONH and CHC***H***$_2OCH_2C_6H_5$), 3.75 – 3.54 (m, 1H, CHC***H***$_2OCH_2C_6H_5$), 3.28 – 2.75 (m, 1H, CHC***H***$_2$Imid($CH_2C_6H_5$)), 2.71 – 1.37 (m, 31H, CHC***H***$_2$Imid($CH_2C_6H_5$), CHC***H***(CH_3)C***H***$_2CH_3$, CHC***H***$(CH_3)_2$, CHC***H***$_2$C***H***$_2$SC***H***$_3$, CHC***H***$_2$C***H***$_2CO_2CH_2C_6H_5$, CHC***H***$_2$C***H***$(CH_3)_2$, CHC***H***$_3$, and (C***H***$_3$)$_3$COCONH), 1.30 – 1.20 (m, 6H, CHCH(C***H***$_3$)$OCH_2C_6H_5$ and CHCH(C***H***$_3$)CH_2CH_3), 1.07 – 0.64 (m, 15H, $CHCH(CH_3)CH_2C$***H***$_3$, CHCH(C***H***$_3$)$_2$ and $CHCH_2CH$(C***H***$_3$)$_2$).

^{13}C NMR (75 MHz, Chloroform-*d*) δ 173.6, 173.1, 173.0, 172.9, 172.8, 172.7, 172.5, 172.5, 172.3, 171.1, 169.5, 156.3, 138.1, 136.1, 136.0, 135.9, 135.6, 135.5, 135.3, 129.3, 128.8, 128.7, 128.5, 128.5, 128.4, 128.2, 128.2, 127.8, 79.9, 75.1, 73.3, 73.2, 71.6, 71.3, 67.5, 67.4, 67.3, 67.1, 66.9, 66.6, 66.4, 66.3, 52.5, 48.7, 32.1, 30.3, 30.2, 29.8, 29.5, 28.4, 28.2, 25.0, 24.9, 22.8, 22.4, 17.9, 17.7, 17.6, 16.5, 14.3, 11.3.

IR (cm^{-1}): 3276, 3032, 2965, 1721, 1629, 1533, 1455, 1212, 1164.

HRMS-ESI (m/z) calculated for $C_{85}H_{115}N_{12}O_{17}S$ $[M+H]^+$ 1607.8224, found 1607.8213.

Fmoc-L-Ala-L-Ala-OH (13a)

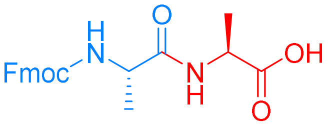

Following the general procedure (Method H) using Fmoc-L-Ala-OH and Fmoc-Ala-Wang resin, Fmoc-L-Ala-L-Ala-OH (**13a**) was obtained as a white solid (220 mg, 96% yield) with 99:1 dr. The characterization data matched previously reported data.[21]

M.p. 198–200 ˚C

$[\alpha]_D^{23}$ = –20.9 (*c* 1.00, MeOH)

^{1}H NMR (300 MHz, DMSO-d_6) δ 8.12 (d, *J* = 7.3 Hz, 1H, N$\underline{\boldsymbol{H}}$), 7.87 (d, *J* = 7.5 Hz, 2H, $C_{12}\underline{\boldsymbol{H}}_8CHCH_2OCONH$), 7.71 (t, *J* = 6.4 Hz, 2H, $C_{12}\underline{\boldsymbol{H}}_8CHCH_2OCONH$), 7.49 (d, *J* = 7.8 Hz, 1H, N$\underline{\boldsymbol{H}}$), 7.40 (t, *J* = 7.5 Hz, 2H, $C_{12}\underline{\boldsymbol{H}}_8CHCH_2OCONH$), 7.31 (t, *J* = 7.4 Hz, 2H, $C_{12}\underline{\boldsymbol{H}}_8CHCH_2OCONH$), 4.32 – 4.13 (m, 4H, $C_{12}H_8C\underline{\boldsymbol{H}}C\underline{\boldsymbol{H}}_2OCONH$ and FmocNHC$\underline{\boldsymbol{H}}CH_3$), 4.13 – 4.00 (m, 1H, C$\underline{\boldsymbol{H}}CH_3$), 1.26 (d, *J* = 7.3 Hz, 3H, FmocNHCHC$\underline{\boldsymbol{H}}_3$), 1.21 (d, *J* = 7.1 Hz, 3H, CHC$\underline{\boldsymbol{H}}_3$).

^{13}C NMR (75 MHz, DMSO-d_6) δ 174.6, 172.9, 156.2, 144.5, 144.4, 141.3, 128.2, 127.7, 125.9, 120.7, 66.2, 50.3, 48.0, 47.2, 18.8, 17.7.

IR (cm^{-1}): 3313, 3067, 2944, 1718, 1660, 1534, 1367, 1217.

HRMS-ESI (m/z) calculated for $C_{21}H_{22}N_2O_5Na$ $[M+Na]^+$ 405.1426, found 405.1425.

Fmoc-L-Ser-L-Ala-OH (13b)

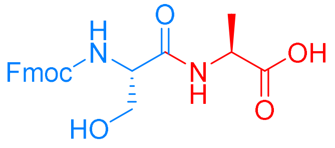

Following the general procedure (Method H) using Fmoc-L-Ser(^{t}Bu)-OH and Fmoc-Ala-Wang resin, Fmoc-L-Ser-L-Ala-OH (**13b**) was obtained as a white solid (208 mg, 87% yield) with 99:1 dr. The characterization data matched previously reported data.[22]

M.p. 130–132 ˚C

$[\alpha]_D^{23}$ = –12.3 (*c* 1.00, MeOH)

^{1}H NMR (300 MHz, DMSO-d_6) δ 8.11 (d, *J* = 7.3 Hz, 1H, N$\underline{\boldsymbol{H}}$), 7.87 (d, *J* = 7.5 Hz, 2H, $C_{12}\underline{\boldsymbol{H}}_8CHCH_2OCONH$), 7.77 – 7.67 (m, 2H, $C_{12}\underline{\boldsymbol{H}}_8CHCH_2OCONH$), 7.46 – 7.36 (m, 2H, $C_{12}\underline{\boldsymbol{H}}_8CHCH_2OCONH$), 7.35 – 7.25 (m, 3H, $C_{12}\underline{\boldsymbol{H}}_8CHCH_2OCONH$ and N$\underline{\boldsymbol{H}}$), 4.35 – 4.06 (m, 5H, $C_{12}H_8C\underline{\boldsymbol{H}}C\underline{\boldsymbol{H}}_2OCONH$, C$\underline{\boldsymbol{H}}CH_3$, and FmocNHC$\underline{\boldsymbol{H}}CH_2$OH), 3.67 – 3.46 (m, 2H, FmocNHCHC$\underline{\boldsymbol{H}}_2$OH), 3.35 (s, 1H, FmocNHCHCH$_2$O$\underline{\boldsymbol{H}}$), 1.26 (d, *J* = 7.2 Hz, 3H, CHC$\underline{\boldsymbol{H}}_3$).

^{13}C NMR (75 MHz, DMSO-d_6) δ 174.6, 170.5, 156.6, 144.5, 144.4, 141.3, 128.2, 127.7, 125.9, 120.7, 66.3, 62.4, 57.7, 48.1, 47.2, 17.9.

IR (cm^{-1}): 3295, 3067, 2944, 1710, 1651, 1535, 1451, 1235.

HRMS-ESI (m/z) calculated for $C_{21}H_{23}N_2O_6$ $[M+H]^+$ 399.1556, found 399.1555.

Fmoc-L-Thr-L-Ala-OH (13c)

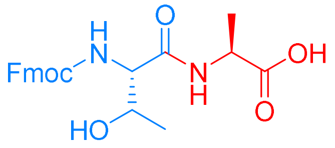

Following the general procedure (Method H) using Fmoc-L-Thr(^{t}Bu)-OH and Fmoc-Ala-Wang resin, Fmoc-L-Thr-L-Ala-OH (**13c**) was obtained as a white solid (0.1995 mg, 80% yield) with 99:1 dr. The characterization data matched previously reported data.[23]

M.p. 185–187 ˚C

[α]$_D^{23}$ = –20.1 (*c* 1.00, MeOH)

^{1}H NMR (300 MHz, DMSO-d_6) δ 8.09 (d, *J* = 7.1 Hz, 1H, N***H***), 7.88 (d, *J* = 7.5 Hz, 2H, C_{12}***H***$_8$CHCH$_2$OCONH), 7.78 – 7.66 (m, 2H, C_{12}***H***$_8$CHCH$_2$OCONH), 7.45 – 7.36 (m, 2H, C_{12}***H***$_8$CHCH$_2$OCONH), 7.36 – 7.26 (m, 2H, C_{12}***H***$_8$CHCH$_2$OCONH), 7.14 – 7.05 (m, 1H, N***H***), 4.35 – 4.13 (m, 4H, $C_{12}H_8$C***H***C***H***$_2$OCONH and FmocNHCHC***H***(CH$_3$)OH), 3.98 – 3.82 (m, 2H, FmocNHC***H***CH$_3$ and C***H***CH$_3$), 3.40 (s, 1H, FmocNHCHCH (CH$_3$)O***H***), 1.27 (d, *J* = 7.3 Hz, 3H, CHC***H***$_3$), 1.06 (d, *J* = 6.1 Hz, 3H, FmocNHCHCH(C***H***$_3$)OH).

^{13}C NMR (75 MHz, DMSO-d_6) δ 174.6, 170.6, 156.6, 144.5, 144.4, 141.3, 128.2, 127.7, 125.9, 120.7, 67.4, 66.3, 61.0, 48.1, 47.3, 20.4, 17.9.

IR (cm^{-1}): 3318, 3064, 2983, 1711, 1656, 1530, 1451, 1247.

HRMS-ESI (m/z) calculated for $C_{22}H_{25}N_2O_6$ [M+H]$^+$ 413.1713, found 413.1711.

Fmoc-L-Lys-L-Ala-OH (13d)

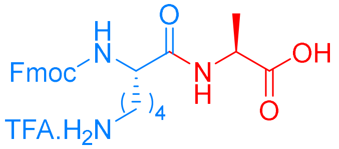

Following the general procedure (Method H) using Fmoc-L-Lys(Boc)-OH and Fmoc-Ala-Wang resin, the trifluoroacetate salt of Fmoc-L-Lys-L-Ala-OH (**13d**) was obtained as a white solid (328 mg, 98% yield) with 99:1 dr.

M.p. 148–150 ˚C

[α]$_D^{24}$ = –12.9 (*c* 1.00, MeOH)

^{1}H NMR (300 MHz, DMSO-d_6) δ 8.18 (d, *J* = 7.2 Hz, 1H, N***H***), 7.87 (d, *J* = 7.5 Hz, 2H, C_{12}***H***$_8$CHCH$_2$OCONH), 7.78 (s, 2H, CH$_2$CH$_2$CH$_2$CH$_2$N***H***$_2$), 7.74 – 7.66 (m, 2H, C_{12}***H***$_8$CHCH$_2$OCONH), 7.46 (d, *J* = 8.3 Hz, 1H, N***H***), 7.43 – 7.36 (m, 2H, C_{12}***H***$_8$CHCH$_2$OCONH), 7.36 – 7.25 (m, 2H, C_{12}***H***$_8$CHCH$_2$OCONH), 4.36 – 4.08 (m, 4H, $C_{12}H_8$C***H***C***H***$_2$OCONH and C***H***CH$_3$), 4.07 – 3.93 (m, 1H, FmocNHC***H***), 2.75 (s, 2H, CH$_2$CH$_2$CH$_2$C***H***$_2$NH$_2$), 1.73 – 1.43 (m, 4H, C***H***$_2$CH$_2$C***H***$_2$CH$_2$NH$_2$), 1.34 (q, *J* = 7.8 Hz, 2H, CH$_2$C***H***$_2$CH$_2$CH$_2$NH$_2$), 1.26 (d, *J* = 7.3 Hz, 3H, CHC***H***$_3$).

^{13}C NMR (75 MHz, DMSO-d_6) δ 174.6, 172.3, 156.5, 144.5, 144.4, 141.3, 128.2, 127.7, 125.9, 120.7, 66.2, 54.6, 48.0, 47.3, 31.9, 27.2, 22.9, 17.7.

IR (cm^{-1}): 3298, 3057, 2942, 1684, 1649, 1534, 1451, 1202.

HRMS-ESI (m/z) calculated for $C_{24}H_{30}N_3O_5$ [M+H]$^+$ 440.2185, found 440.2184.

Fmoc-L-Lys-L-Glu-Ser-Val-Ala-OH (13e)

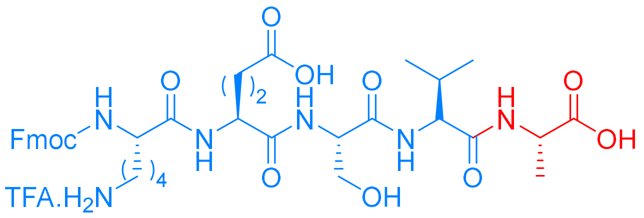

Following the general procedure (Method H) using Fmoc-L-Lys(Boc)-OH, Fmoc-L-Glu(^{t}Bu)-OH, Fmoc-L-Ser(^{t}Bu)-OH, Fmoc-L-Val-OH and Fmoc-L-Ala-Wang resin, the trifluoroacetate salt of Fmoc-L-Lys-L-Glu-Ser-Val-Ala-OH (**13e**) was obtained as a white solid (376 mg, 83% yield) with 99:1 dr.

M.p. 200–202 ˚C

$[\alpha]_D^{24}$ = –26.9 (*c* 1.00, MeOH)

^{1}H NMR (300 MHz, DMSO-d_6) δ 8.24 (d, *J* = 7.7 Hz, 1H, N$\underline{\boldsymbol{H}}$), 8.07 – 8.01 (m, 1H, N$\underline{\boldsymbol{H}}$), 7.88 – 7.69 (m, 6H, $C_{12}\underline{\boldsymbol{H}}_8CHCH_2OCONH$ and 2 x N$\underline{\boldsymbol{H}}$), 7.53 (d, *J* = 8.0 Hz, 1H, N$\underline{\boldsymbol{H}}$), 7.42 – 7.29 (m, 4H, $C_{12}\underline{\boldsymbol{H}}_8CHCH_2OCONH$), 4.88 – 3.29 (m, 11H, , $C_{12}H_8C\underline{\boldsymbol{H}}C\underline{\boldsymbol{H}}_2OCONH$, 5 x NHC$\underline{\boldsymbol{H}}$CO and $NHCHC\underline{\boldsymbol{H}}_2O\underline{\boldsymbol{H}}$), 2.79 (q, *J* = 7.1 Hz, 2H, $CHCH_2CH_2CH_2C\underline{\boldsymbol{H}}_2NH_2$), 2.26 – 1.12 (m, 16H, $CHC\underline{\boldsymbol{H}}(CH_3)_2$ $CHC\underline{\boldsymbol{H}}_2C\underline{\boldsymbol{H}}_2CO_2H$, $CHC\underline{\boldsymbol{H}}_2C\underline{\boldsymbol{H}}_2C\underline{\boldsymbol{H}}_2CH_2N\underline{\boldsymbol{H}}_2$ and $CHC\underline{\boldsymbol{H}}_3$), 0.89 (d, *J* = 6.8 Hz, 3H, $CHCH(C\underline{\boldsymbol{H}}_3)_2$), 0.85 (d, *J* = 6.6 Hz, 3H, $CHCH(C\underline{\boldsymbol{H}}_3)_2$).

^{13}C NMR (101 MHz, DMSO-d_6) δ 174.5, 174.4, 172.3, 171.6, 171.0, 170.2, 156.5, 144.3, 144.2, 141.2, 128.1, 127.5, 125.7, 120.5, 66.0, 62.0, 57.6, 55.3, 54.8, 52.1, 47.9, 47.1, 31.4, 30.4, 28.1, 27.5, 27.0, 22.9, 19.5, 18.1, 17.4.

IR (cm^{-1}): 3280, 3071, 2965, 1629, 1537, 1451, 1201.

HRMS-ESI (m/z) calculated for $C_{37}H_{51}N_6O_{11}$ $[M+H]^+$ 755.3616, found 755.3612.

Classical synthetic methodologies in peptide synthesis

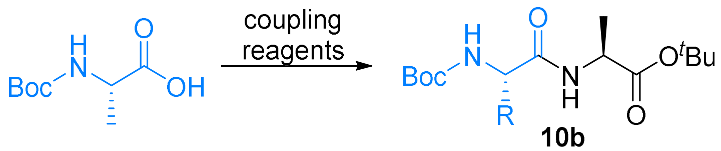

10b

DCC/HOBt

Boc-L-Ala-OH (0.6 mmol, 1 equiv.) was dissolved in DMF (10 mL), and the solution was cooled in an ice bath to 0 °C. DCC (0.72 mmol, 1.2 equiv.) and HOBt (0.72 mmol, 1.2 equiv.) were added and stirred for 30 min at 0 °C. L-Ala-O^{t}Bu (0.6 mmol, 1 equiv.) was subsequently added, and the resulting mixture was stirred at room temperature for 4 h. After the completion of the reaction the organic layer was diluted with EtOAc (20 mL) and washed with 1.0 M HCl (30 mL), 0.1 M $NaHCO_3$ (30 mL), and brine (30 mL). The organic phase was dried over Na_2SO_4, and the solvent was evaporated under reduced pressure. The crude was purified by flash column chromatography (10–50% EtOAc in hexane) to provide Boc-L-Ala-L-Ala-O^{t}Bu (**10b**) as a white solid (79 mg, 42% yield) with 99:1 dr.

PyBOP

Boc-L-Ala-OH (0.6 mmol, 1 equiv.) was dissolved in DCM (10 mL). PyBOP (0.6 mmol, 1 equiv.), L-Ala-O^{t}Bu (0.6 mmol, 1 equiv.), and DIPEA (1.2 mmol, 2 equiv.) were added and stirred at room temperature for 4 h. After the completion of the reaction the organic layer was diluted with DCM (20 mL) and washed with 1.0 M HCl (30 mL), 0.1 M $NaHCO_3$ (30 mL), and brine (30 mL). The organic phase was dried over Na_2SO_4, and the solvent was evaporated under reduced pressure. The crude was purified by flash column chromatography (10–50% EtOAc in hexane) to provide Boc-L-Ala-L-Ala-O^{t}Bu (**10b**) as a white solid (145 mg, 77% yield) with 99:1 dr.

HBTU

Boc-L-Ala-OH (0.6 mmol, 1 equiv.) was dissolved in 10 mL of dry DMF together with HBTU (0.9 mmol, 1.5 equiv.). The solution was cooled in an ice bath to 0 °C, DIPEA (2.1 mmol, 3.5 eq) was added, and the mixture was allowed to react for 4 h. L-Ala-O^{t}Bu (0.6 mmol, 1 equiv.) was subsequently added, and the resulting mixture was stirred at room temperature for 4 h. After the completion of the reaction the organic layer was diluted with EtOAc (20 mL) and washed 1 M HCl (30 mL), 0.1 M $NaHCO_3$ (30 mL), and brine (30 mL). The organic phase was dried over Na_2SO_4, and the solvent was evaporated under reduced pressure. The crude was purified by flash column chromatography (10–50% EtOAc in hexane) to provide Boc-L-Ala-L-Ala-O^{t}Bu (**10b**) as a white solid (144 mg, 76% yield) with 99:1 dr.

Carboxylic acid kinetic experiments monitored by ^{19}F NMR spectroscopy

SOF_2

Two 20 mL vials equipped with magnetic stir-bars were capped with septum-fitted vial caps connected by a 6 mL syringe-filled imidazole column (2.7 g, 40 mmol) (Figure S2). To vial B was added DCM (10 mL). To vial A was charged KHF_2 (0.702 g, 9 mmol, 3 equiv.) followed by thionyl chloride (0.22 mL, 3 mmol, 1 equiv.) in one portion (Note: leave the syringe in vial A). The bubbling rate was slow at the beginning, then increased over 30 mins. Vial B was vented with an empty balloon when the bubbling subsided (this triggered more bubbling of SOF_2 through the solution). The tubing was removed when the bubbling subsided again. The mixture was analyzed by ^{19}F NMR spectroscopy with trifluorotoluene as internal standard (0.70 M SOF_2 in DCM). The vial B was charged with carboxylic acid (0.6 mmol, 1 equiv.) and pyridine (48 µL, 0.6 mmol, 1 equiv.). The mixture was stirred at room temperature and aliquots were taken and diluted in $CDCl_3$ for quantitative ^{19}F NMR spectroscopy.

(Note: SOF_2 is a toxic gas and all work should be performed in a fume hood.)

SO_2F_2

Two 20 mL vials equipped with magnetic stir-bars were capped with septum-fitted vial caps connected by a 6 mL syringe-filled imidazole column (2.7 g, 40 mmol) (Figure S2). To vial B was added DCM (10 mL). To vial A was charged 1,1'-sulfonyldiimidazole (5.53 mmol, 9.2 equiv.) and anhydrous KF (14.7 mmol, 24.5 equiv) followed by TFA (3 mL) in one portion (Note: leave the syringe in vial A). Vigorous bubbling of SO_2F_2 and fuming were observed in vial B for 1 minute and when the bubbling subsided, vial B was vented via an empty balloon for approximately 30 seconds (this triggered more bubbling of SO_2F_2 through the solution). The tubing was removed when the bubbling subsided again. The mixture was analyzed by ^{19}F NMR spectroscopy with trifluorotoluene as internal standard (0.72 M SO_2F_2 in DCM). The vial B was charged with carboxylic acid (0.6 mmol, 1 equiv.) and pyridine (48 µL, 0.6 mmol, 1 equiv.). The mixture was stirred at room temperature and aliquots were taken and diluted in $CDCl_3$ for quantitative ^{19}F NMR spectroscopy.

(Note: SO_2F_2 is a toxic gas and all work should be performed in a fume hood.)

References

1 S. B. Munoz, H. Dang, X. Ispizua-Rodriguez, T. Mathew and G. K. S. Prakash, *Org. Lett.*, 2019, **21**, 1659–1663.

2 T. Scattolin, K. Deckers and F. Schoenebeck, *Org. Lett.*, 2017, **19**, 5740–5743.

3 A. Boreux, K. Indukuri, F. Gagosz and O. Riant, *ACS Catal.*, 2017, **7**, 8200–8204.

4 J. Radebner, M. Leypold, A. Eibel, J. Maier, L. Schuh, A. Torvisco, R. Fischer, N. Moszner, G. Gescheidt, H. Stueger and M. Haas, *Organometallics*, 2017, **36**, 3624–3632.

5 Y. Liang, Z. Zhao and N. Shibata, *Commun. Chem.*, 2020, **3**, 59.

6 M. Dinda, C. Bose, T. Ghosh and S. Maity, *RSC Adv.*, 2015, **5**, 44928–44932.

7 W.-M. Cheng, R. Shang, B. Zhao, W.-L. Xing and Y. Fu, *Org. Lett.*, 2017, **19**, 4291–4294.

8 P. J. Foth, T. C. Malig, H. Yu, T. G. Bolduc, J. E. Hein and G. M. Sammis, *Org. Lett.*, 2020, **22**, 6682–6686.

9 B. Tan, N. Toda and C. F. Barbas III, *Angew. Chemie Int. Ed.*, 2012, **51**, 12538–12541.

10 J. Han, W. Zhou, P.-C. Zhang, H. Wang, R. Zhang, H.-H. Wu and J. Zhang, *ACS Catal.*, 2019, **9**, 6890–6895.

11 F. Beaulieu, L.-P. Beauregard, G. Courchesne, M. Couturier, F. LaFlamme and A. L'Heureux, *Org. Lett.*, 2009, **11**, 5050–5053.

12 L. A. Carpino, E.-S. M. E. Mansour and D. Sadat-Aalaee, *J. Org. Chem.*, 1991, **56**, 2611–2614.

13 G. A. R. Y. Suaifan, M. F. Mahon, T. Arafat and M. D. Threadgill, *Tetrahedron*, 2006, **62**, 11245–11266.

14 V. V Sureshbabu, H. P. Hemantha and S. A. Naik, *Tetrahedron Lett.*, 2008, **49**, 5133–5136.

15 L. A. Carpino, D. Sadat-Aalaee, H. G. Chao and R. H. DeSelms, *J. Am. Chem. Soc.*, 1990, **112**, 9651–9652.

16 B. J. van Lierop, W. R. Jackson and A. J. Robinson, *Tetrahedron*, 2010, **66**, 5357–5366.

17 W. Muramatsu, T. Hattori and H. Yamamoto, *J. Am. Chem. Soc.*, 2019, **141**, 12288–12295.

18 W. Muramatsu, C. Manthena, E. Nakashima and H. Yamamoto, *ACS Catal.*, 2020, **10**, 9594–9603.

19 T. Ohshima, Y. Hayashi, K. Agura, Y. Fujii, A. Yoshiyama and K. Mashima, *Chem. Commun.*, 2012, **48**, 5434–5436.

20 A. S. Y. Yim and M. Wills, *Tetrahedron*, 2005, **61**, 7994–8004.

21 H. Bukya, K. Nayani, P. Gangireddy and P. S. Mainkar, *European J. Org. Chem.*, 2020, **2020**, 5358–5362.

22 A. R. Katritzky, P. Angrish and K. Suzuki, *Synthesis (Stuttg).*, 2006, **2006**, 411–424.

23 M. Gobbo, L. Biondi, F. Filira, R. Rocchi and V. Lucchini, *Tetrahedron*, 1988, **44**, 887–893.

<u>HPLC data</u>

Boc-Gly-DL-Ala-O^{t}Bu

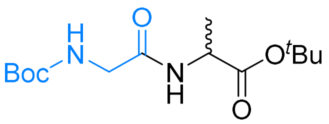

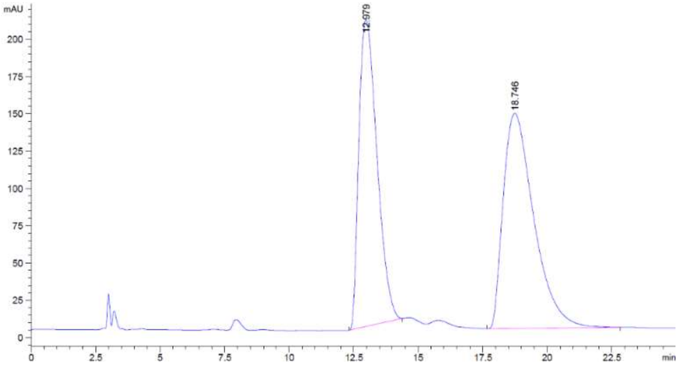

Peak#	RetTime	Area	Area%
1	12.979	1.00692e4	45.7889
2	18.746	1.19213e4	54.2111

12.979 min = Boc-Gly-D-Ala-O^{t}Bu; 18.746 min = Boc-Gly-L-Ala-O^{t}Bu

Conditions: 2-propanol/hexane = 3:97, v = 1.0 mL/min, λ = 210 nm, temperature = 25 ˚C

Chiral Column: AD-H column from TharSFC

Boc-Gly-L-Ala-O^{t}Bu (10a)

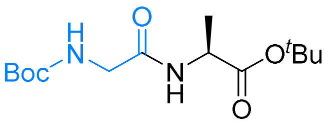

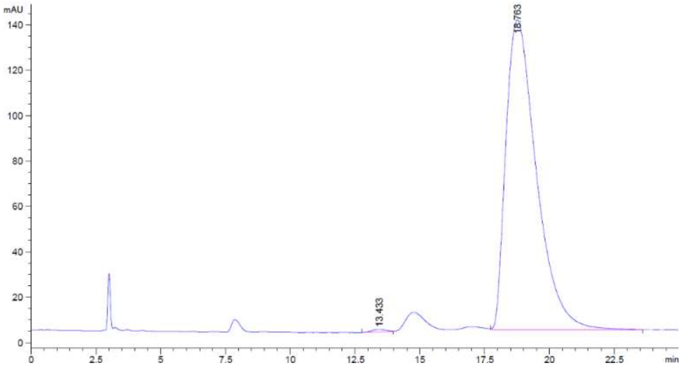

Peak#	RetTime	Area	Area%
1	13.433	40.11262	0.3574
2	18.763	1.11845e4	99.6426

Boc-DL-Ala-L-Ala-O^{t}Bu

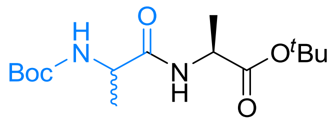

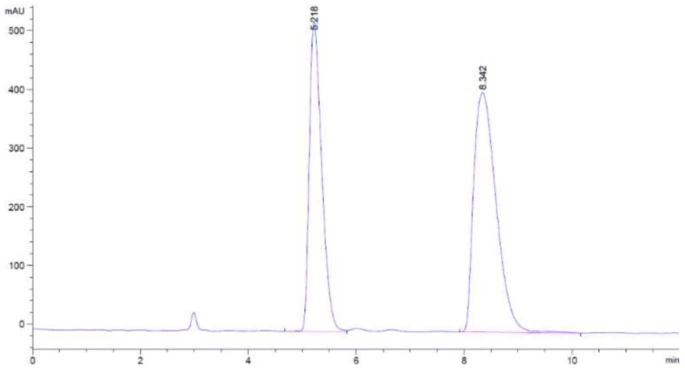

Peak#	RetTime	Area	Area%
1	5.218	8468.95703	41.7277
2	8.342	1.18268e4	58.2723

5.218 min = Boc-L-Ala-L-Ala-O^{t}Bu; 8.342 min = Boc-D-Ala-L-Ala-O^{t}Bu

Conditions: 2-propanol/hexane = 3:97, v = 1.0 mL/min, λ = 210 nm, temperature = 25 ˚C

Chiral Column: AD-H column from TharSFC

Boc-L-Ala-L-Ala-O^{t}Bu (10b)

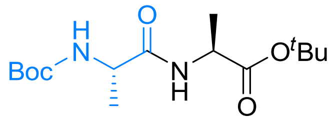

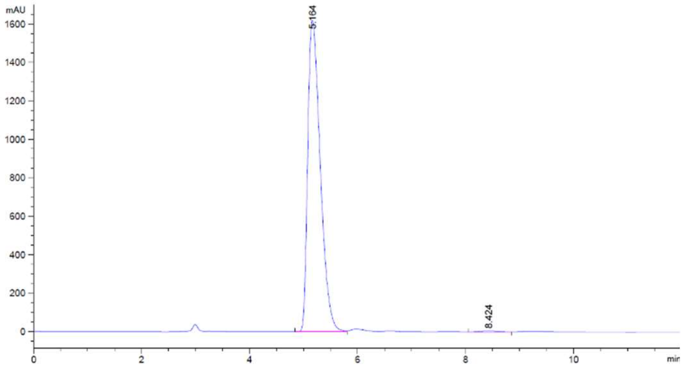

Peak#	RetTime	Area	Area%
1	5.164	2.60862e4	99.6177
2	8.424	100.10620	0.3823

Boc-DL-Phe-L-Ala-O^{t}Bu

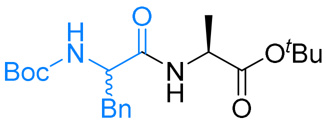

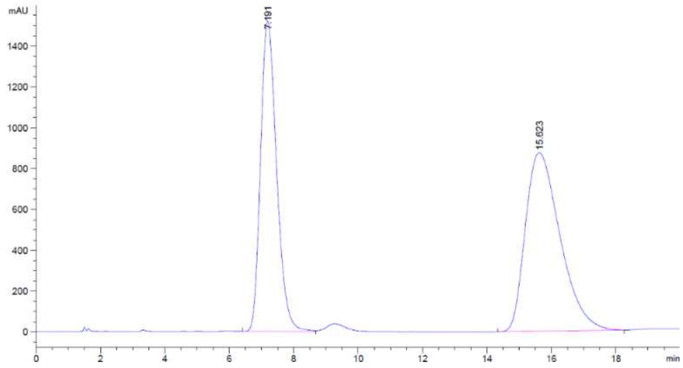

Peak#	RetTime	Area	Area%
1	7.191	5.25840e4	44.5345
2	15.623	6.54907e4	55.4655

7.191 min = Boc-L-Phe-L-Ala-O^{t}Bu; 15.623 min = Boc-D-Phe-L-Ala-O^{t}Bu

Conditions: 2-propanol/hexane = 3:97, v = 2.0 mL/min, λ = 210 nm, temperature = 25 ˚C

Chiral Column: AD-H column from TharSFC

Boc-L-Phe-L-Ala-O^{t}Bu (10c)

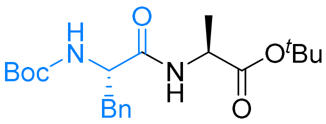

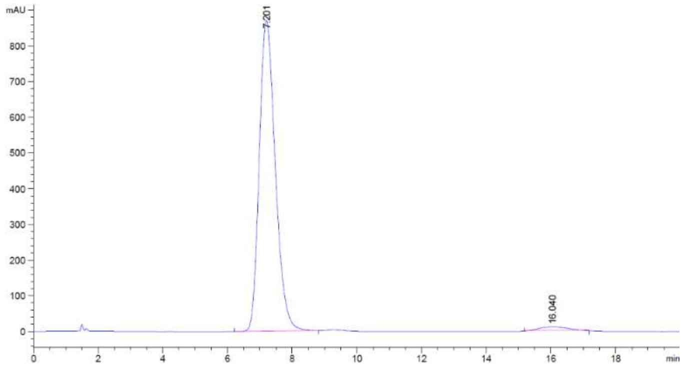

Peak#	RetTime	Area	Area%
1	7.201	2.99678e4	98.1030
2	16.040	579.47644	1.8970

Boc-DL-Phg-L-Ala-O^{t}Bu

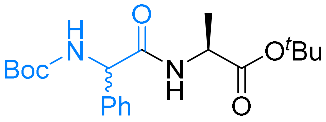

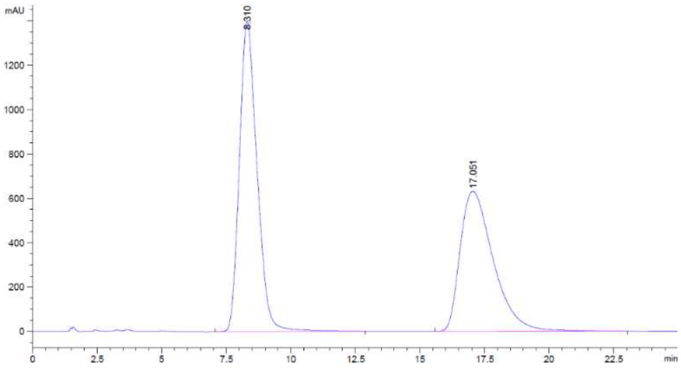

Peak#	RetTime	Area	Area%
1	8.310	6.63083e4	54.0837
2	17.051	5.62948e4	45.9163

8.310 min = Boc-L-Phg-L-Ala-O^{t}Bu; 17.051 min = Boc-D-Phg-L-Ala-O^{t}Bu

Conditions: 2-propanol/hexane = 3:97, v = 2.0 mL/min, λ = 210 nm, temperature = 25 ˚C

Chiral Column: AD-H column from TharSFC

Boc-L-Phg-L-Ala-O^{t}Bu (10p)

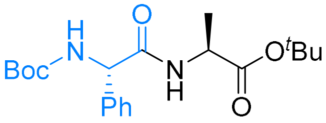

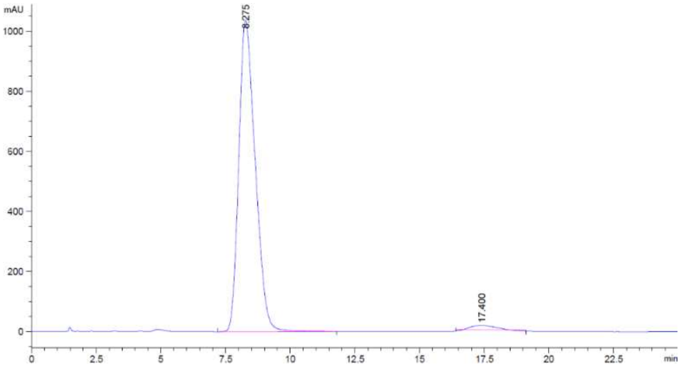

Peak#	RetTime	Area	Area%
1	8.275	4.58750e4	98.0482
2	17.400	913.18896	1.9518

Boc-DL-Ala-L-Ala-OBn

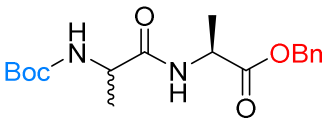

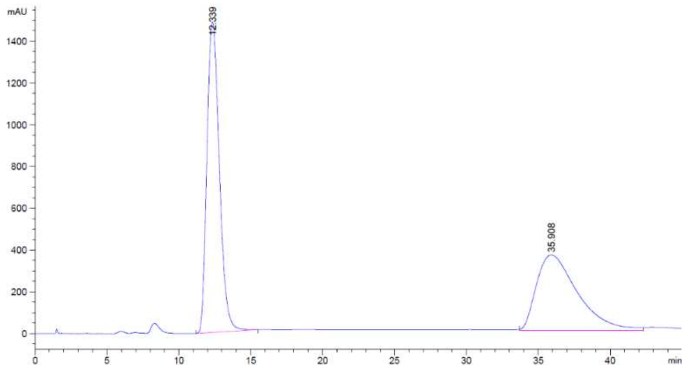

Peak#	RetTime	Area	Area%
1	12.339	8.92919e4	55.5004
2	35.908	7.15934e4	44.4996

12.339 min = Boc-L-Ala-L-Ala-OBn; 35.908 min = Boc-D-Ala-L-Ala-OBn

Conditions: 2-propanol/hexane = 3:97, v = 2.0 mL/min, λ = 210 nm, temperature = 25 ˚C

Chiral Column: AD-H column from TharSFC

Boc-D-Ala-L-Ala-OBn (11a)

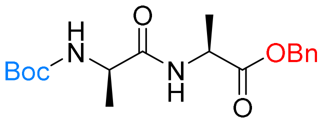

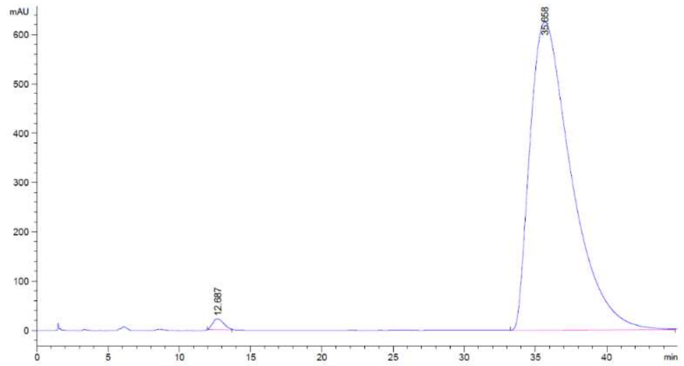

Peak#	RetTime	Area	Area%
1	12.687	1179.24146	0.9259
2	35.658	1.26176e5	99.0741

9a $^{19}F\{^1H\}$ NMR (282 MHz, Chloroform-*d*)

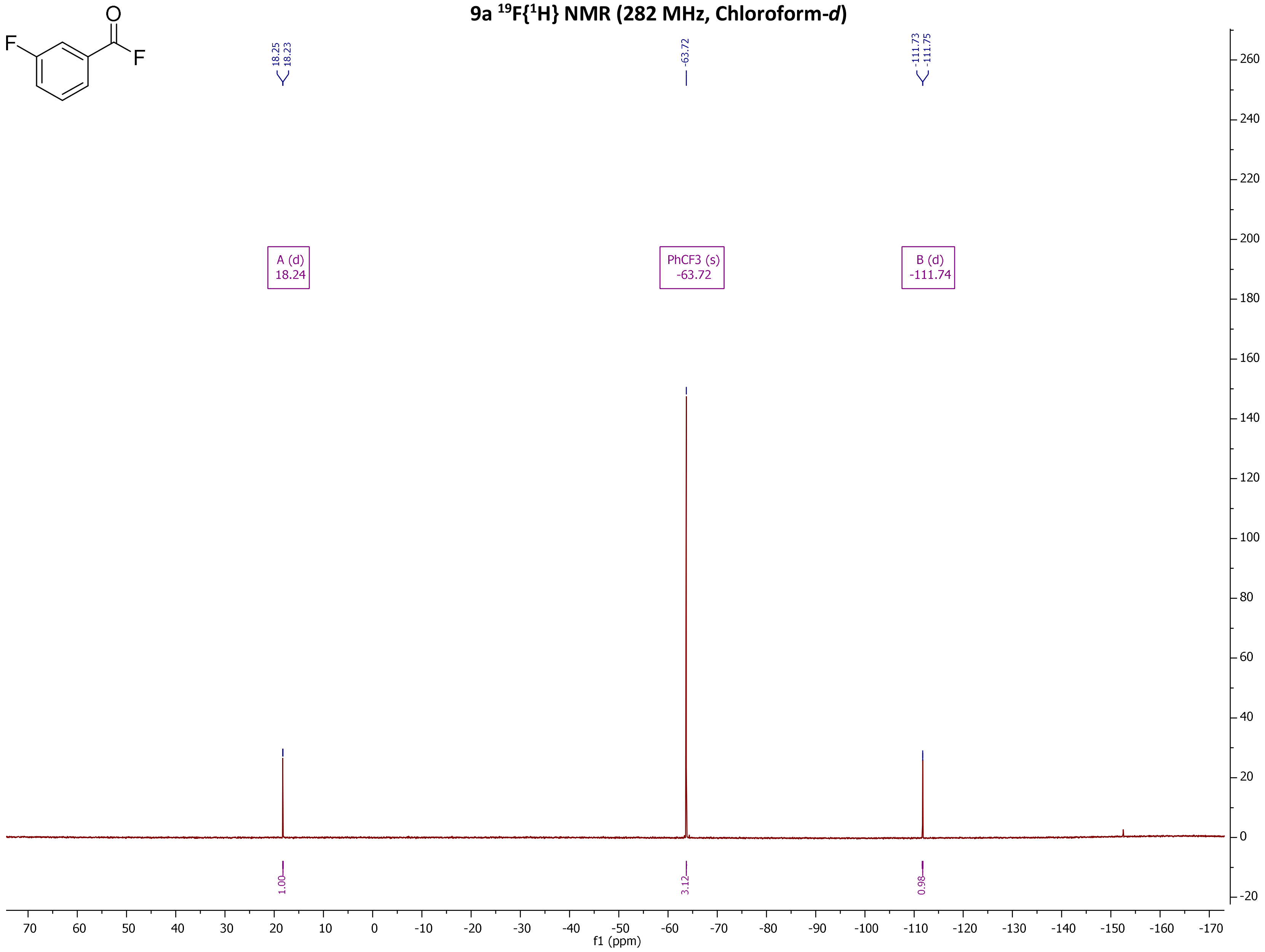

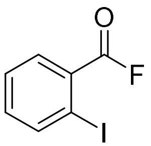

9b ^{1}H NMR (300 MHz, Chloroform-*d*)

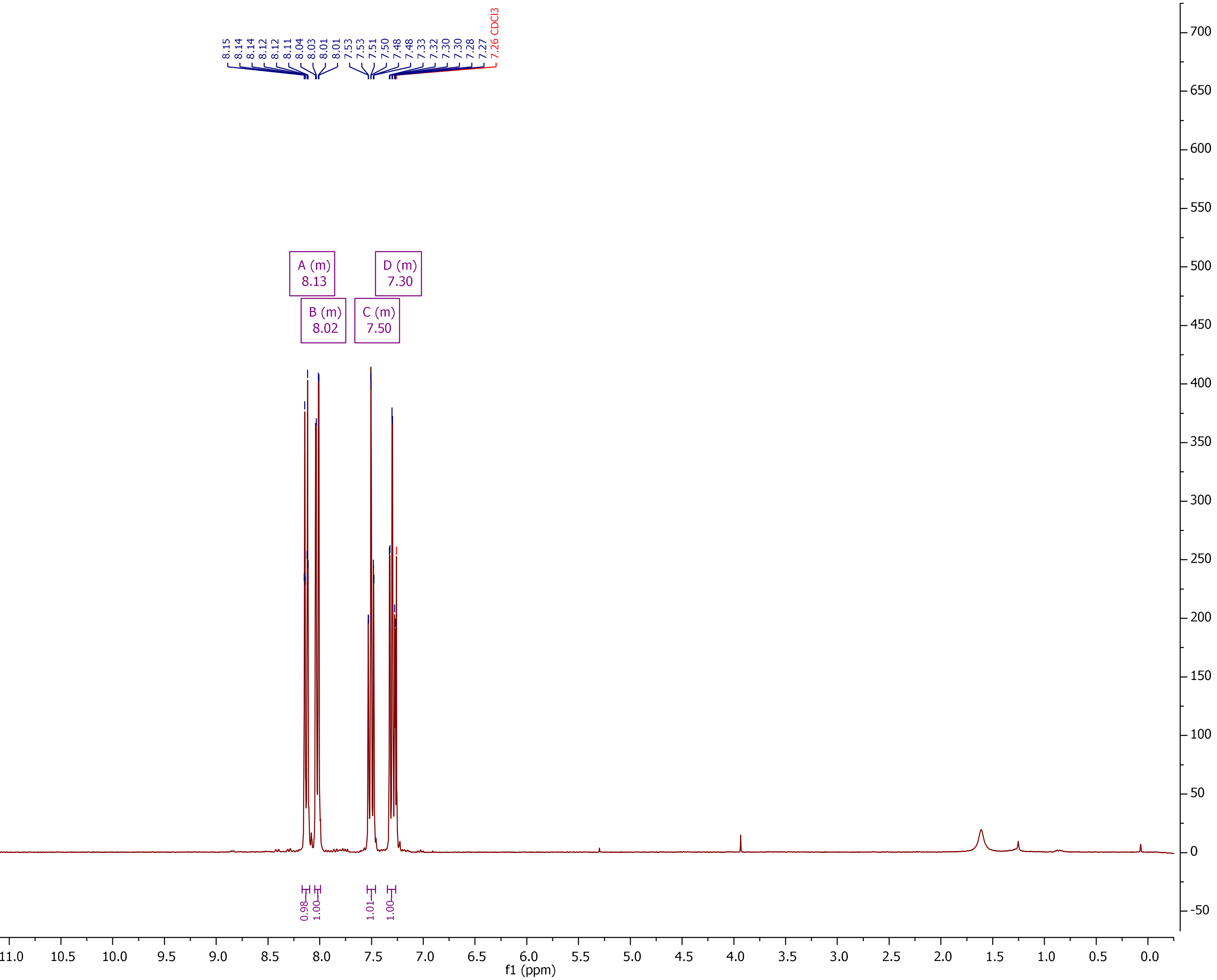

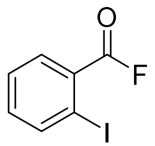

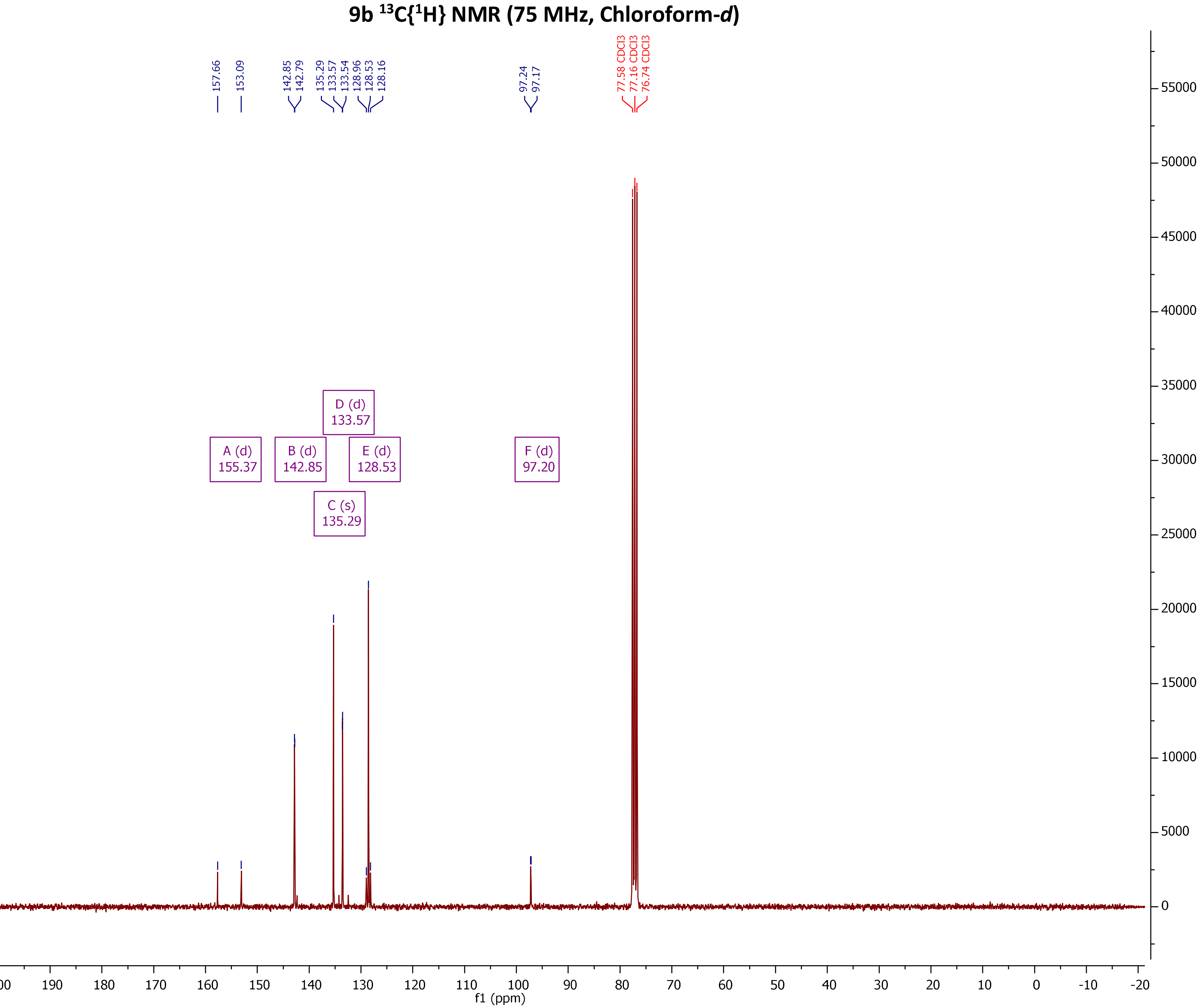
9b $^{13}C\{^{1}H\}$ NMR (75 MHz, Chloroform-*d*)
157.66
153.09
142.85
142.79
135.29
133.57
133.54
128.96
128.53
128.16
97.24
97.17
77.58 CDCl3
77.16 CDCl3
76.74 CDCl3
A (d) 155.37
B (d) 142.85
C (s) 135.29
D (d) 133.57
E (d) 128.53
F (d) 97.20
f1 (ppm)

9b $^{19}F\{^1H\}$ NMR (282 MHz, Chloroform-*d*)

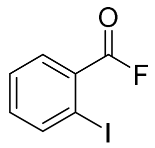

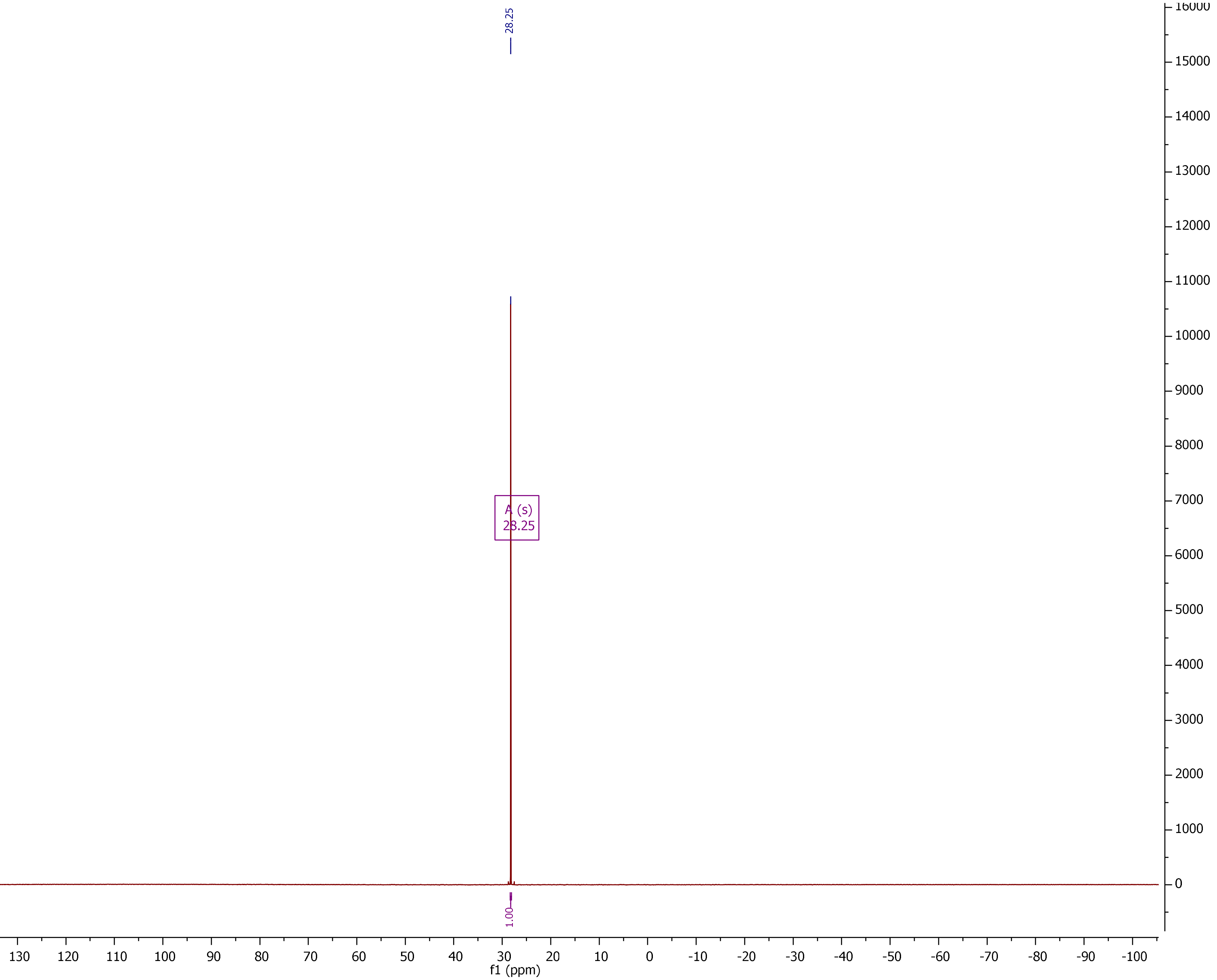

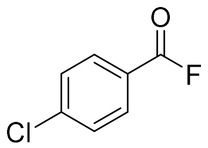

9c $^{19}F\{^{1}H\}$ NMR (282 MHz, Chloroform-*d*)

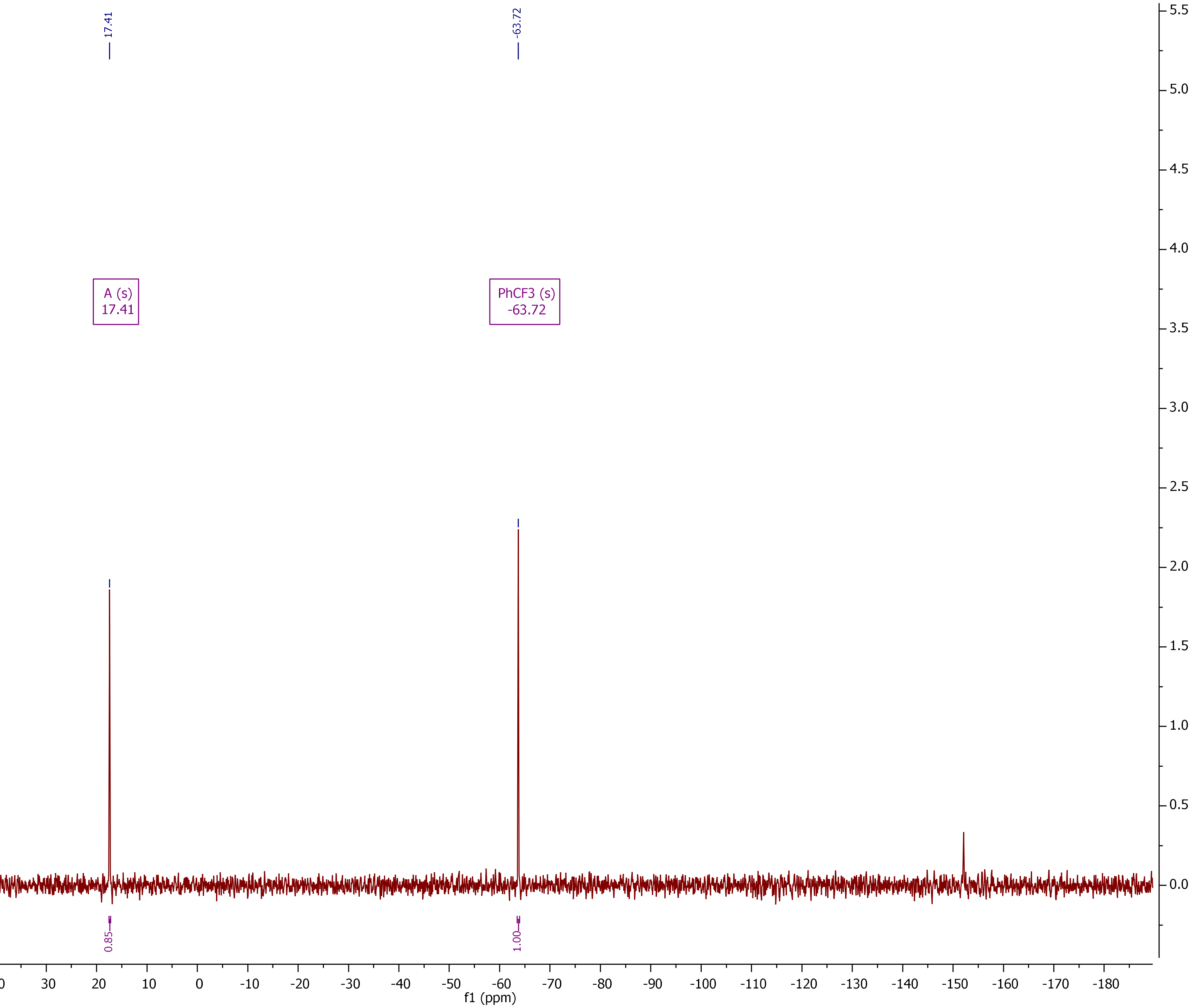

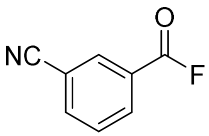

9d ^{1}H NMR (300 MHz, Chloroform-*d*)

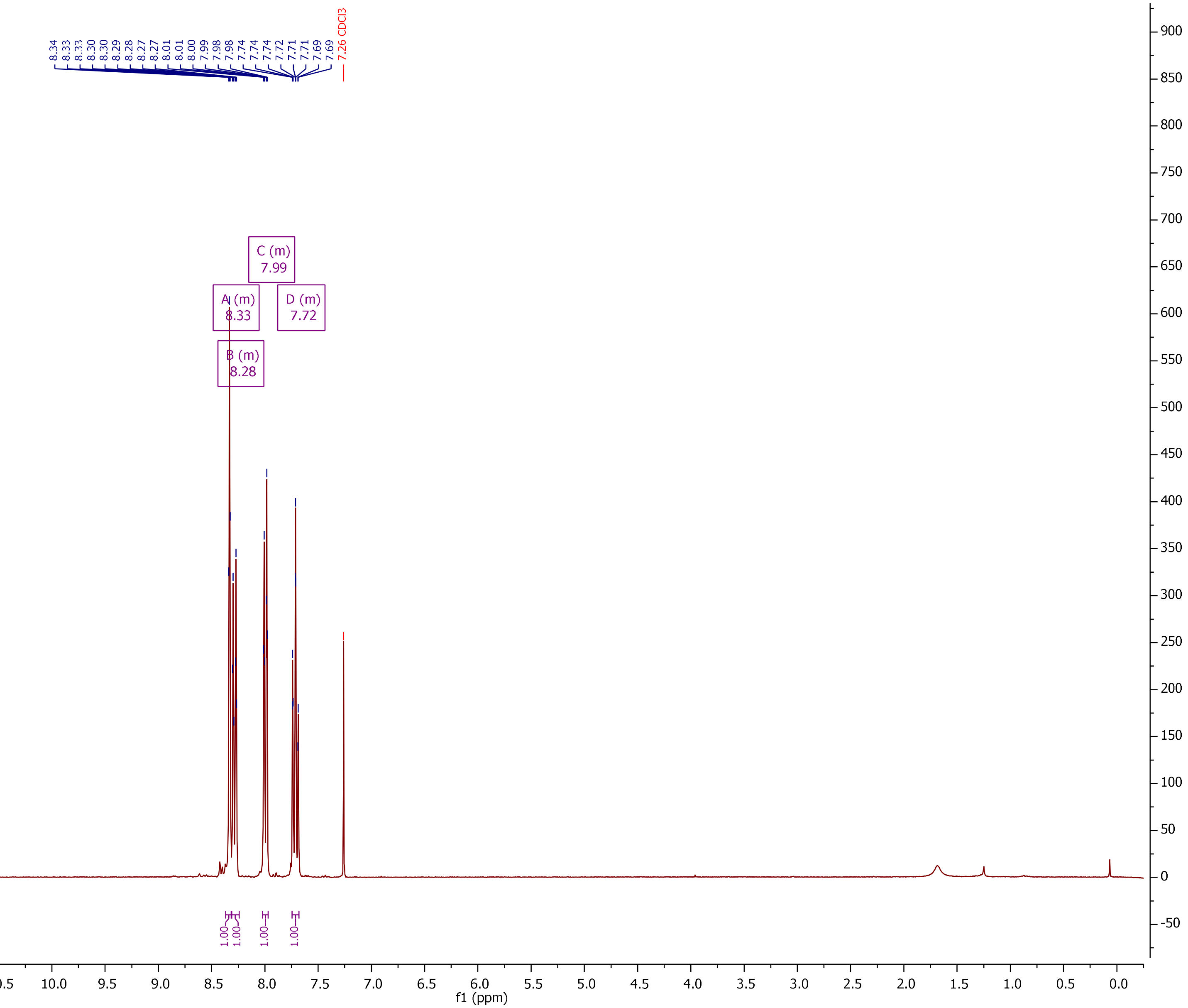

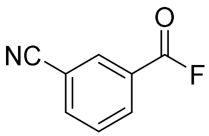

9d $^{13}C\{^{1}H\}$ NMR (75 MHz, Chloroform-*d*)

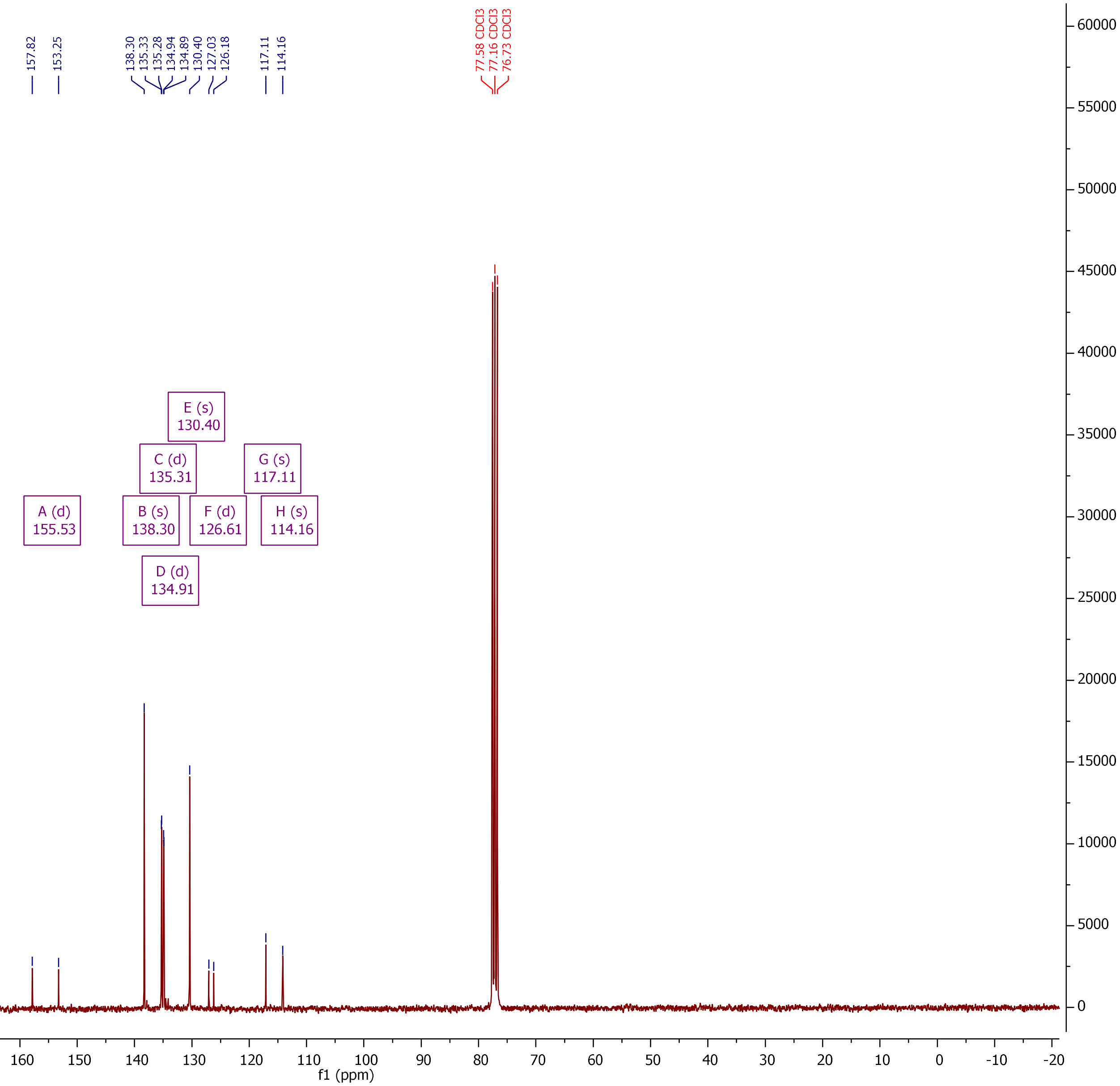

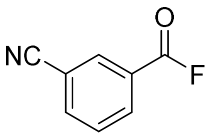

9d $^{19}F\{^1H\}$ NMR (282 MHz, Chloroform-*d*)

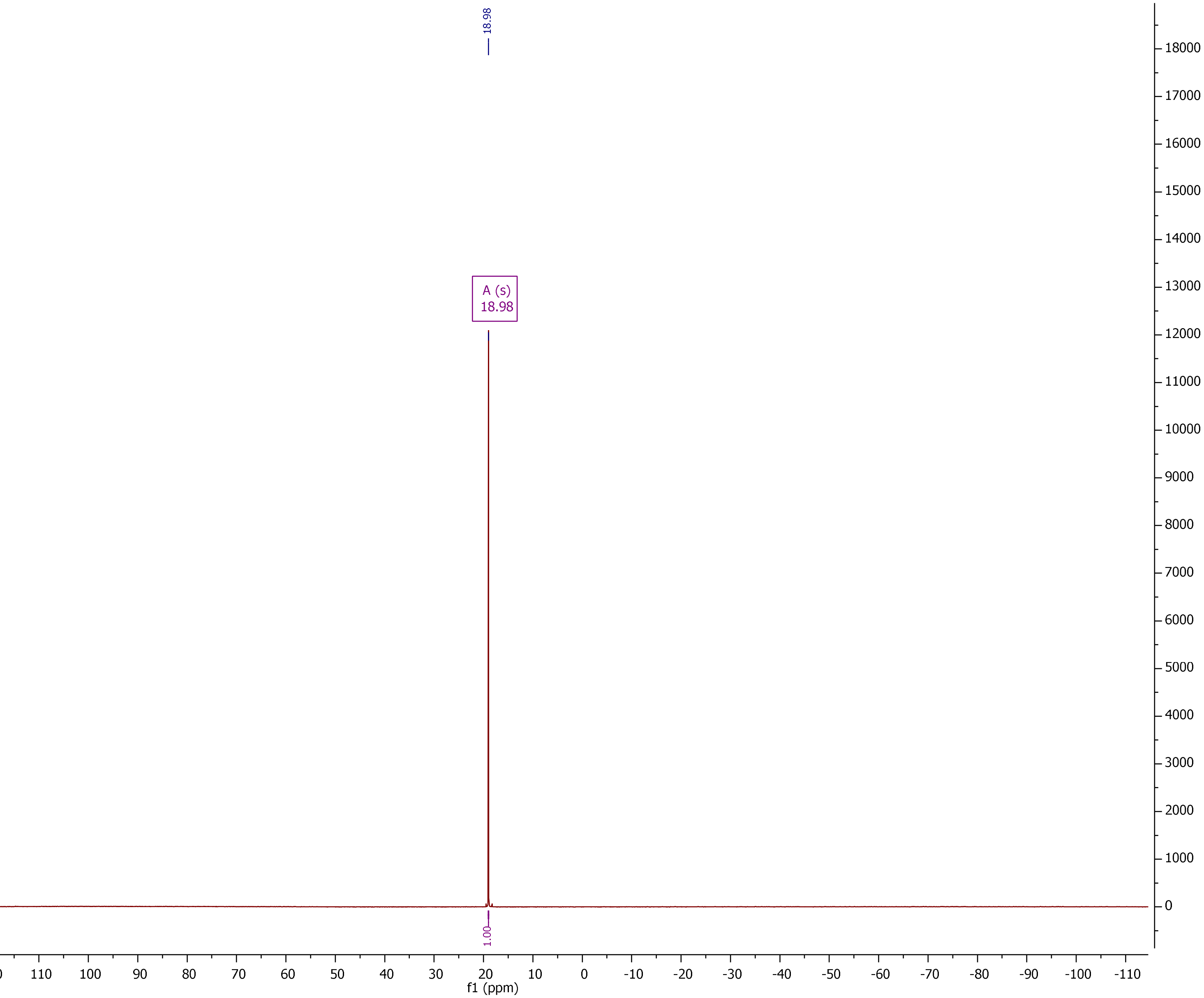

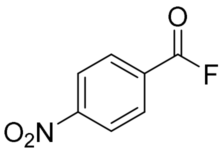

9e ^{1}H NMR (300 MHz, Chloroform-*d*)

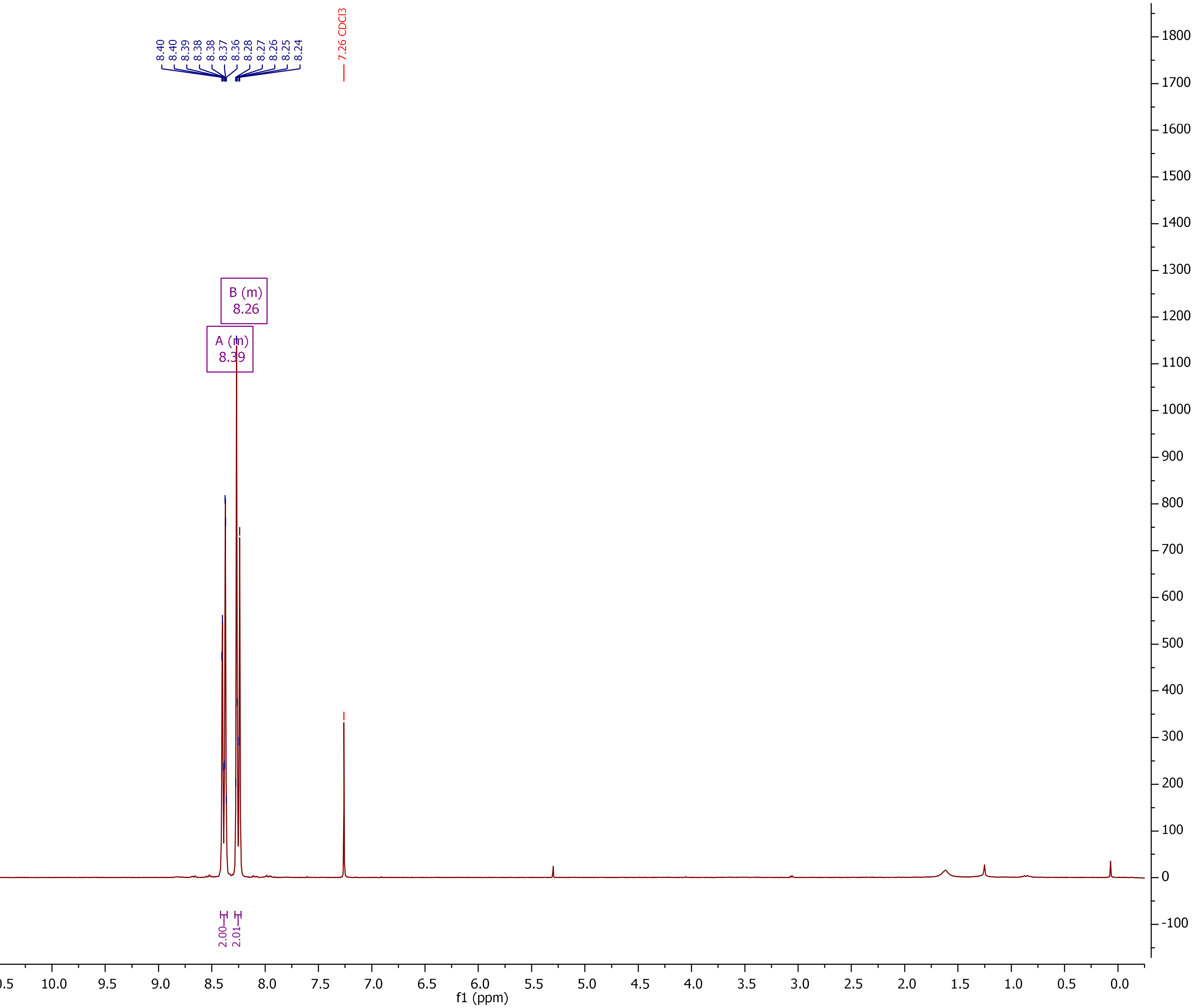

9e $^{13}C\{^1H\}$ NMR (75 MHz, Chloroform-*d*)

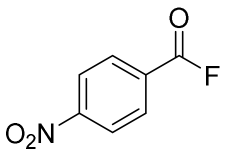

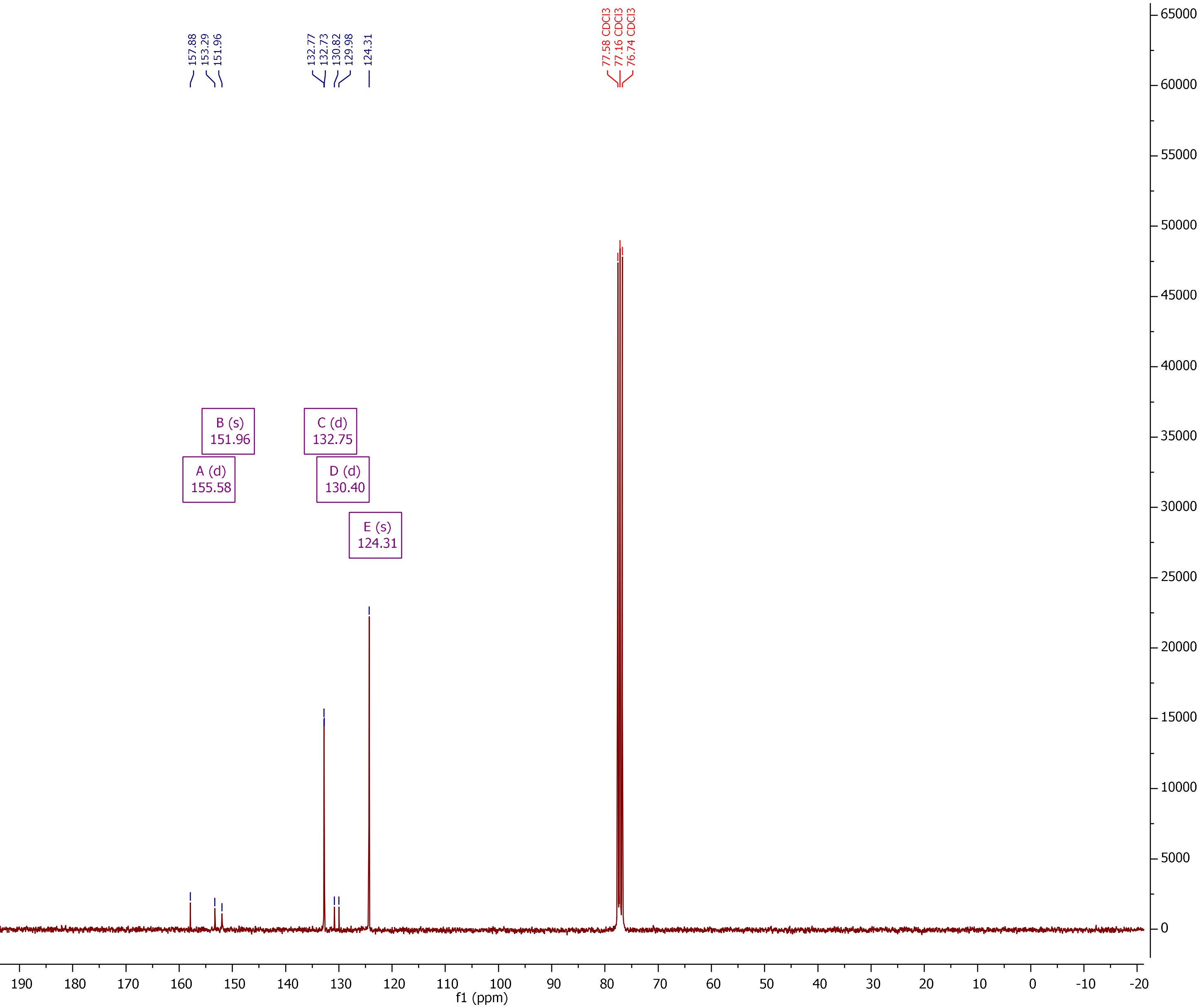

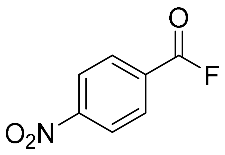

9e $^{19}F\{^{1}H\}$ NMR (282 MHz, Chloroform-*d*)

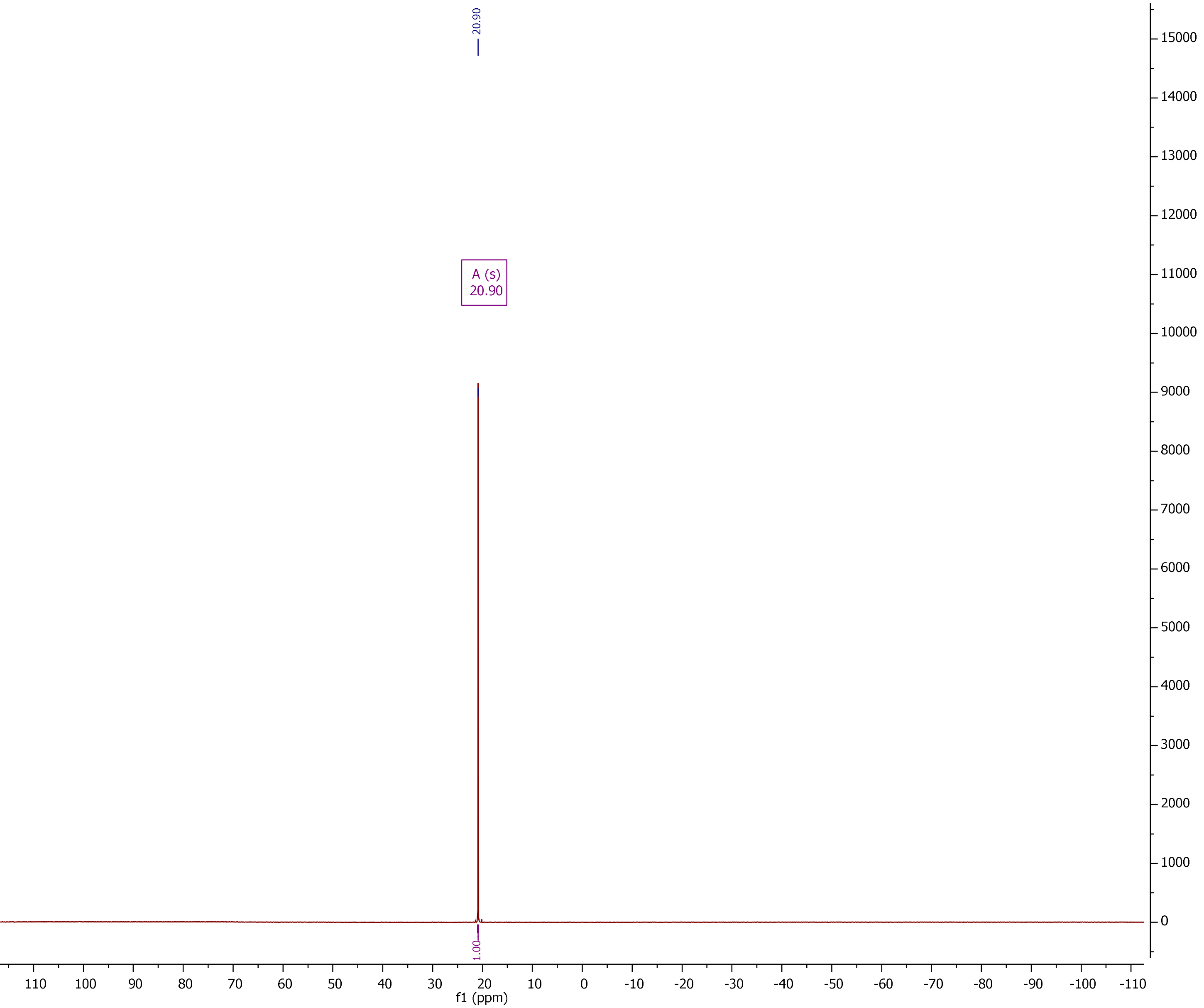

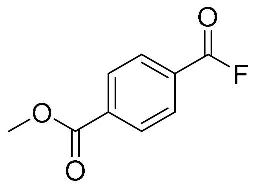

9f ^{1}H NMR (300 MHz, Chloroform-*d*)

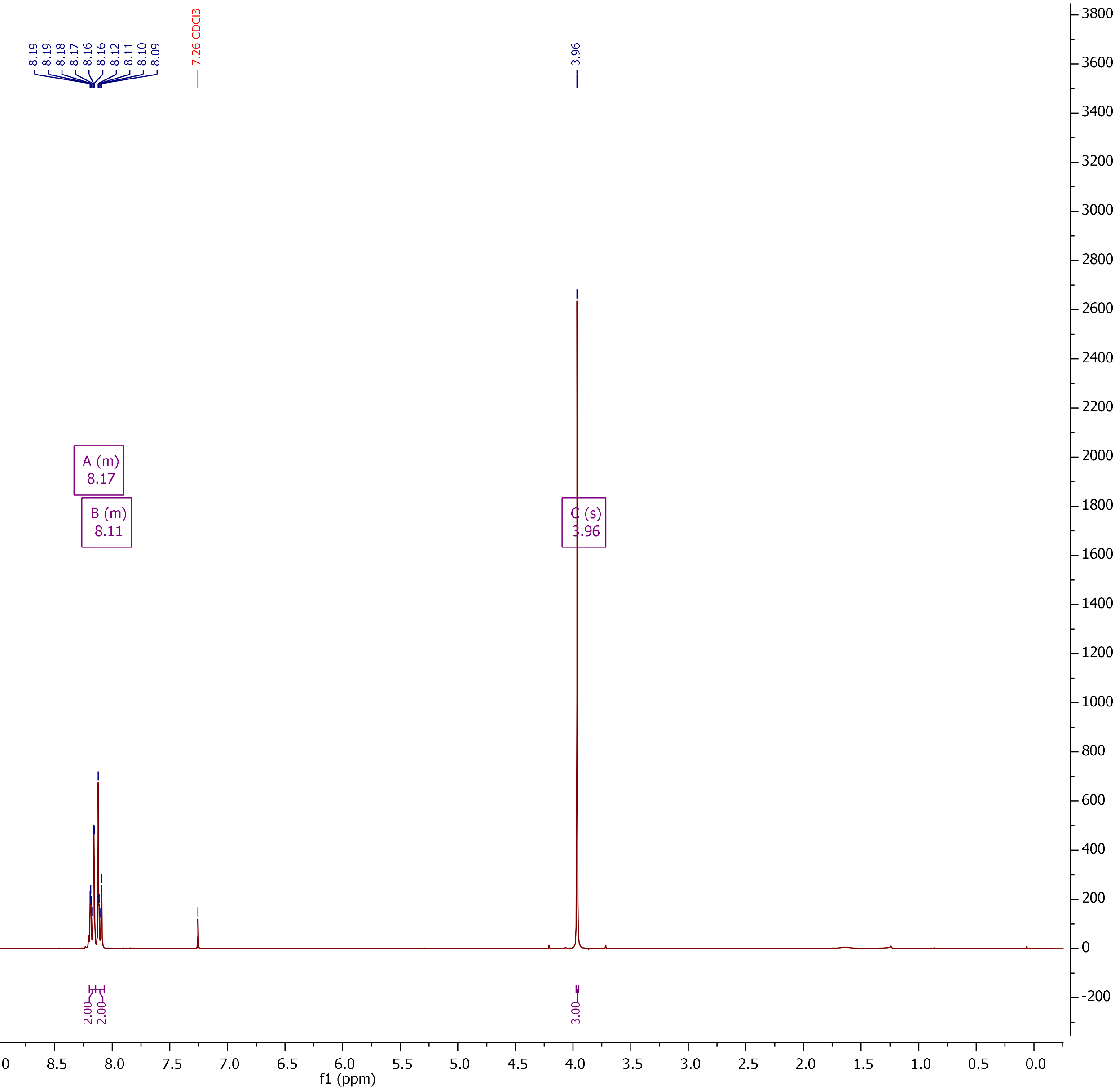

9f $^{13}C\{^{1}H\}$ NMR (75 MHz, Chloroform-*d*)

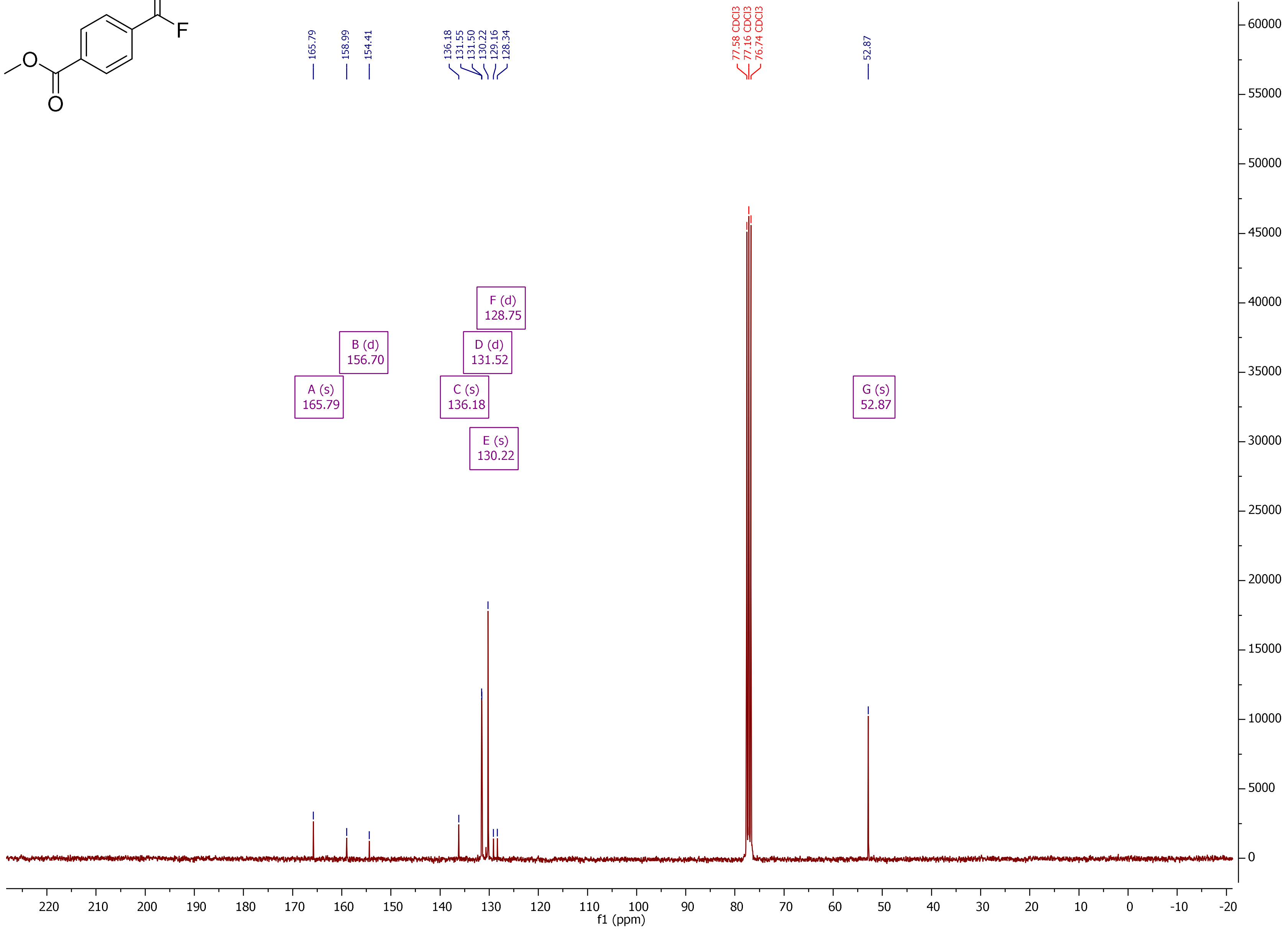

9f $^{19}F\{^{1}H\}$ NMR (282 MHz, Chloroform-*d*)

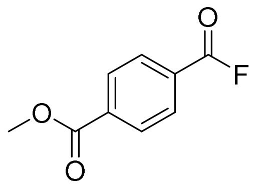

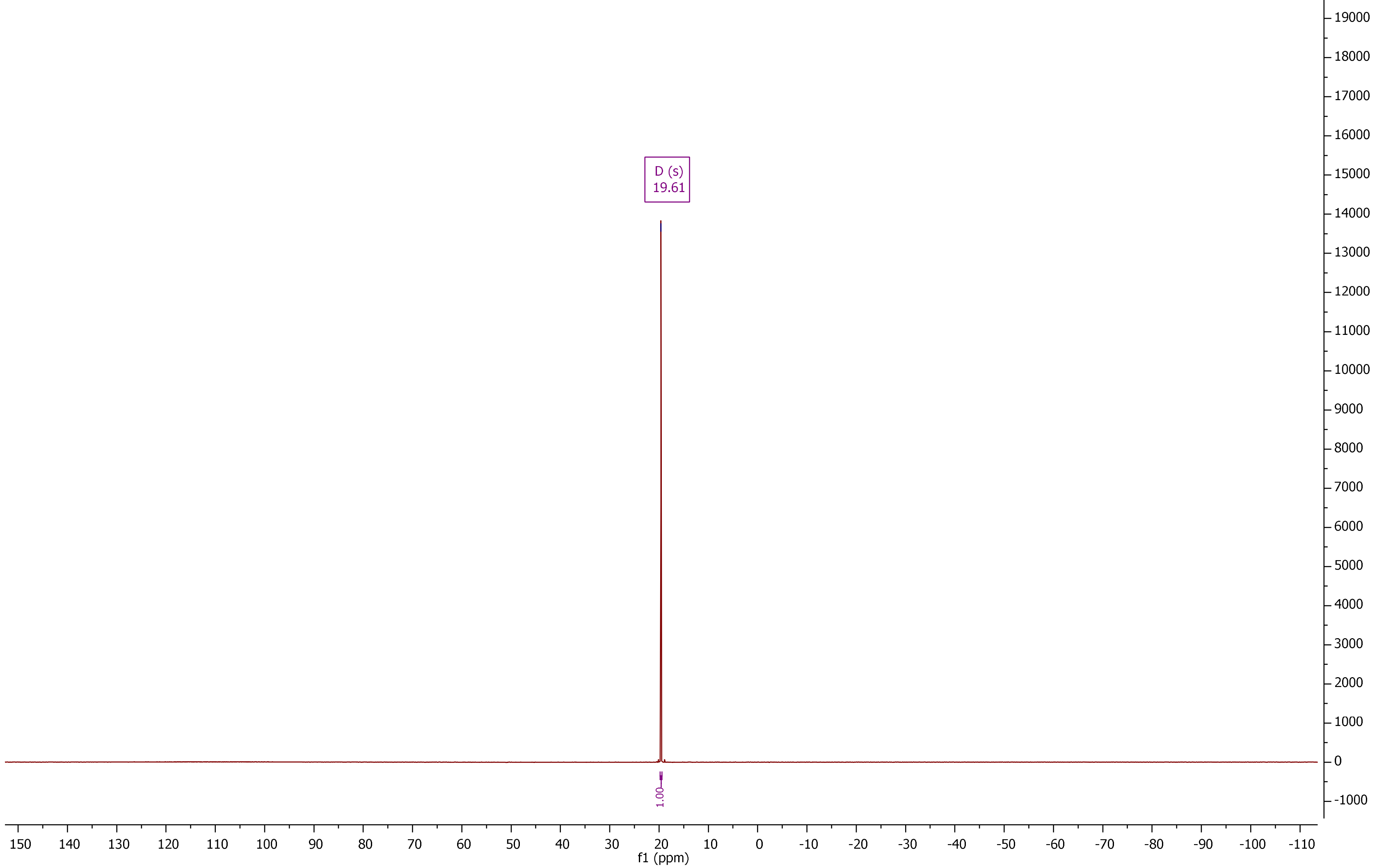

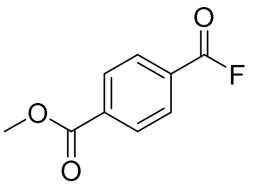

9g $^{19}F\{^1H\}$ NMR (282 MHz, Chloroform-*d*)

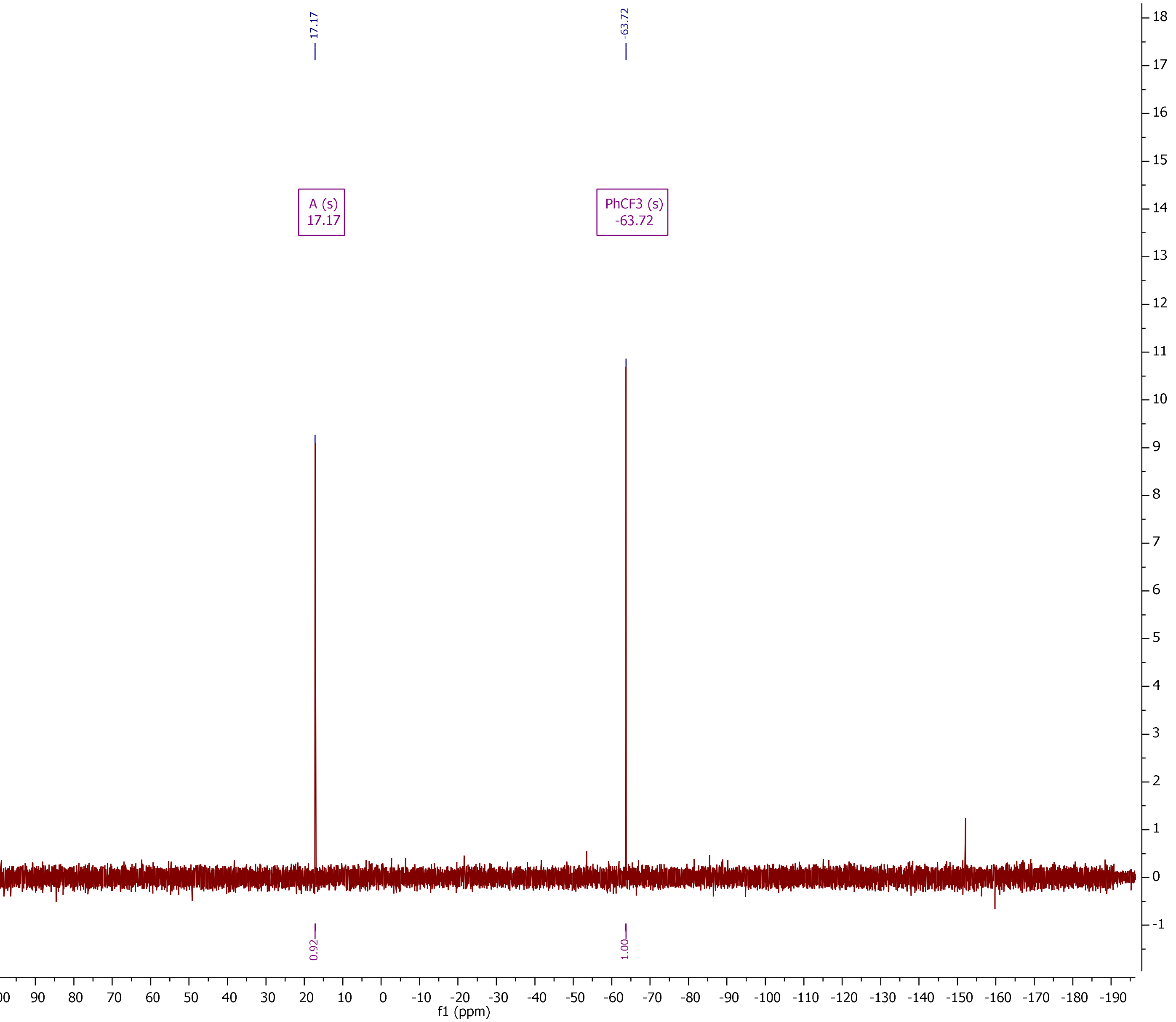

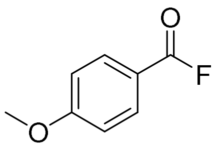

9h $^{19}F\{^1H\}$ NMR (282 MHz, Chloroform-*d*)

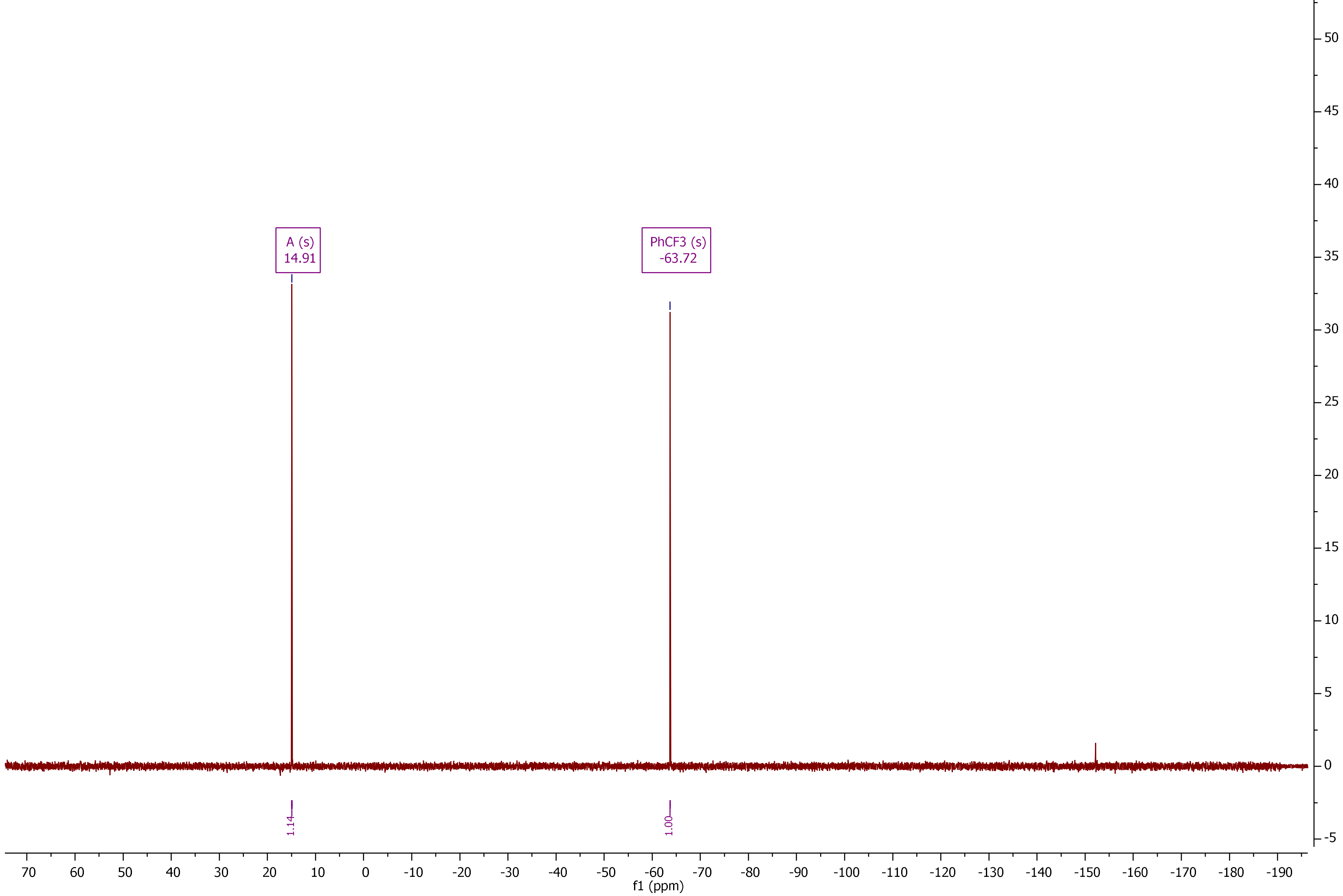

9i $^{19}F\{^1H\}$ NMR (282 MHz, Chloroform-*d*)

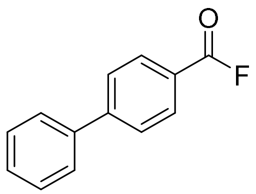

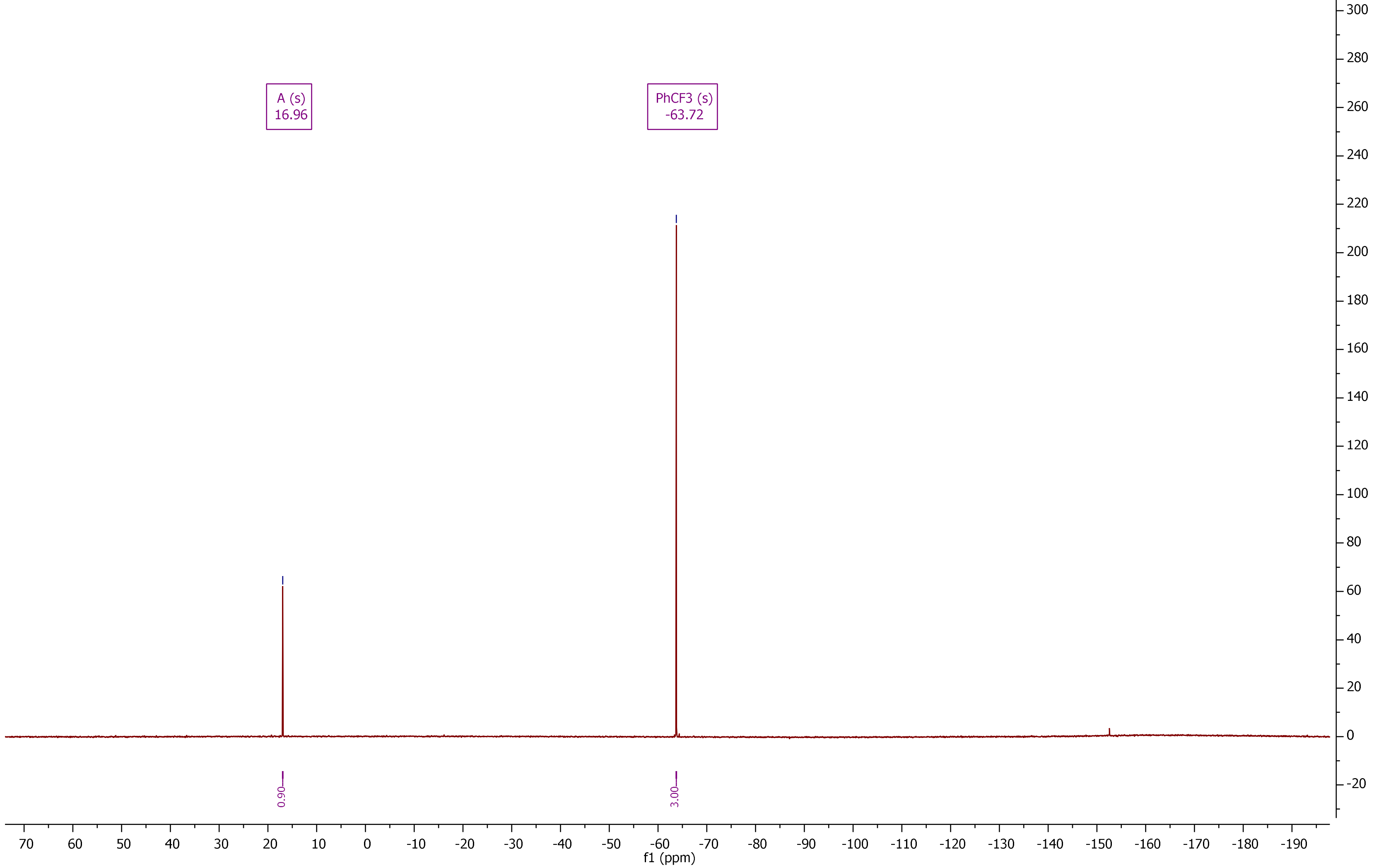

9j $^{19}F\{^{1}H\}$ NMR (282 MHz, Chloroform-*d*)

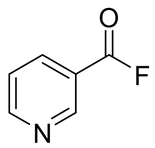

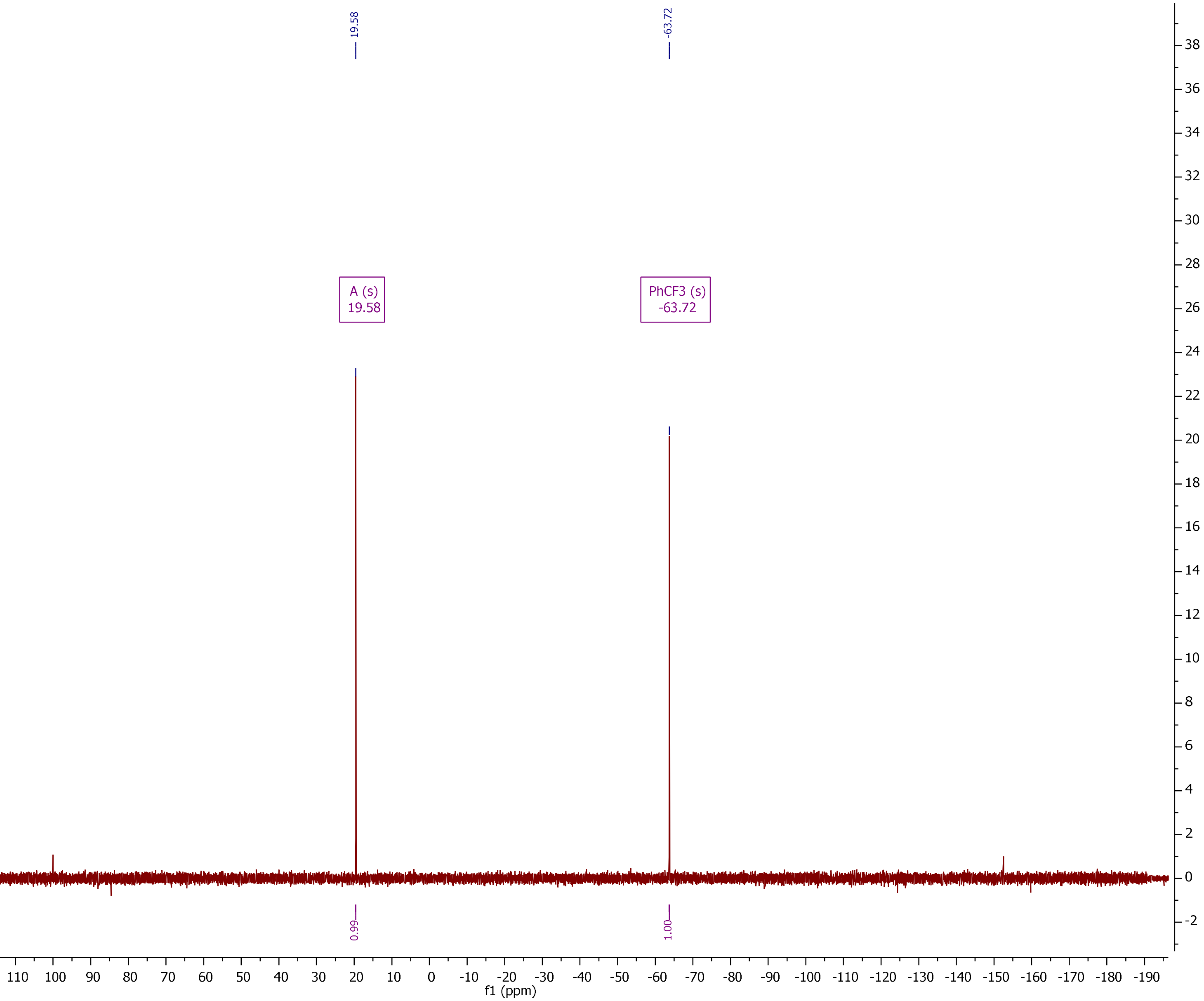

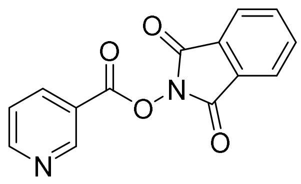

9j’ ^{1}H NMR (300 MHz, Chloroform-*d*)

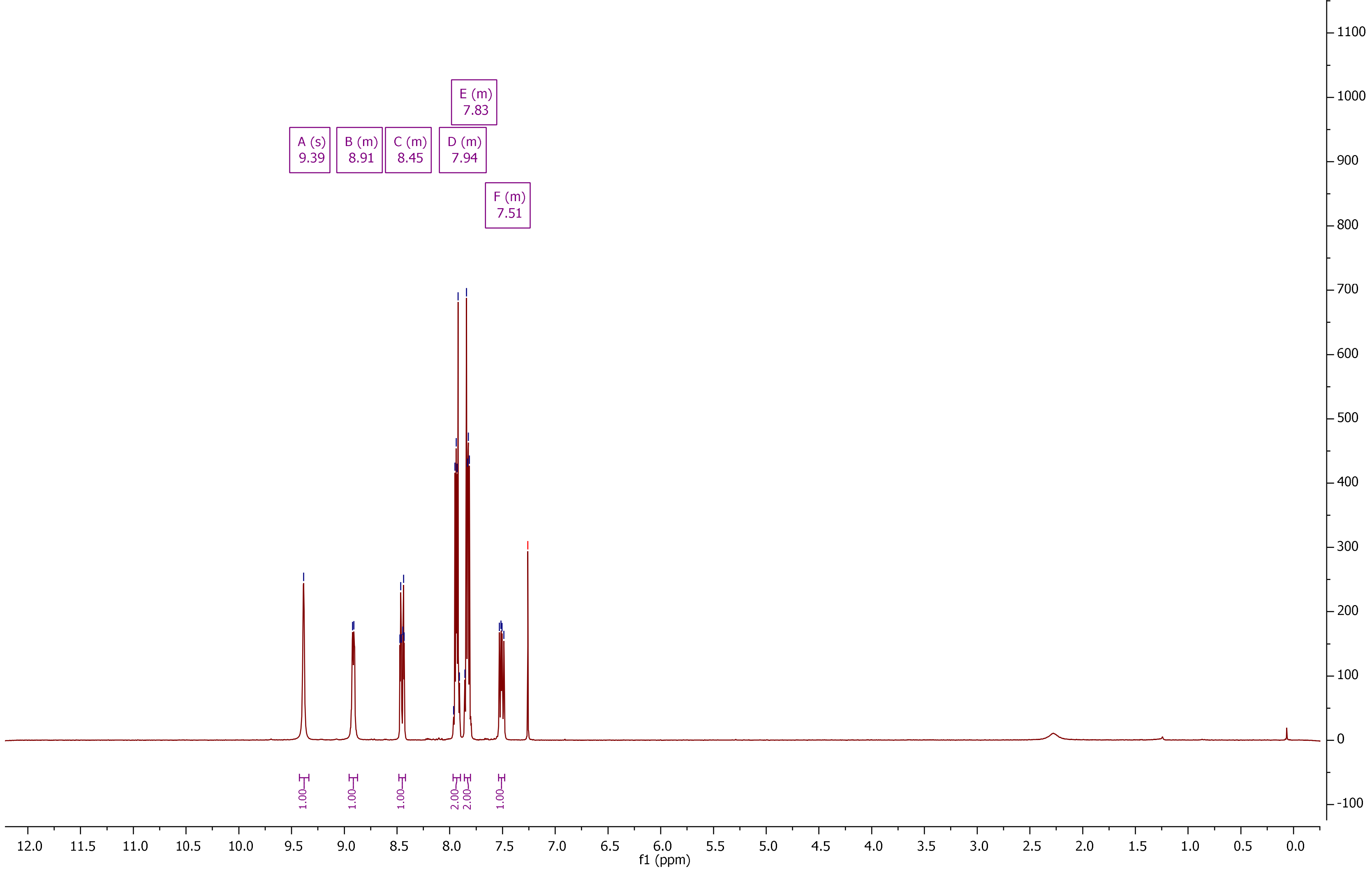

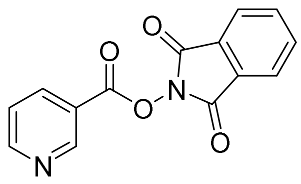

9j' $^{13}C\{^{1}H\}$ NMR (75 MHz, Chloroform-*d*)

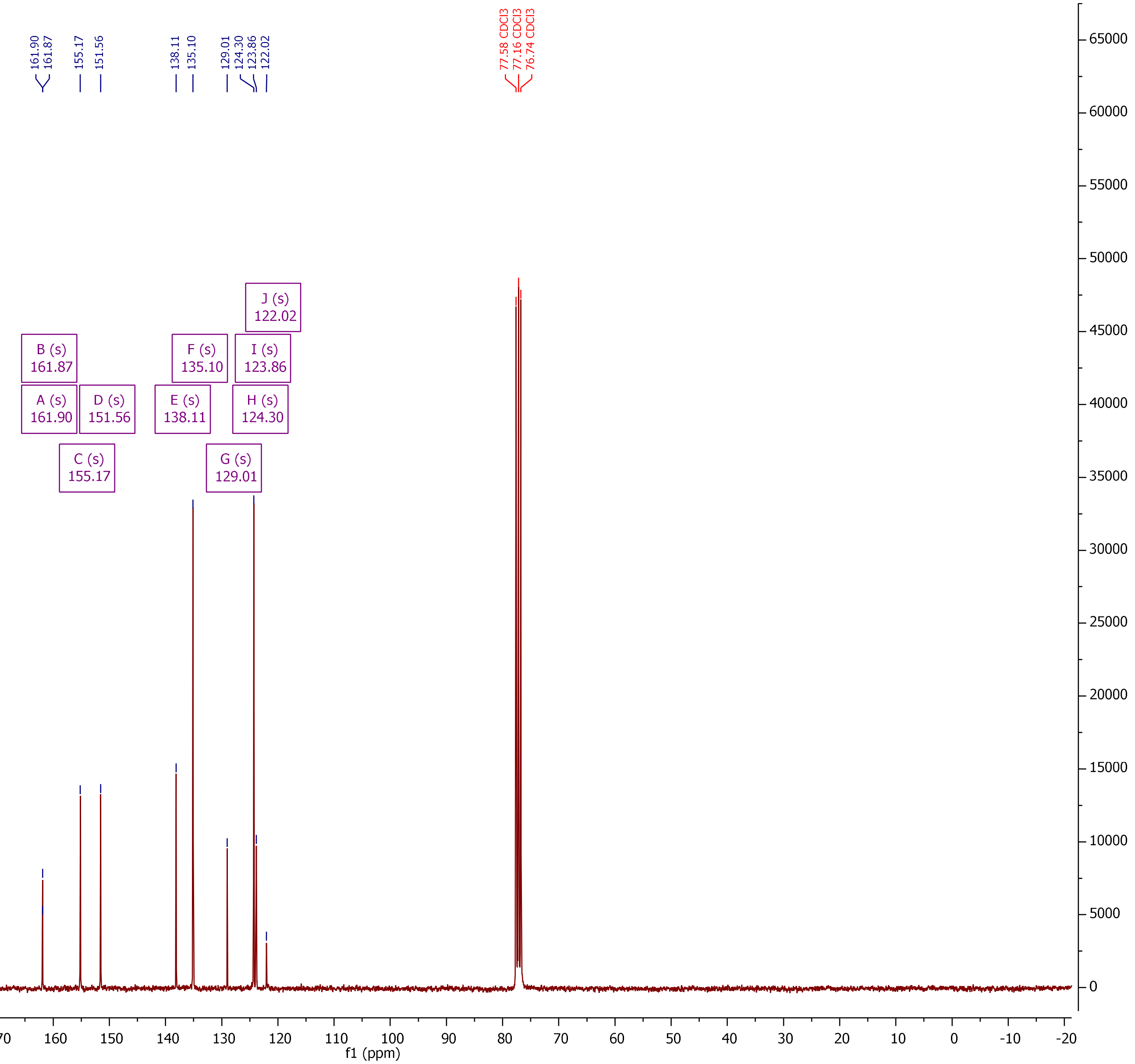

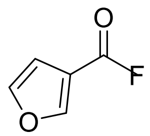

9k $^{19}F\{^1H\}$ NMR (282 MHz, Chloroform-*d*)

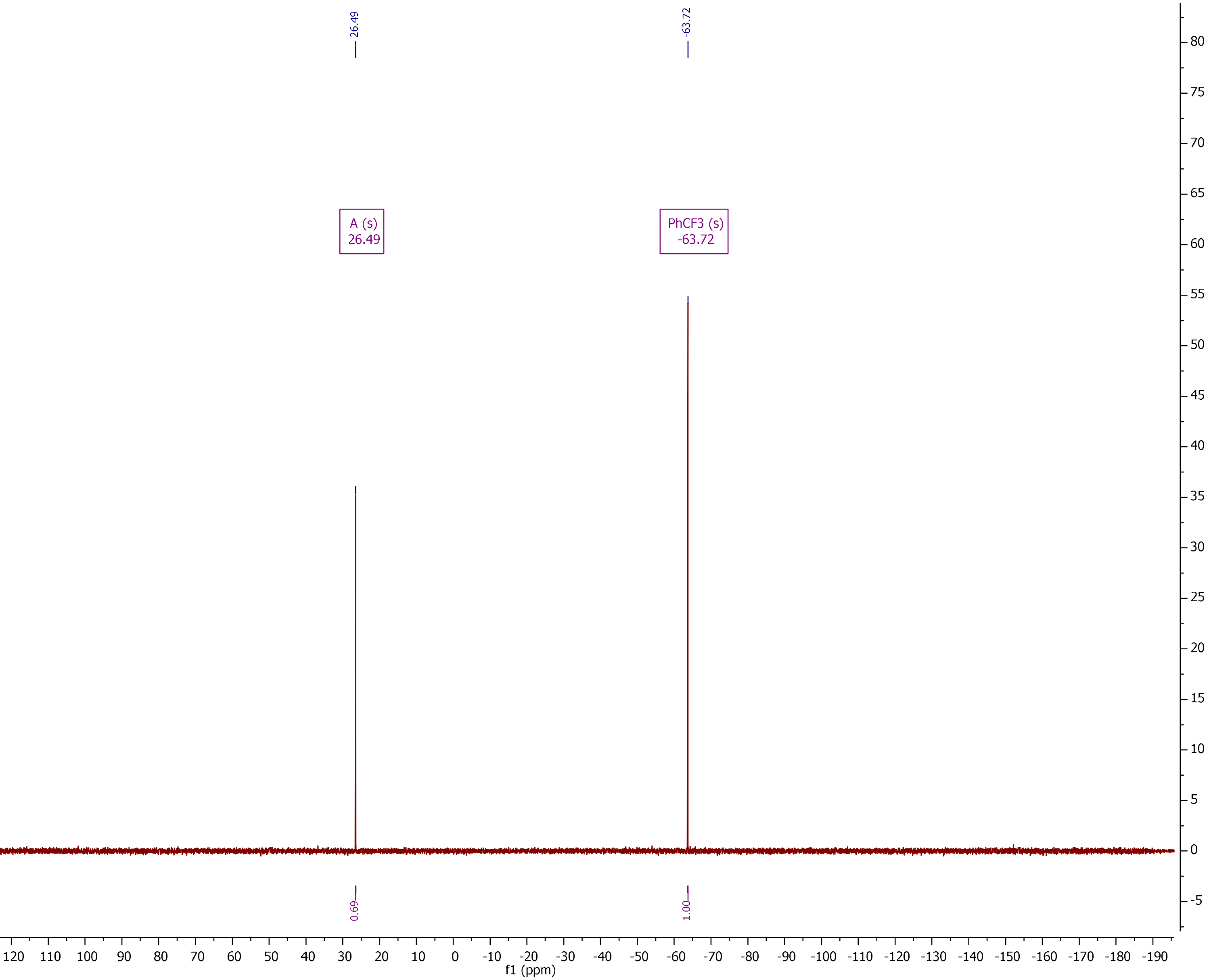

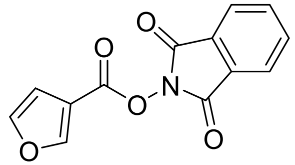

9k' ¹H NMR (300 MHz, Chloroform-*d*)

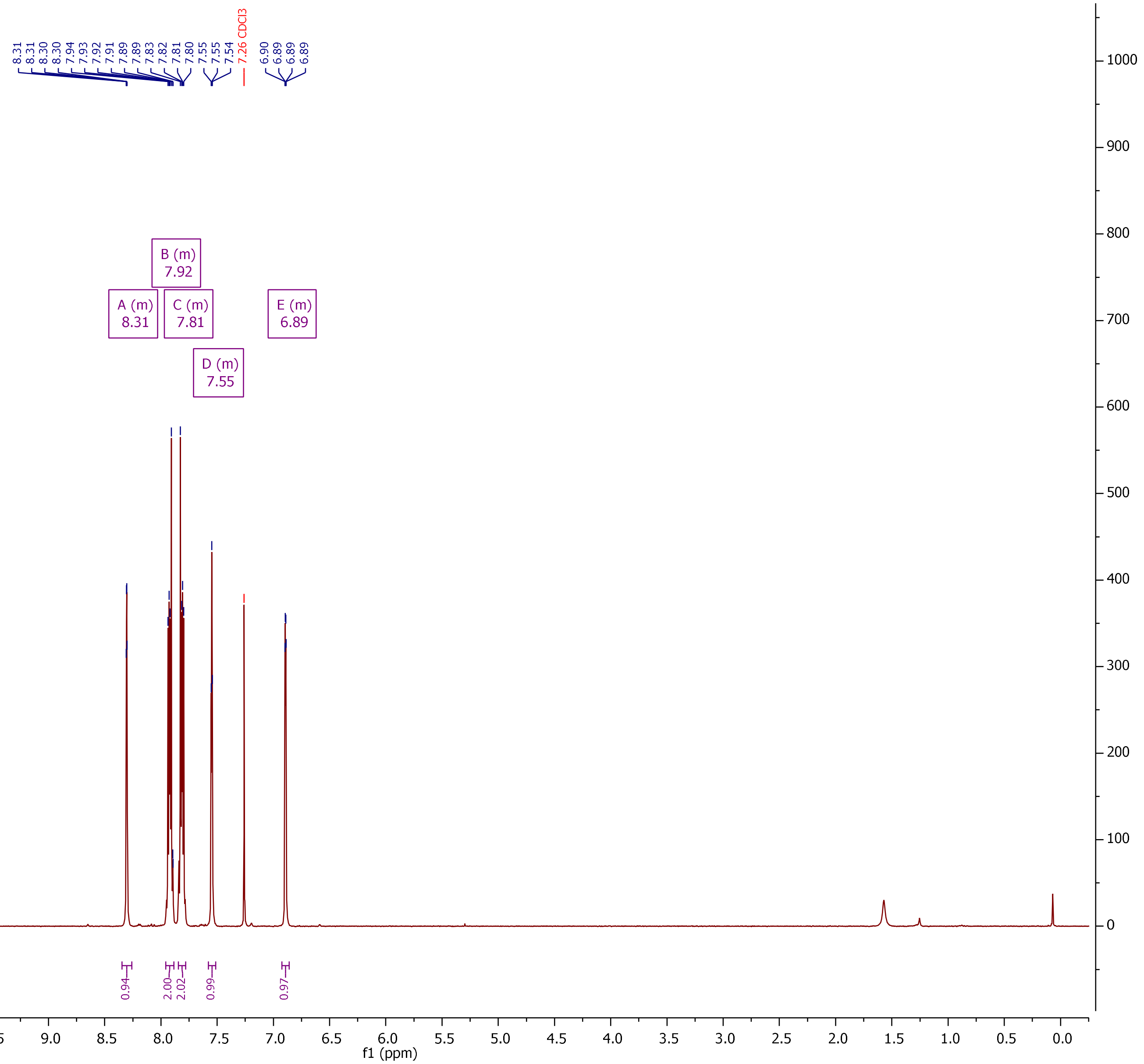

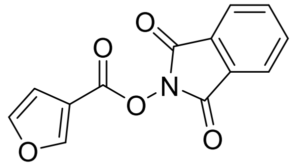

9k' $^{13}C\{^{1}H\}$ NMR (75 MHz, Chloroform-*d*)

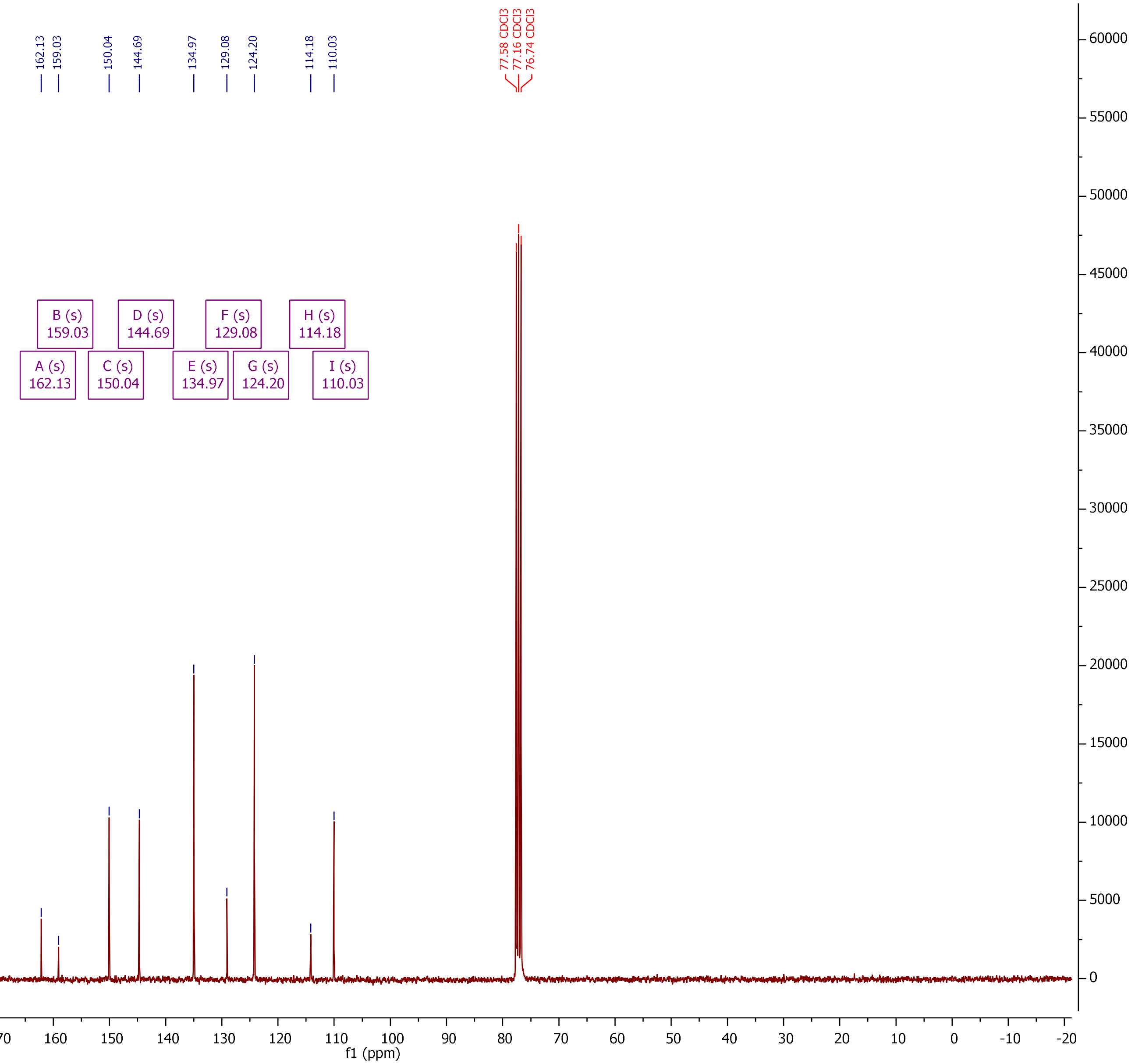

9l $^{19}F\{^{1}H\}$ NMR (282 MHz, Chloroform-*d*)

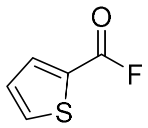

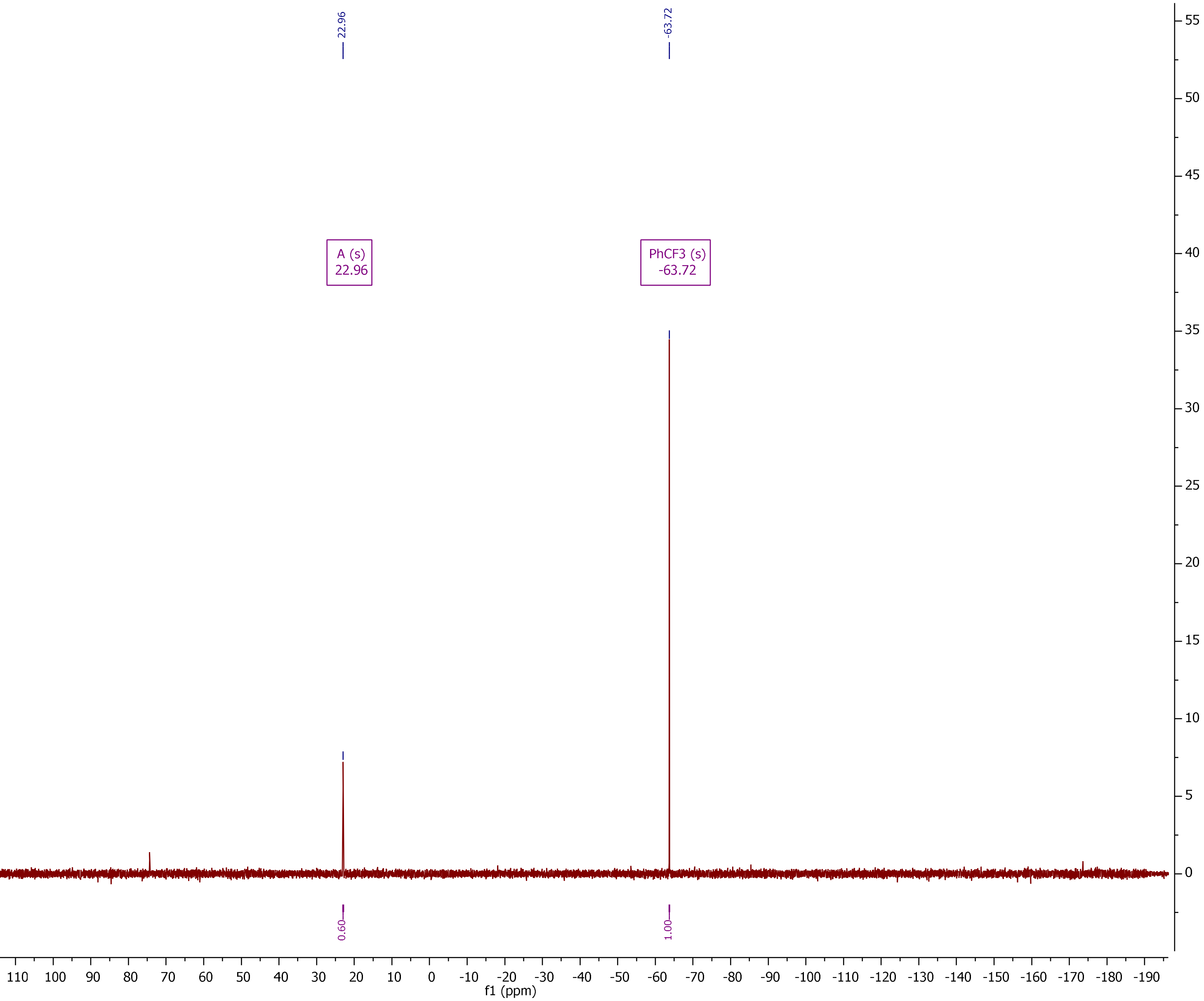

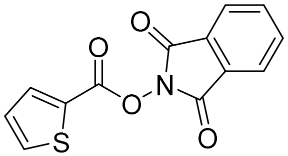

9l’ ^{1}H NMR (300 MHz, Chloroform-*d*)

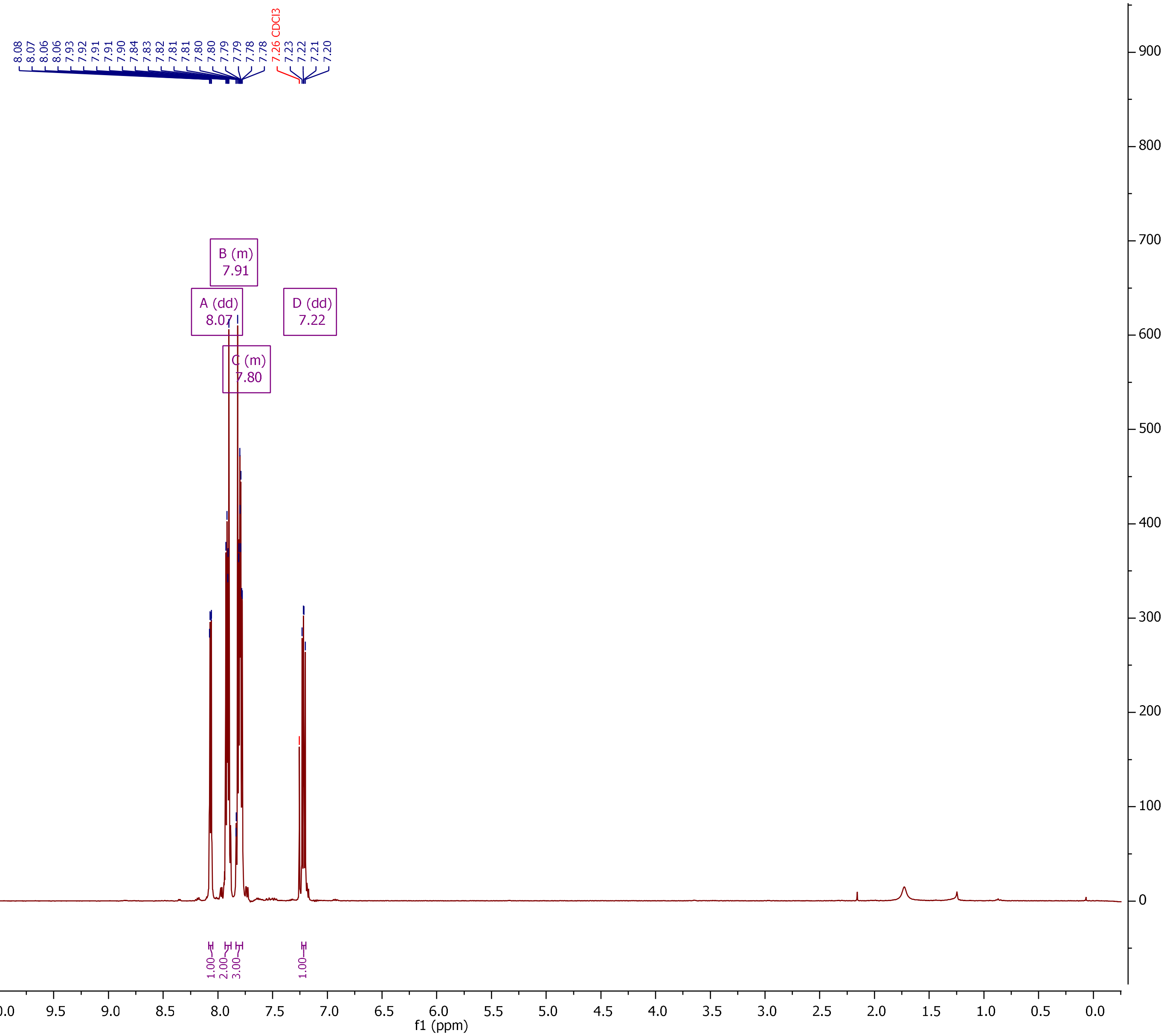

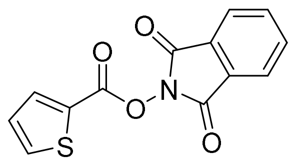

9l’ $^{13}C\{^1H\}$ NMR (75 MHz, Chloroform-*d*)

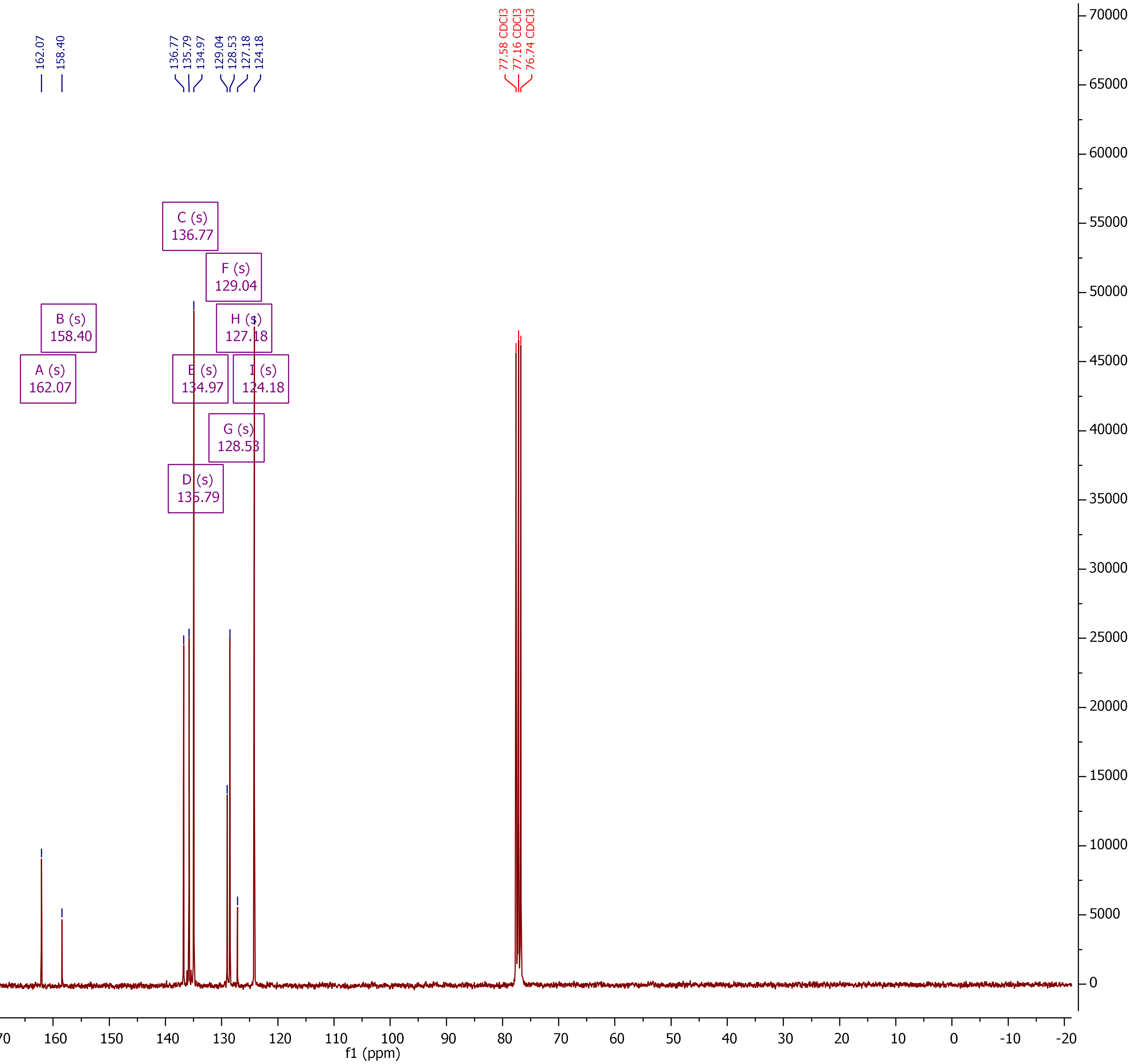

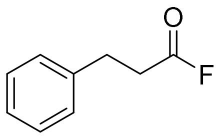

9m ¹H NMR (300 MHz, Chloroform-*d*)

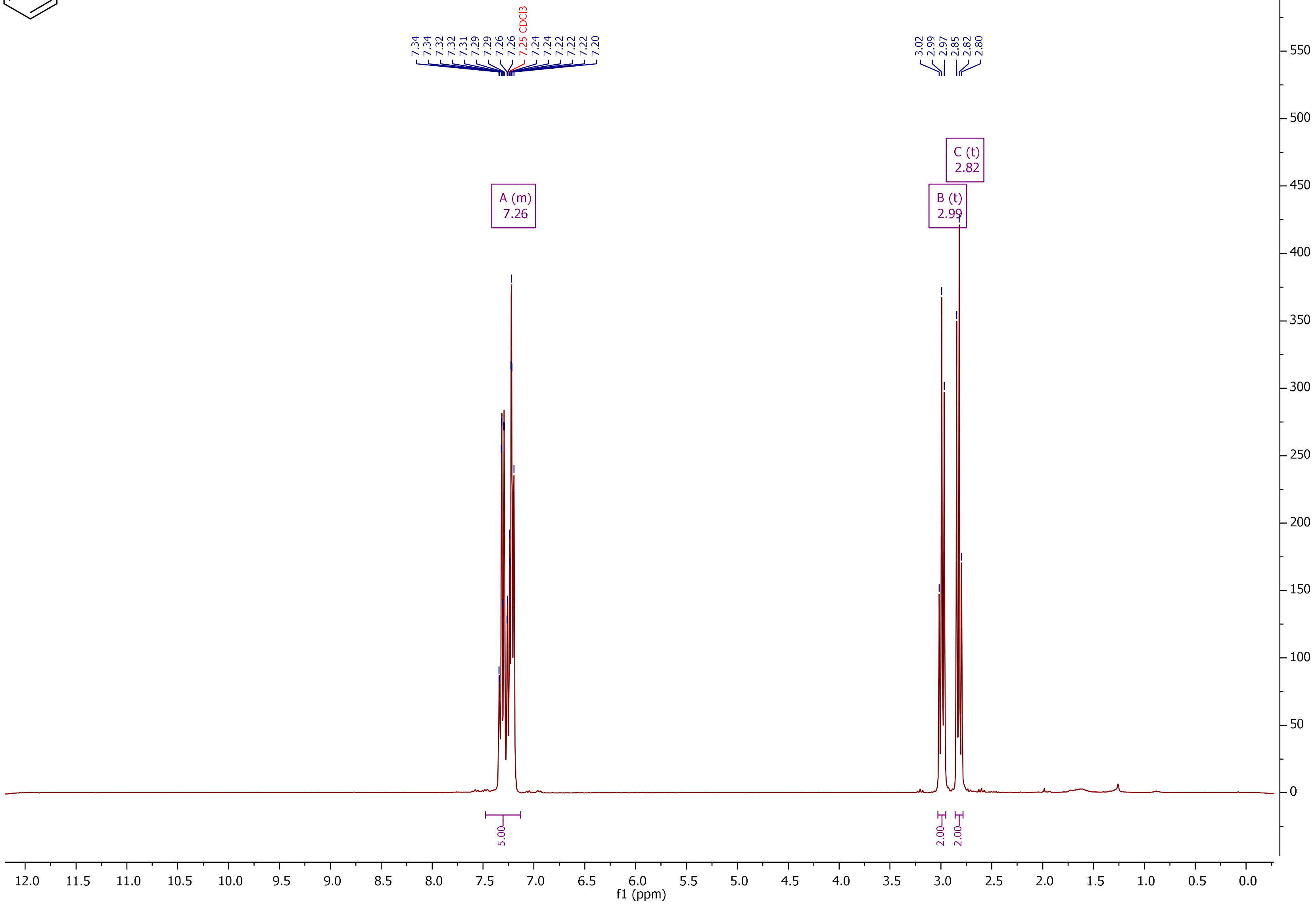

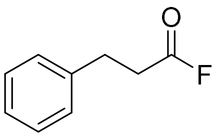

9m $^{13}C\{^1H\}$ NMR (75 MHz, Chloroform-*d*)

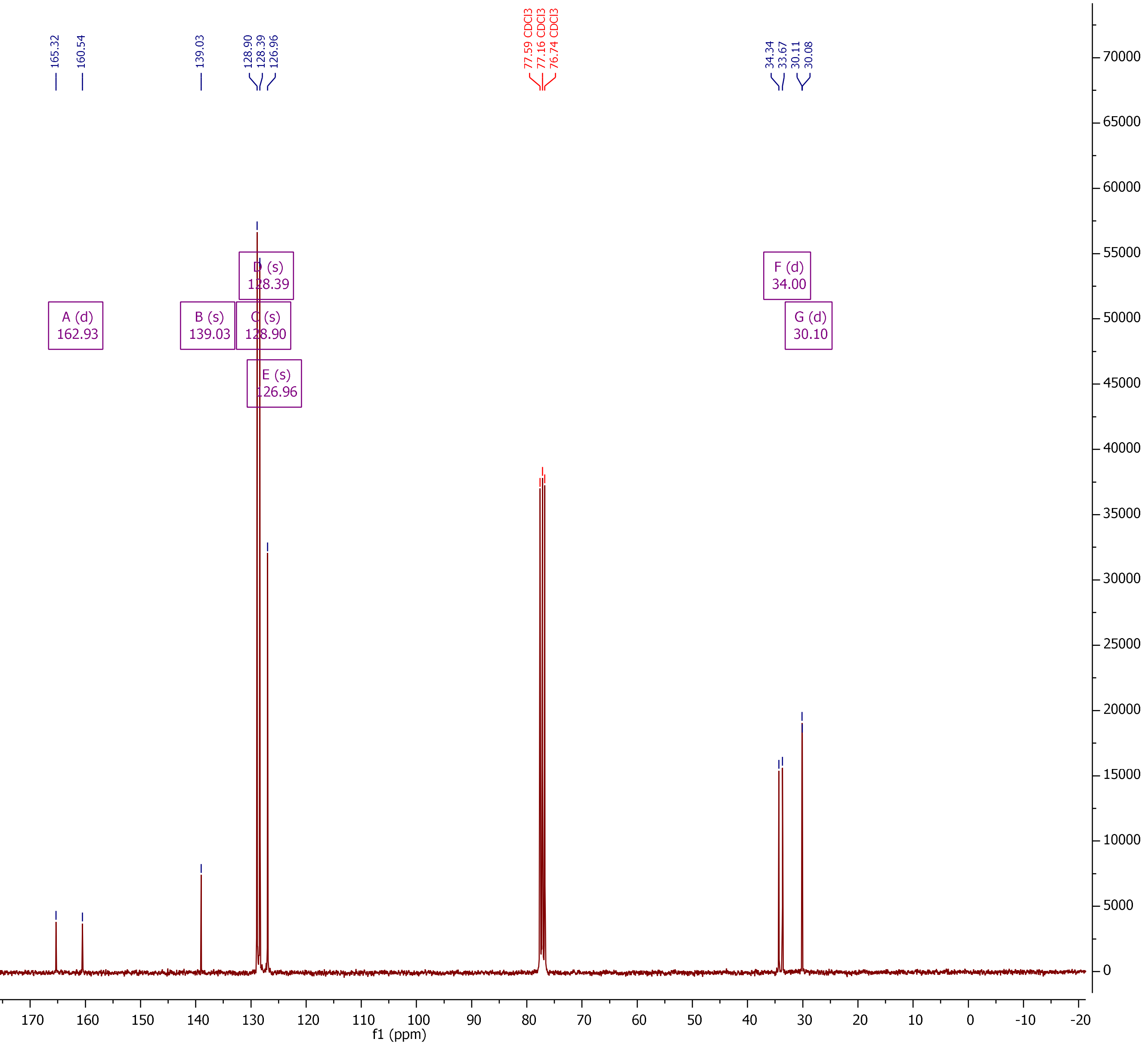

9m $^{19}F\{^{1}H\}$ NMR (282 MHz, Chloroform-*d*)

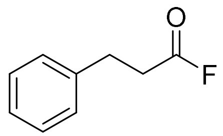

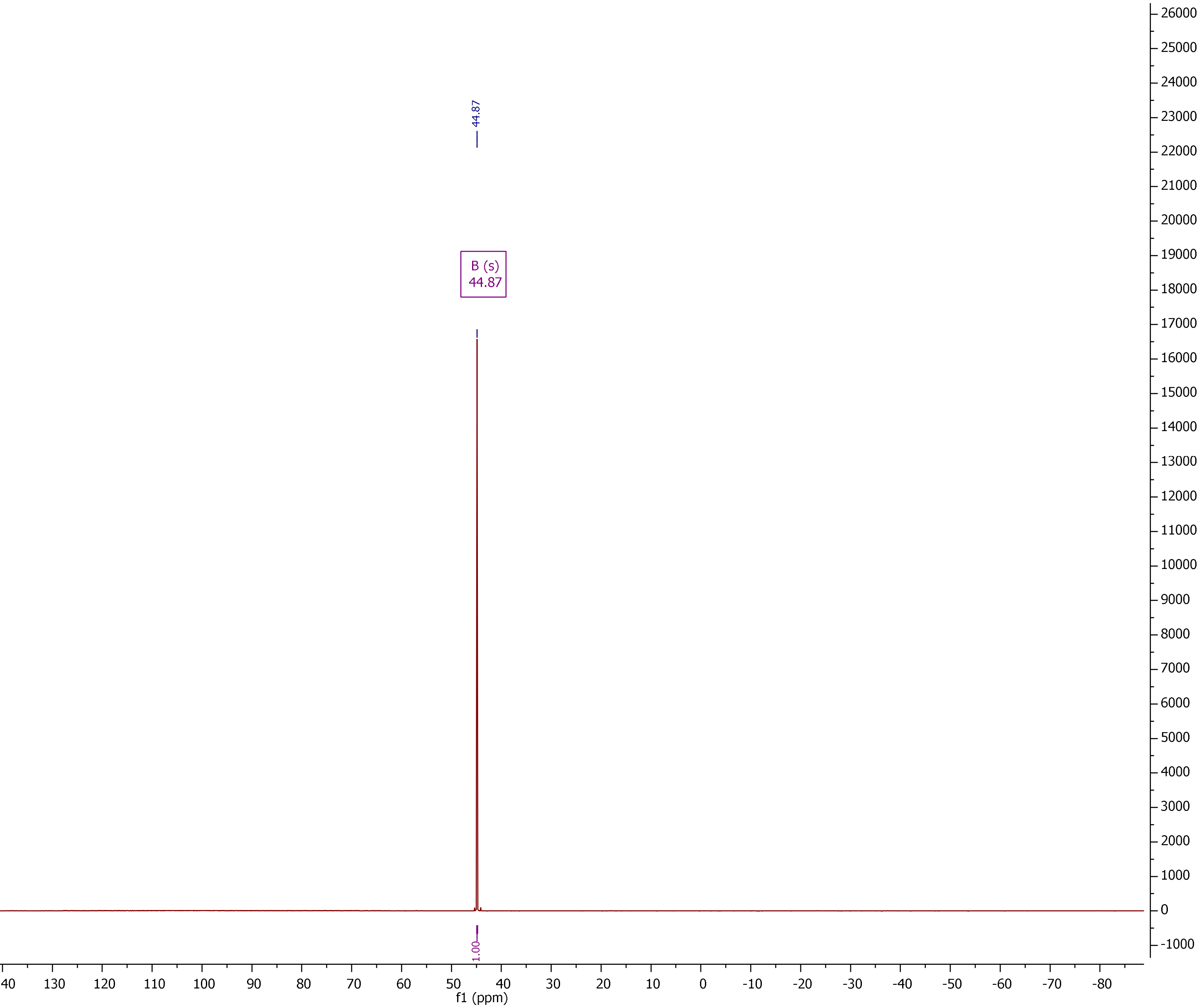

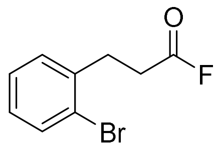

9n ^{1}H NMR (300 MHz, Chloroform-*d*)

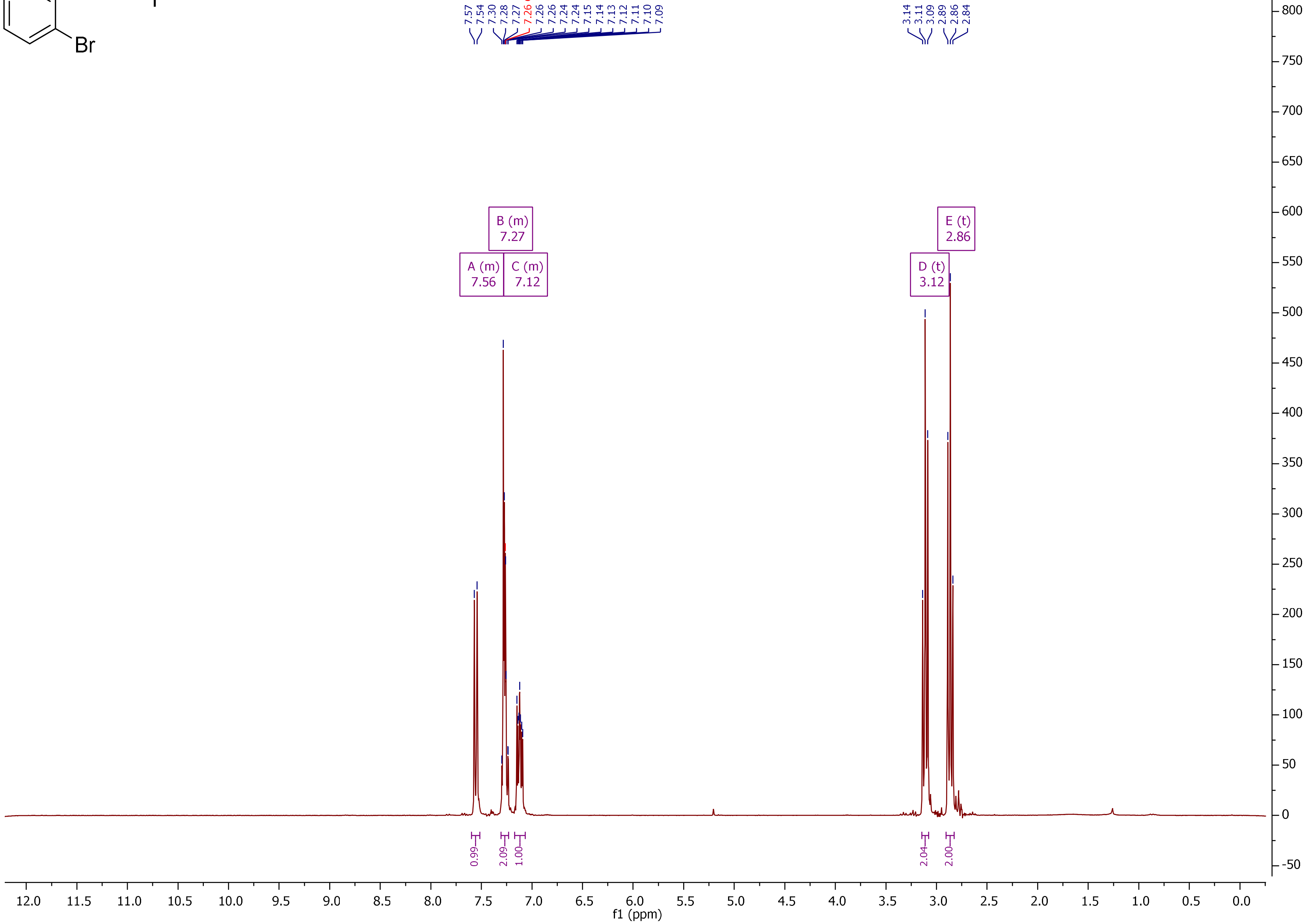

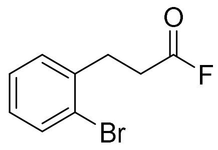

9n $^{13}C\{^{1}H\}$ NMR (75 MHz, Chloroform-*d*)

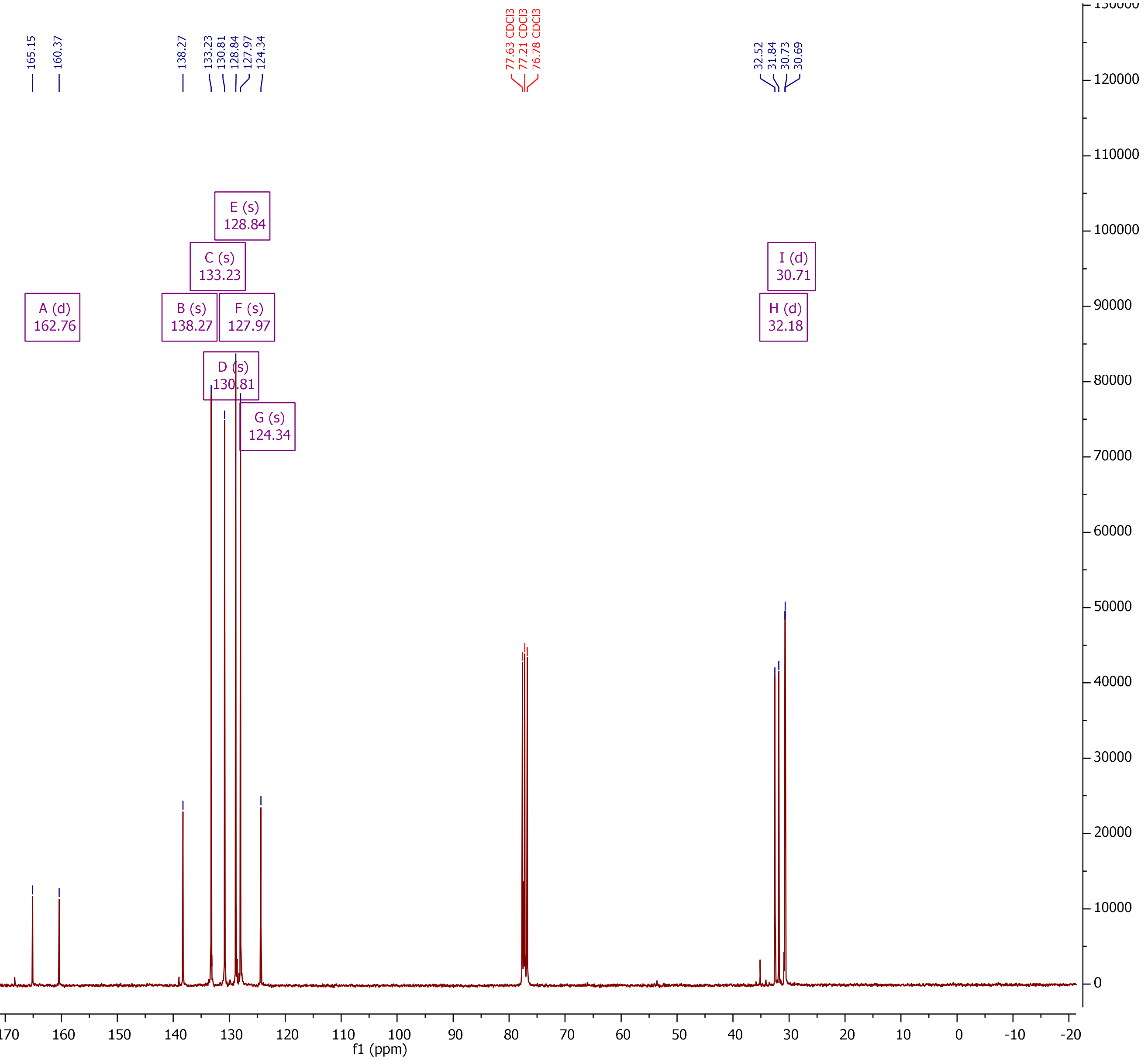

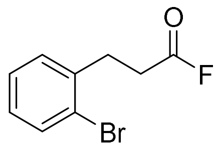

9n $^{19}F\{^{1}H\}$ NMR (282 MHz, Chloroform-*d*)

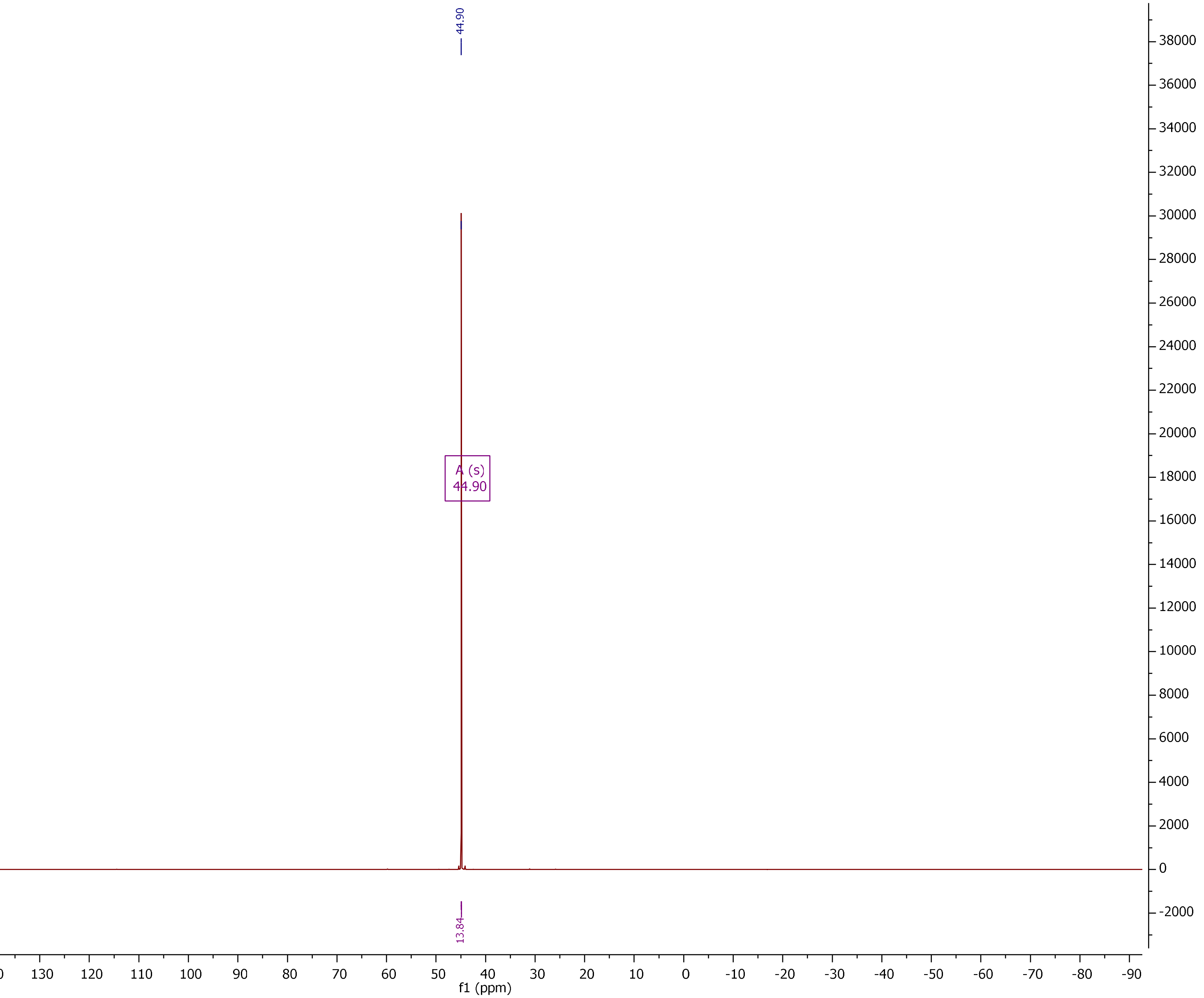

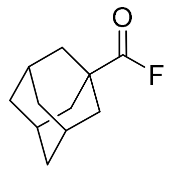

9o $^{19}F\{^{1}H\}$ NMR (282 MHz, Chloroform-*d*)

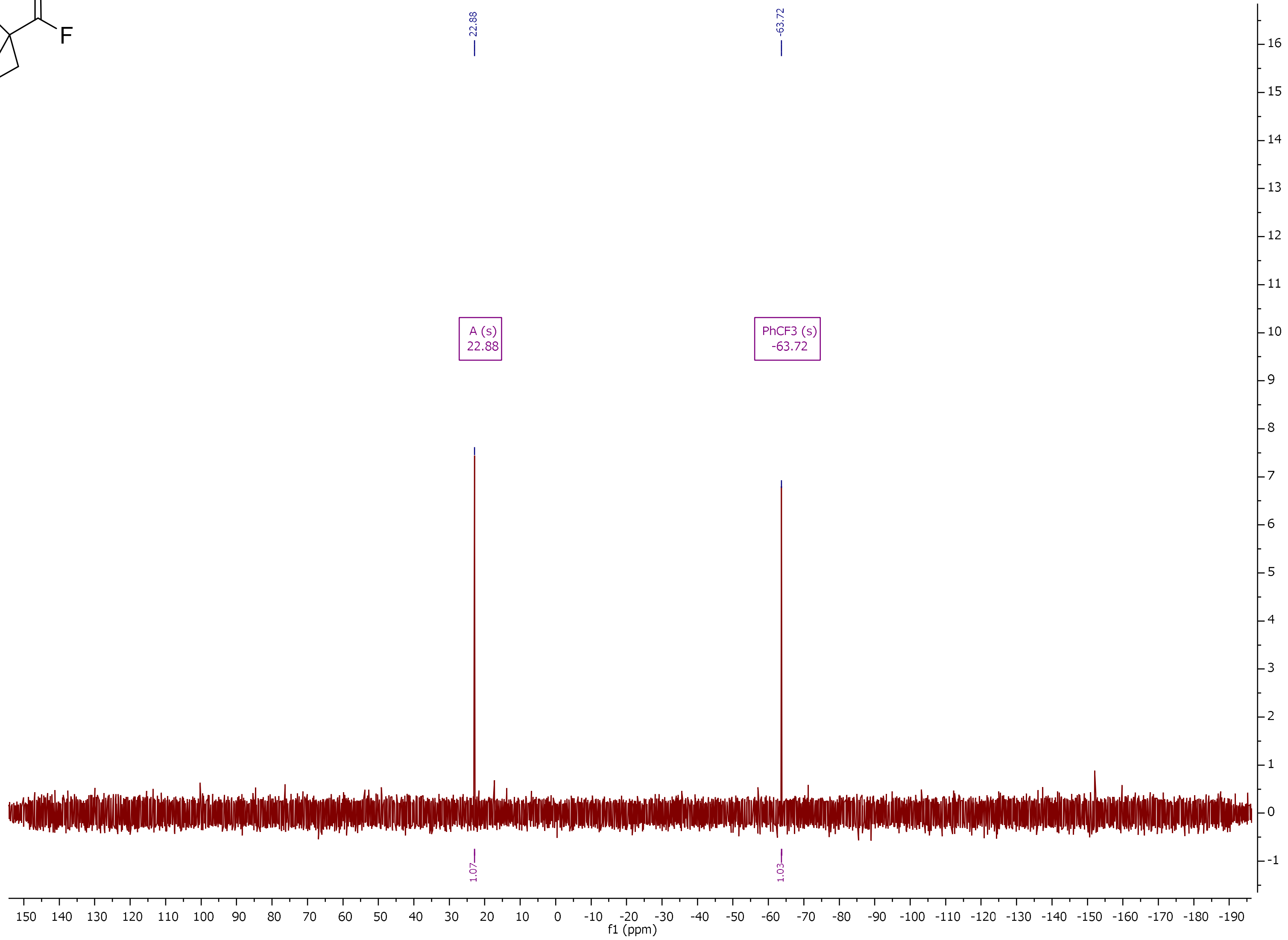

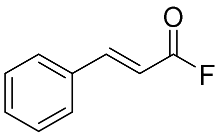

9p $^{19}F\{^{1}H\}$ NMR (282 MHz, Chloroform-*d*)

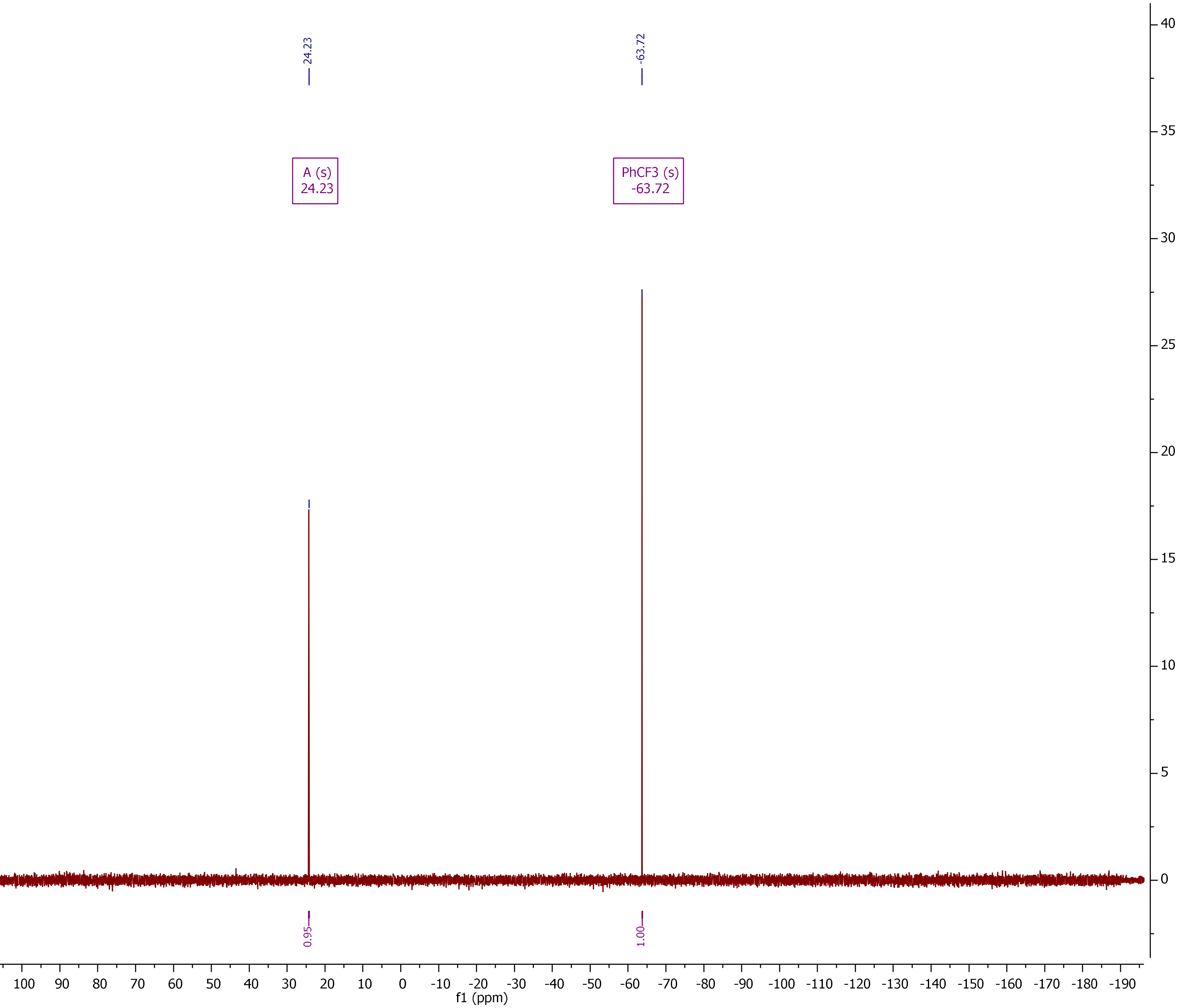

9q ¹H NMR (300 MHz, Chloroform-*d*)

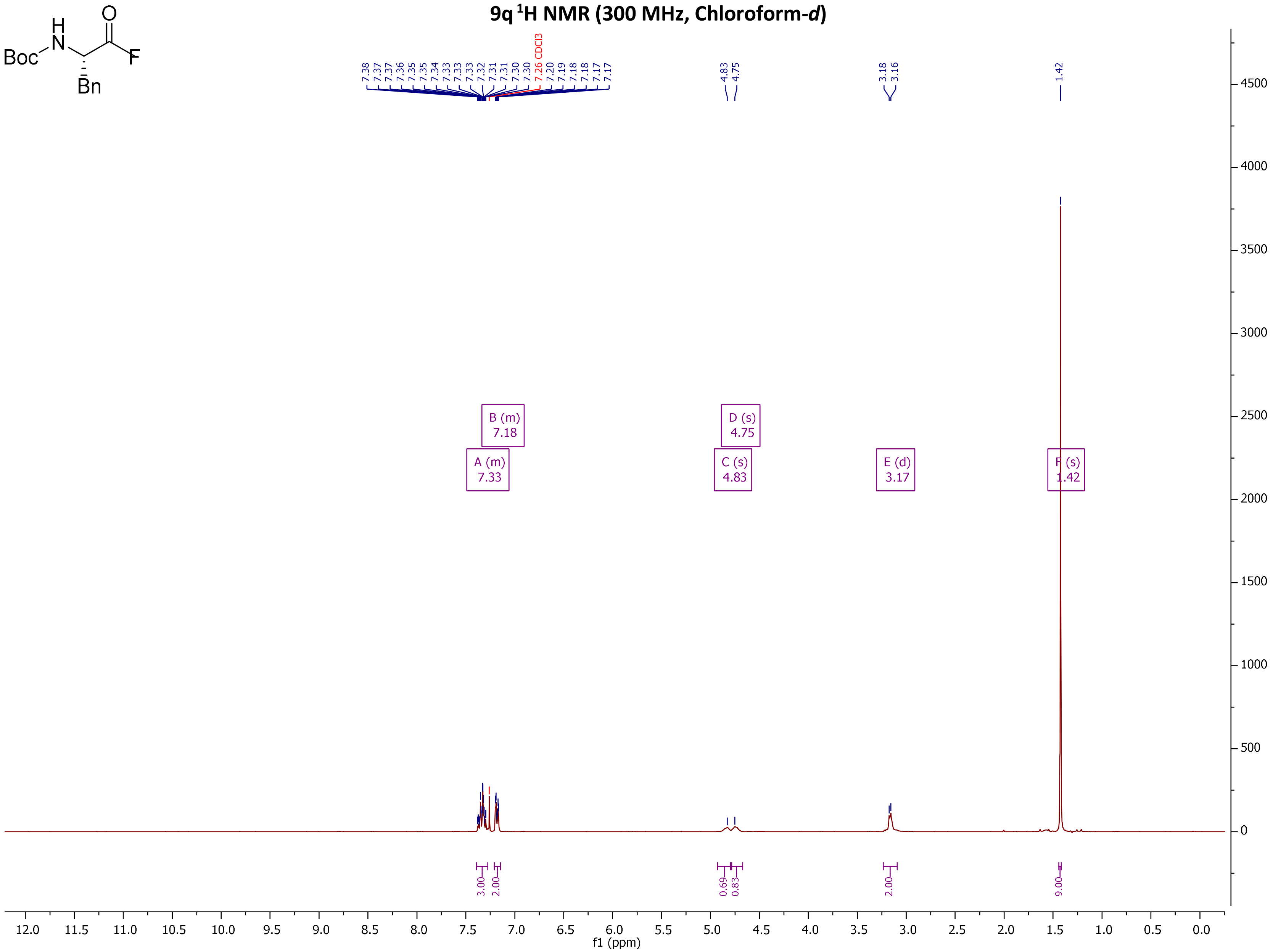

9q $^{13}C\{^{1}H\}$ NMR (75 MHz, Chloroform-*d*)

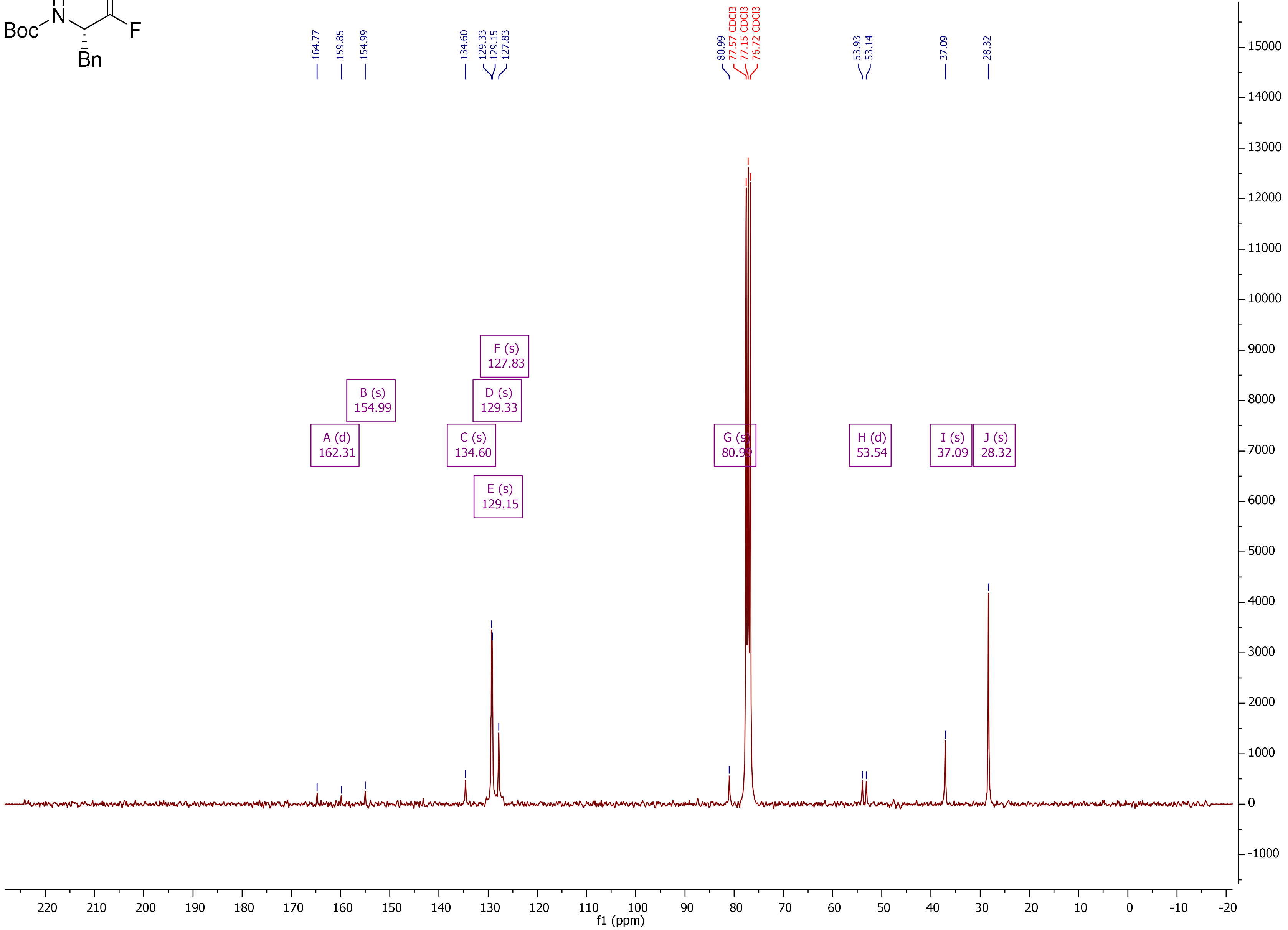

9q $^{19}F\{^{1}H\}$ NMR (282 MHz, Chloroform-*d*)

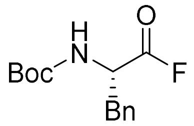

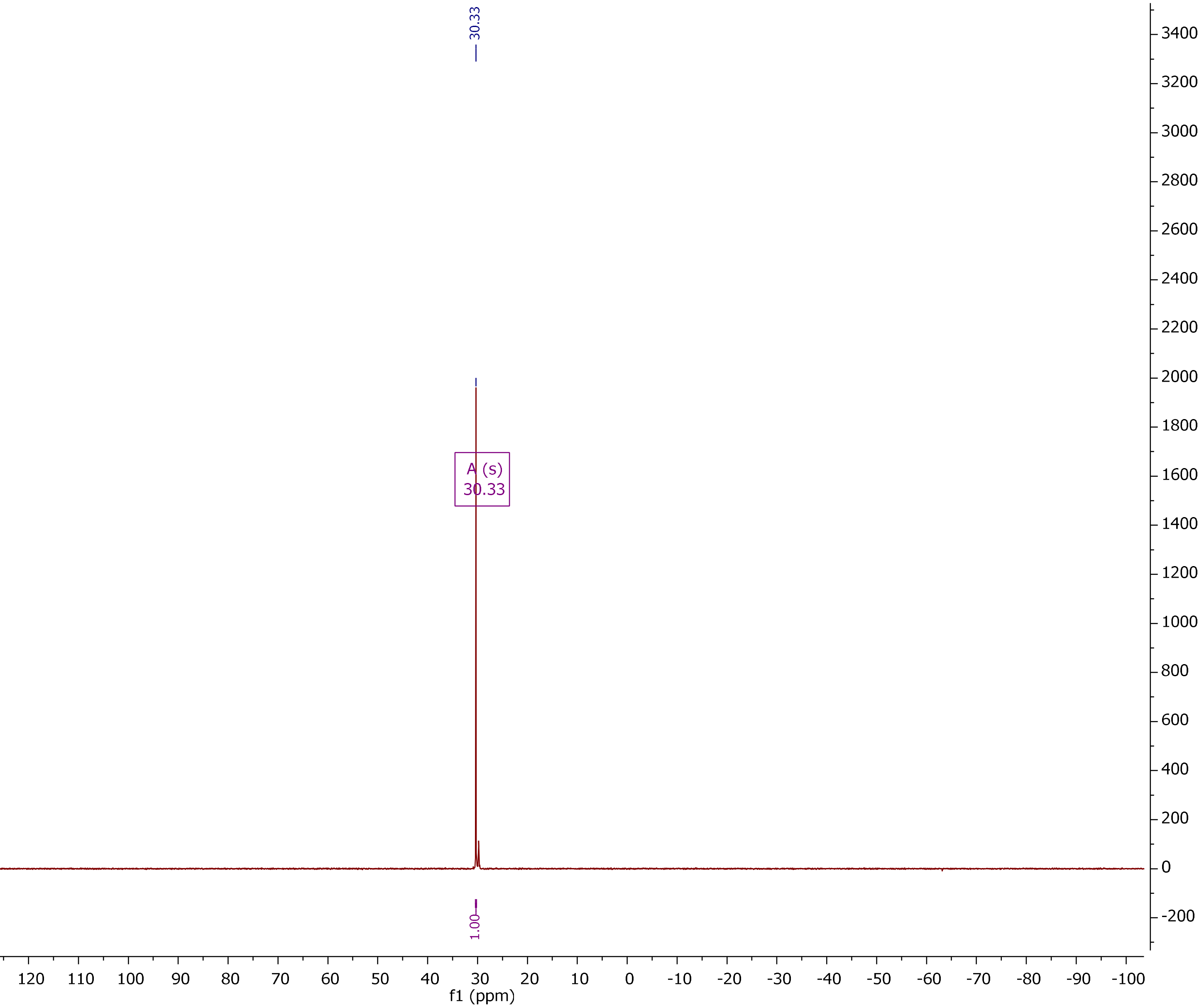

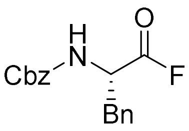

9r ^{1}H NMR (300 MHz, Chloroform-*d*)

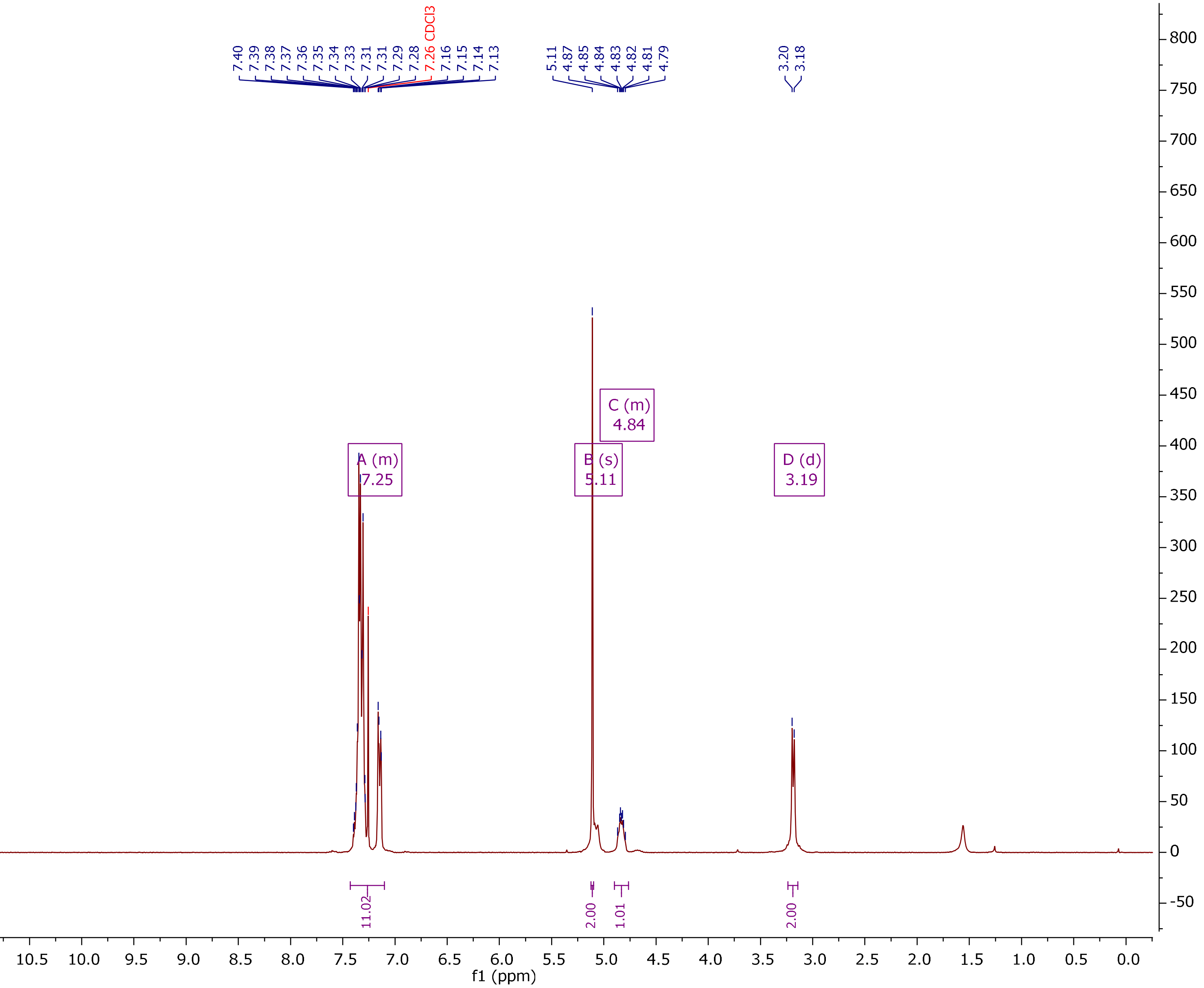

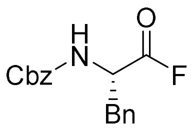

9r $^{13}C\{^1H\}$ NMR (75 MHz, Chloroform-*d*)

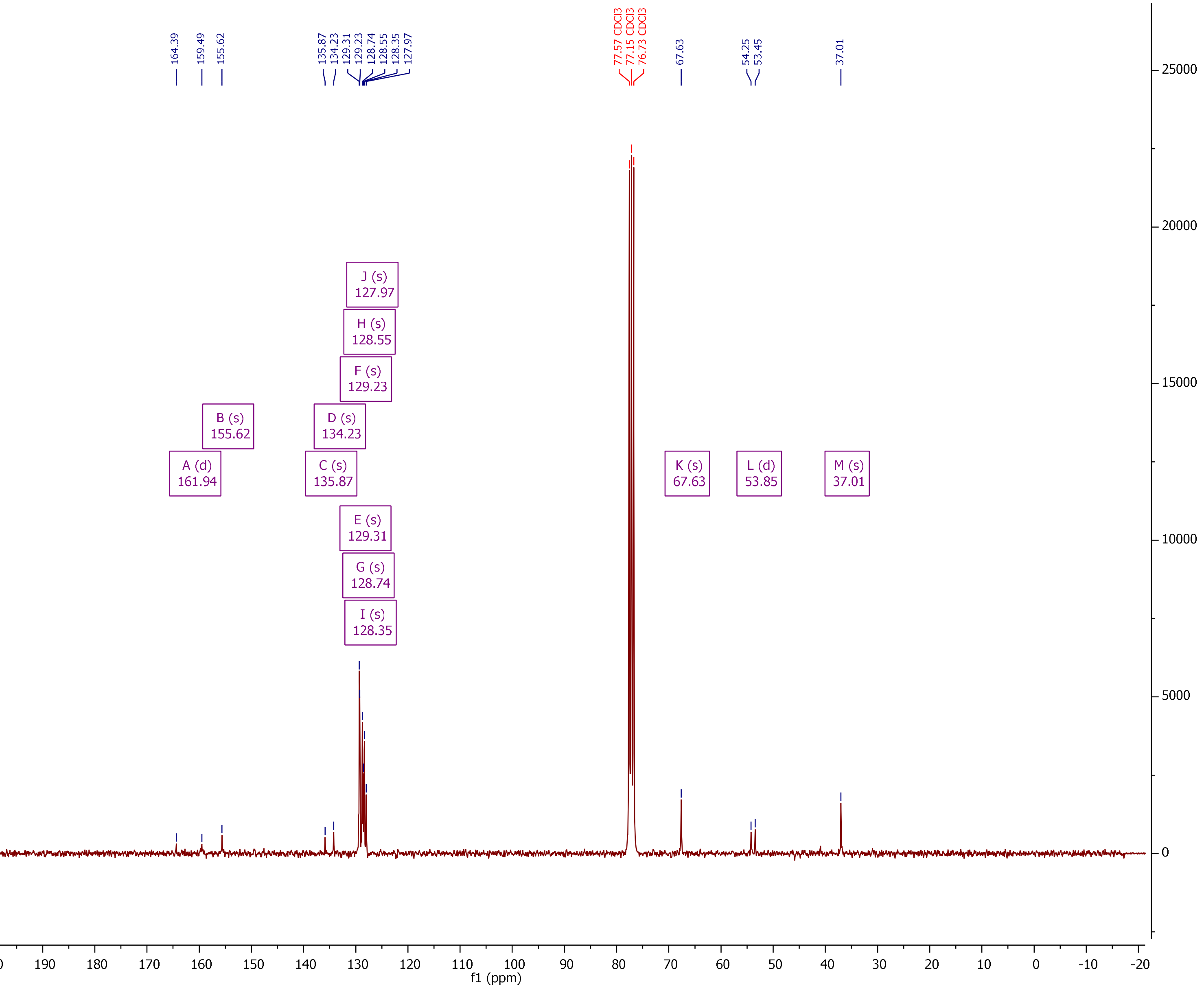

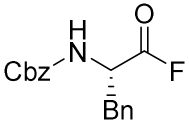

9r $^{19}F\{^1H\}$ NMR (282 MHz, Chloroform-*d*)

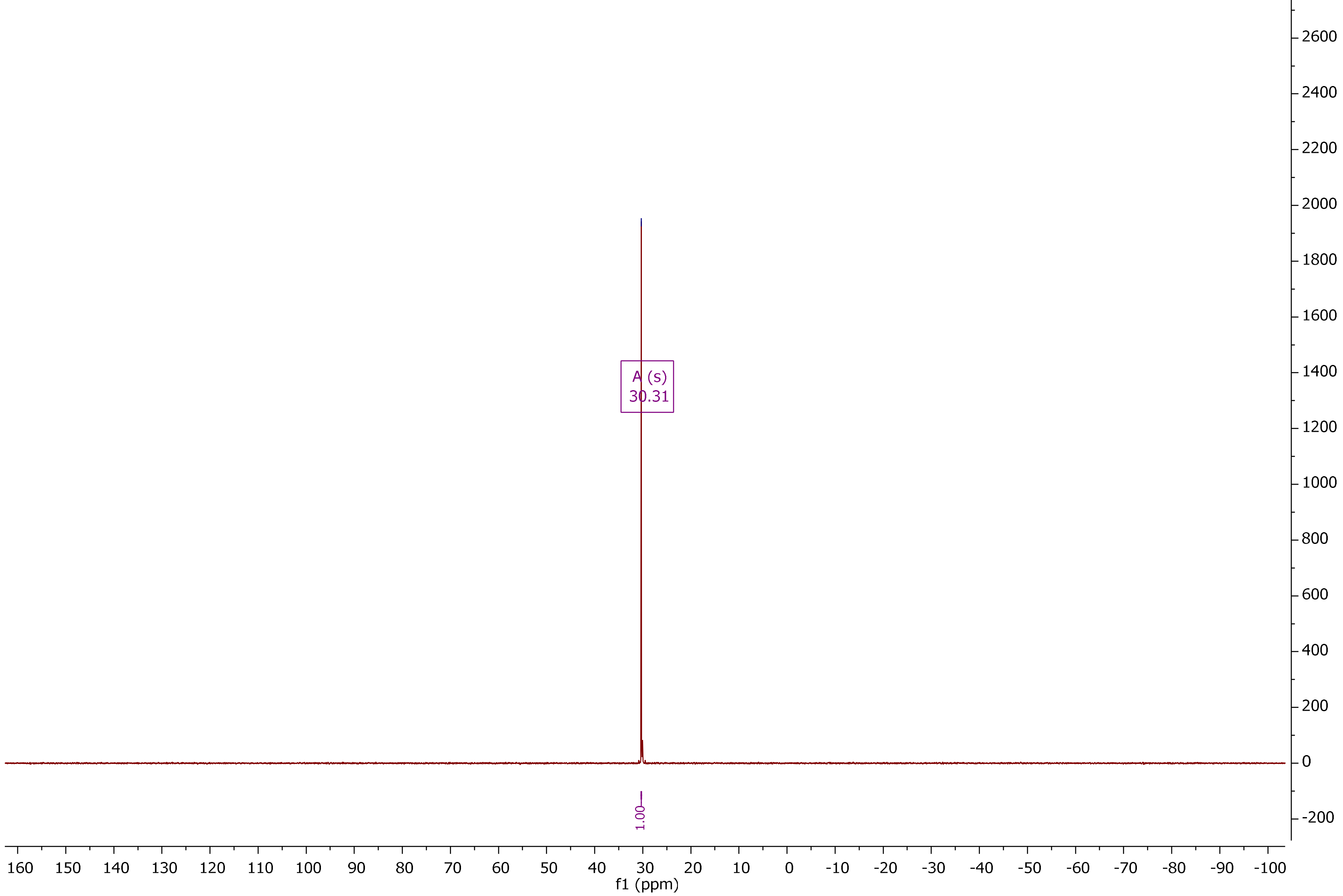

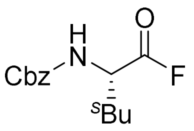

9s ¹H NMR (300 MHz, Chloroform-*d*)

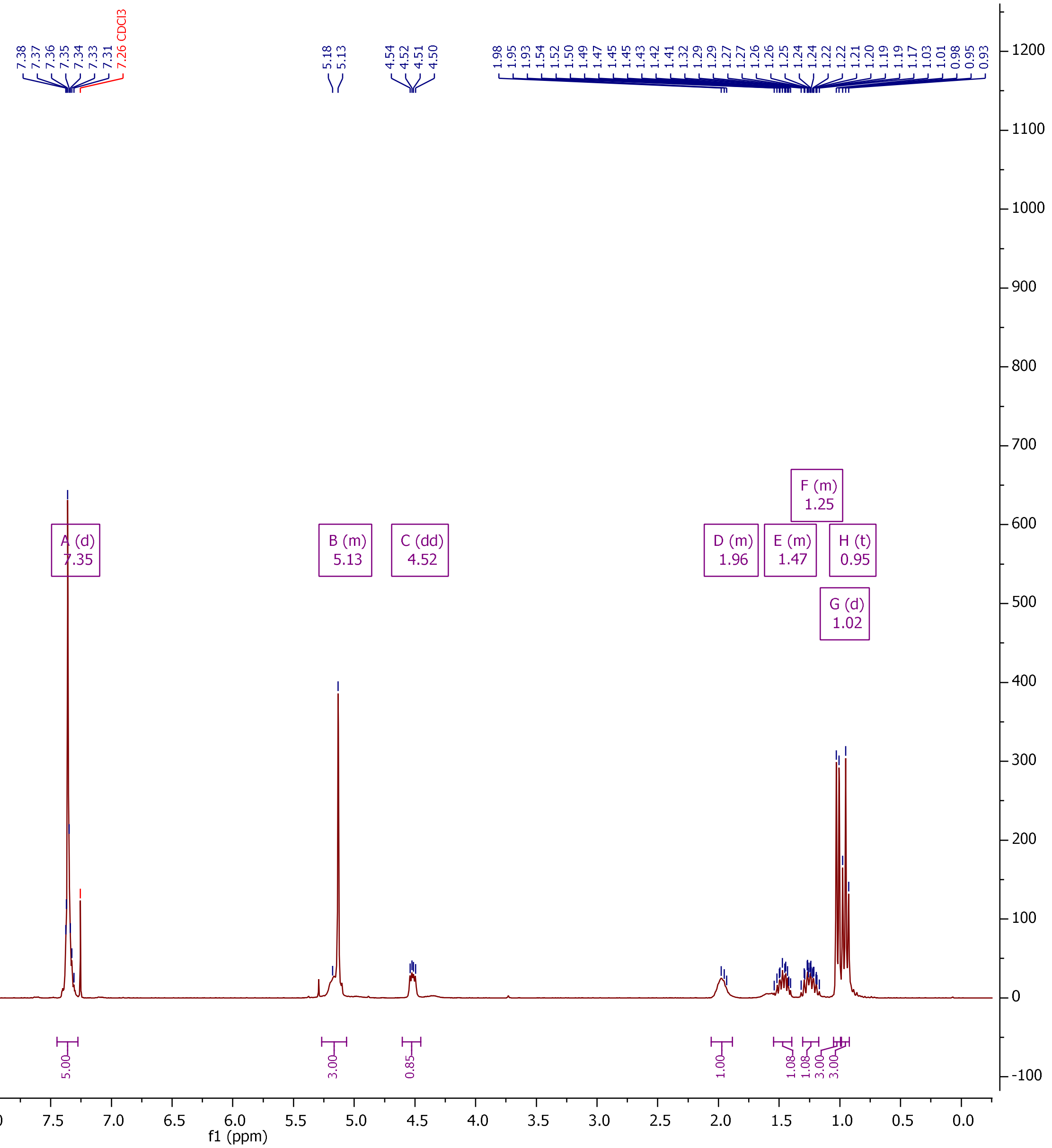

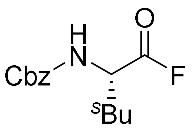

9s $^{13}C\{^{1}H\}$ NMR (75 MHz, Chloroform-*d*)

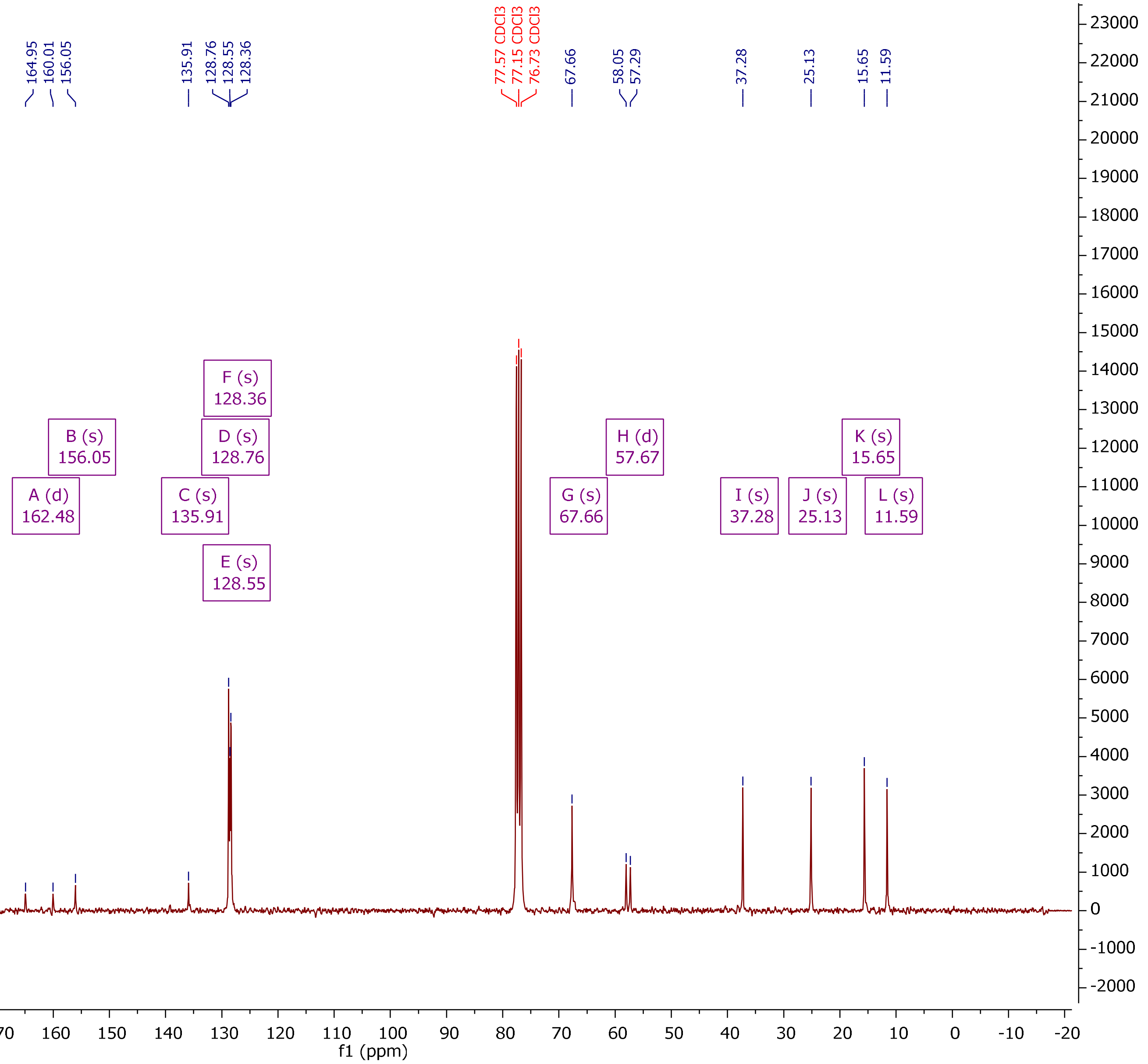

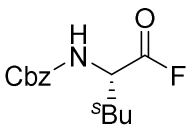

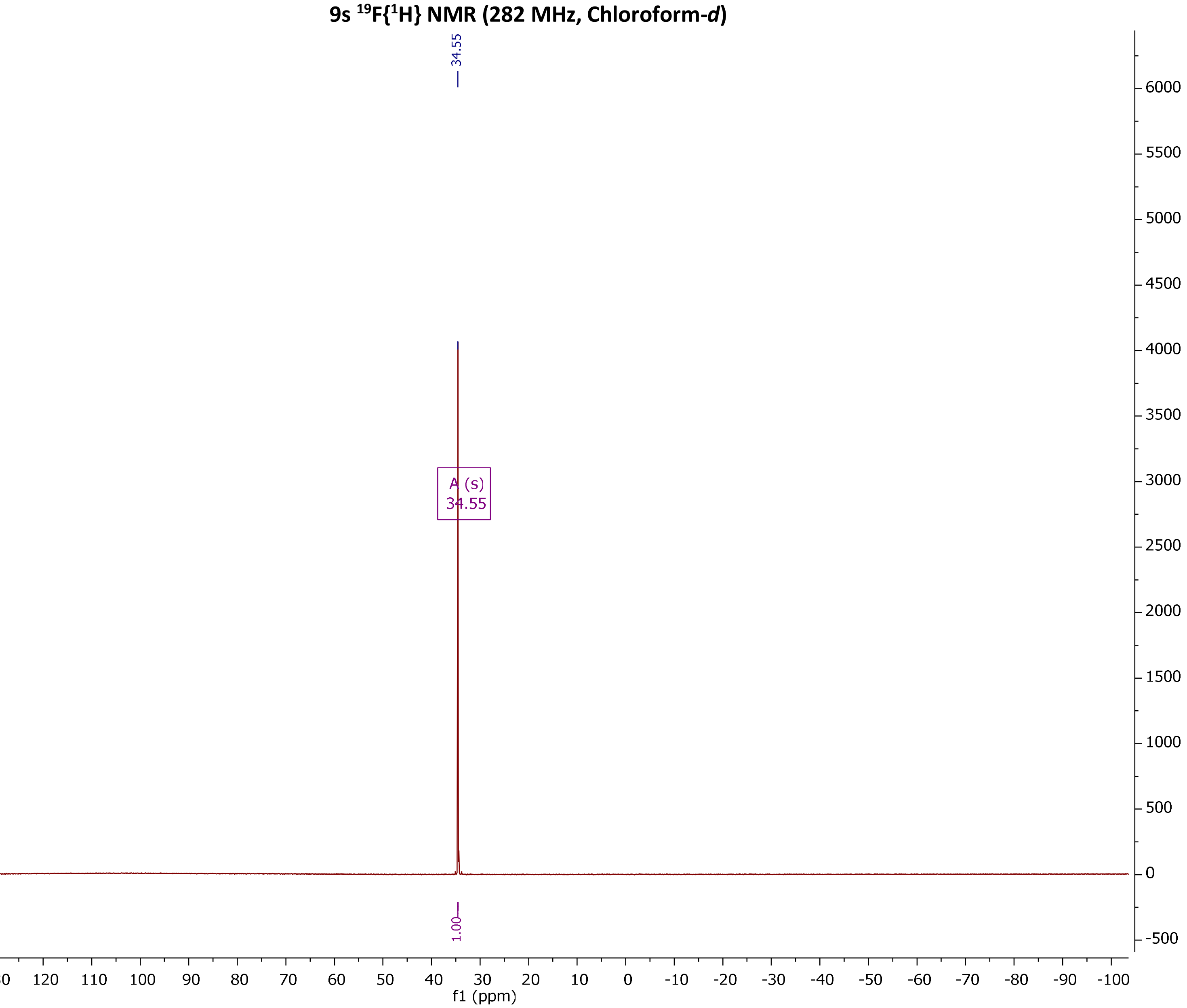
9s ^{19}F{^{1}H} NMR (282 MHz, Chloroform-*d*)
34.55
A (s)
34.55
1.00
6000
5500
5000
4500
4000
3500
3000
2500
2000
1500
1000
500
0
-500
160 150 140 130 120 110 100 90 80 70 60 50 40 30 20 10 0 -10 -20 -30 -40 -50 -60 -70 -80 -90 -100
f1 (ppm)

9t ^{1}H NMR (300 MHz, Chloroform-*d*)

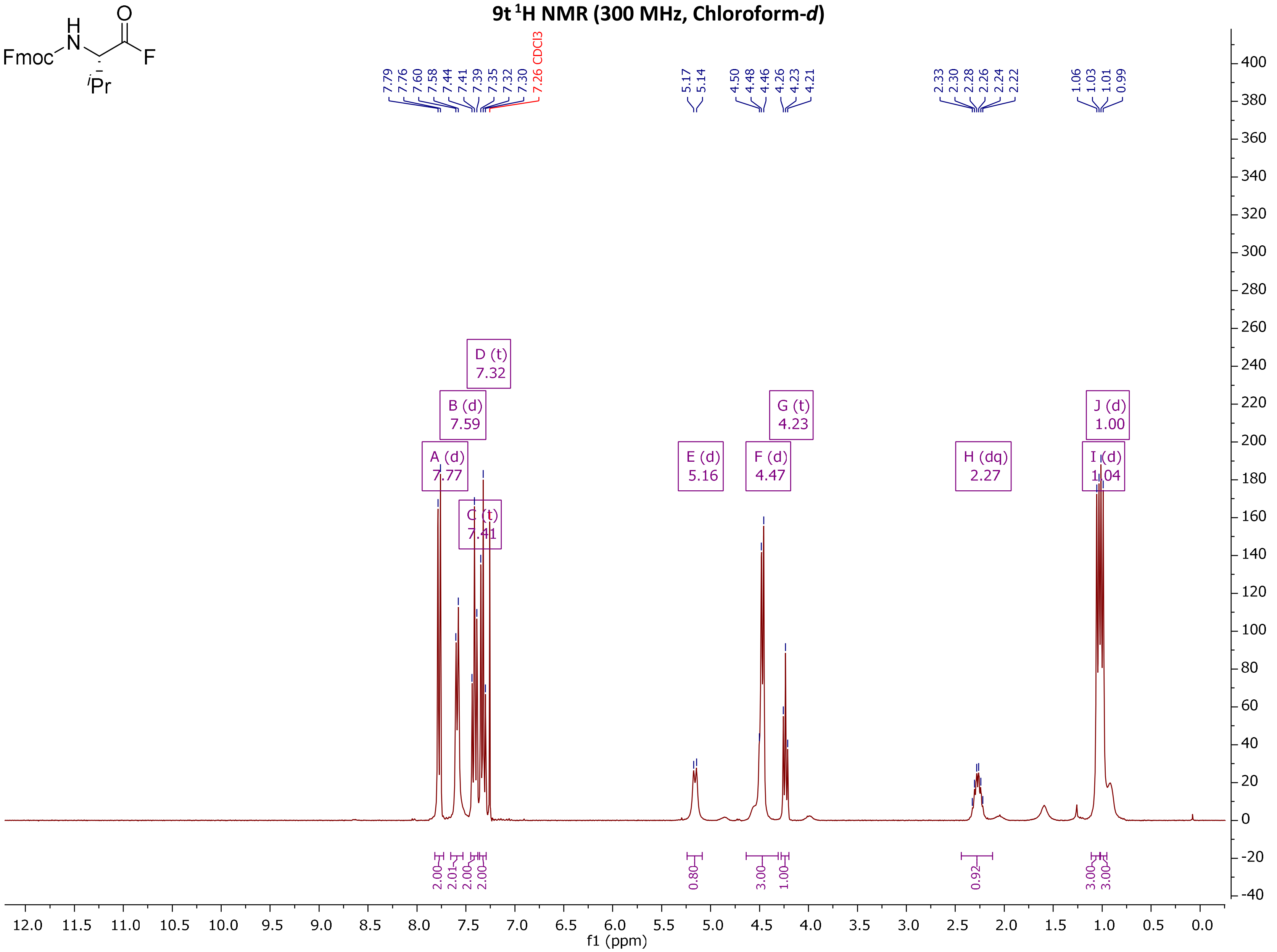

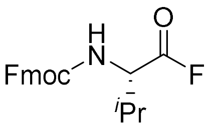

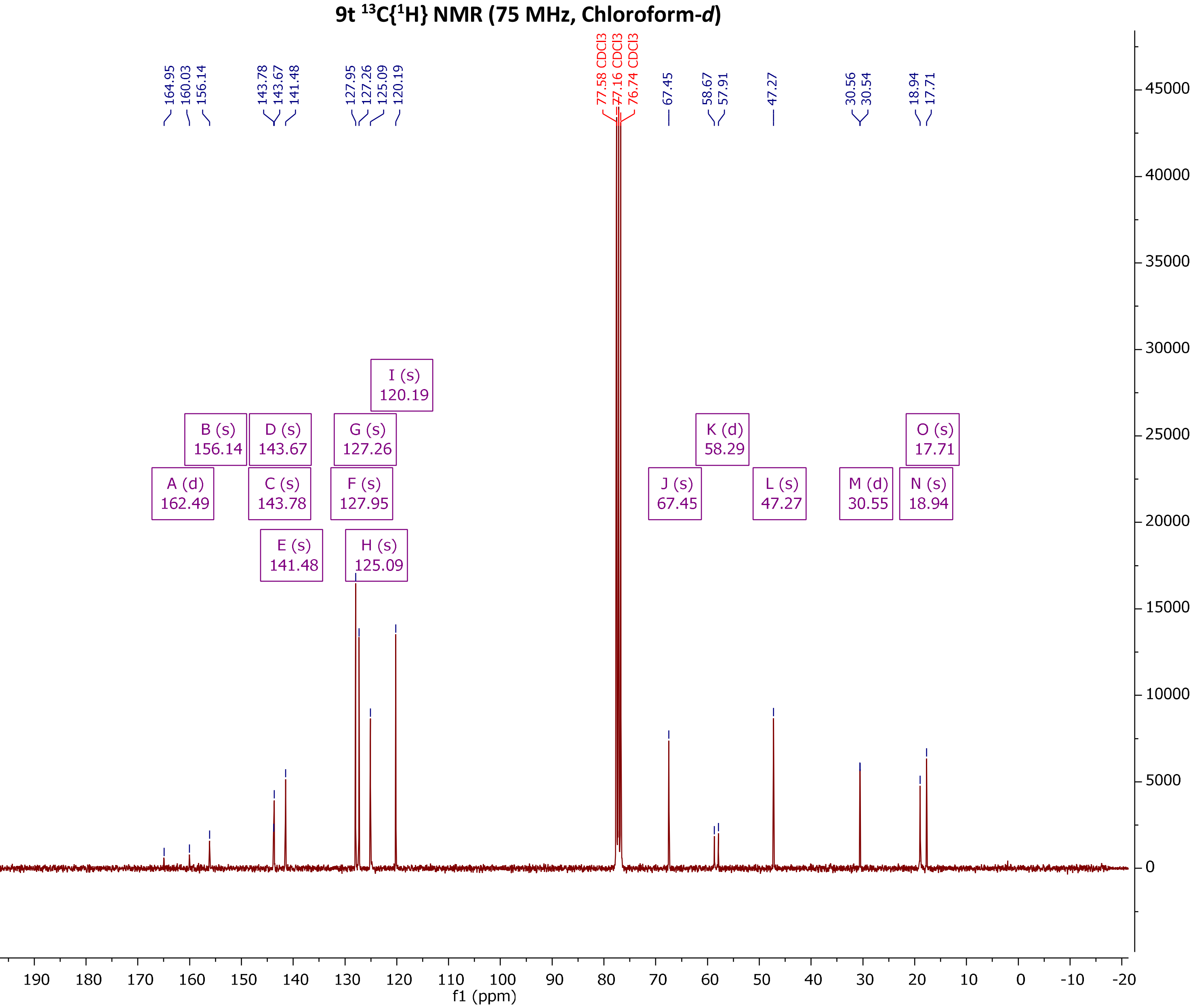
9t $^{13}C\{^1H\}$ NMR (75 MHz, Chloroform-*d*)
164.95
160.03
156.14
143.78
143.67
141.48
127.95
127.26
125.09
120.19
77.58 CDCl3
77.16 CDCl3
76.74 CDCl3
67.45
58.67
57.91
47.27
30.56
30.54
18.94
17.71
A (d) 162.49
B (s) 156.14
C (s) 143.78
D (s) 143.67
E (s) 141.48
F (s) 127.95
G (s) 127.26
H (s) 125.09
I (s) 120.19
J (s) 67.45
K (d) 58.29
L (s) 47.27
M (d) 30.55
N (s) 18.94
O (s) 17.71
f1 (ppm)

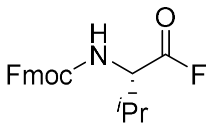
Fmoc
H
N
O
F
iPr

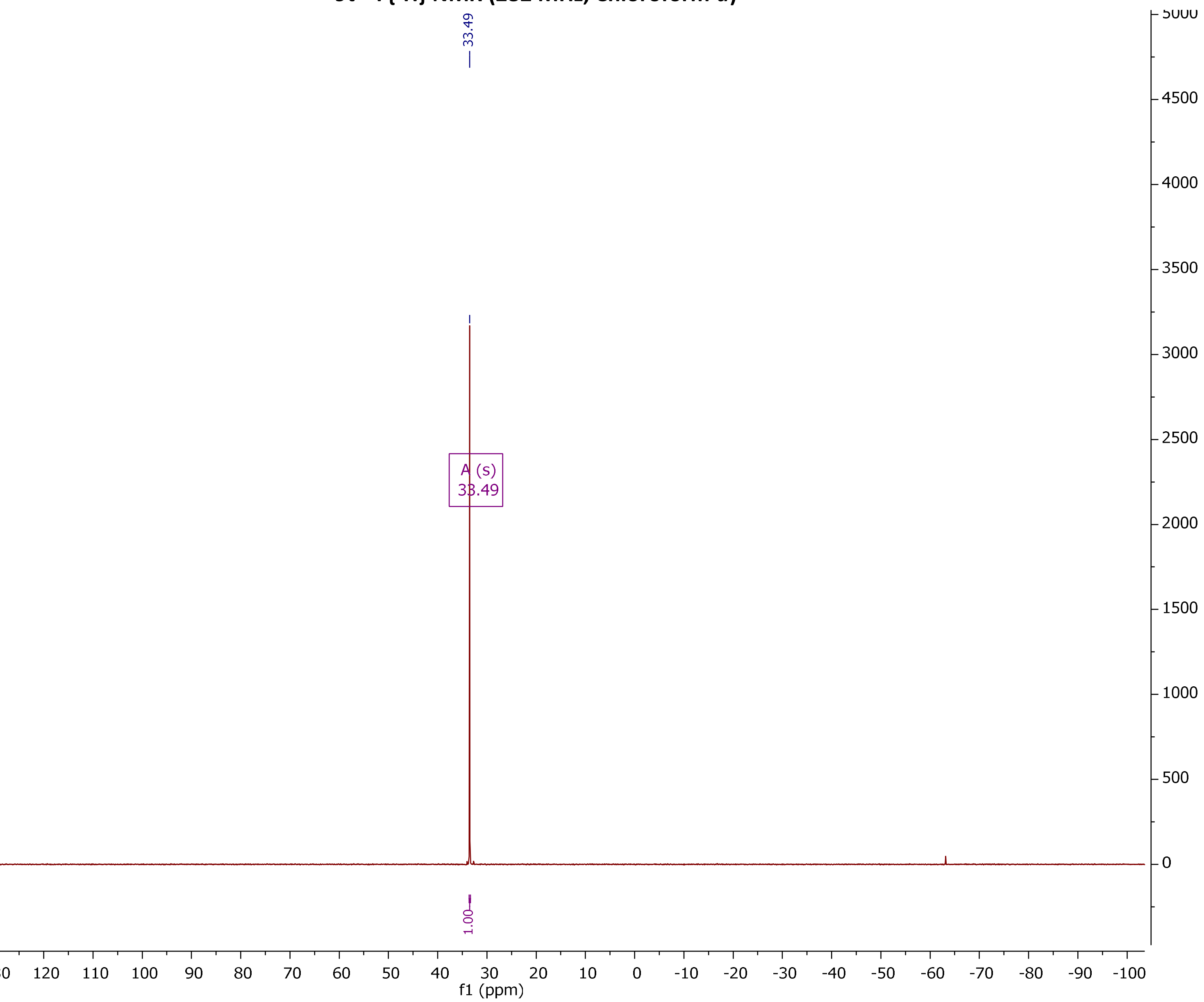
9t 19F{1H} NMR (282 MHz, Chloroform-d)
33.49
A (s)
33.49
1.00
f1 (ppm)

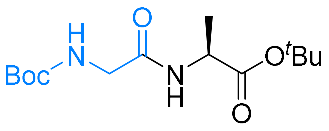

10a ^{1}H NMR (300 MHz, Chloroform-d)

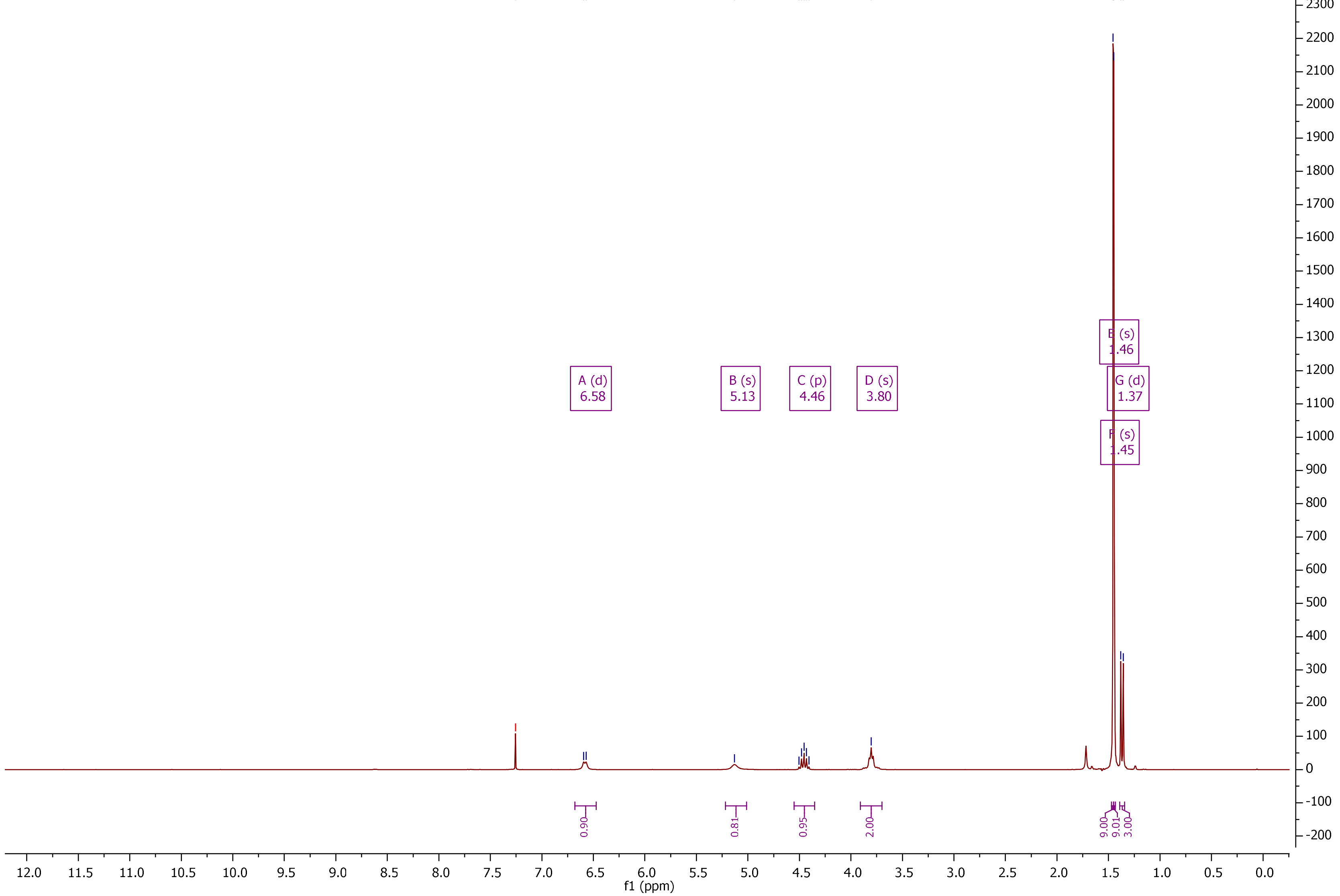

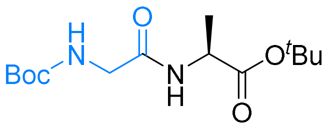

10a $^{13}C\{^1H\}$ NMR (75 MHz, Chloroform-d)

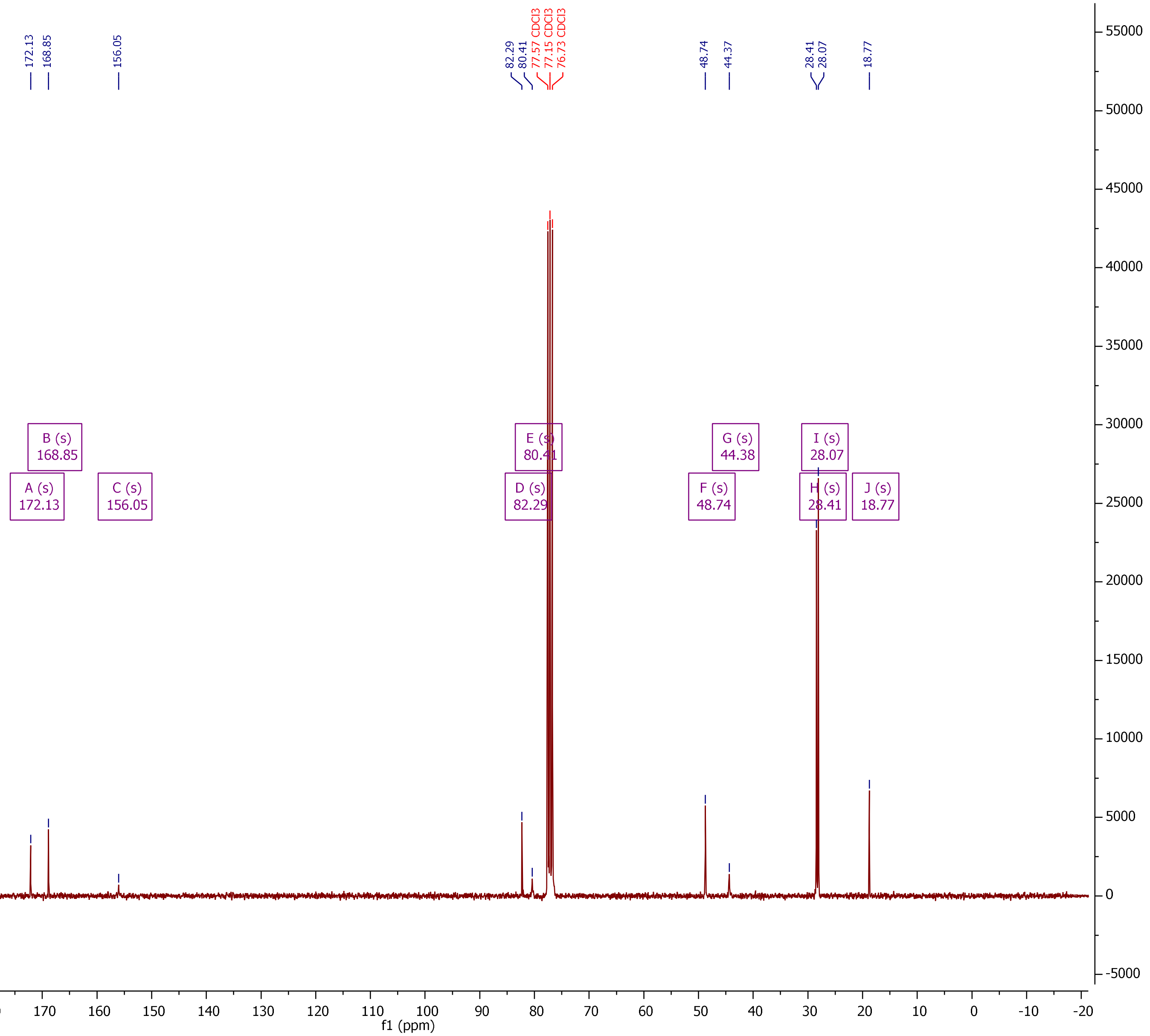

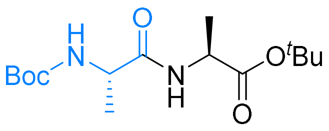

10b ^{1}H NMR (300 MHz, Chloroform-*d*)

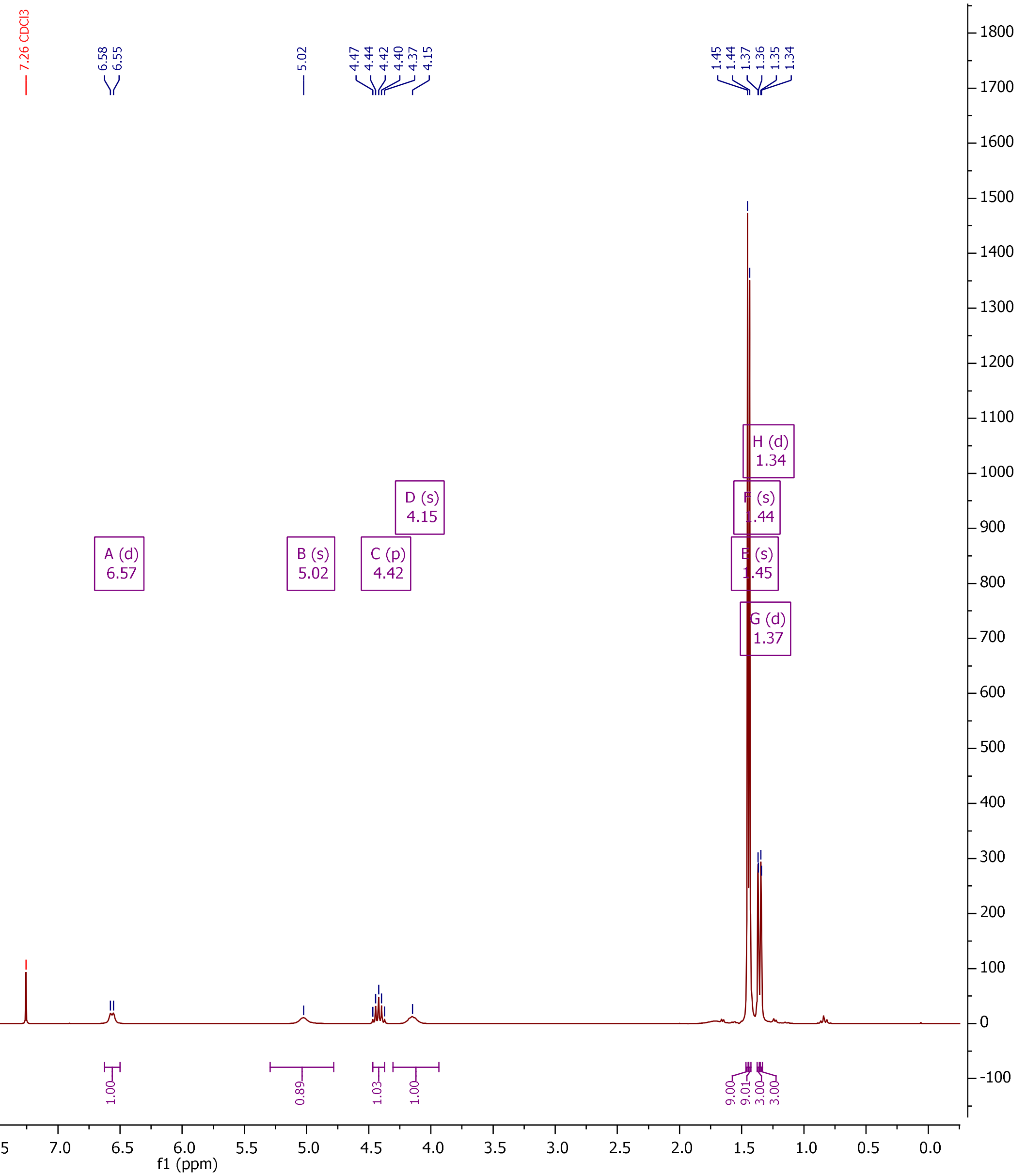

10b $^{13}C\{^{1}H\}$ NMR (75 MHz, Chloroform-*d*)

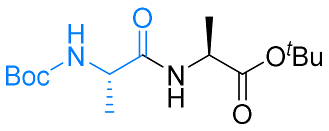

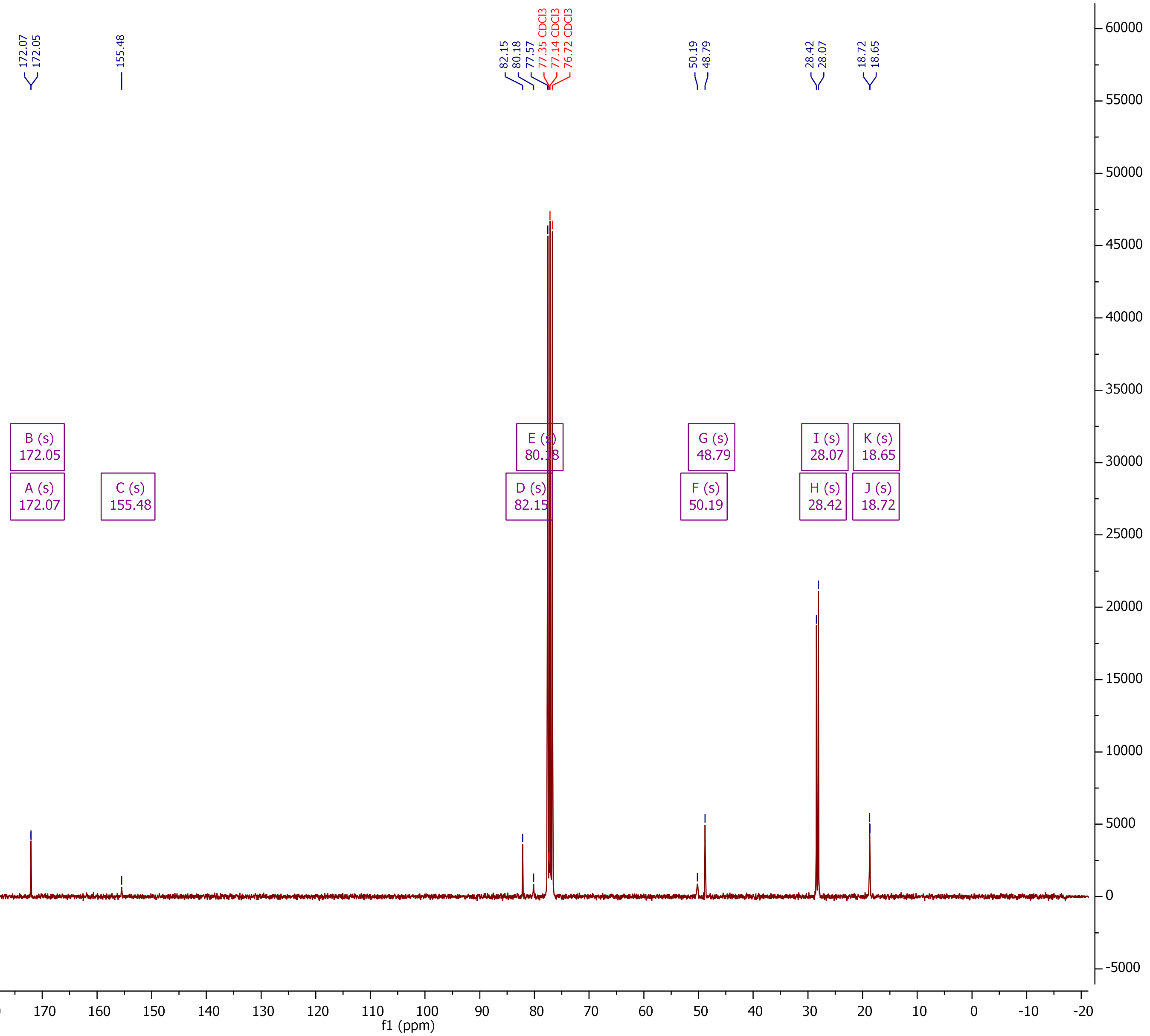

10c ^{1}H NMR (300 MHz, Chloroform-*d*)

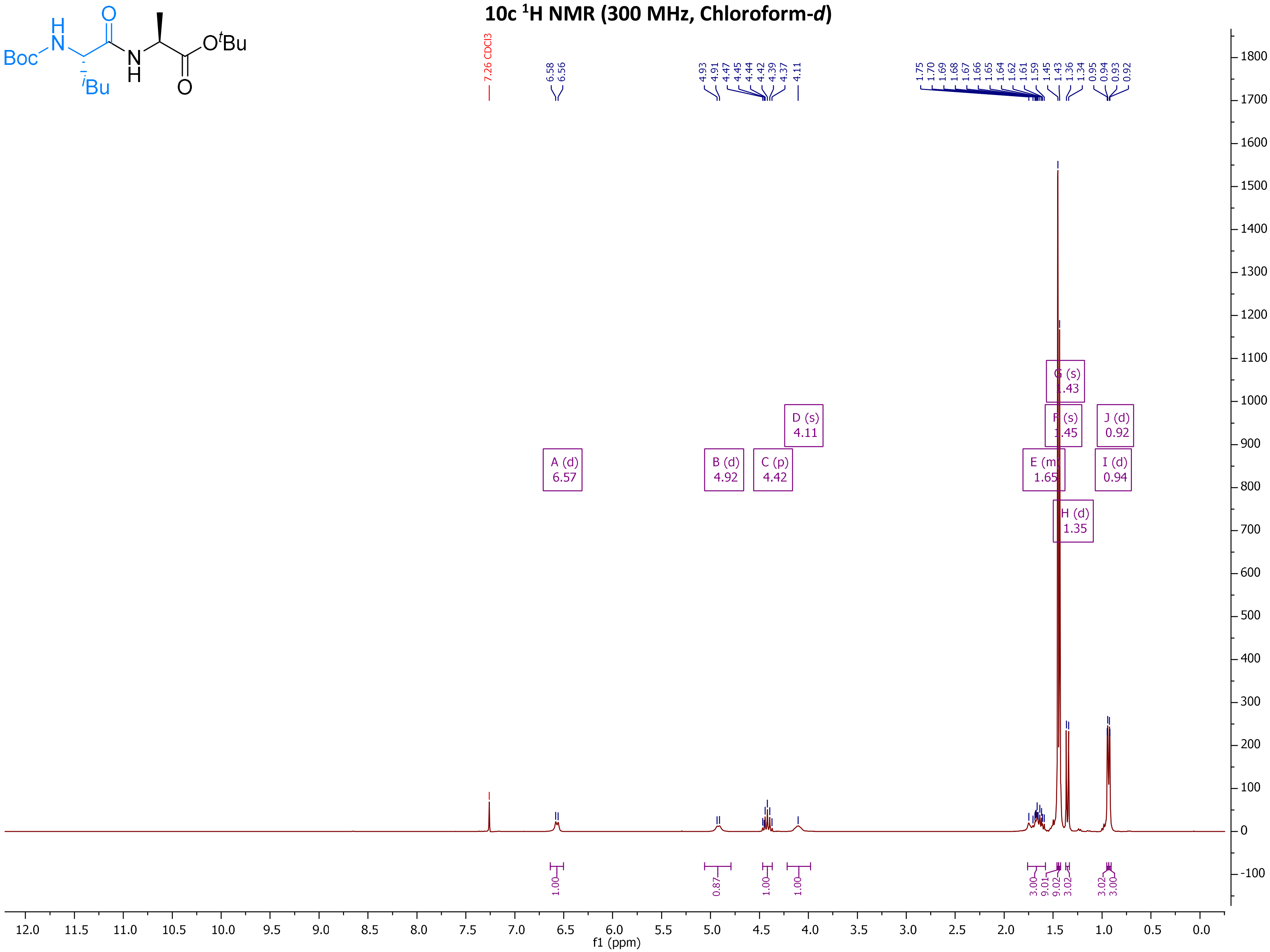

10c $^{13}C\{^{1}H\}$ NMR (75 MHz, Chloroform-*d*)

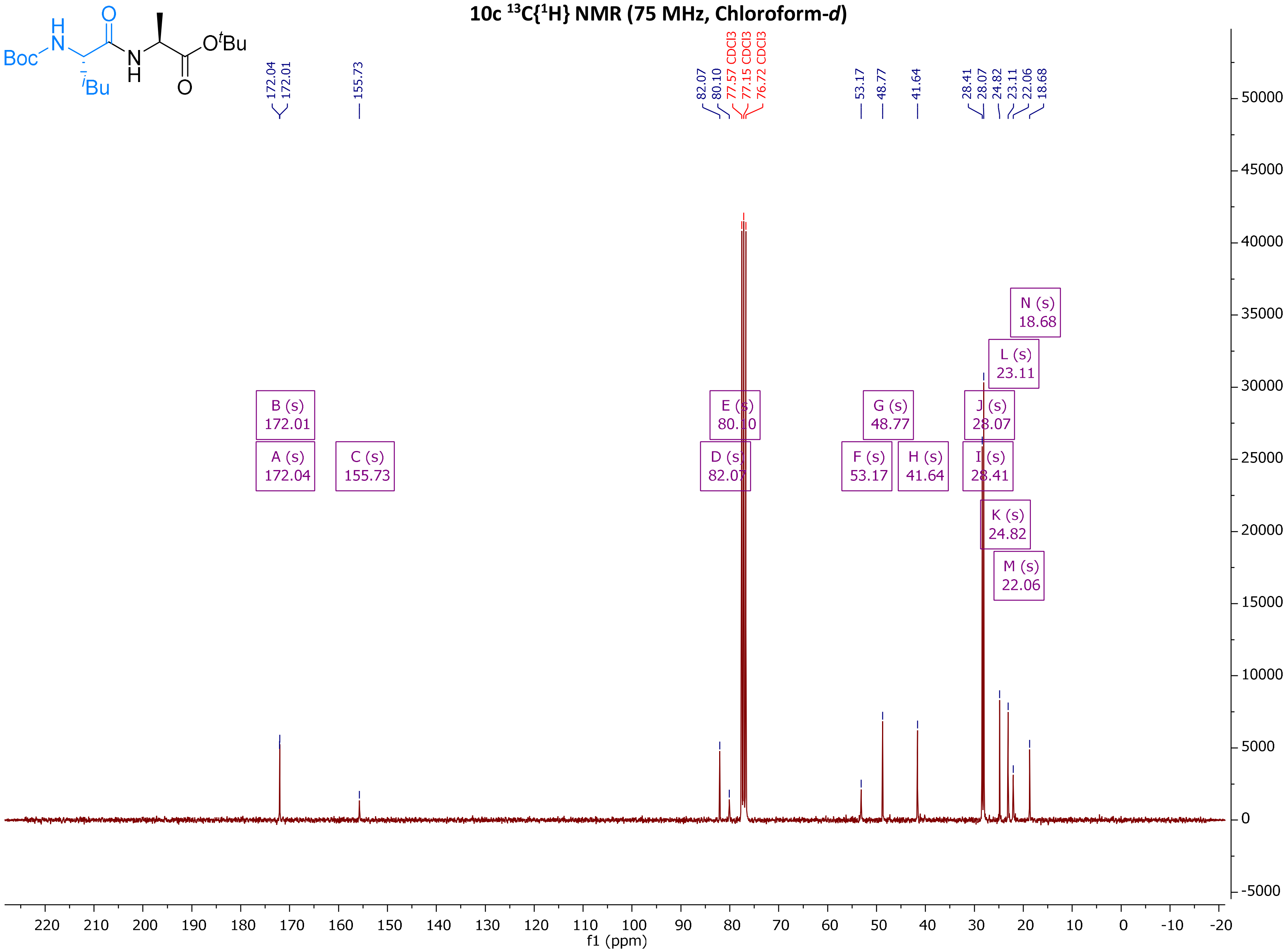

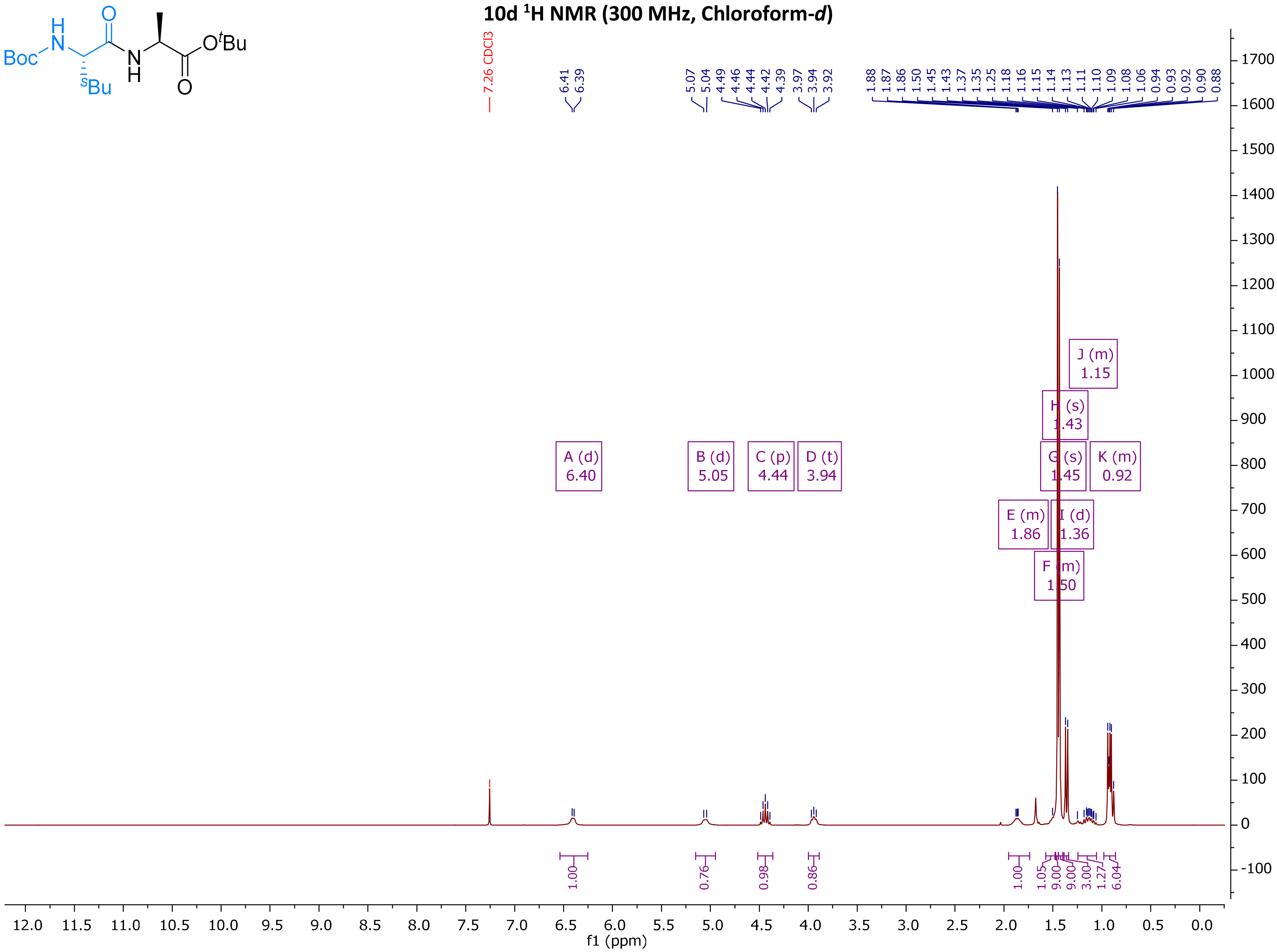
10d ¹H NMR (300 MHz, Chloroform-d)
Boc
O'Bu
sBu
7.26 CDCl3
A (d) 6.40
B (d) 5.05
C (p) 4.44
D (t) 3.94
E (m) 1.86
F (m) 1.50
G (s) 1.45
H (s) 1.43
I (d) 1.36
J (m) 1.15
K (m) 0.92
f1 (ppm)

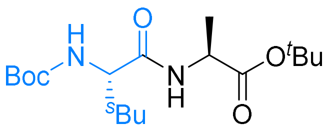

10d $^{13}C\{^1H\}$ NMR (75 MHz, Chloroform-*d*)

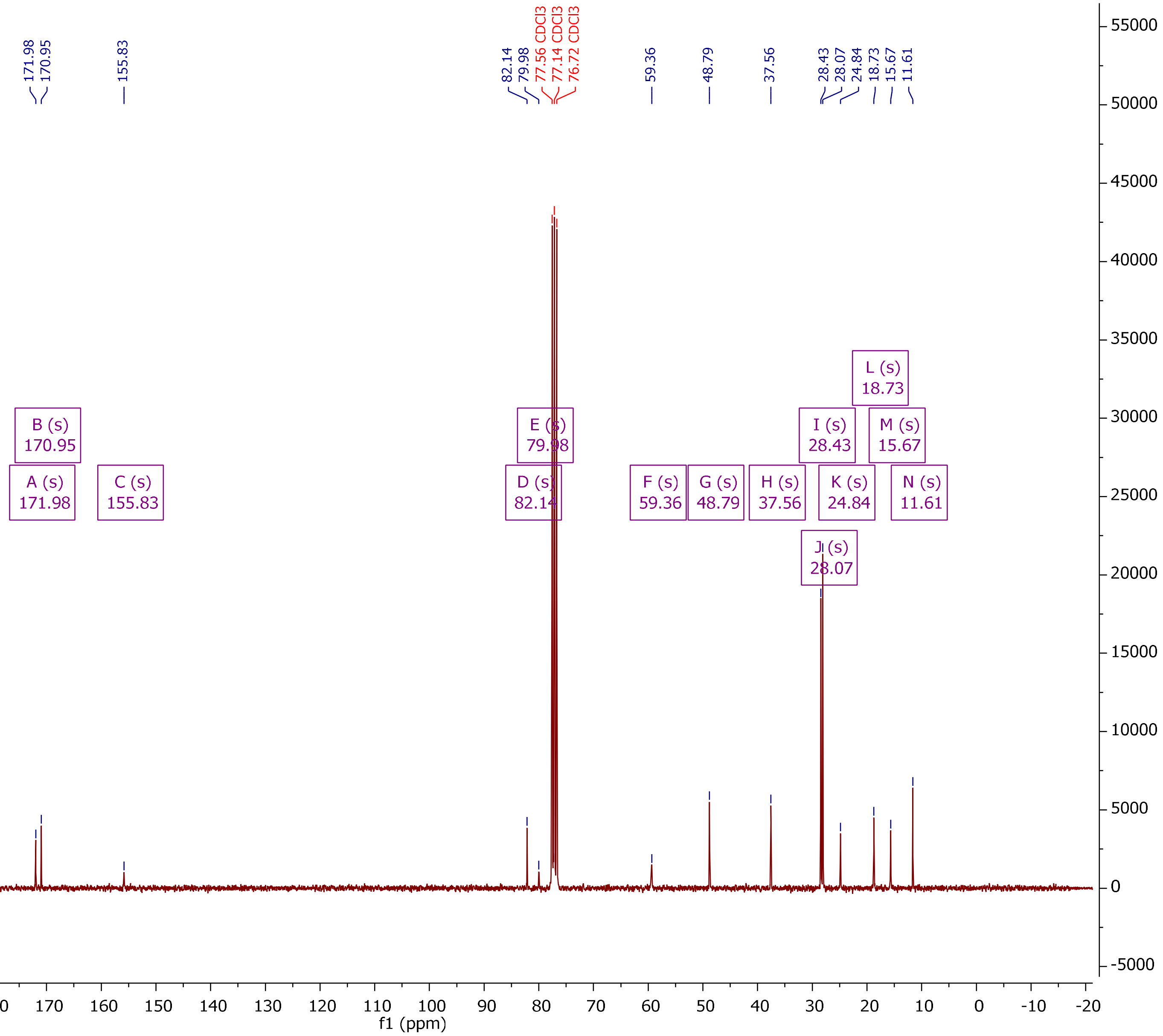

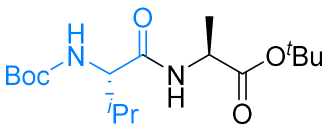

10e ^{1}H NMR (300 MHz, Chloroform-*d*)

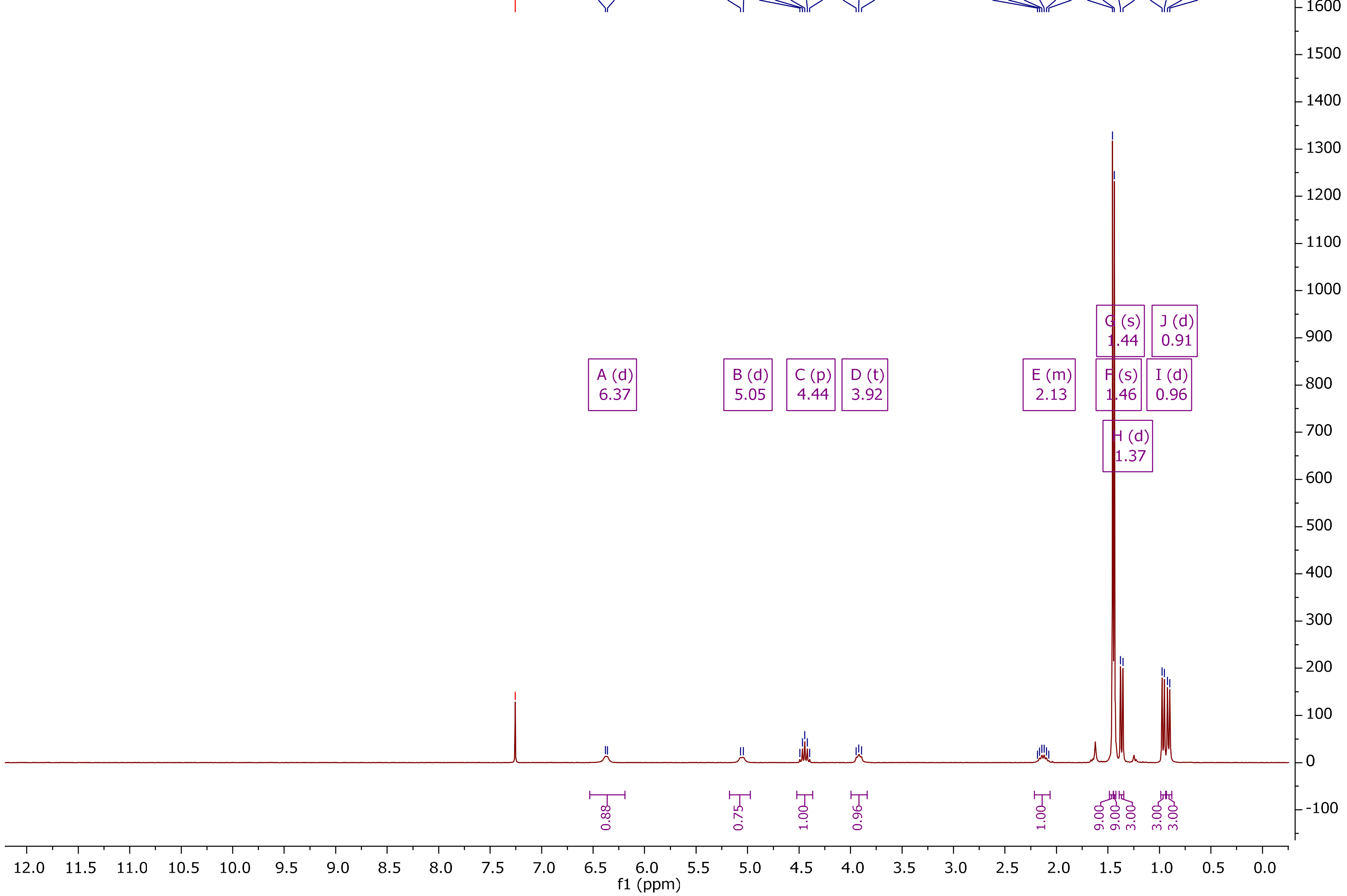

10e $^{13}C\{^{1}H\}$ NMR (75 MHz, Chloroform-*d*)

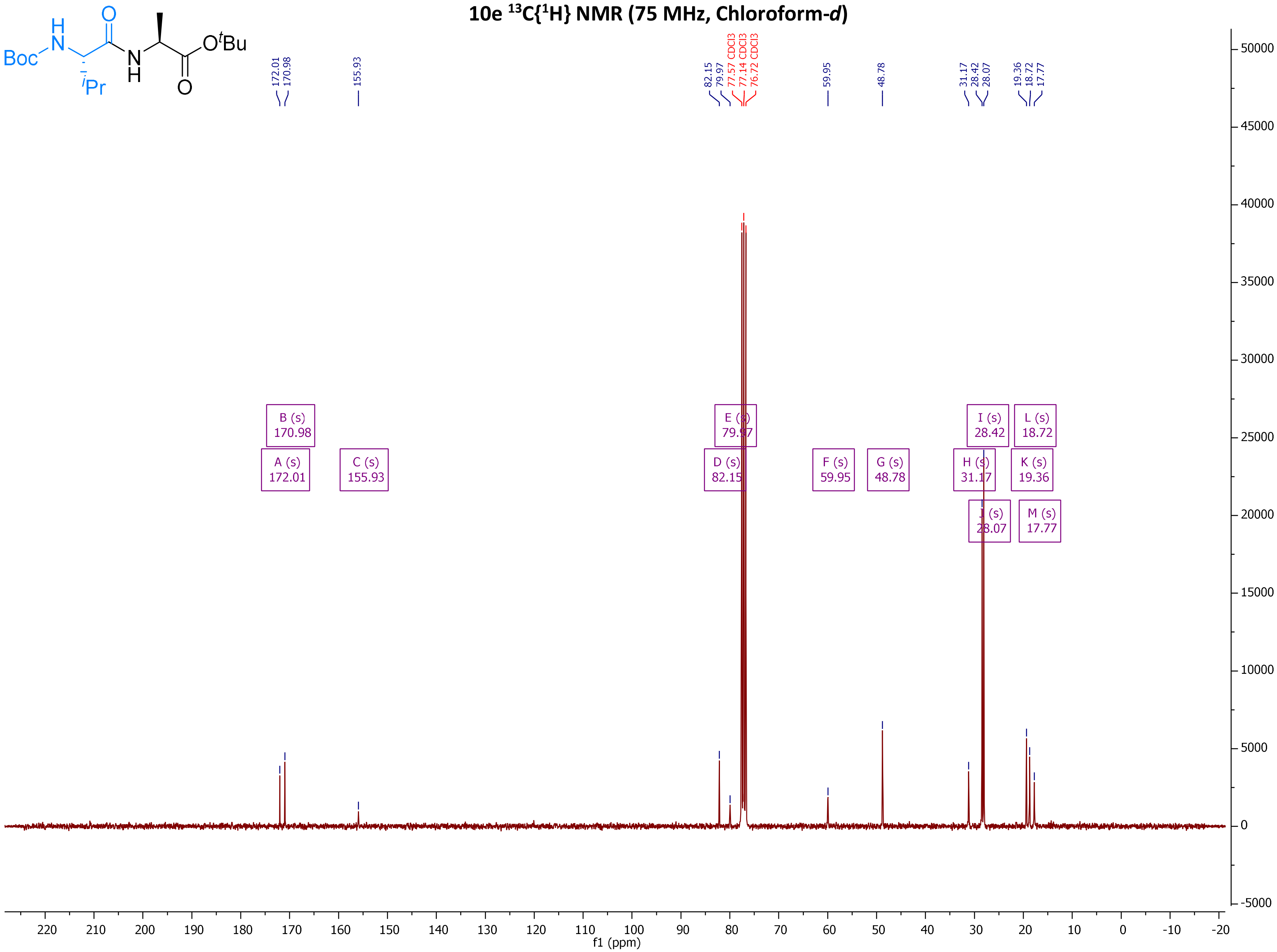

10f ^{1}H NMR (300 MHz, Chloroform-*d*)

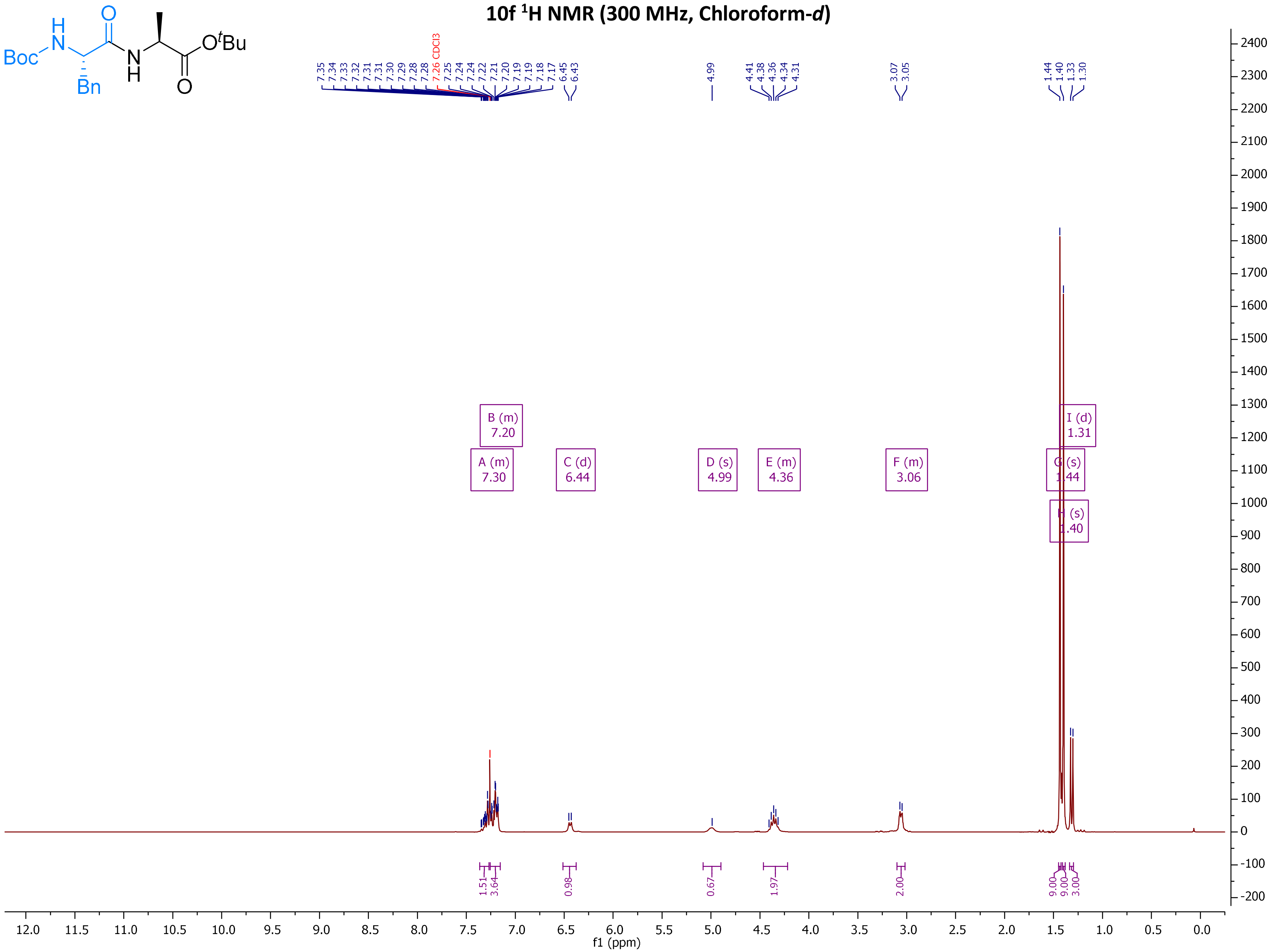

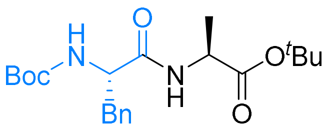

10f $^{13}C\{^{1}H\}$ NMR (75 MHz, Chloroform-*d*)

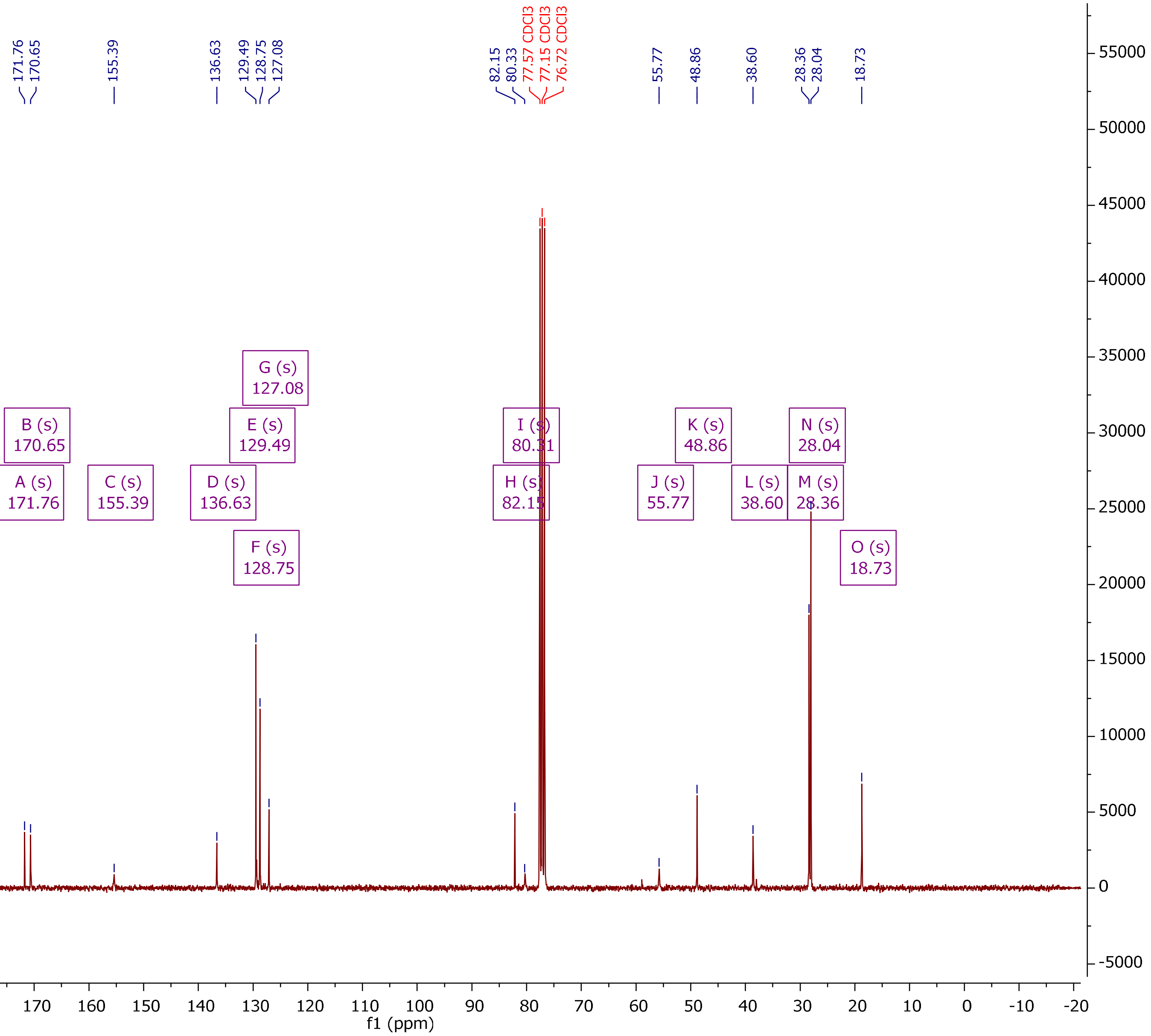

10g ^{1}H NMR (300 MHz, Chloroform-*d*)

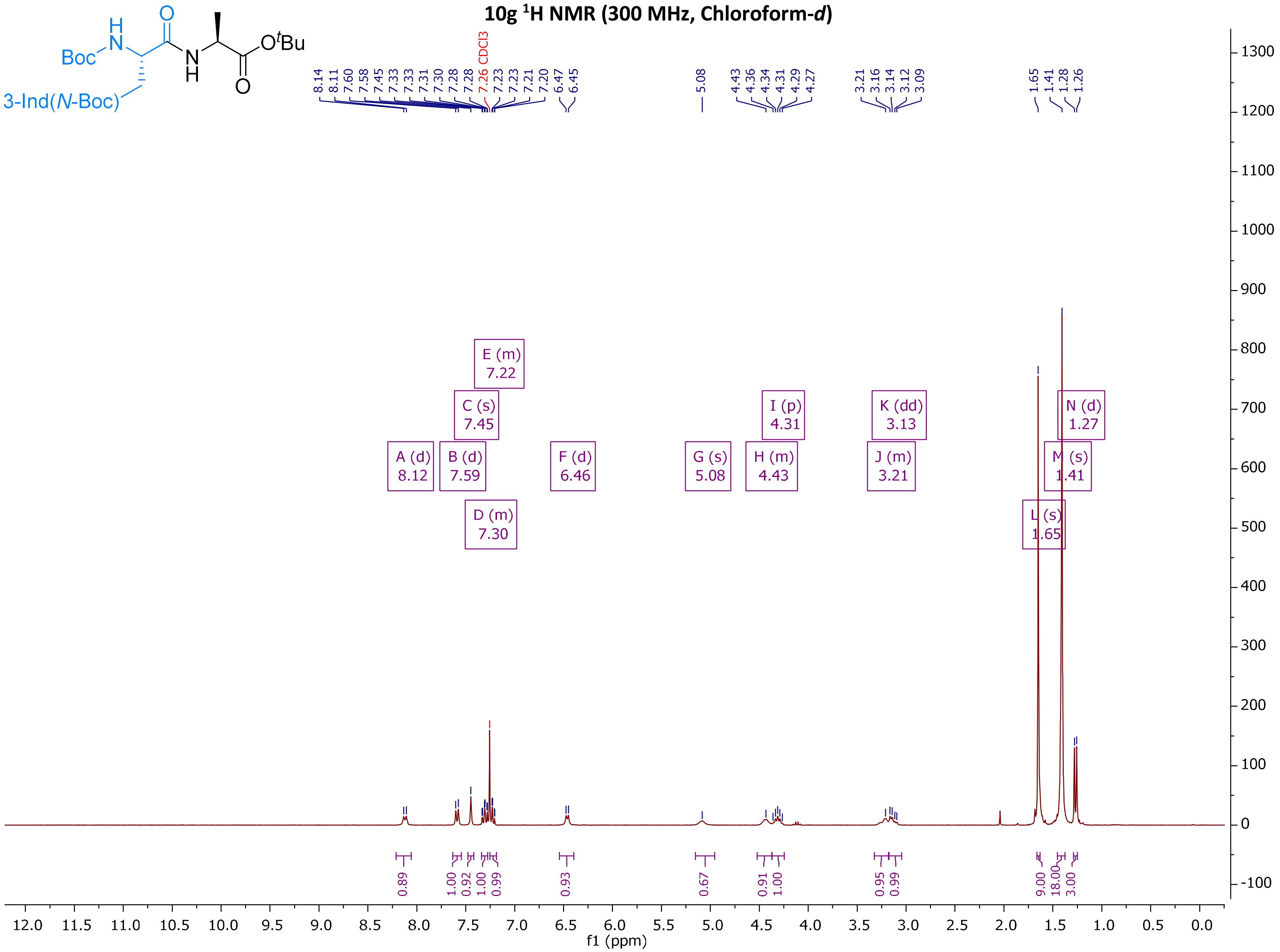

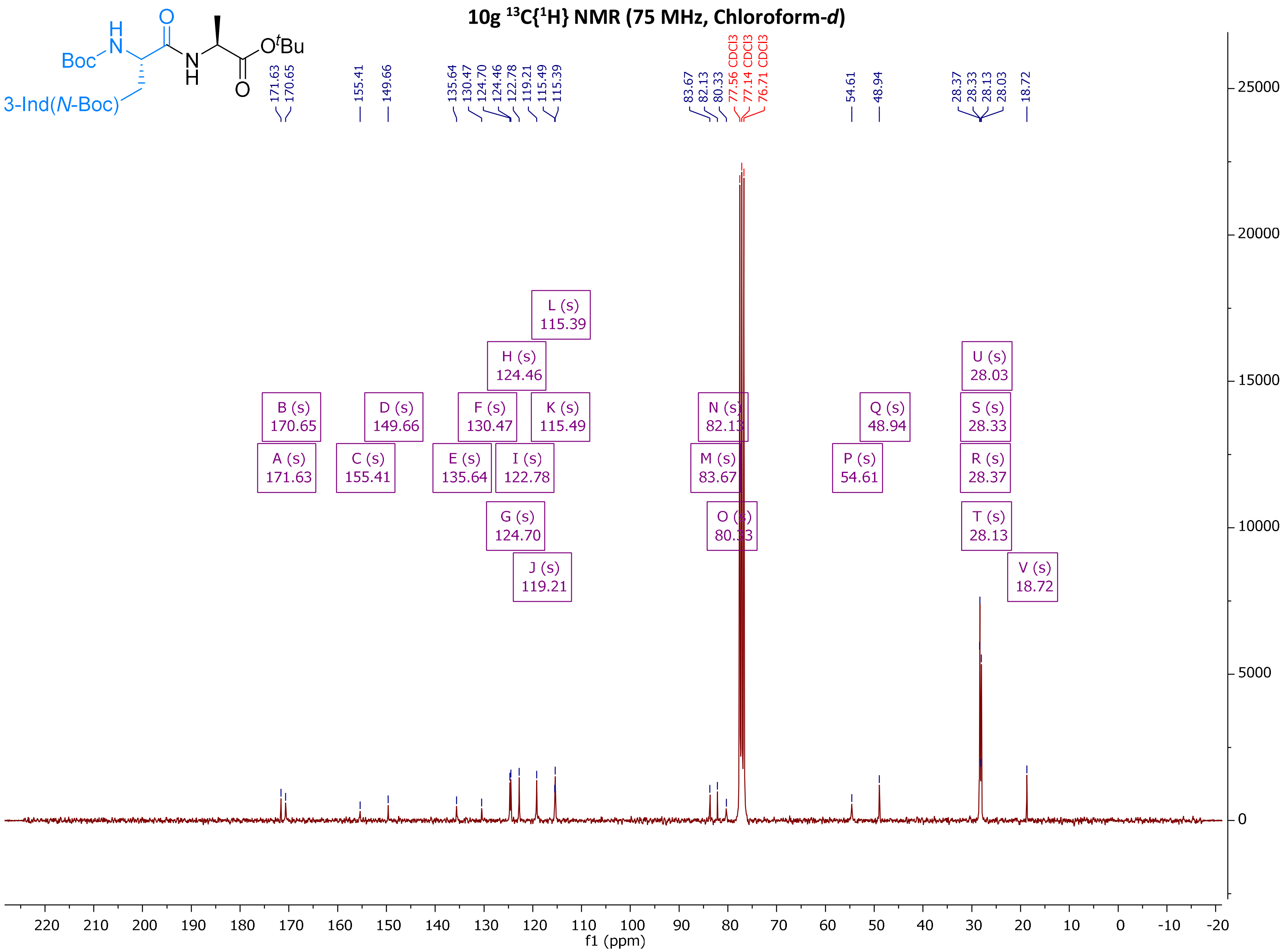
10g $^{13}C\{^1H\}$ NMR (75 MHz, Chloroform-d)
Boc
H
N
O
N
H
O
O^{t}Bu
3-Ind(N-Boc)
171.63
170.65
155.41
149.66
135.64
130.47
124.70
124.46
122.78
119.21
115.49
115.39
83.67
82.13
80.33
77.56 CDCl3
77.14 CDCl3
76.71 CDCl3
54.61
48.94
28.37
28.33
28.13
28.03
18.72
A (s) 171.63
B (s) 170.65
C (s) 155.41
D (s) 149.66
E (s) 135.64
F (s) 130.47
G (s) 124.70
H (s) 124.46
I (s) 122.78
J (s) 119.21
K (s) 115.49
L (s) 115.39
M (s) 83.67
N (s) 82.13
O (s) 80.33
P (s) 54.61
Q (s) 48.94
R (s) 28.37
S (s) 28.33
T (s) 28.13
U (s) 28.03
V (s) 18.72
25000
20000
15000
10000
5000
0
220 210 200 190 180 170 160 150 140 130 120 110 100 90 80 70 60 50 40 30 20 10 0 -10 -20
f1 (ppm)

10h ¹H NMR (300 MHz, Chloroform-*d*)

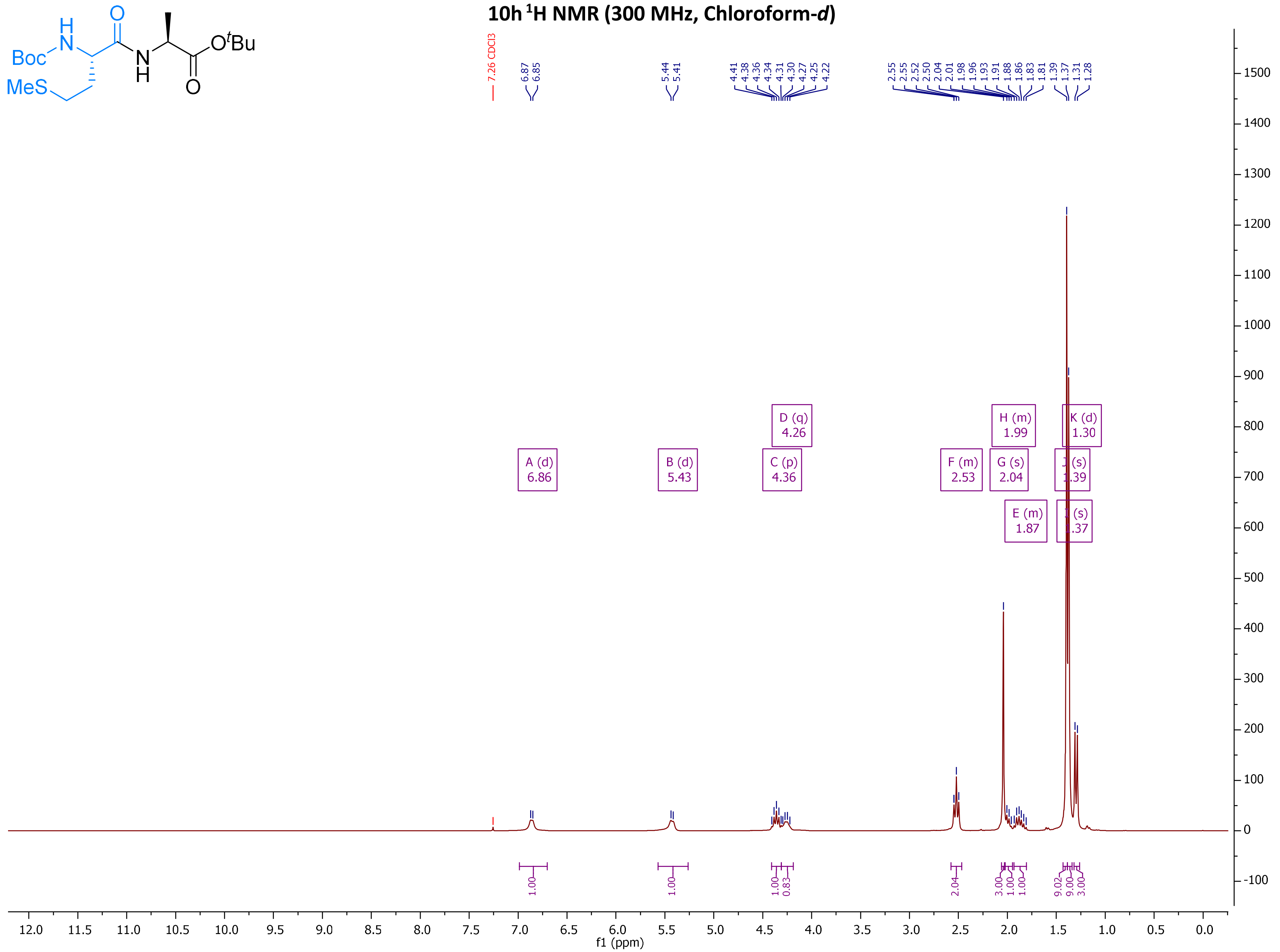

10h $^{13}C\{^1H\}$ NMR (75 MHz, Chloroform-*d*)

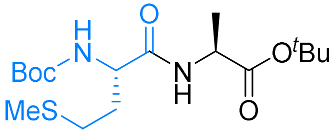

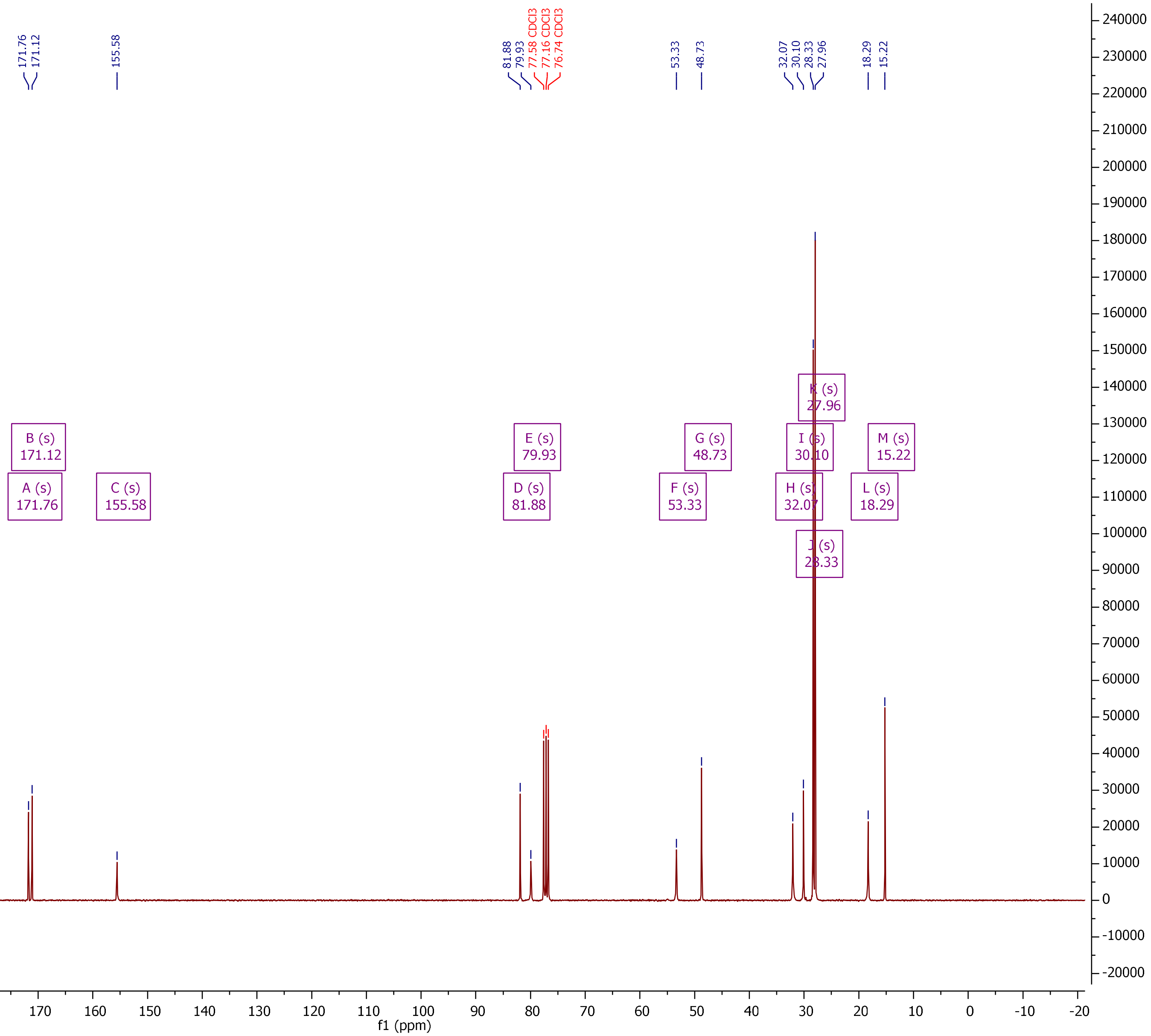

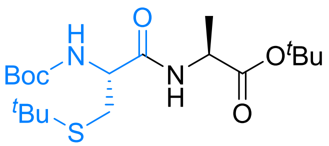

10i ^{1}H NMR (300 MHz, Chloroform-*d*)

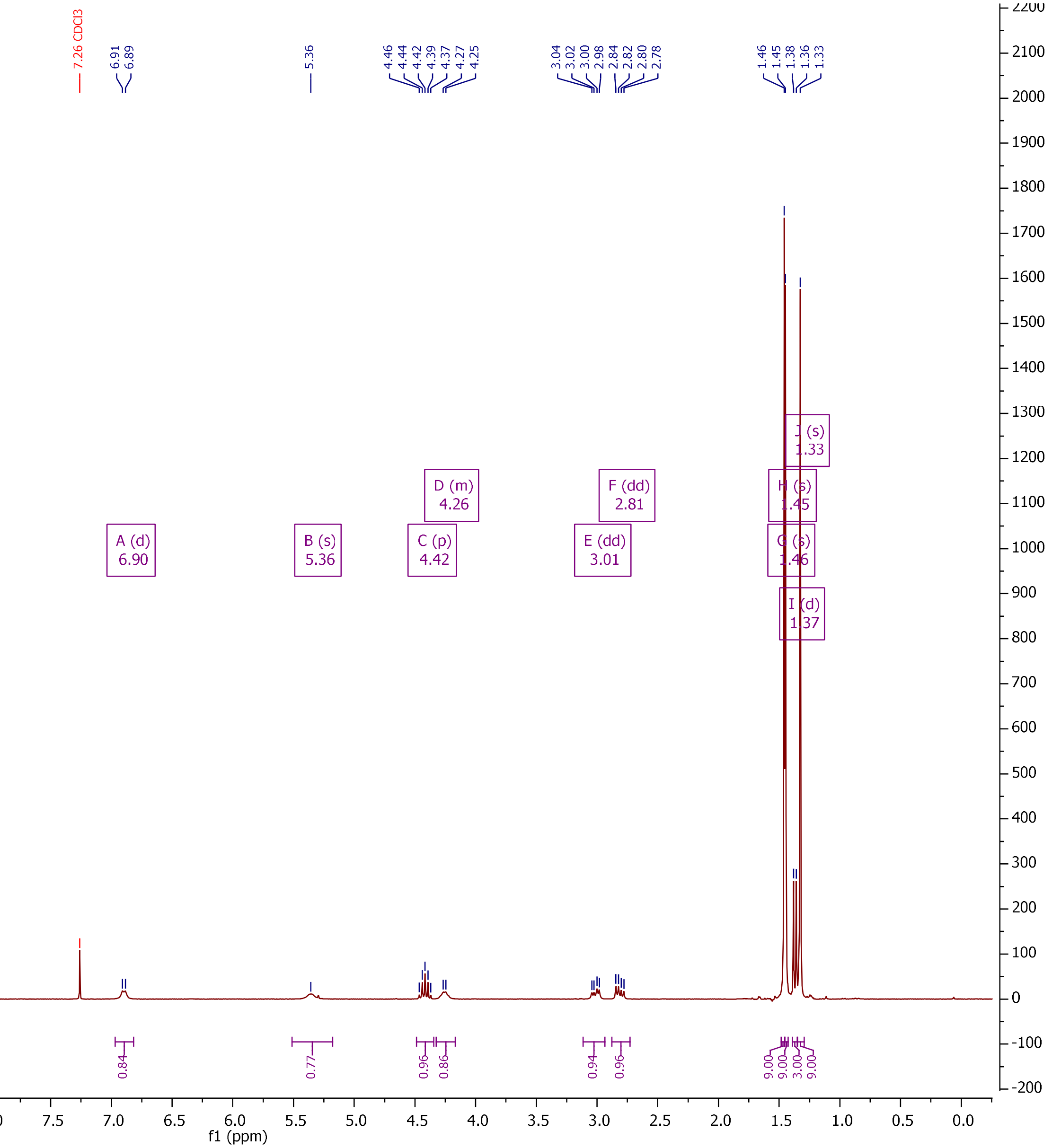

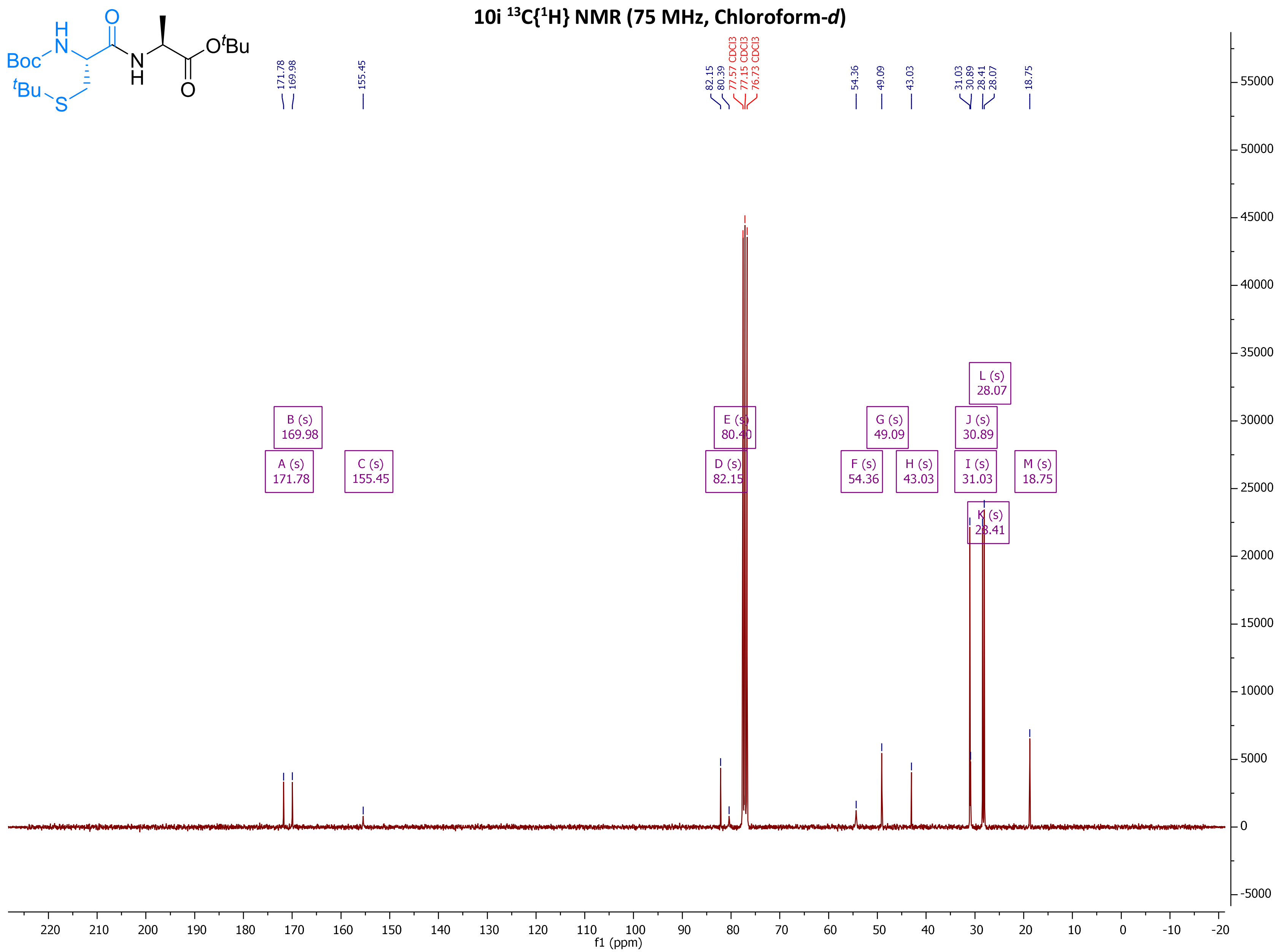
10i $^{13}C\{^1H\}$ NMR (75 MHz, Chloroform-*d*)
Boc
H
N
O
N
H
O^{t}Bu
O
^{t}Bu
S
171.78
169.98
155.45
82.15
80.39
77.57 CDCl3
77.15 CDCl3
76.73 CDCl3
54.36
49.09
43.03
31.03
30.89
28.41
28.07
18.75
A (s) 171.78
B (s) 169.98
C (s) 155.45
D (s) 82.15
E (s) 80.40
F (s) 54.36
G (s) 49.09
H (s) 43.03
I (s) 31.03
J (s) 30.89
K (s) 28.41
L (s) 28.07
M (s) 18.75
f1 (ppm)

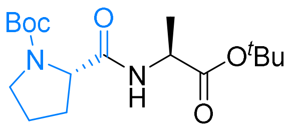

10j ^{1}H NMR (300 MHz, Chloroform-*d*)

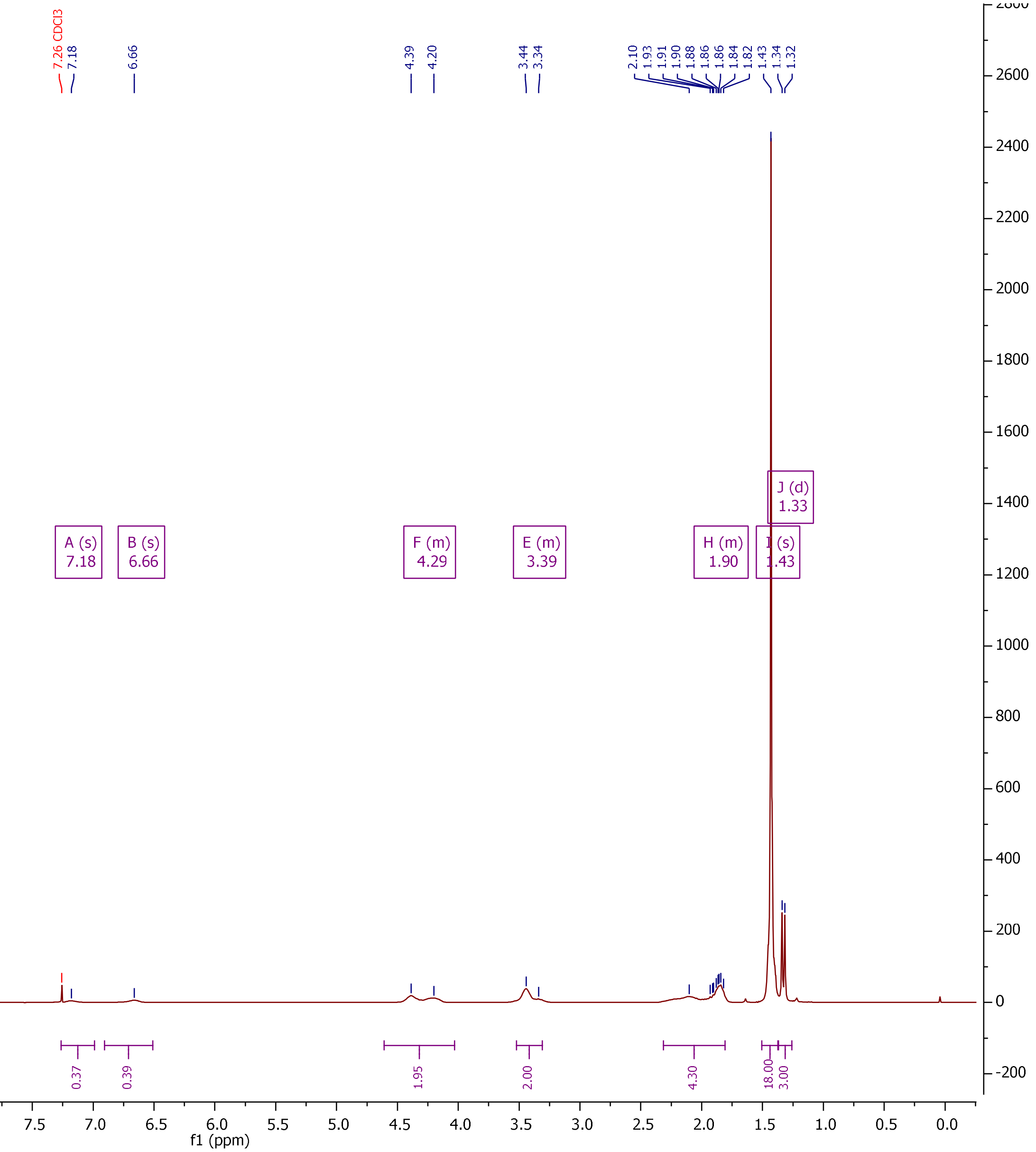

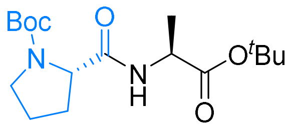

10j $^{13}C\{^{1}H\}$ NMR (75 MHz, Chloroform-*d*)

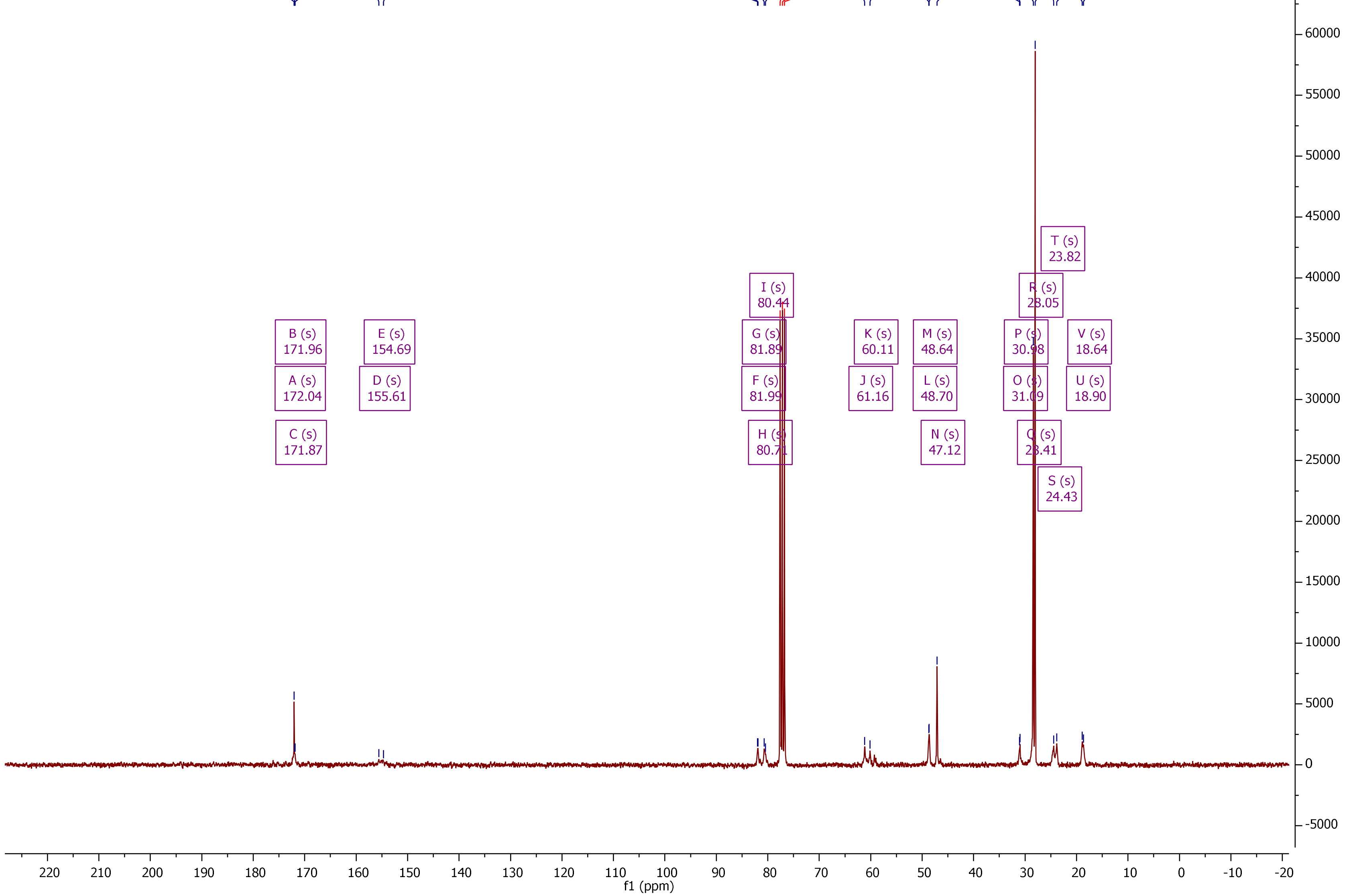

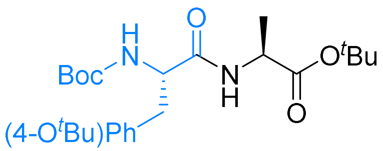

10k ^{1}H NMR (300 MHz, Chloroform-*d*)

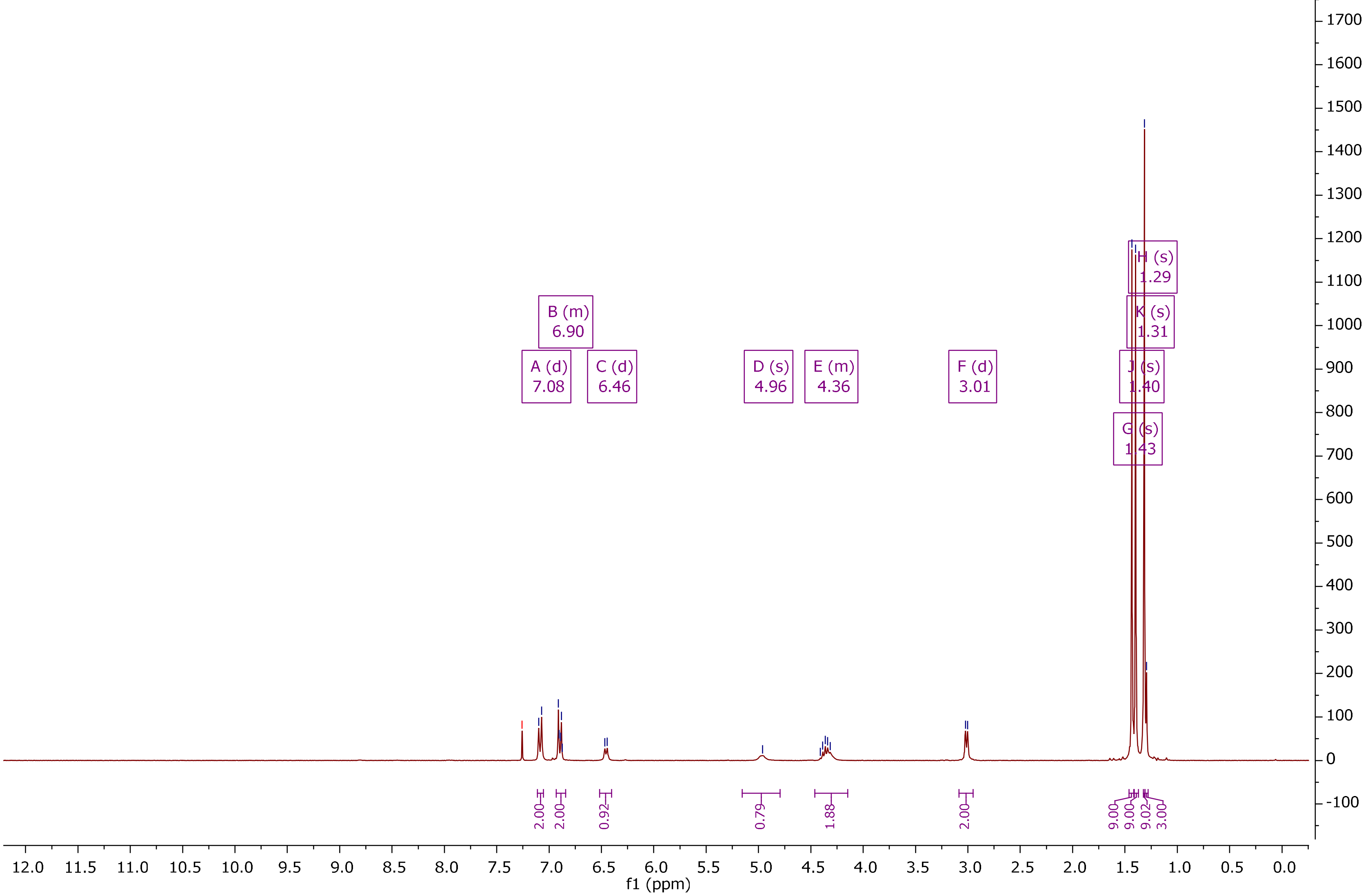

10k $^{13}C\{^{1}H\}$ NMR (75 MHz, Chloroform-*d*)

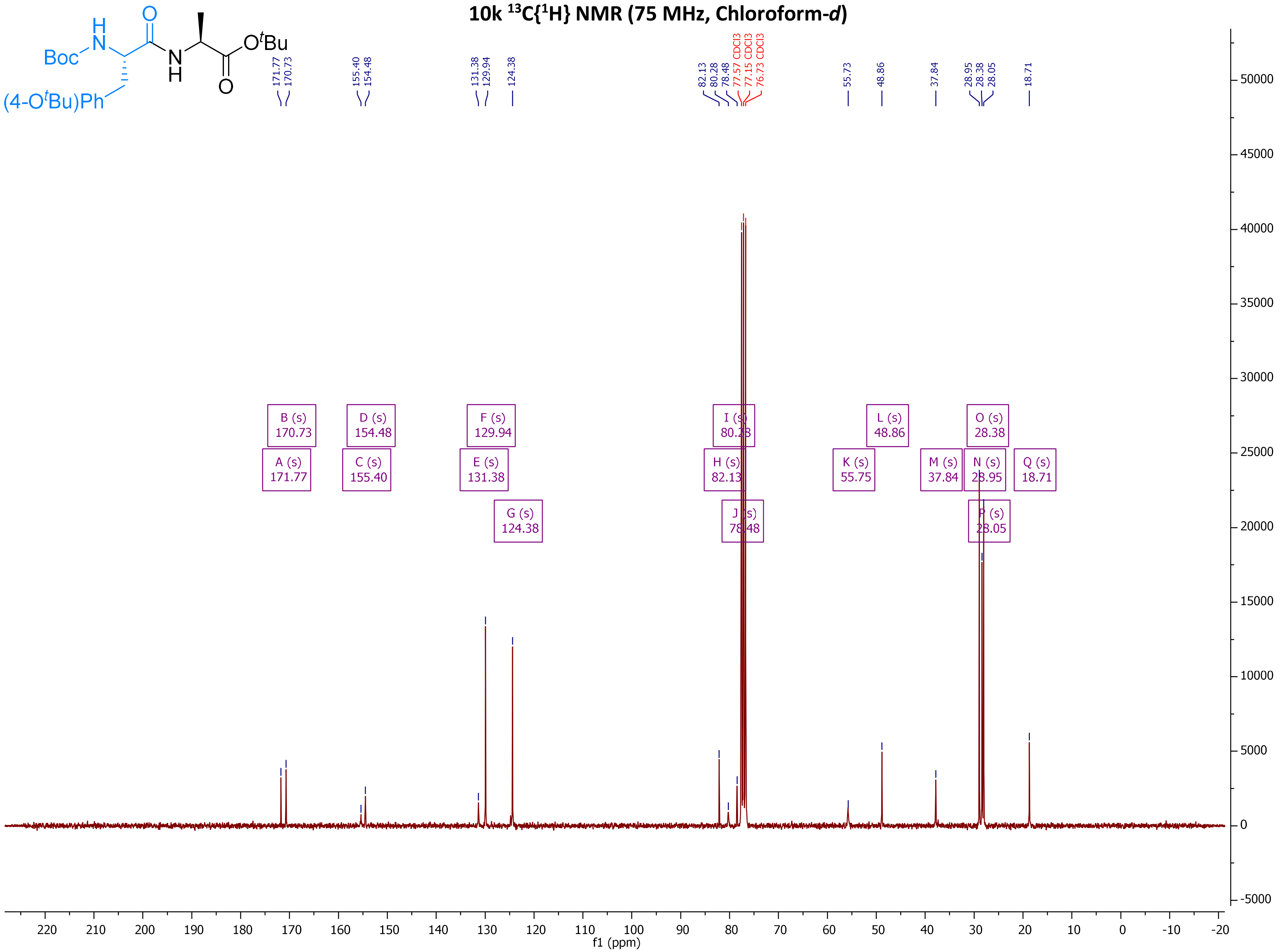

10l ^{1}H NMR (300 MHz, Chloroform-*d*)

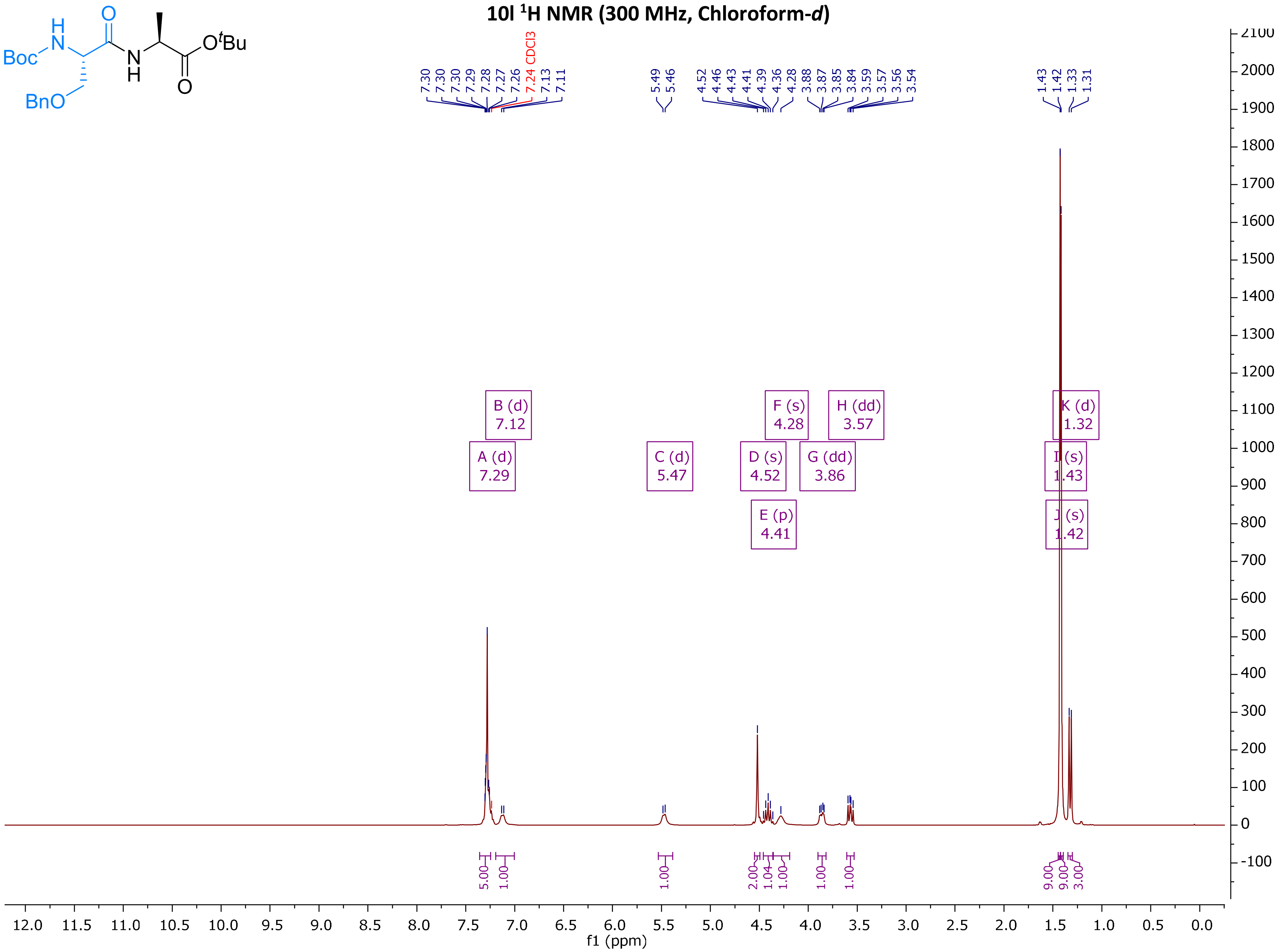

10l $^{13}C\{^{1}H\}$ NMR (75 MHz, Chloroform-*d*)

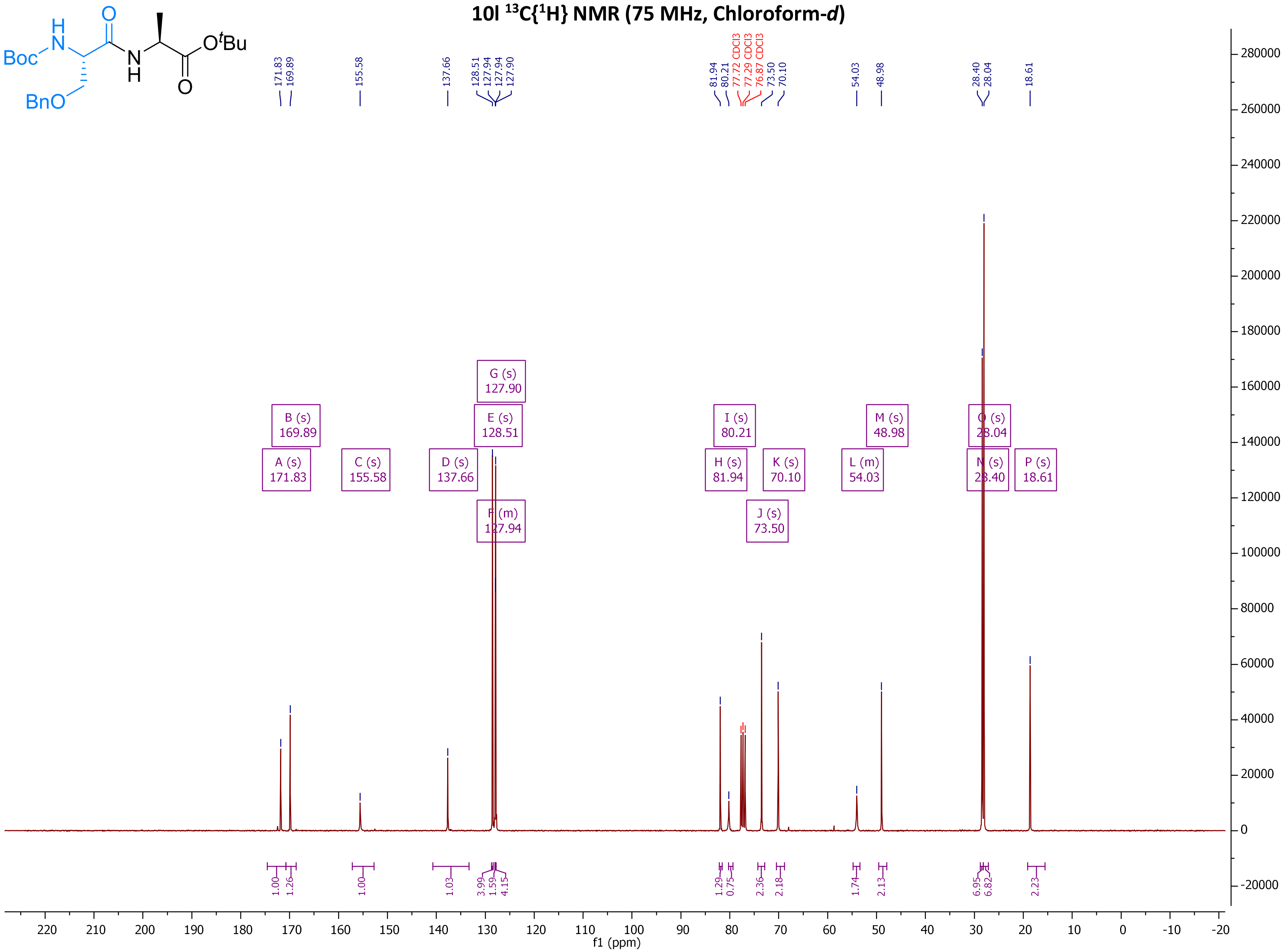

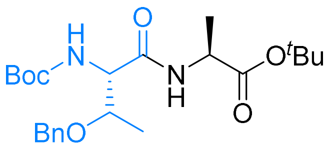

10m ^{1}H NMR (300 MHz, Chloroform-*d*)

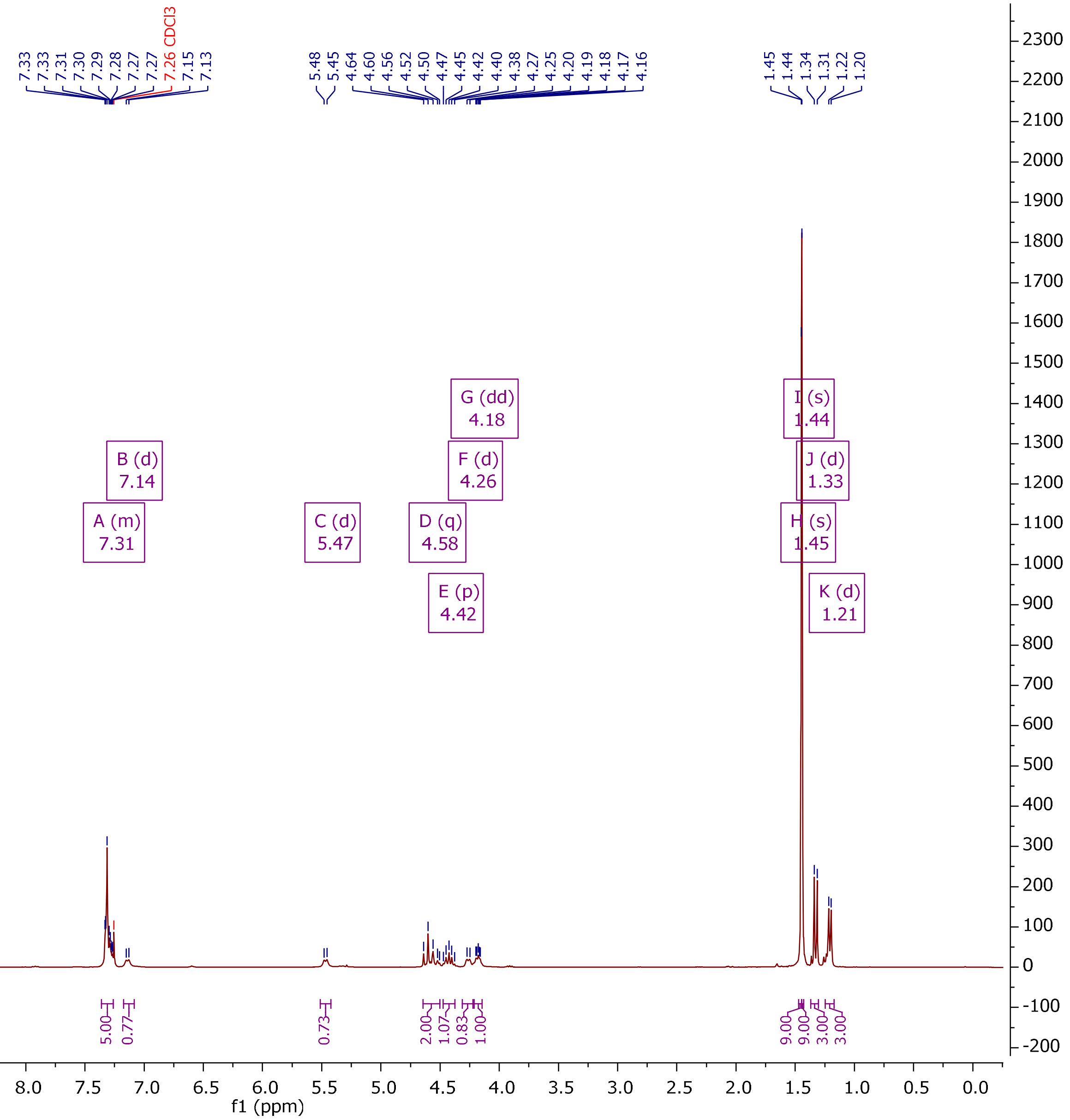

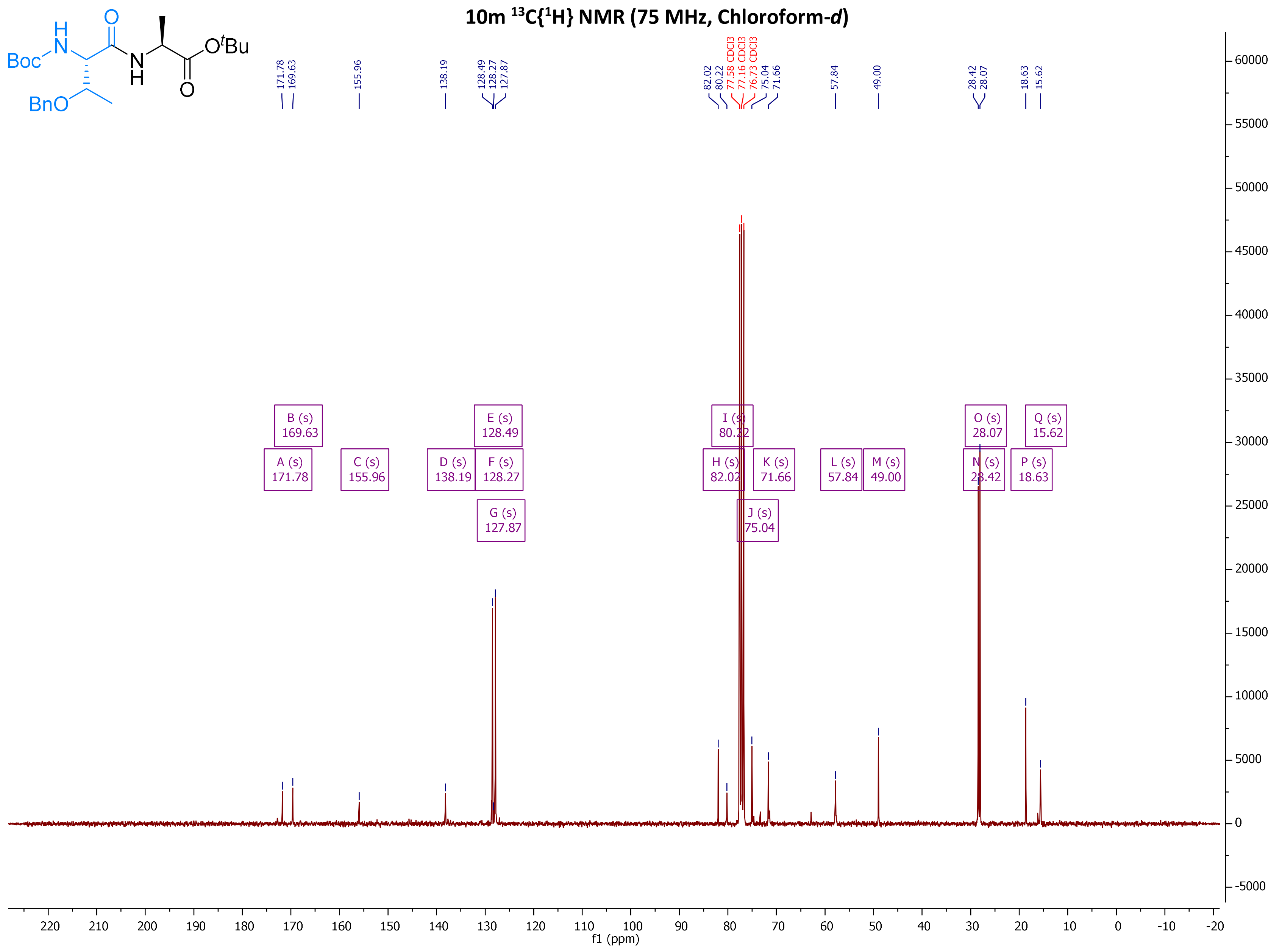
10m ¹³C{¹H} NMR (75 MHz, Chloroform-d)
Boc
BnO
O^{t}Bu
171.78
169.63
155.96
138.19
128.49
128.27
127.87
82.02
80.22
77.58 CDCl3
77.16 CDCl3
76.73 CDCl3
75.04
71.66
57.84
49.00
28.42
28.07
18.63
15.62
A (s) 171.78
B (s) 169.63
C (s) 155.96
D (s) 138.19
E (s) 128.49
F (s) 128.27
G (s) 127.87
H (s) 82.02
I (s) 80.22
J (s) 75.04
K (s) 71.66
L (s) 57.84
M (s) 49.00
N (s) 28.42
O (s) 28.07
P (s) 18.63
Q (s) 15.62
f1 (ppm)

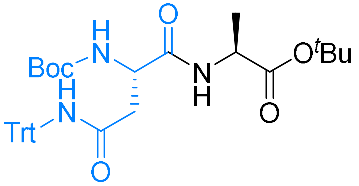

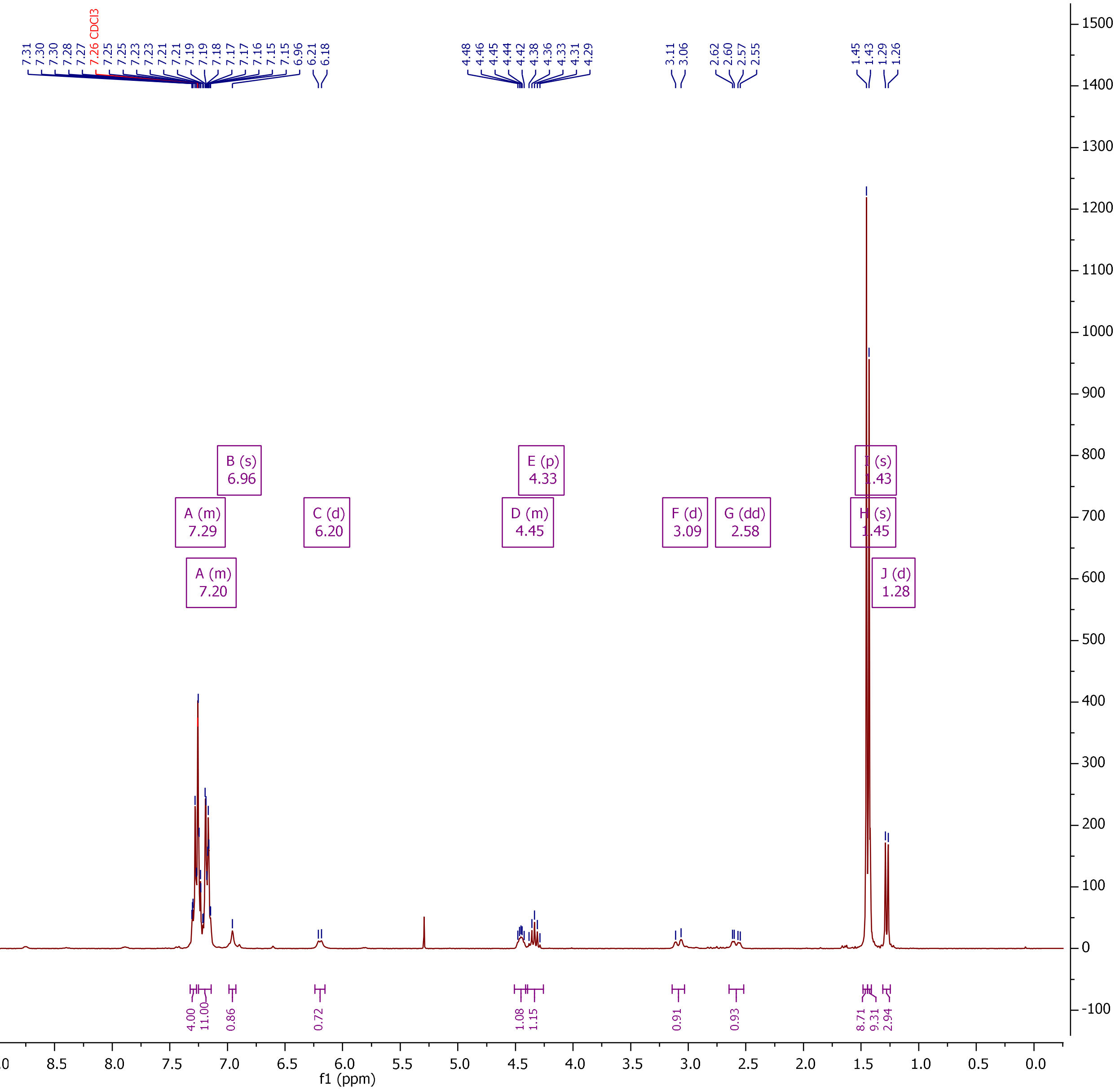
10n ¹H NMR (300 MHz, Chloroform-d)
7.31
7.30
7.30
7.28
7.27
7.26 CDCl3
7.25
7.25
7.23
7.23
7.21
7.21
7.19
7.19
7.18
7.17
7.17
7.16
7.15
7.15
6.96
6.21
6.18
4.48
4.46
4.45
4.44
4.42
4.38
4.36
4.33
4.31
4.29
3.11
3.06
2.62
2.60
2.57
2.55
1.45
1.43
1.29
1.26
B (s)
6.96
E (p)
4.33
I (s)
1.43
A (m)
7.29
C (d)
6.20
D (m)
4.45
F (d)
3.09
G (dd)
2.58
H (s)
1.45
A (m)
7.20
J (d)
1.28
4.00
11.00
0.86
0.72
1.08
1.15
0.91
0.93
8.71
9.31
2.94
f1 (ppm)

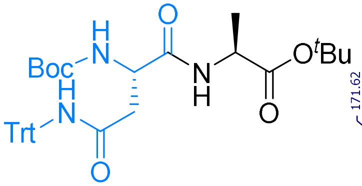

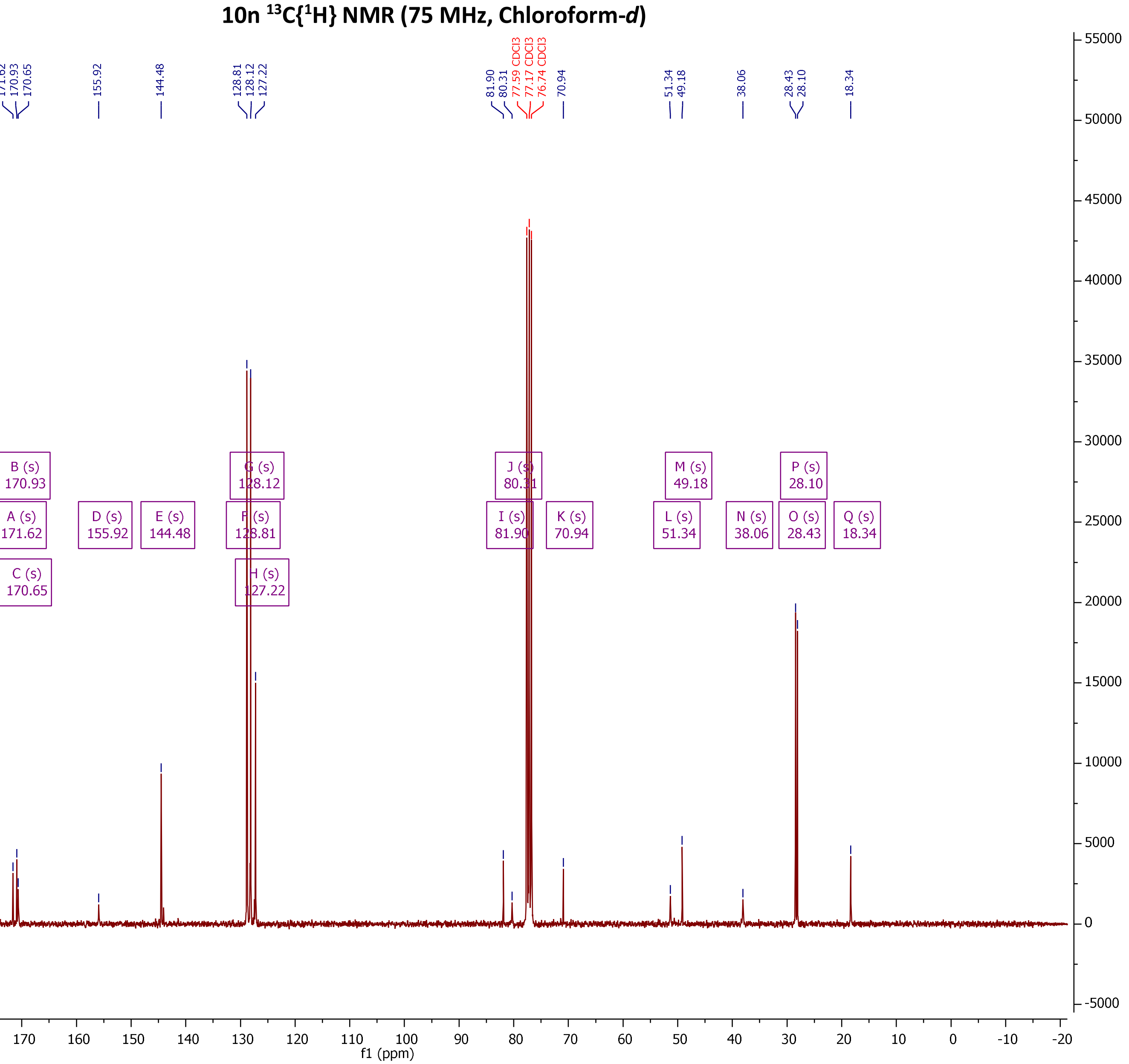

10n $^{13}C\{^1H\}$ NMR (75 MHz, Chloroform-*d*)
171.62
170.93
170.65
155.92
144.48
128.81
128.12
127.22
81.90
80.31
77.59 CDCl3
77.17 CDCl3
76.74 CDCl3
70.94
51.34
49.18
38.06
28.43
28.10
18.34
B (s) 170.93
A (s) 171.62
C (s) 170.65
D (s) 155.92
E (s) 144.48
G (s) 128.12
F (s) 128.81
H (s) 127.22
J (s) 80.31
I (s) 81.90
K (s) 70.94
M (s) 49.18
L (s) 51.34
N (s) 38.06
P (s) 28.10
O (s) 28.43
Q (s) 18.34
f1 (ppm)

10o ^{1}H NMR (300 MHz, Chloroform-*d*)

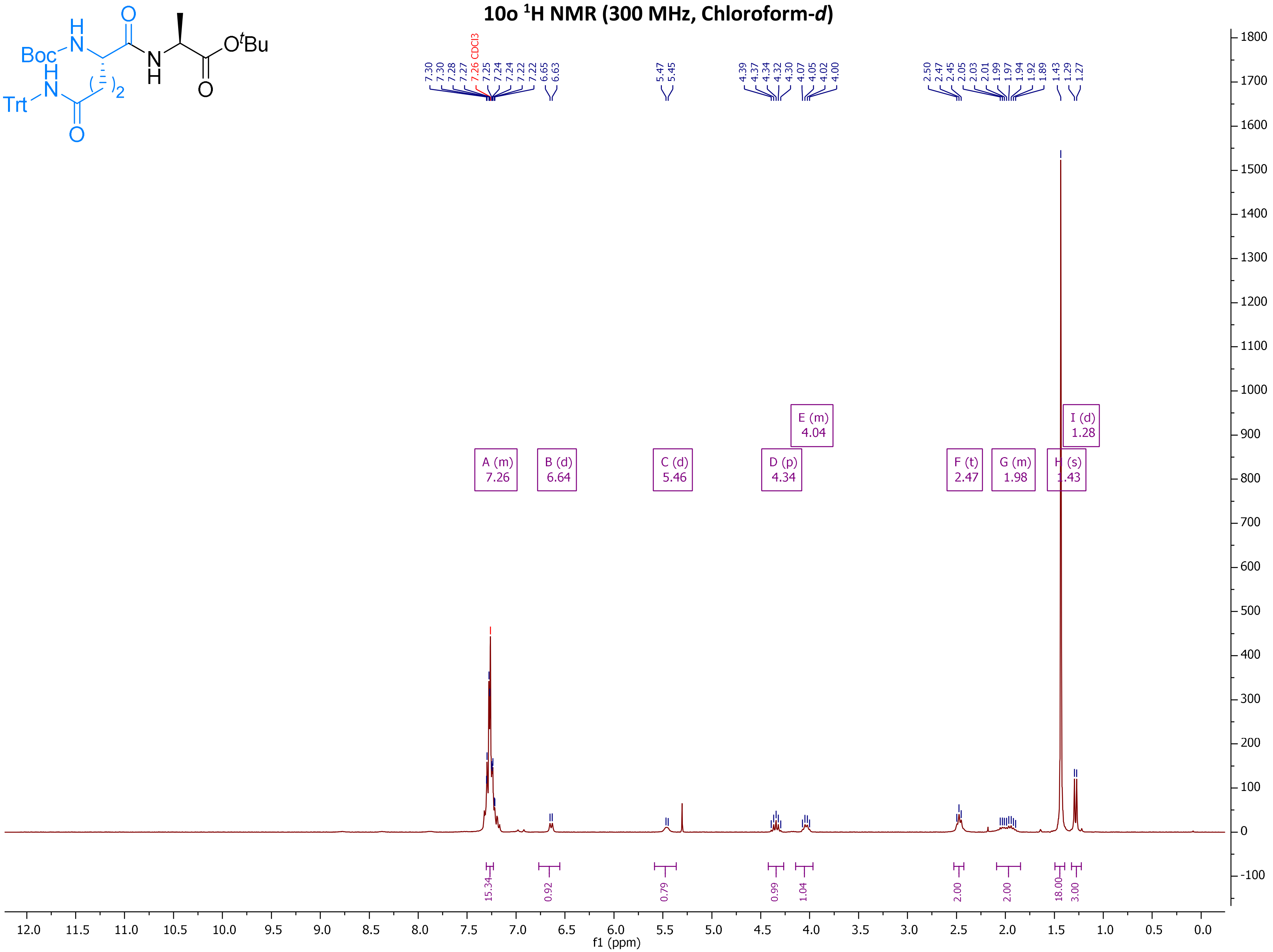

10o $^{13}C\{^1H\}$ NMR (75 MHz, Chloroform-*d*)

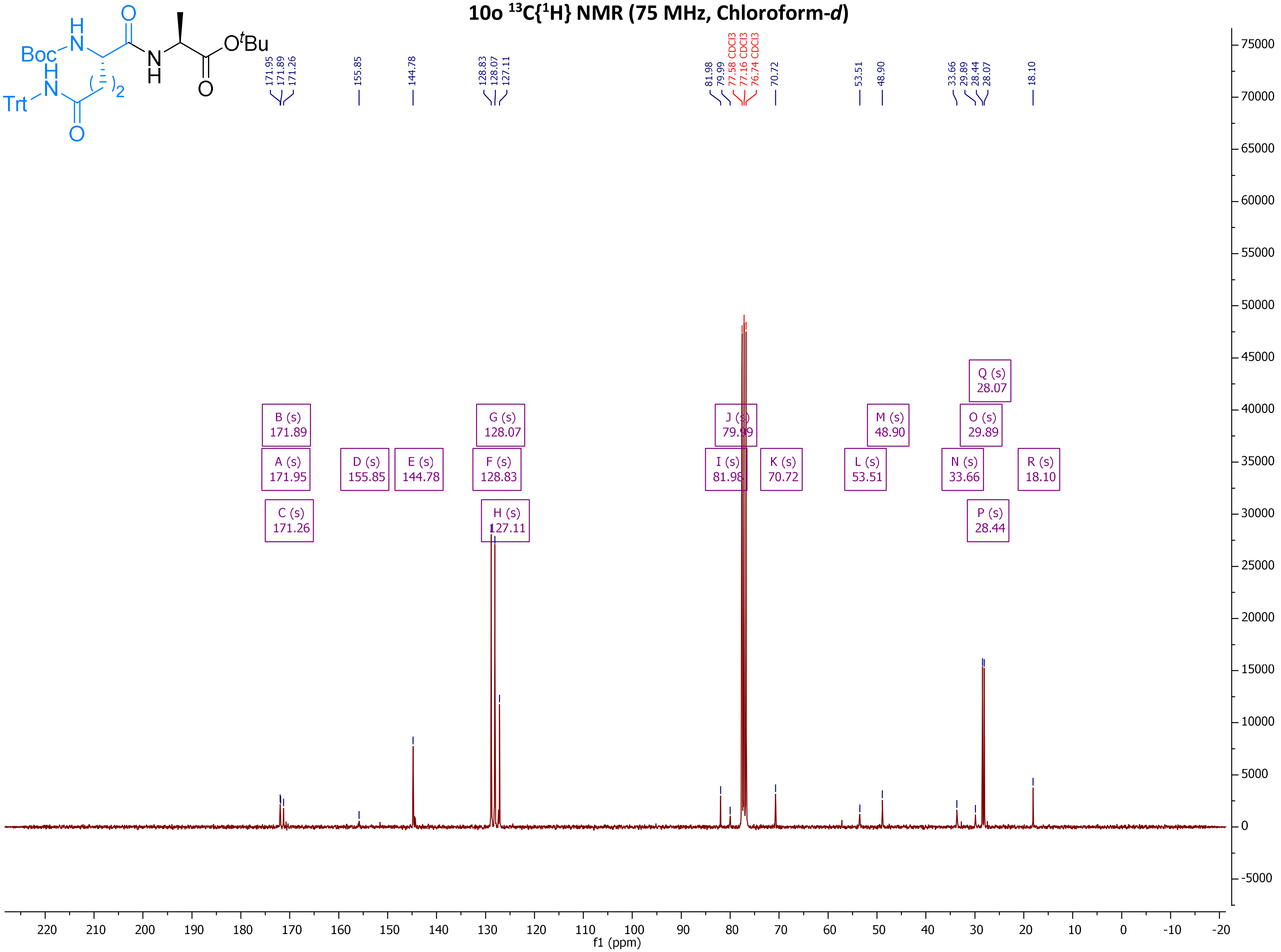

10p ^{1}H NMR (300 MHz, Chloroform-*d*)

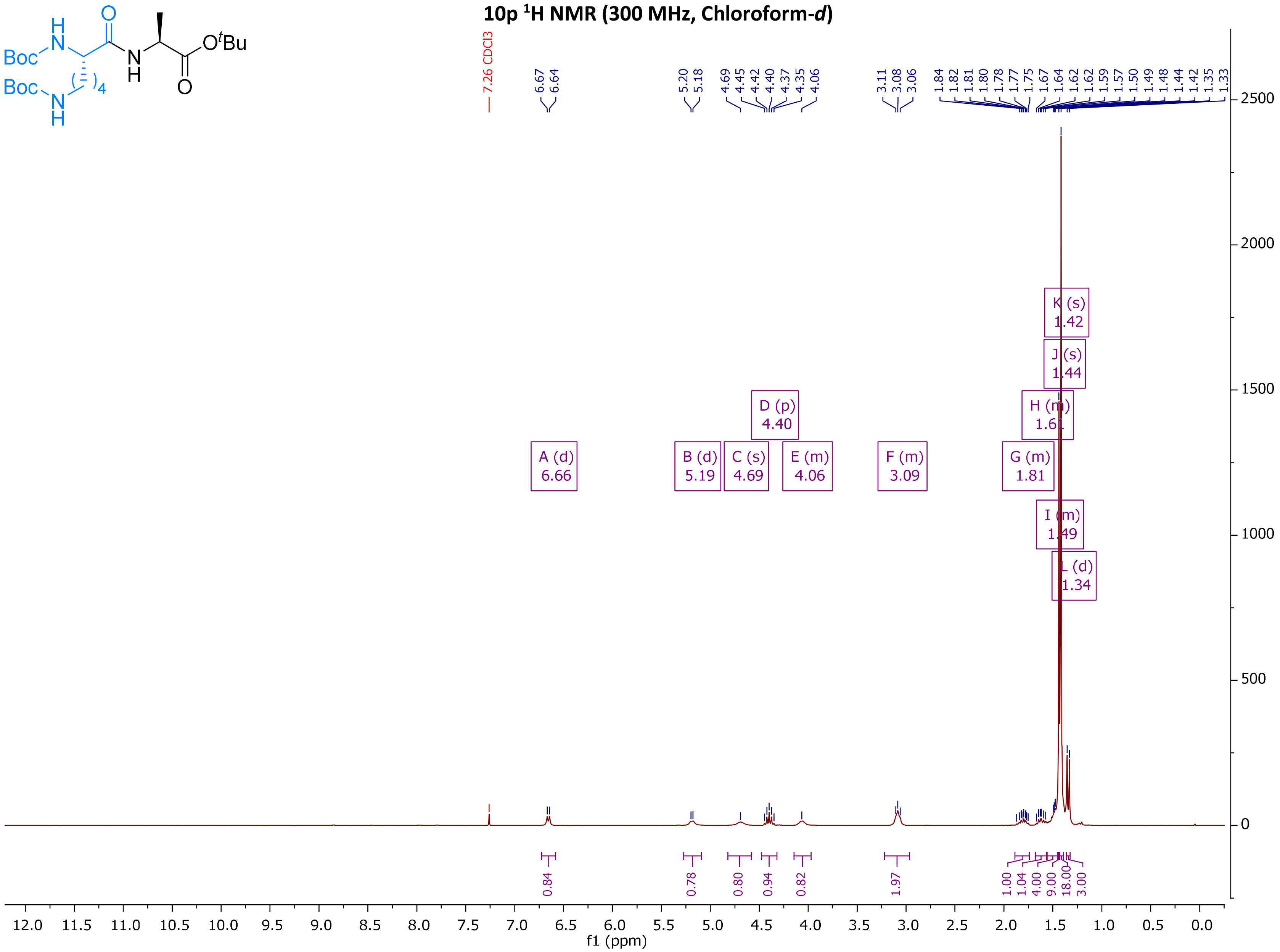

10p $^{13}C\{^1H\}$ NMR (75 MHz, Chloroform-*d*)

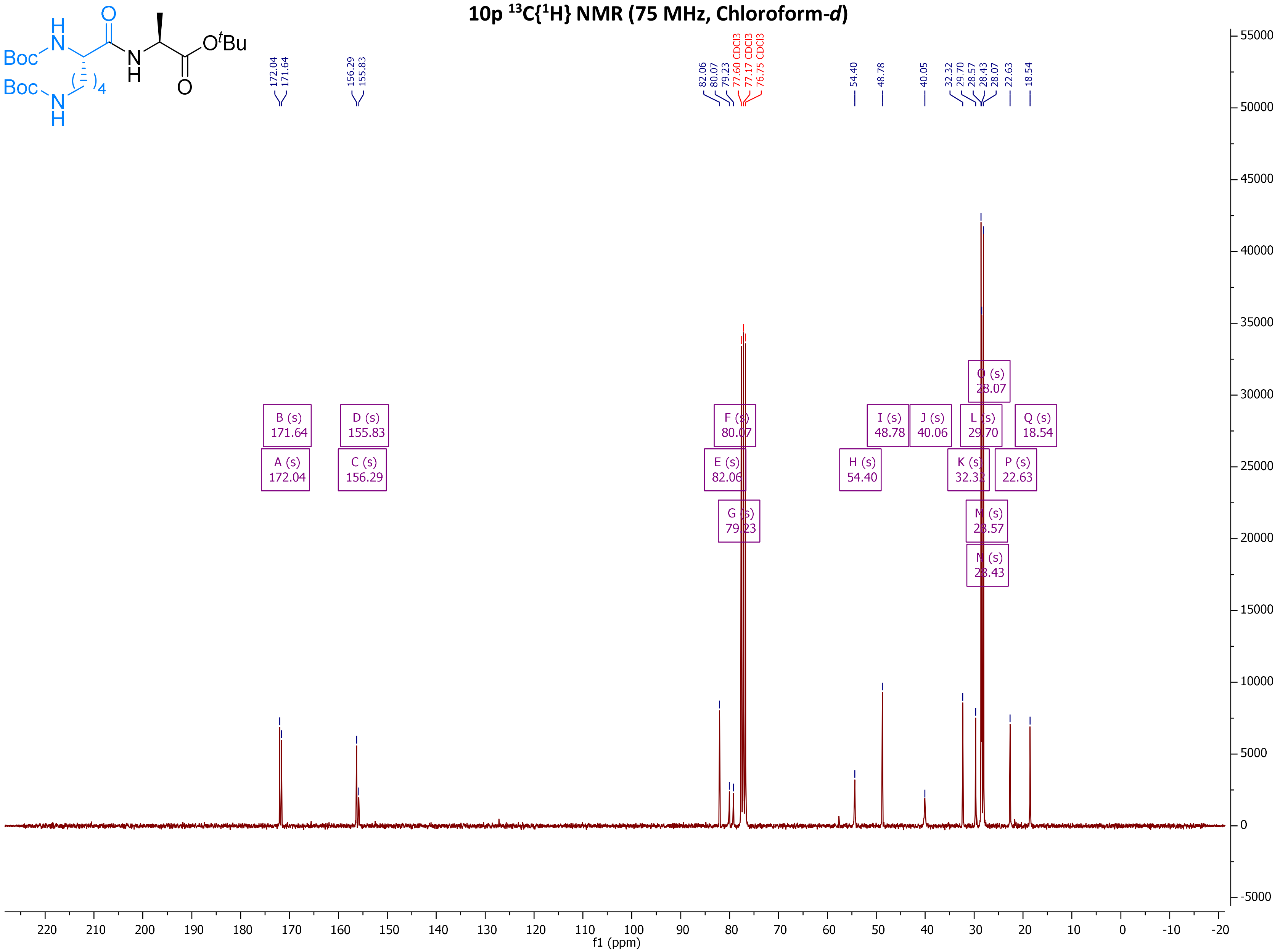

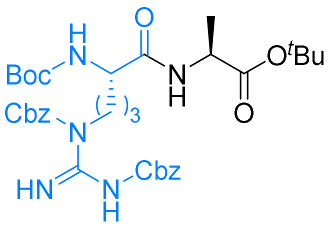

10q ^{1}H NMR (300 MHz, Chloroform-*d*)

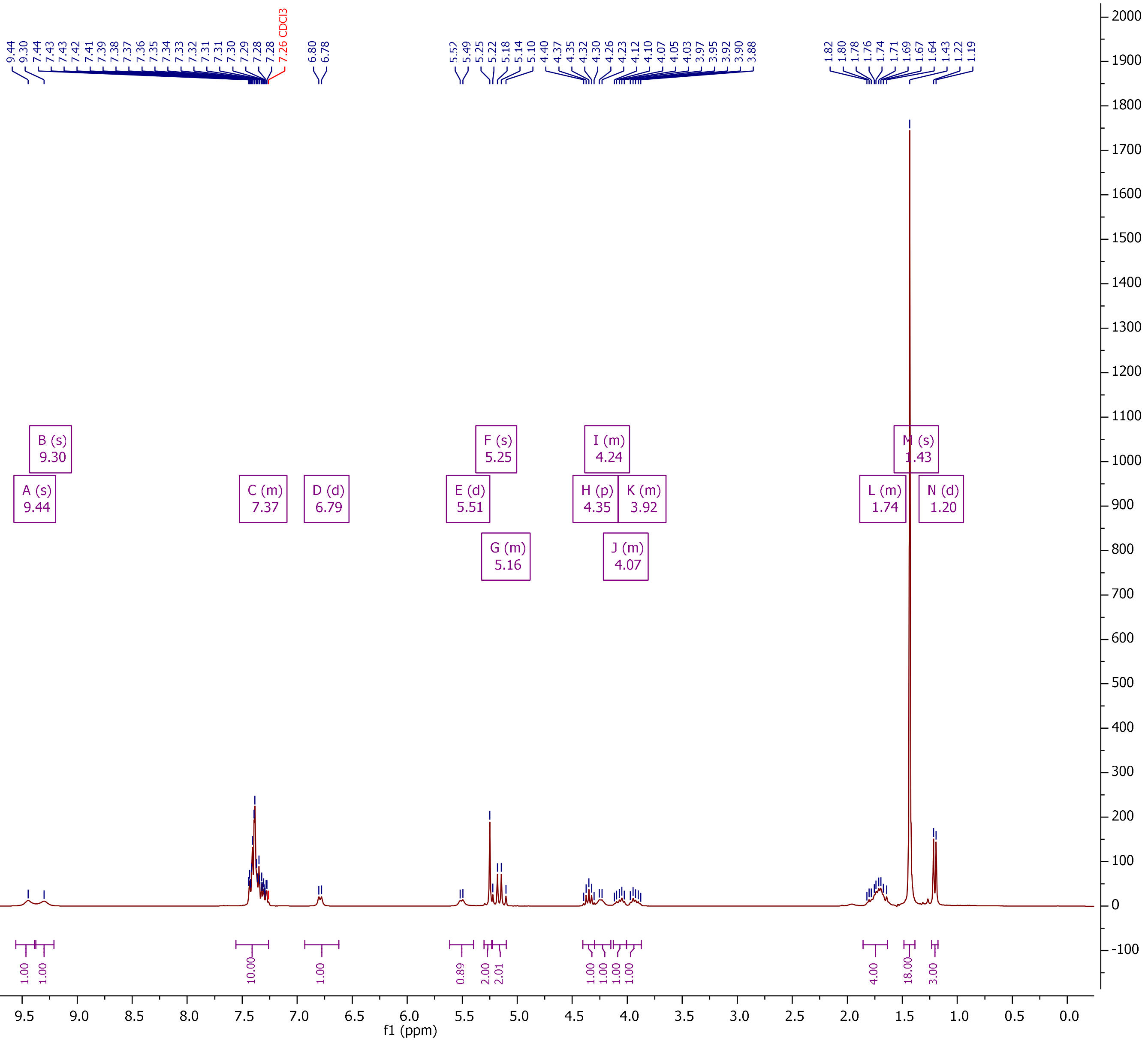

10q $^{13}C\{^1H\}$ NMR (75 MHz, Chloroform-*d*)

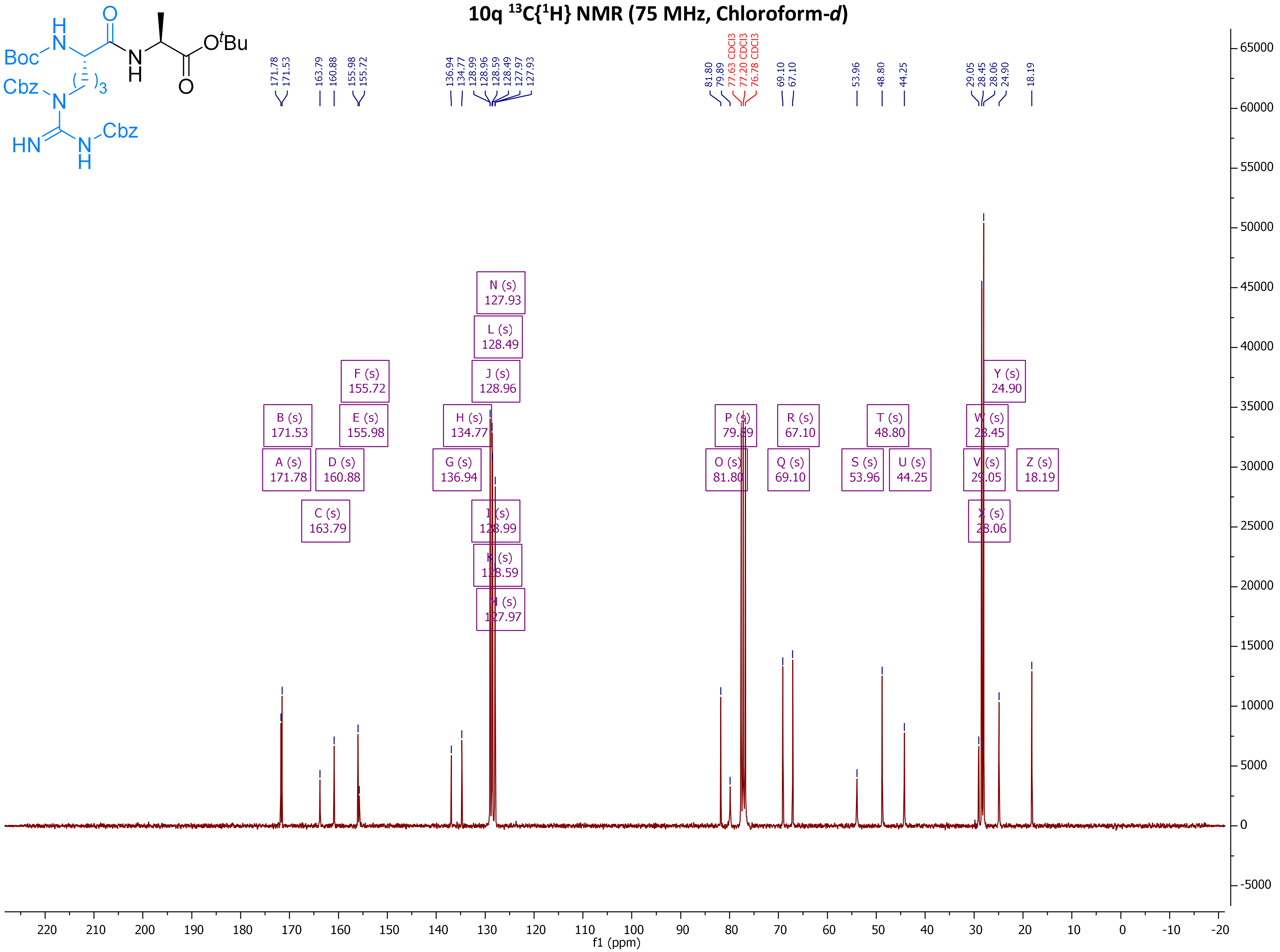

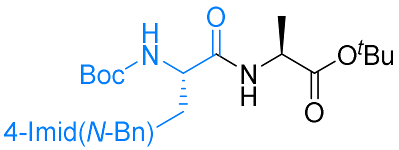

10r ^{1}H NMR (300 MHz, Chloroform-*d*)

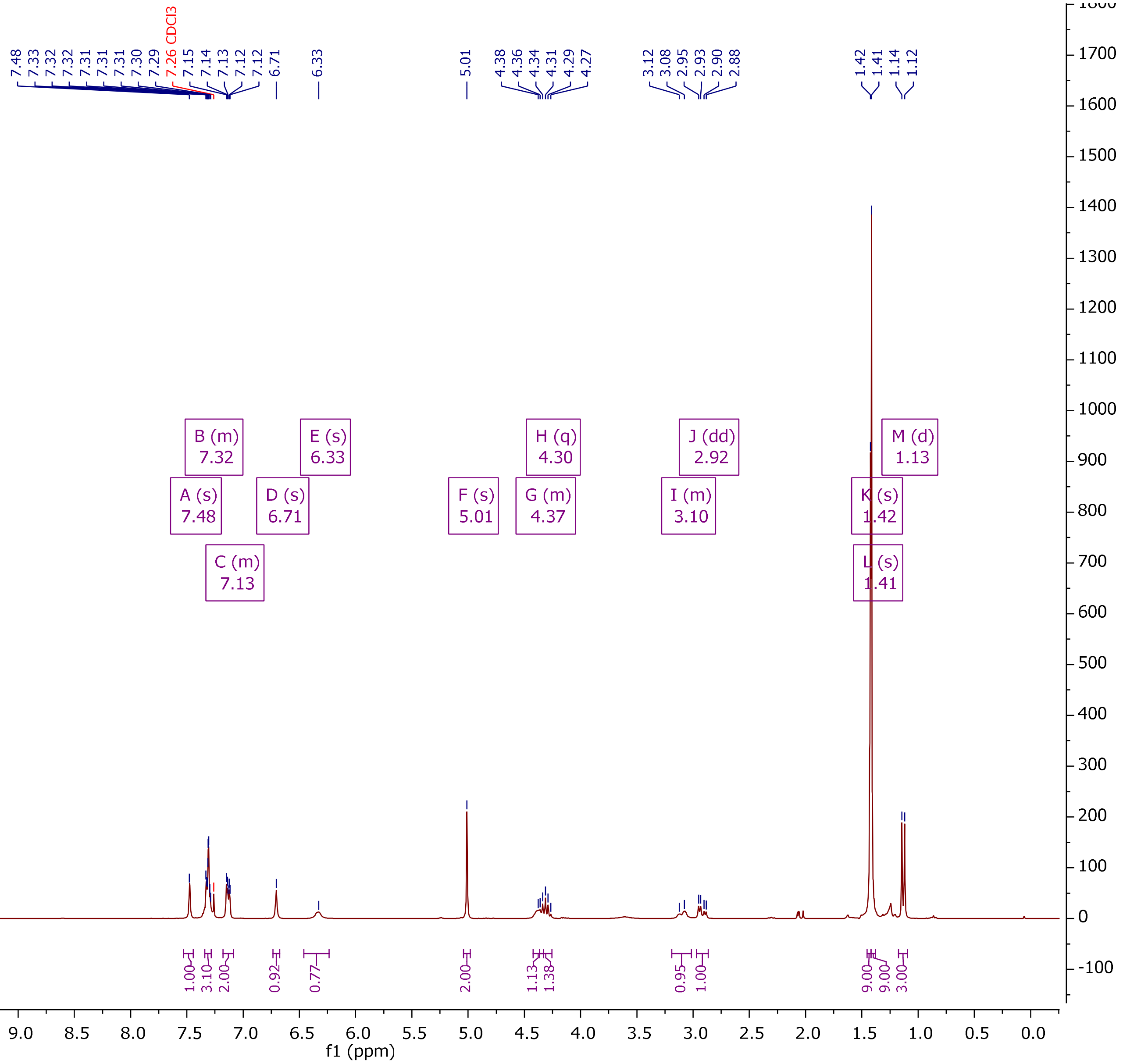

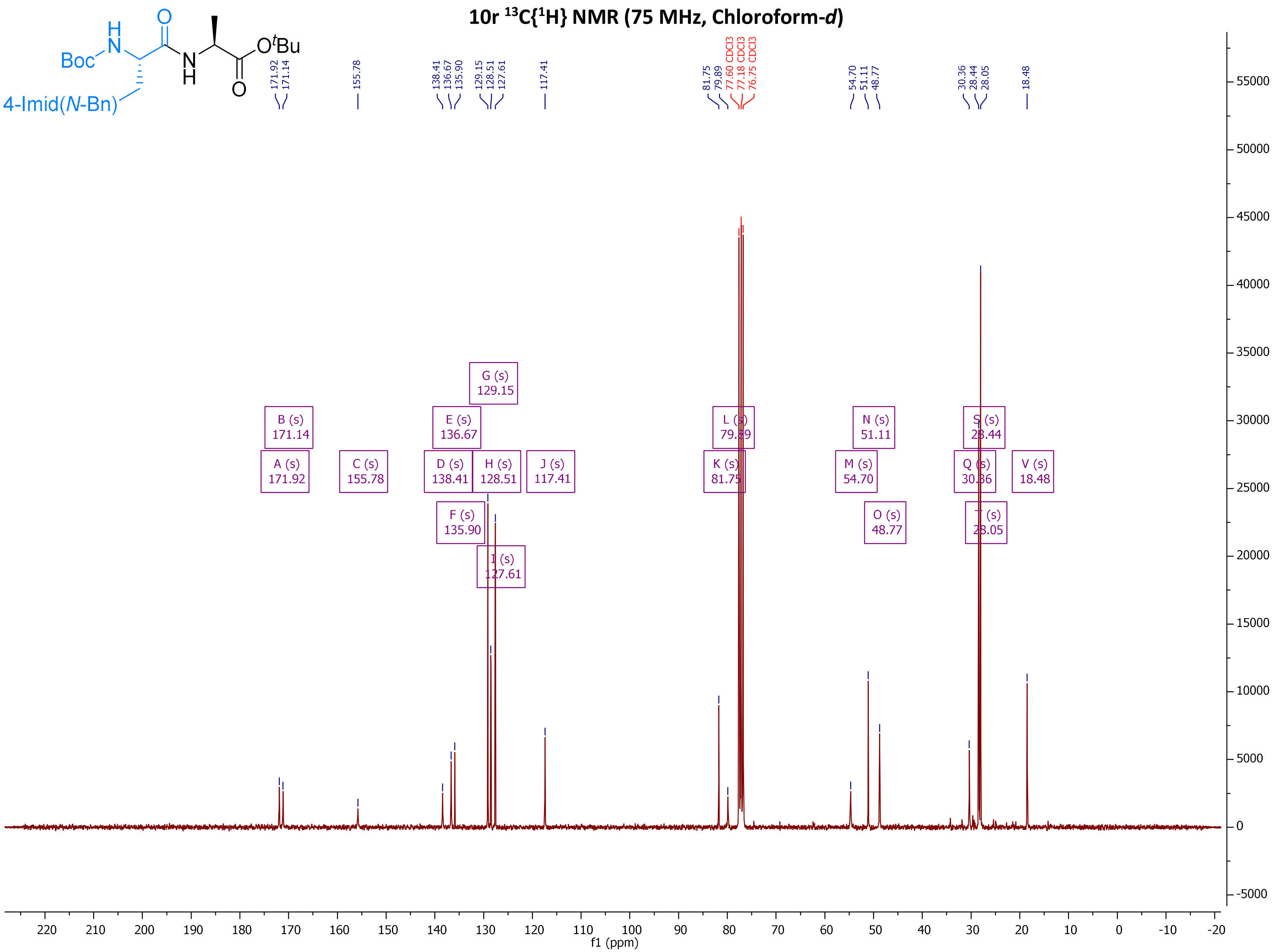

10r $^{13}C\{^1H\}$ NMR (75 MHz, Chloroform-d)
Boc
H
N
O
N
H
O^{t}Bu
O
4-Imid(N-Bn)
171.92
171.14
155.78
138.41
136.67
135.90
129.15
128.51
127.61
117.41
81.75
79.89
77.60 CDCl3
77.18 CDCl3
76.75 CDCl3
54.70
51.11
48.77
30.36
28.44
28.05
18.48
A (s) 171.92
B (s) 171.14
C (s) 155.78
D (s) 138.41
E (s) 136.67
F (s) 135.90
G (s) 129.15
H (s) 128.51
I (s) 127.61
J (s) 117.41
K (s) 81.75
L (s) 79.89
M (s) 54.70
N (s) 51.11
O (s) 48.77
Q (s) 30.36
S (s) 28.44
T (s) 28.05
V (s) 18.48
f1 (ppm)

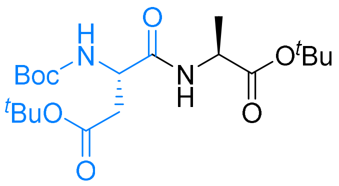

10s ^{1}H NMR (300 MHz, Chloroform-*d*)

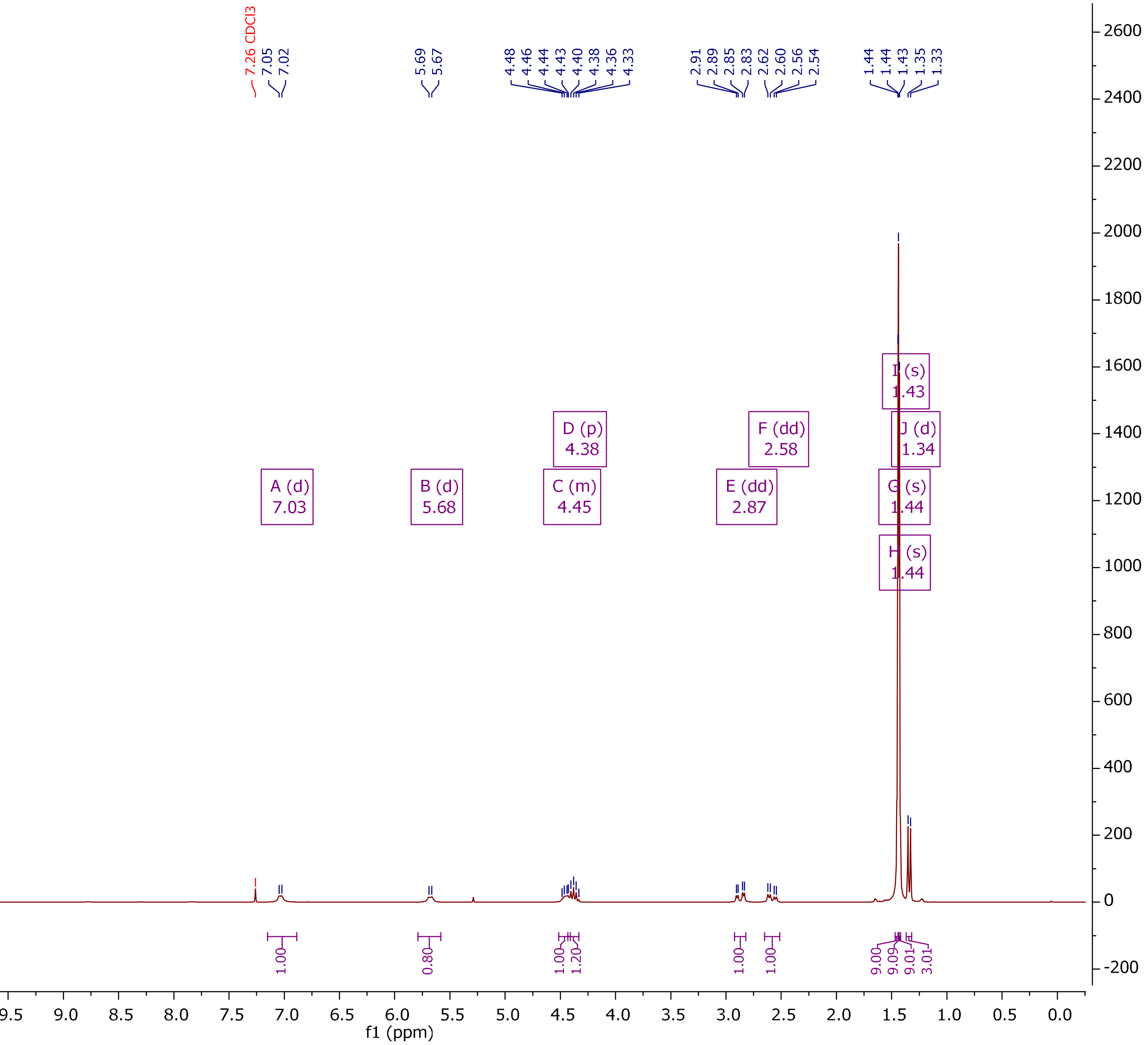

10s $^{13}C\{^{1}H\}$ NMR (75 MHz, Chloroform-*d*)

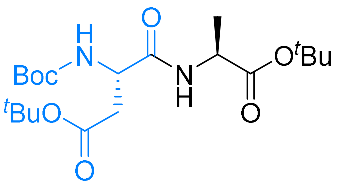

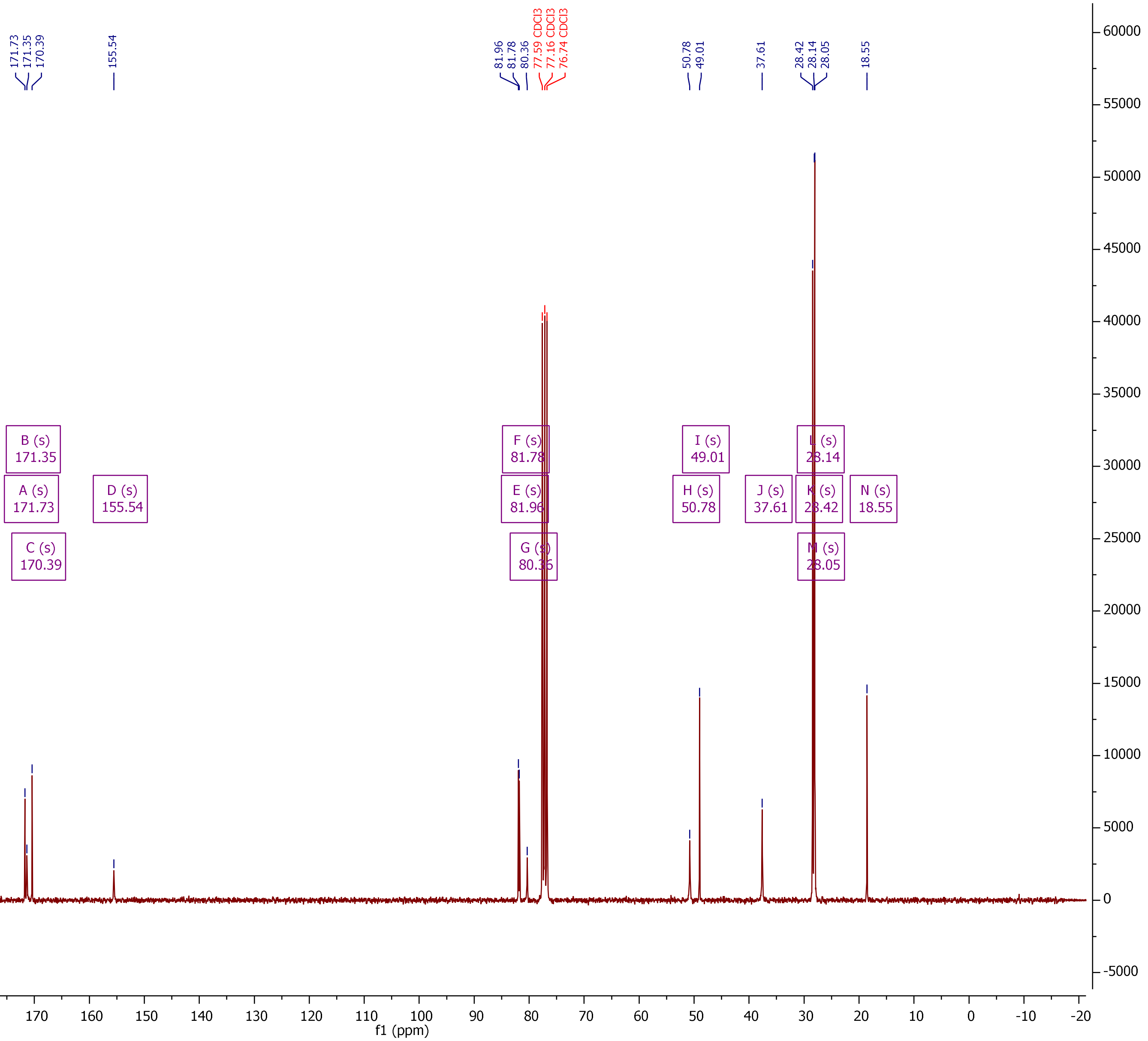

10t ^{1}H NMR (300 MHz, Chloroform-*d*)

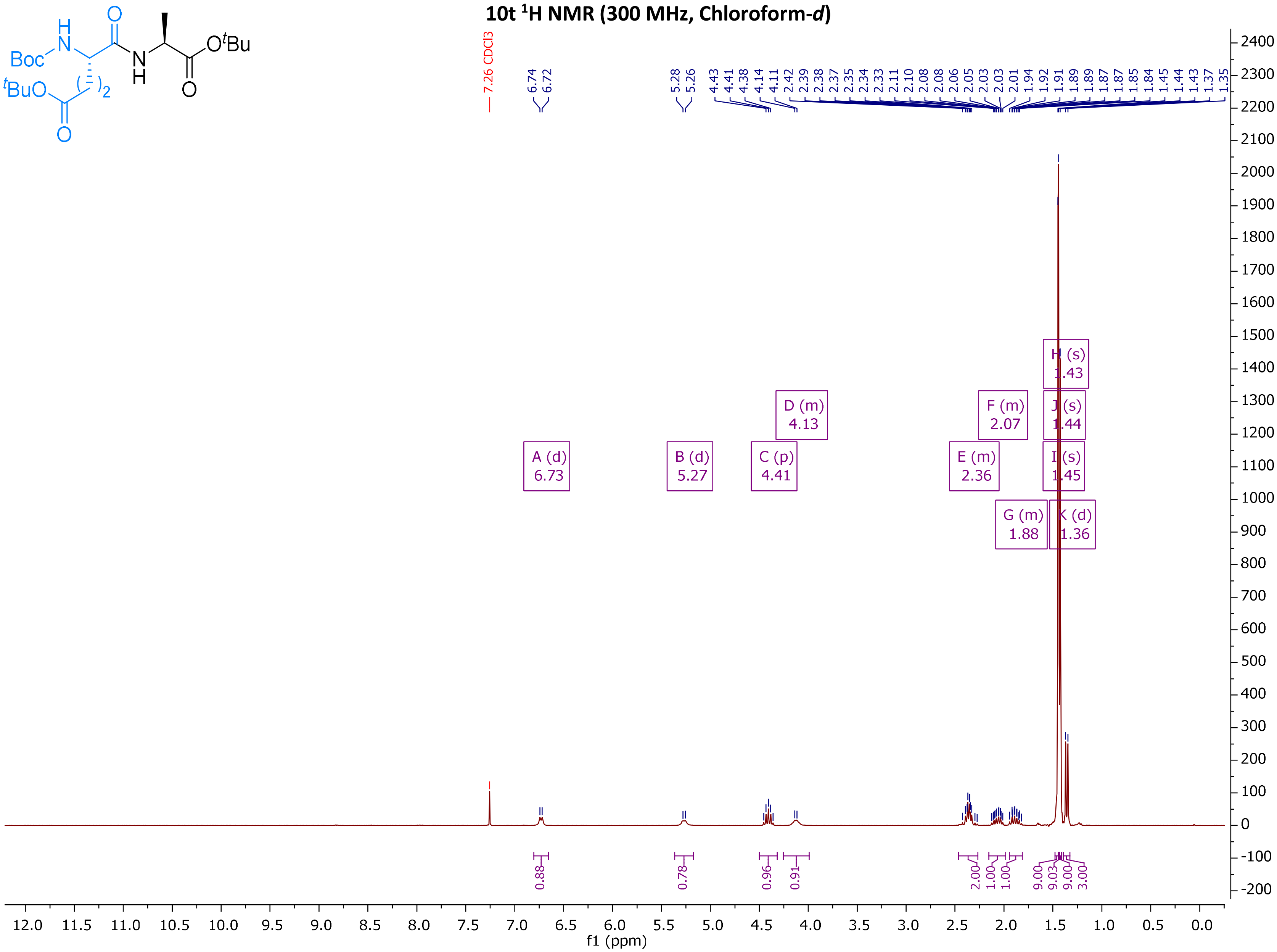

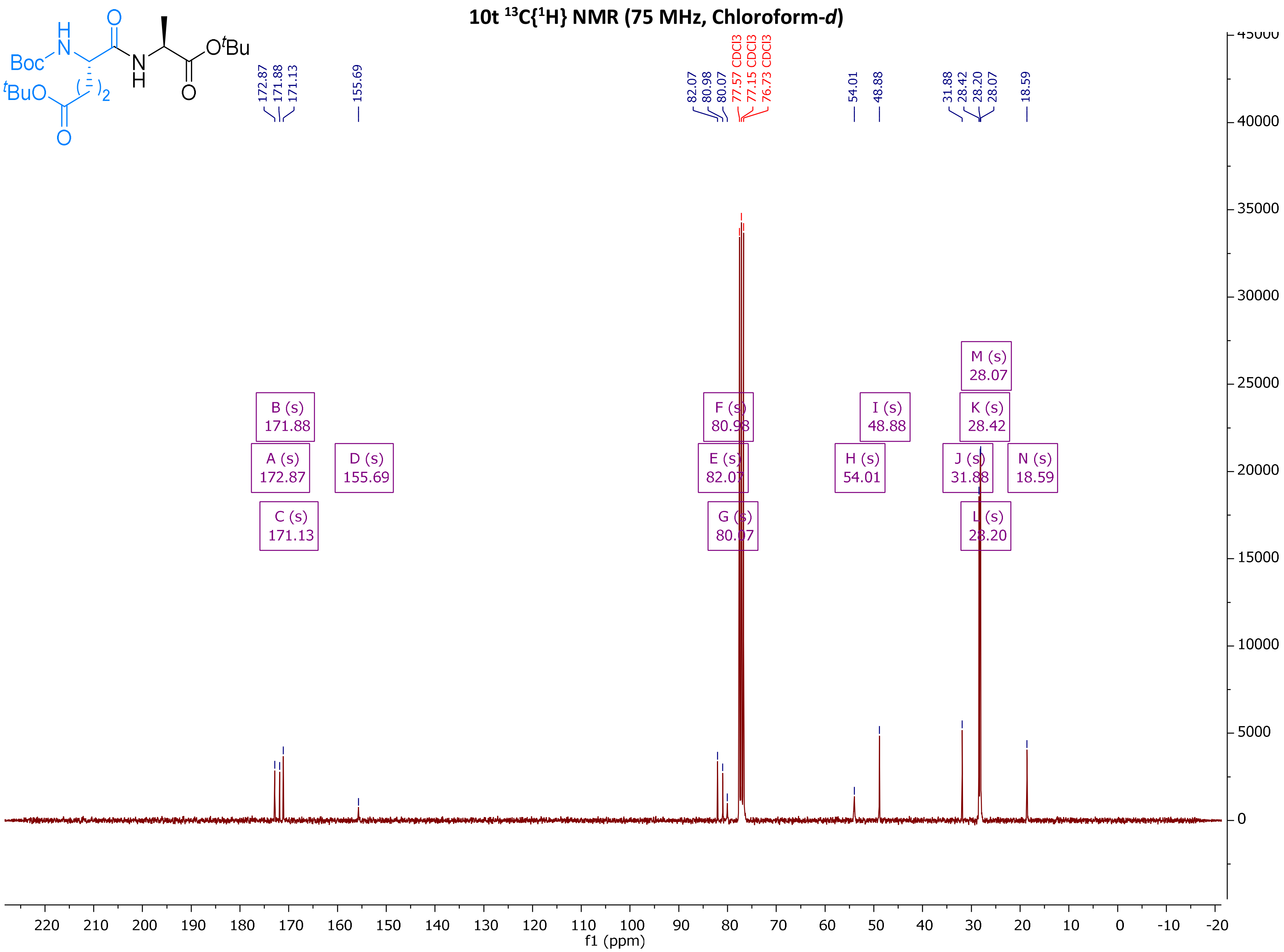
10t ¹³C{¹H} NMR (75 MHz, Chloroform-d)
Boc
H
N
O
N
H
O
O^tBu
^tBuO
( )2
O
172.87
171.88
171.13
155.69
82.07
80.98
80.07
77.57 CDCl3
77.15 CDCl3
76.73 CDCl3
54.01
48.88
31.88
28.42
28.20
28.07
18.59
A (s) 172.87
B (s) 171.88
C (s) 171.13
D (s) 155.69
E (s) 82.07
F (s) 80.98
G (s) 80.07
H (s) 54.01
I (s) 48.88
J (s) 31.88
K (s) 28.42
L (s) 28.20
M (s) 28.07
N (s) 18.59
f1 (ppm)

10u ^{1}H NMR (300 MHz, Chloroform-*d*)

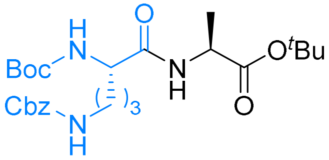

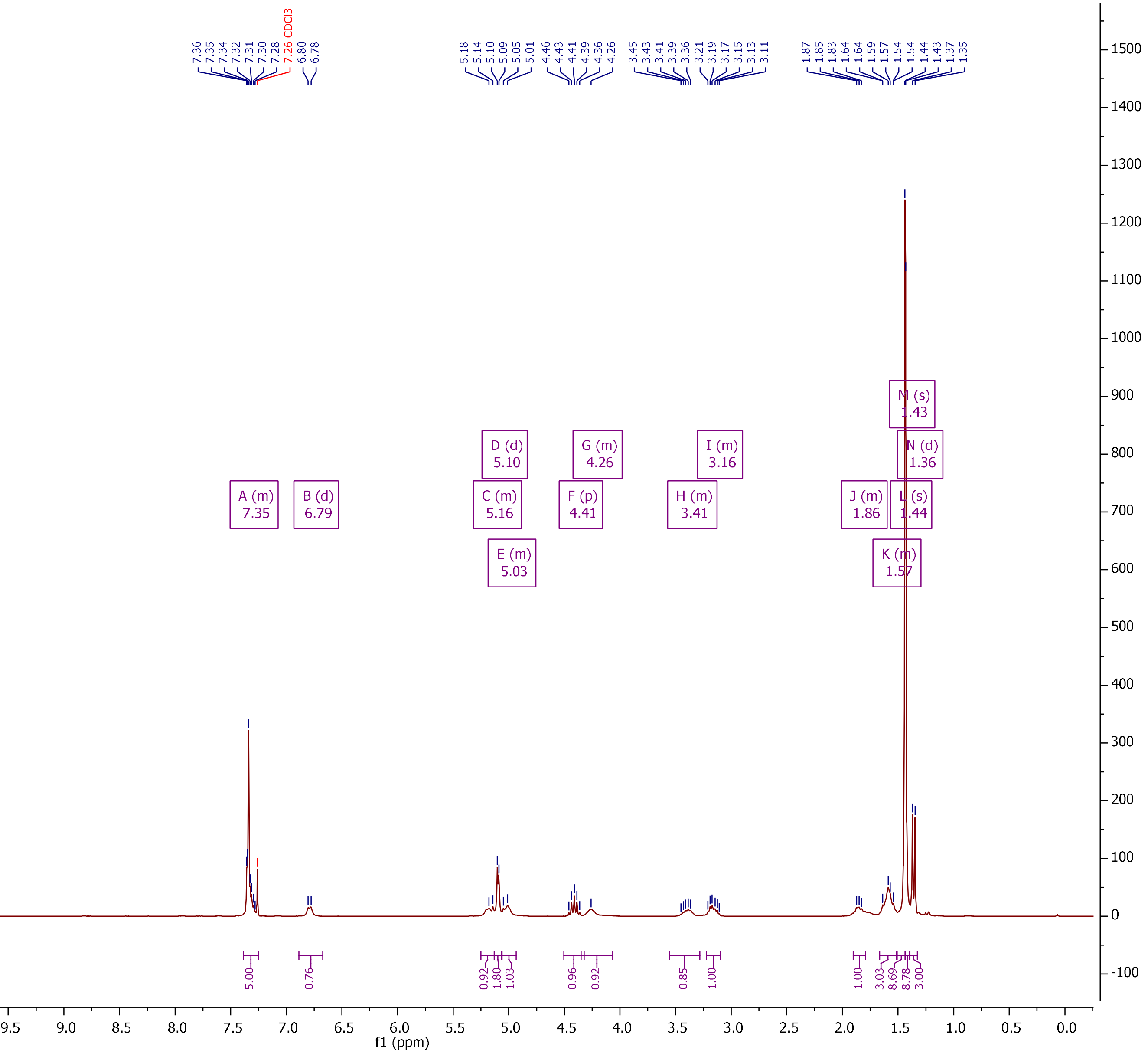

10u $^{13}C\{^{1}H\}$ NMR (75 MHz, Chloroform-*d*)

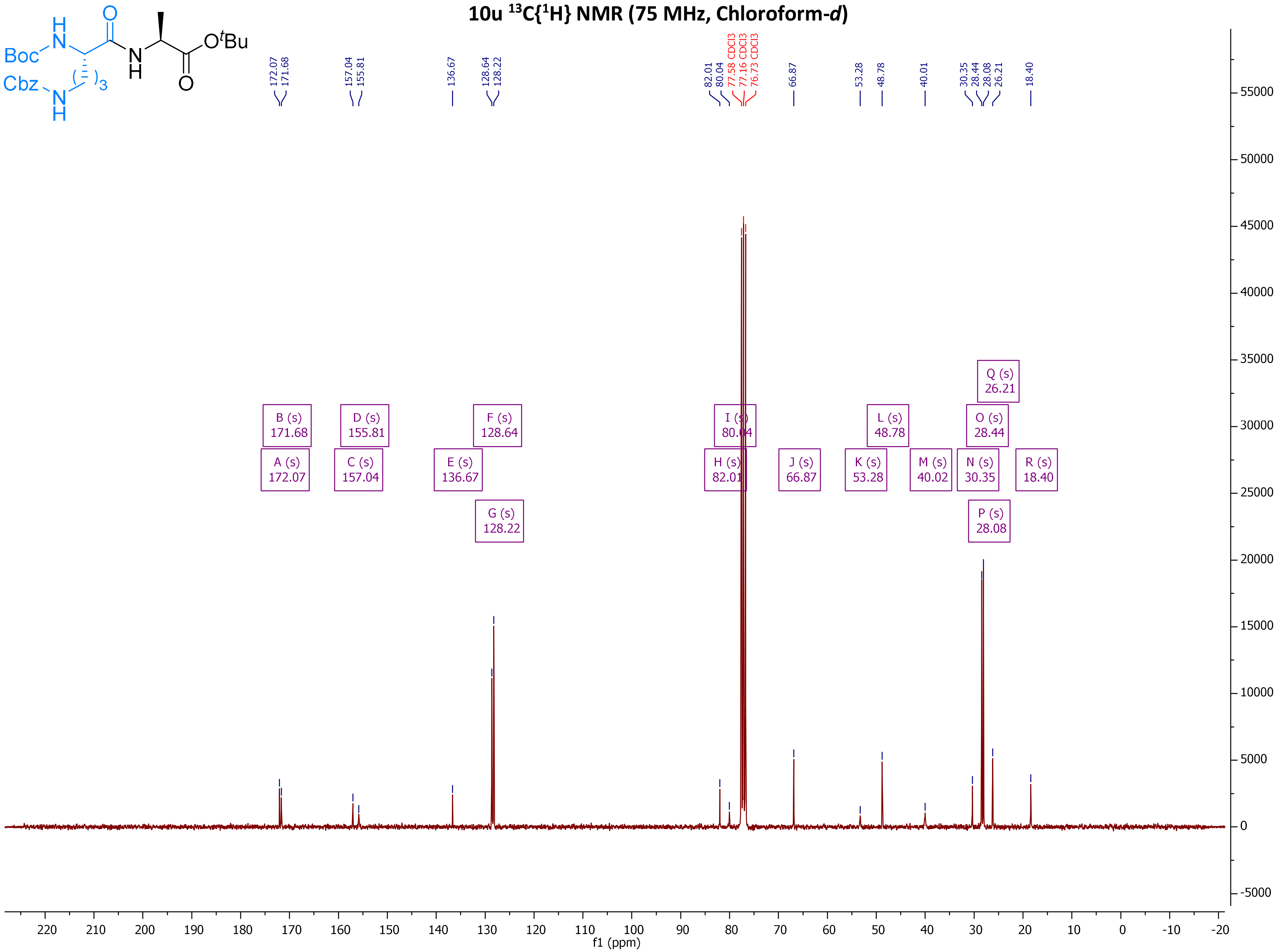

10v ^{1}H NMR (300 MHz, Chloroform-d)

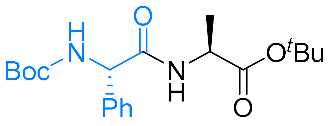

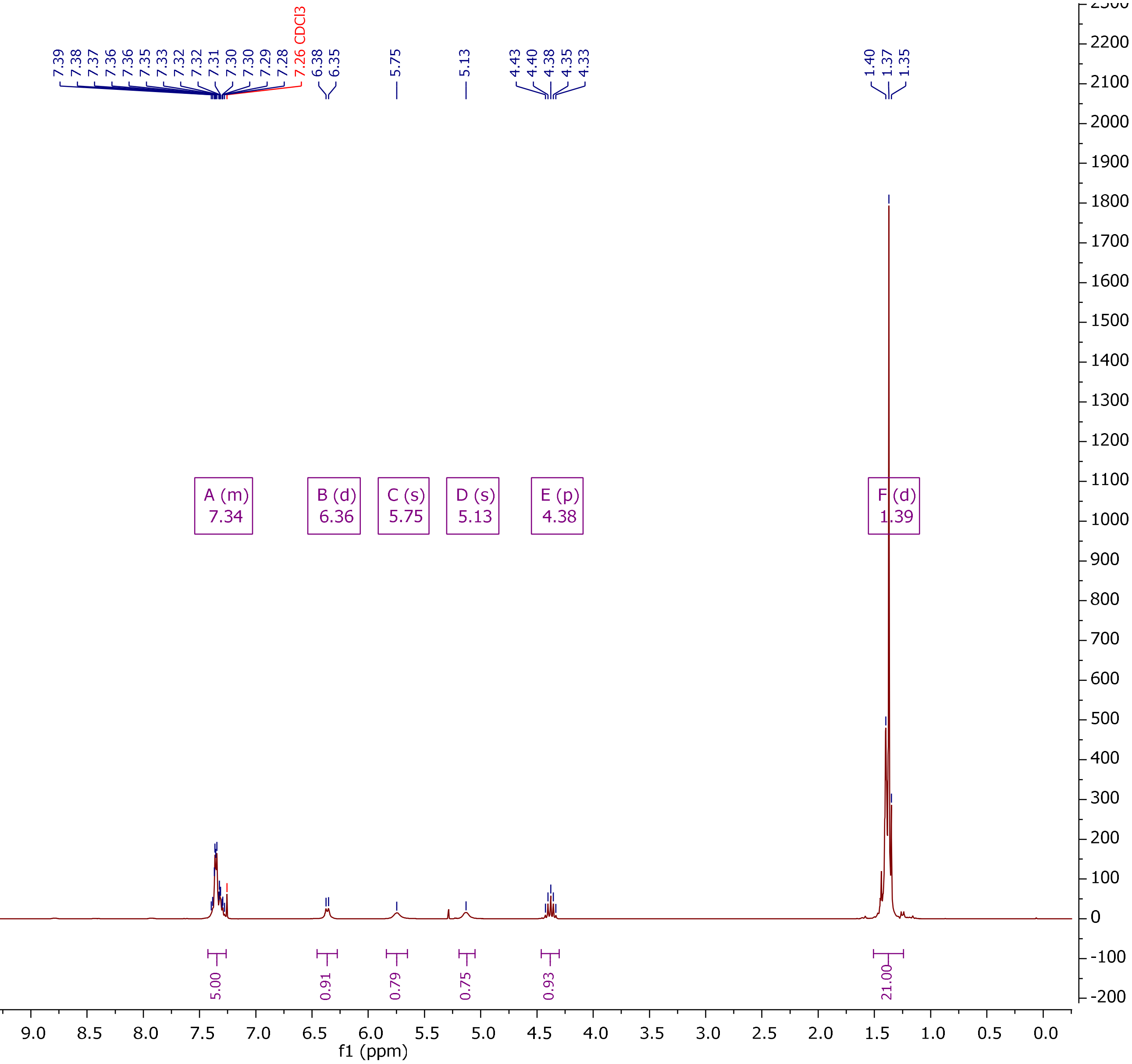

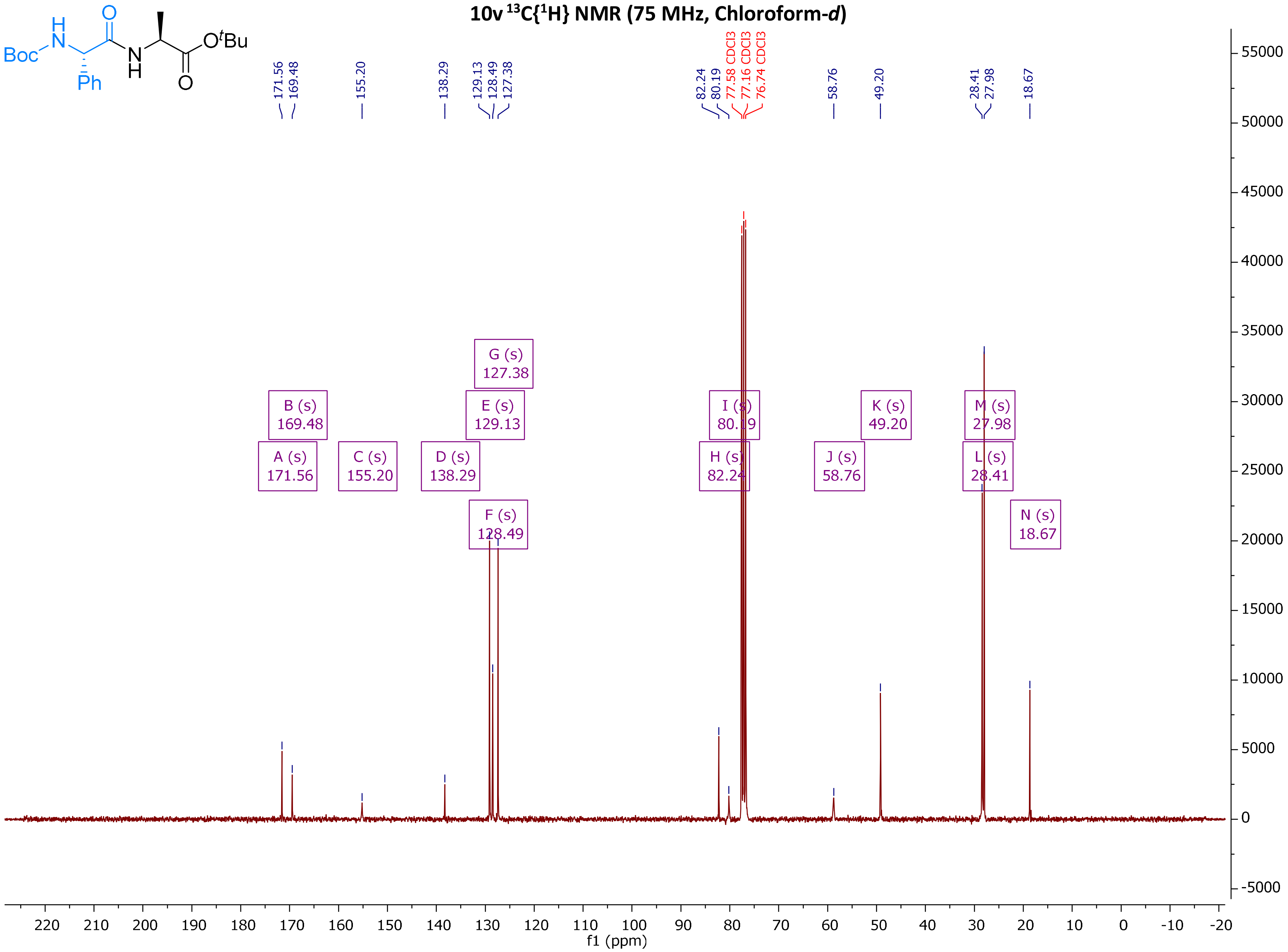

10v 13C{1H} NMR (75 MHz, Chloroform-d)
Boc
H
N
O
N
H
O^tBu
O
Ph
171.56
169.48
155.20
138.29
129.13
128.49
127.38
82.24
80.19
77.58 CDCl3
77.16 CDCl3
76.74 CDCl3
58.76
49.20
28.41
27.98
18.67
A (s) 171.56
B (s) 169.48
C (s) 155.20
D (s) 138.29
E (s) 129.13
F (s) 128.49
G (s) 127.38
H (s) 82.24
I (s) 80.19
J (s) 58.76
K (s) 49.20
L (s) 28.41
M (s) 27.98
N (s) 18.67
f1 (ppm)

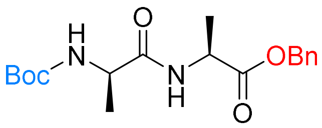

11a ^{1}H NMR (300 MHz, Chloroform-*d*)

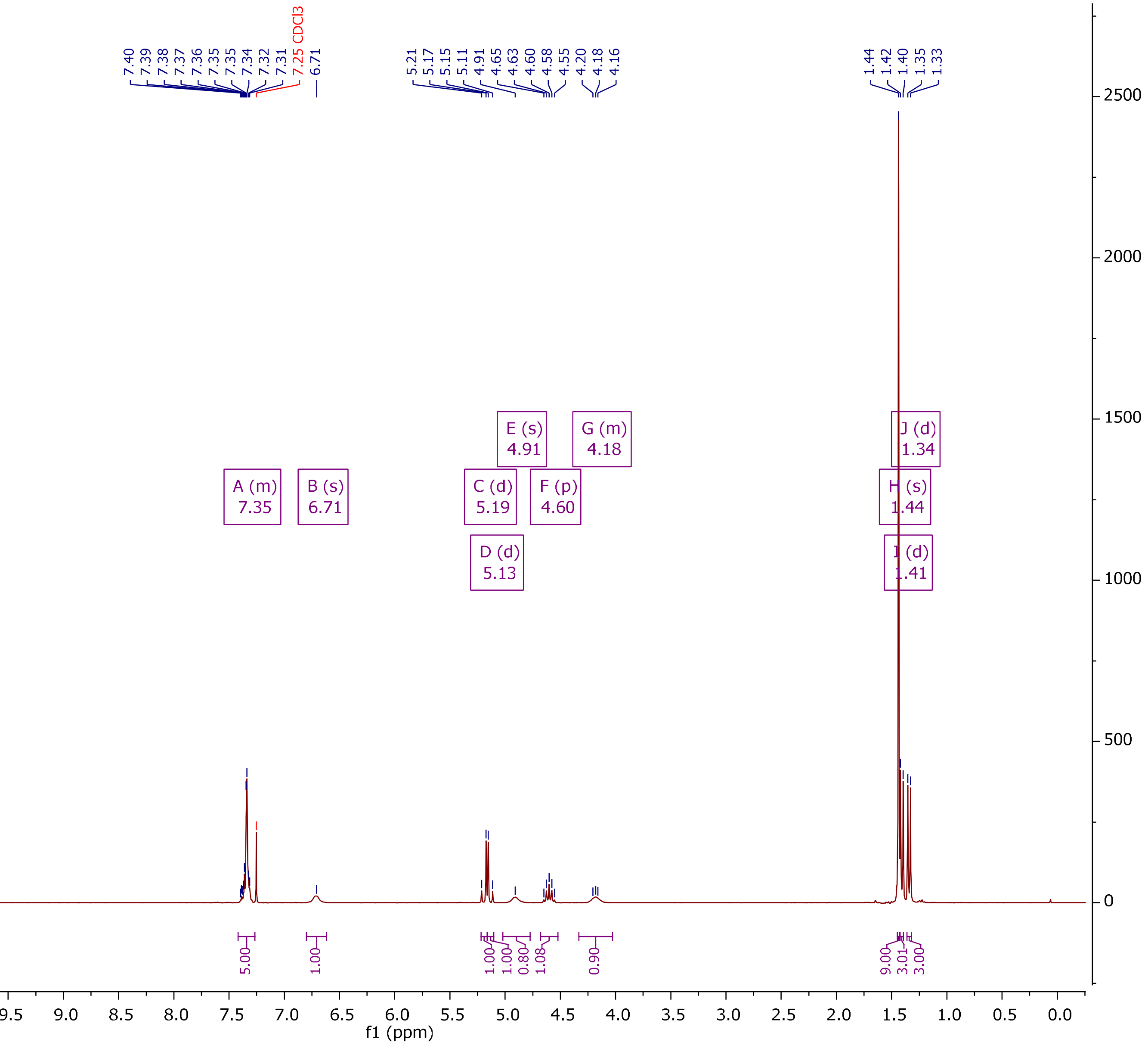

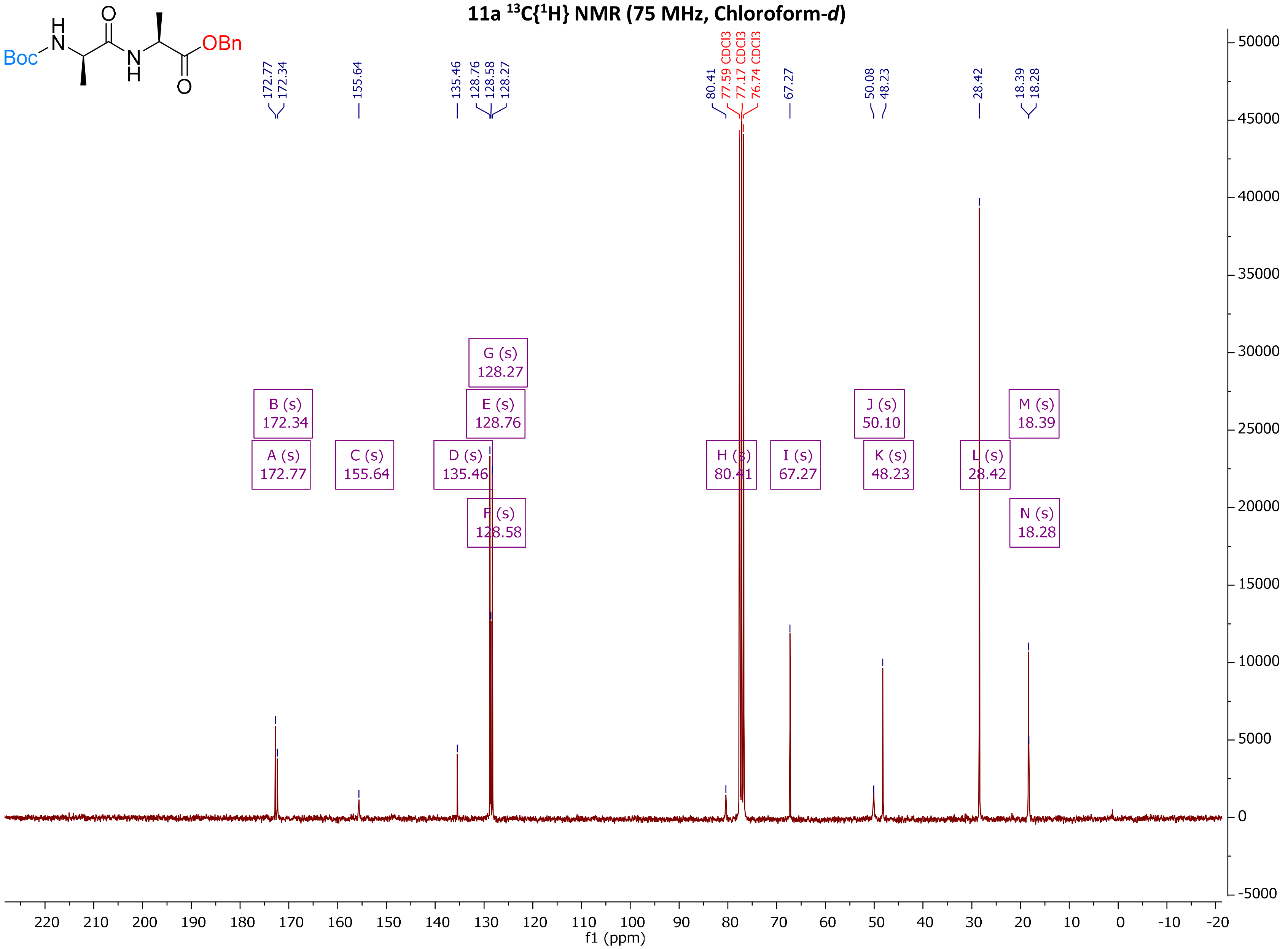

Boc
H
N
O
N
H
OBn
O
11a ¹³C{¹H} NMR (75 MHz, Chloroform-d)
172.77
172.34
155.64
135.46
128.76
128.58
128.27
80.41
77.59 CDCl3
77.17 CDCl3
76.74 CDCl3
67.27
50.08
48.23
28.42
18.39
18.28
A (s) 172.77
B (s) 172.34
C (s) 155.64
D (s) 135.46
E (s) 128.76
F (s) 128.58
G (s) 128.27
H (s) 80.41
I (s) 67.27
J (s) 50.10
K (s) 48.23
L (s) 28.42
M (s) 18.39
N (s) 18.28
f1 (ppm)

11b ¹H NMR (300 MHz, Chloroform-*d*)

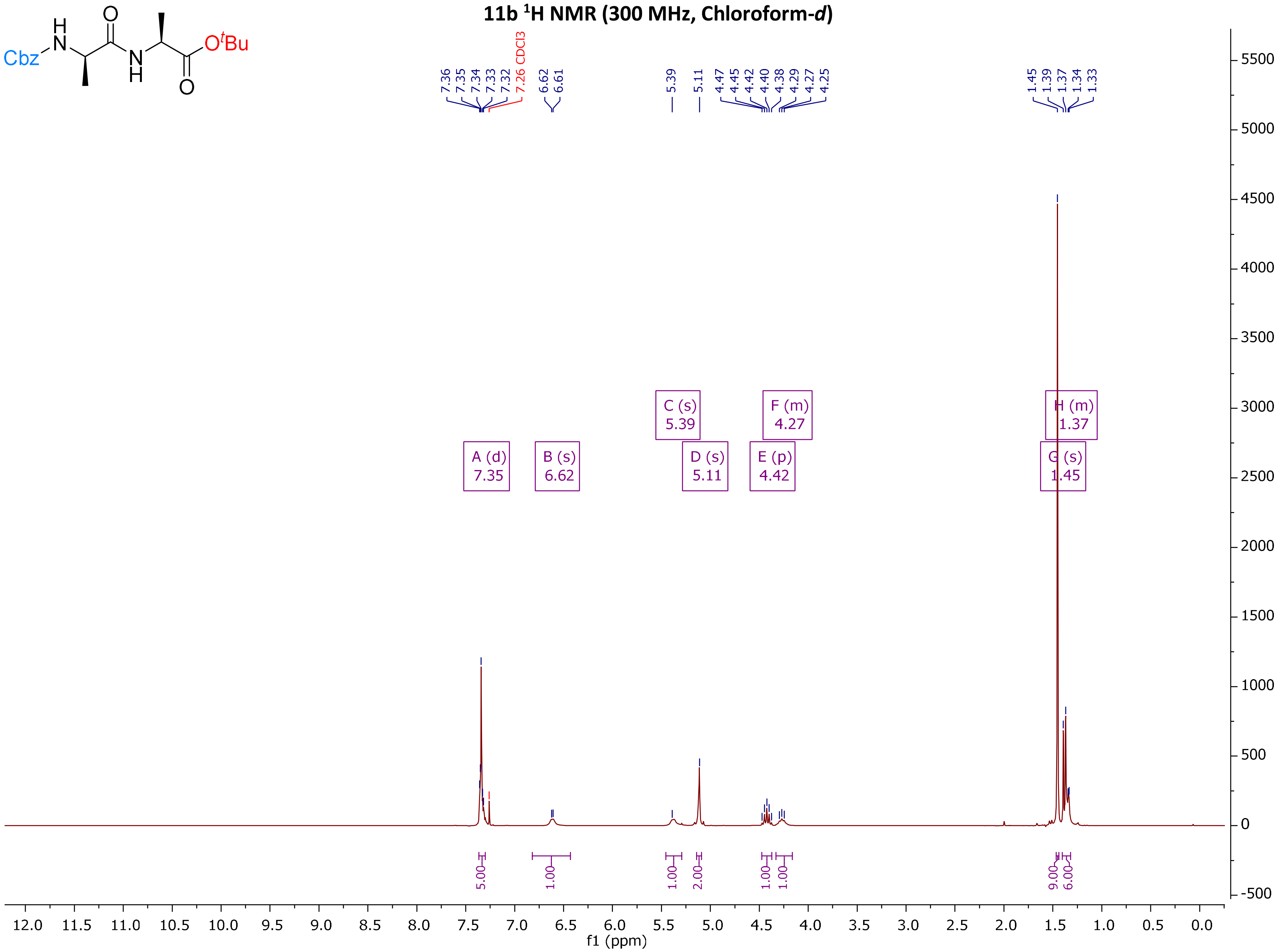

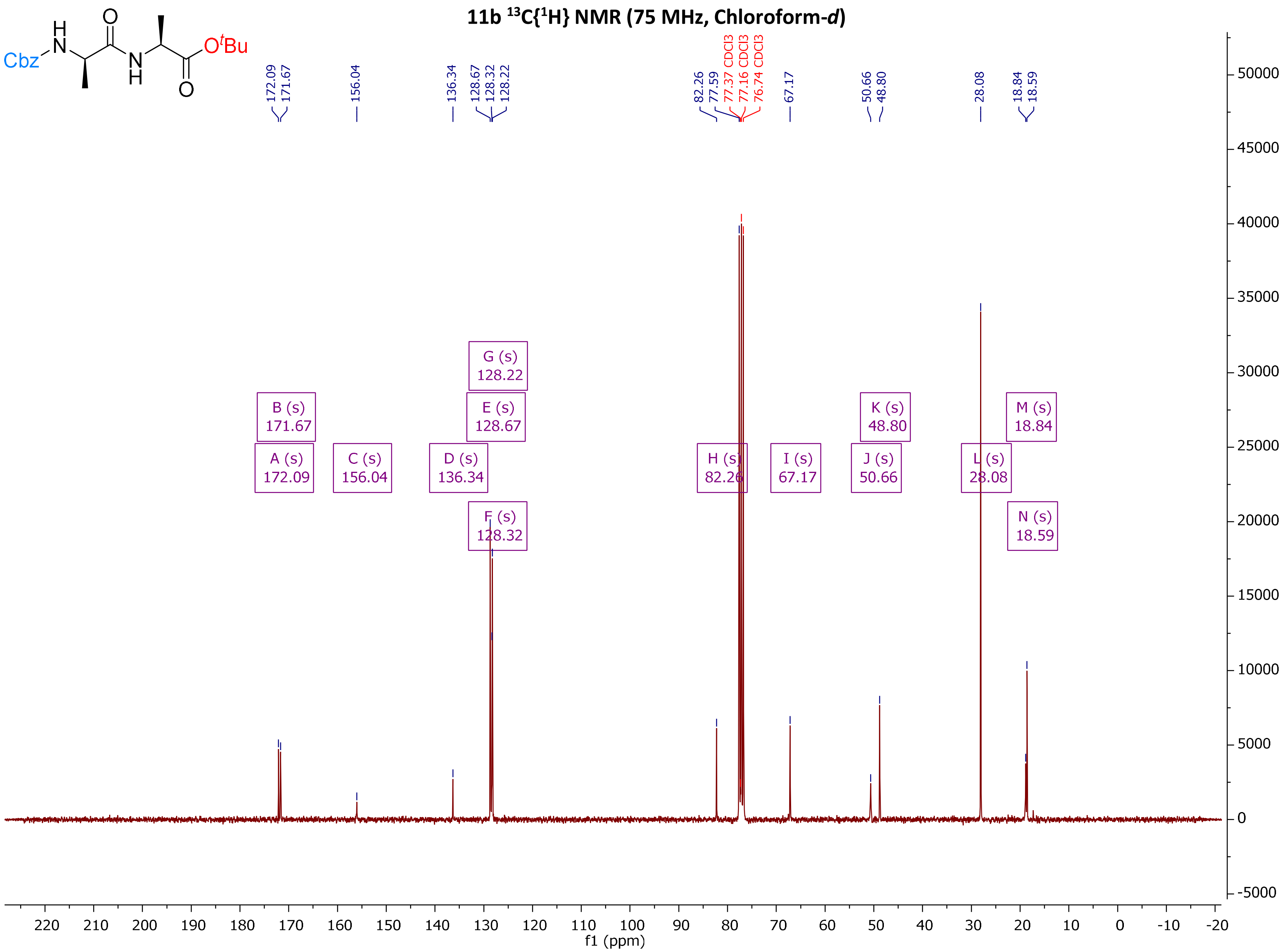
Cbz
O^tBu
11b ^13C{^1H} NMR (75 MHz, Chloroform-d)
172.09
171.67
156.04
136.34
128.67
128.32
128.22
82.26
77.59
77.37 CDCl3
77.16 CDCl3
76.74 CDCl3
67.17
50.66
48.80
28.08
18.84
18.59
A (s) 172.09
B (s) 171.67
C (s) 156.04
D (s) 136.34
E (s) 128.67
F (s) 128.32
G (s) 128.22
H (s) 82.26
I (s) 67.17
J (s) 50.66
K (s) 48.80
L (s) 28.08
M (s) 18.84
N (s) 18.59
f1 (ppm)

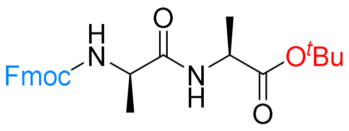

11c ^{1}H NMR (300 MHz, Chloroform-*d*)

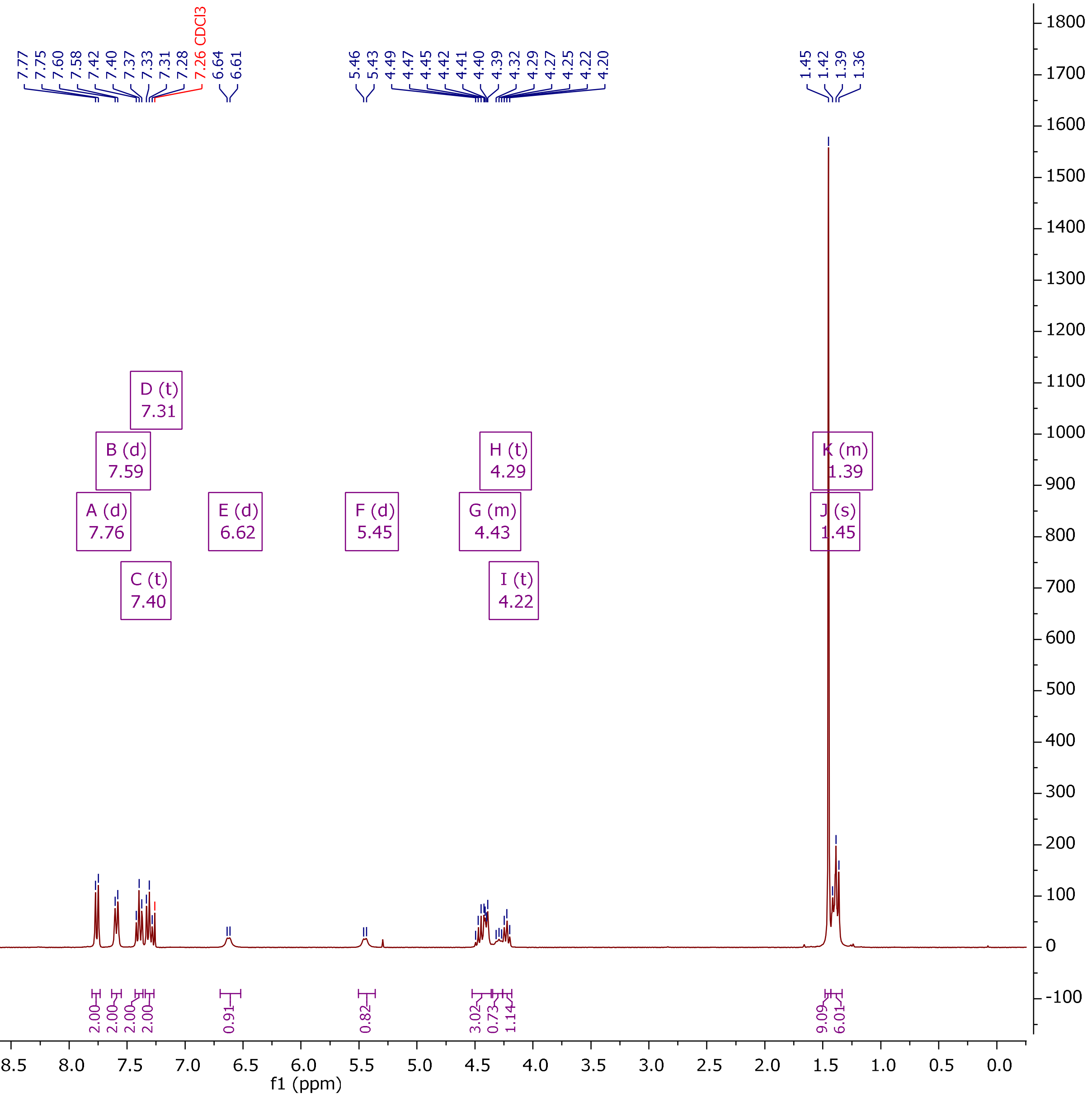

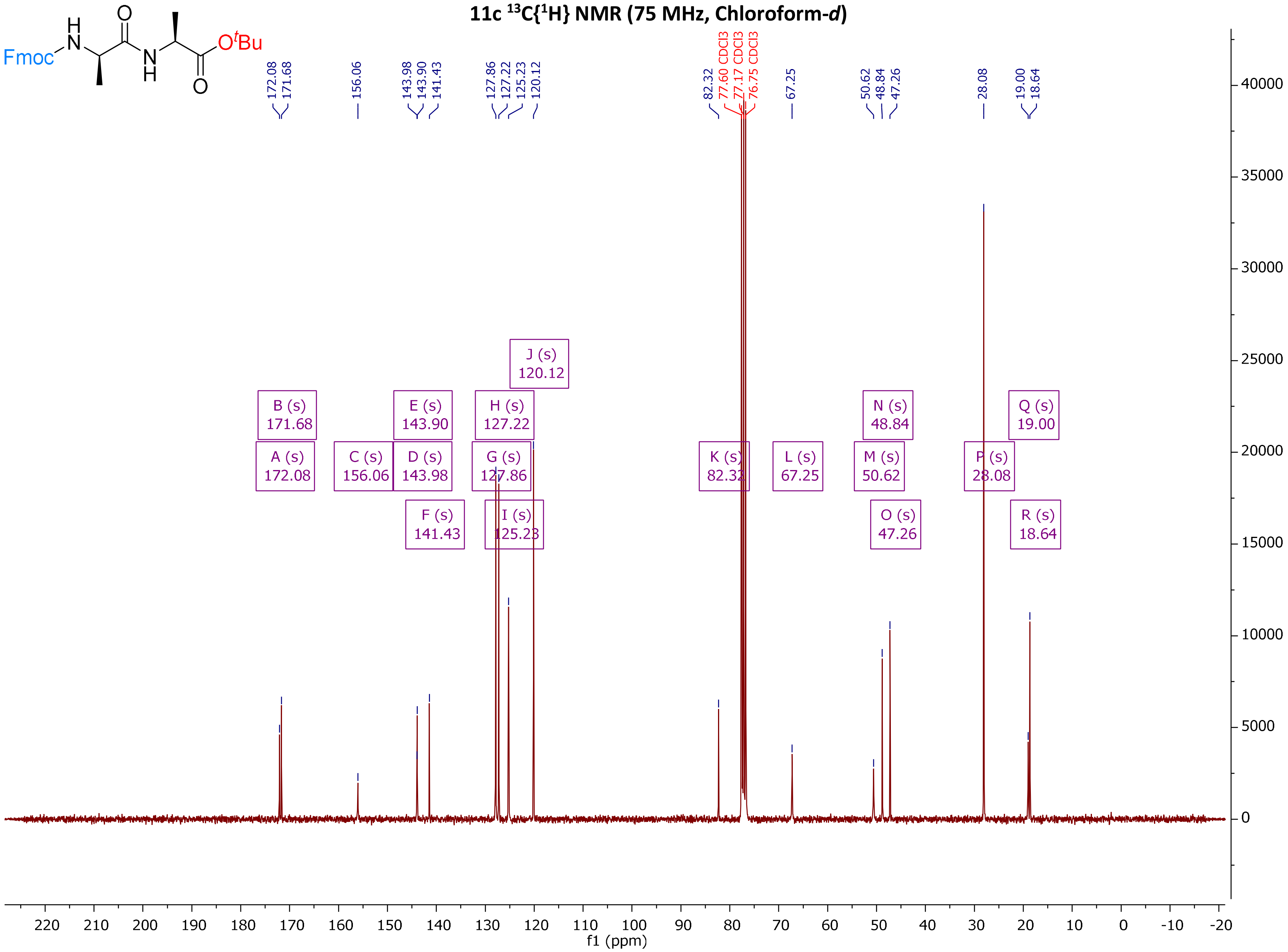

Fmoc
O
N
H
O
OtBu
11c 13C{1H} NMR (75 MHz, Chloroform-d)
172.08
171.68
156.06
143.98
143.90
141.43
127.86
127.22
125.23
120.12
82.32
77.60 CDCl3
77.17 CDCl3
76.75 CDCl3
67.25
50.62
48.84
47.26
28.08
19.00
18.64
A (s) 172.08
B (s) 171.68
C (s) 156.06
D (s) 143.98
E (s) 143.90
F (s) 141.43
G (s) 127.86
H (s) 127.22
I (s) 125.23
J (s) 120.12
K (s) 82.32
L (s) 67.25
M (s) 50.62
N (s) 48.84
O (s) 47.26
P (s) 28.08
Q (s) 19.00
R (s) 18.64
f1 (ppm)

11d ^1H NMR (300 MHz, Chloroform-*d*)

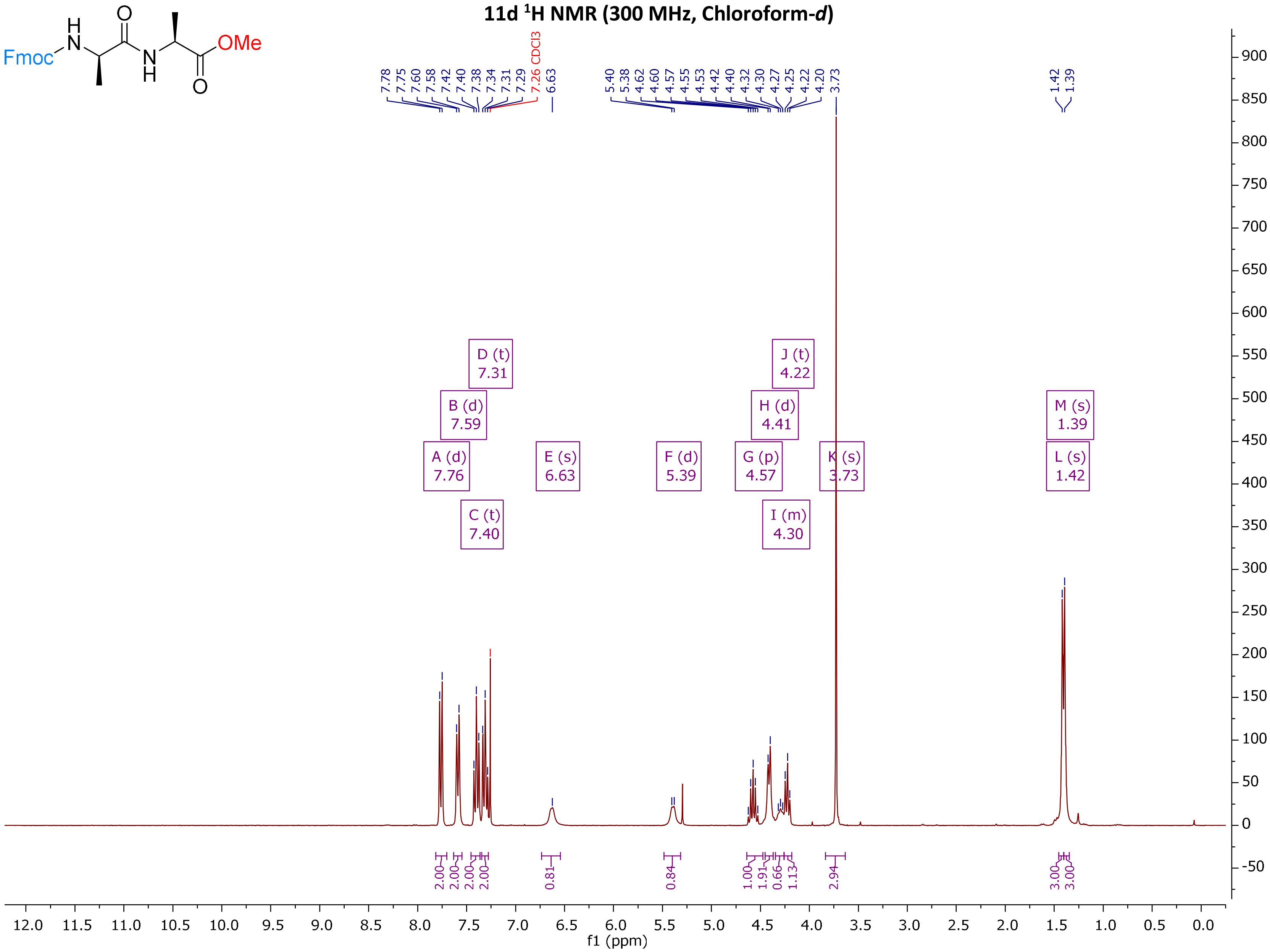

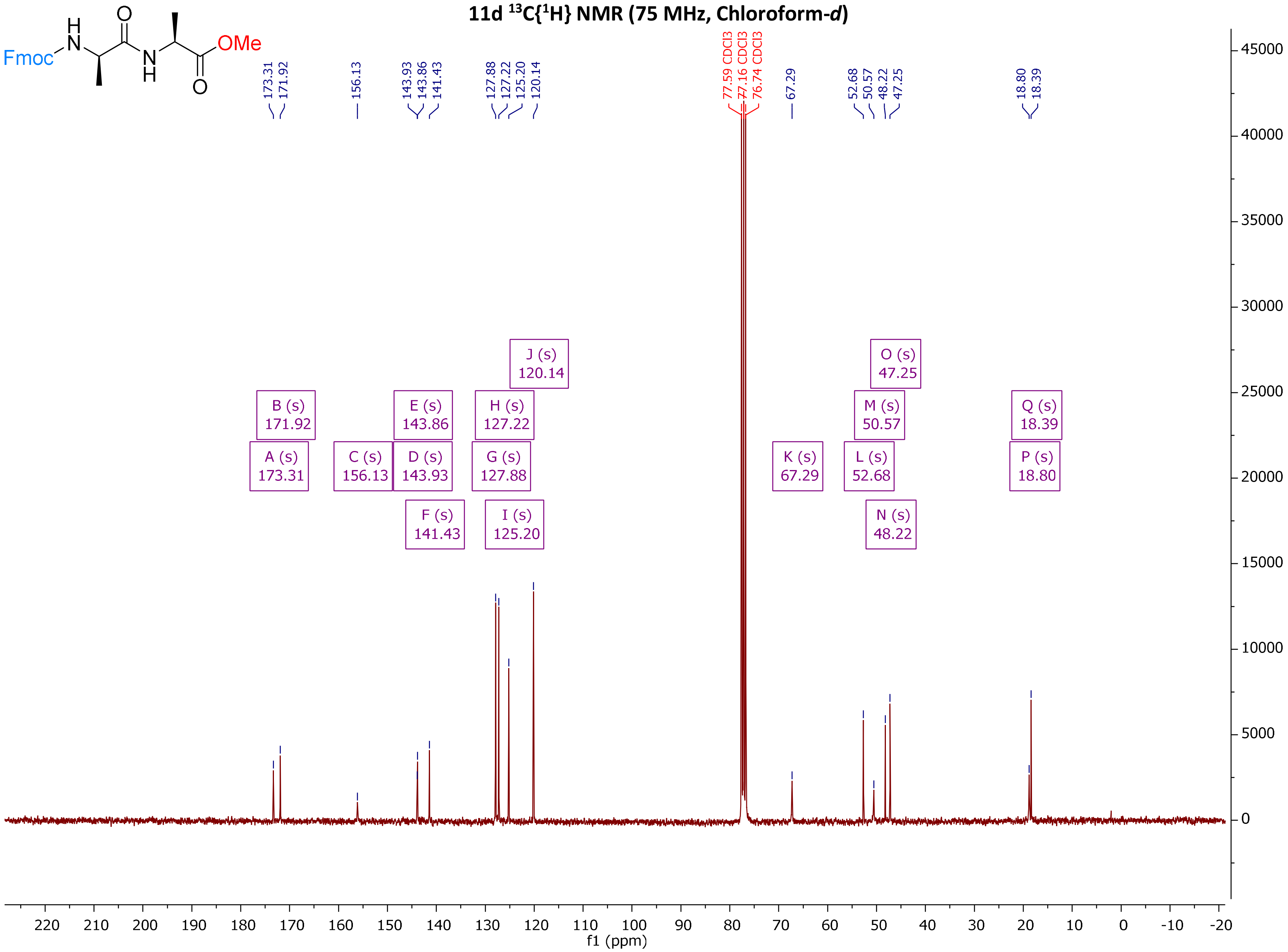

Fmoc
OMe
11d ¹³C{¹H} NMR (75 MHz, Chloroform-d)
173.31
171.92
156.13
143.93
143.86
141.43
127.88
127.22
125.20
120.14
77.59 CDCl3
77.16 CDCl3
76.74 CDCl3
67.29
52.68
50.57
48.22
47.25
18.80
18.39
A (s) 173.31
B (s) 171.92
C (s) 156.13
D (s) 143.93
E (s) 143.86
F (s) 141.43
G (s) 127.88
H (s) 127.22
I (s) 125.20
J (s) 120.14
K (s) 67.29
L (s) 52.68
M (s) 50.57
N (s) 48.22
O (s) 47.25
P (s) 18.80
Q (s) 18.39
f1 (ppm)

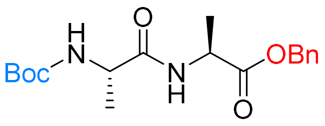

11e ^{1}H NMR (300 MHz, Chloroform-*d*)

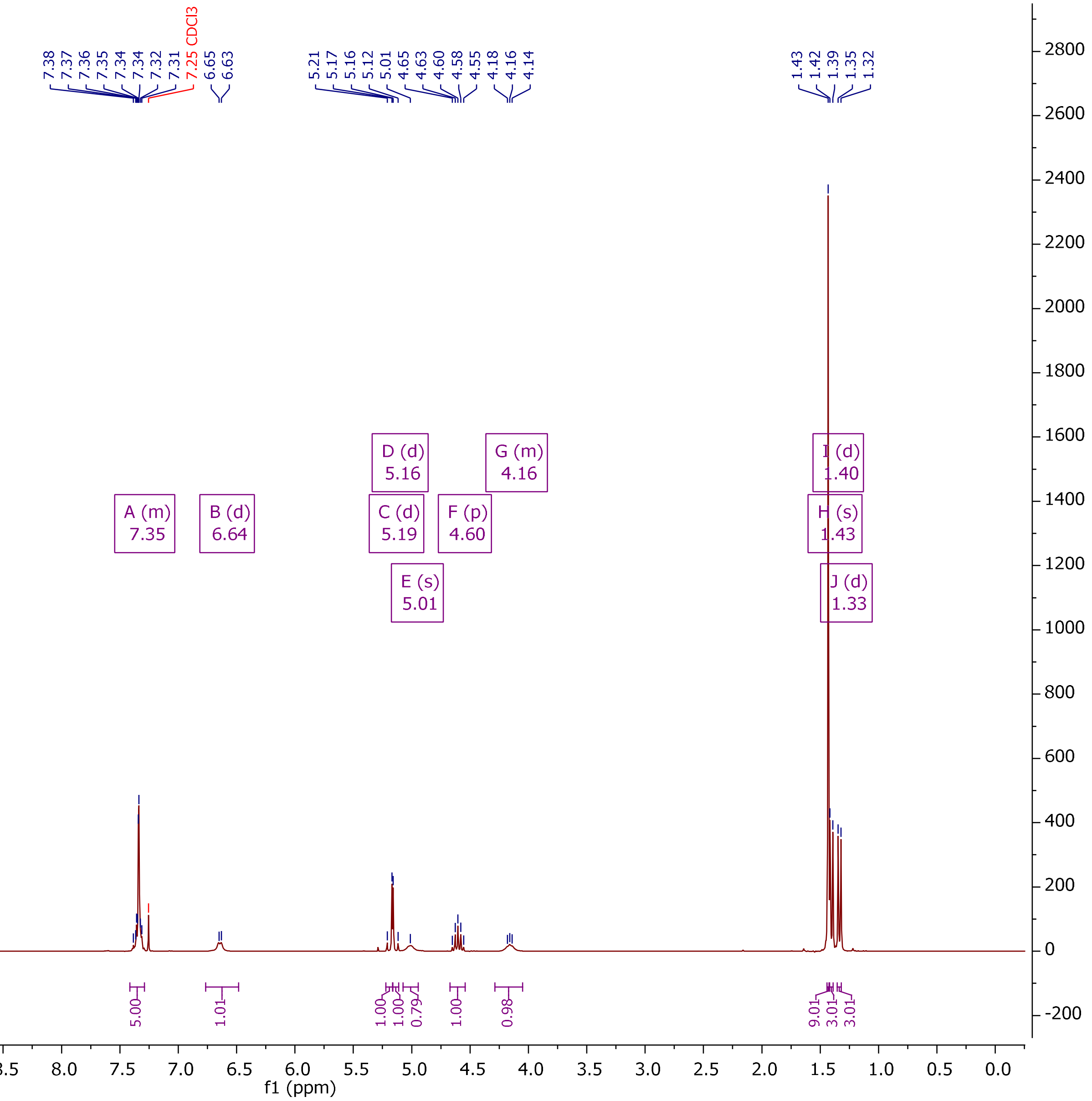

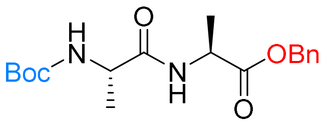

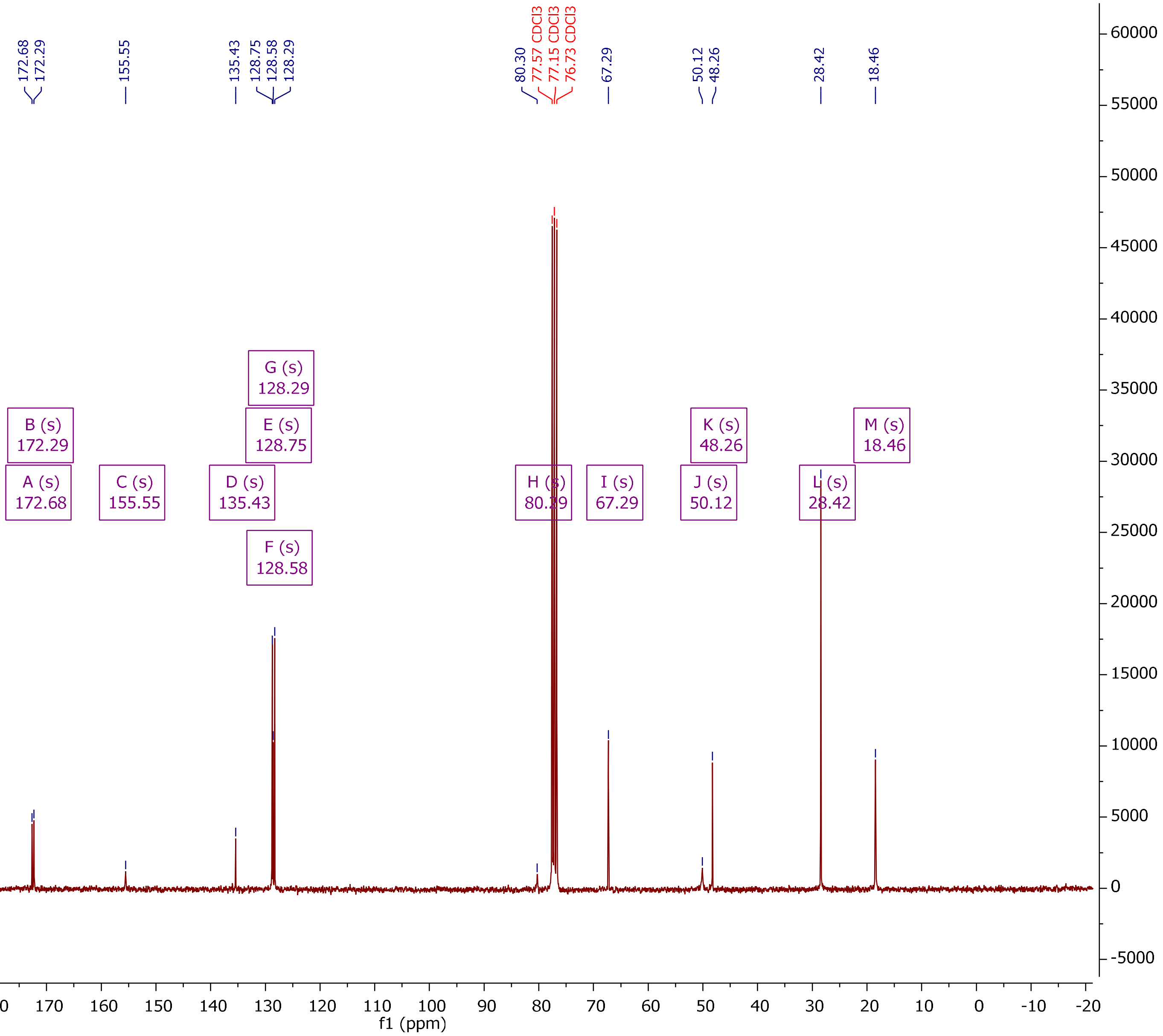

$^{13}C\{^{1}H\}$ NMR (75 MHz, Chloroform-*d*)
172.68
172.29
155.55
135.43
128.75
128.58
128.29
80.30
77.57 CDCl3
77.15 CDCl3
76.73 CDCl3
67.29
50.12
48.26
28.42
18.46
A (s) 172.68
B (s) 172.29
C (s) 155.55
D (s) 135.43
E (s) 128.75
F (s) 128.58
G (s) 128.29
H (s) 80.29
I (s) 67.29
J (s) 50.12
K (s) 48.26
L (s) 28.42
M (s) 18.46
f1 (ppm)

12a ^{1}H NMR (300 MHz, Chloroform-*d*)

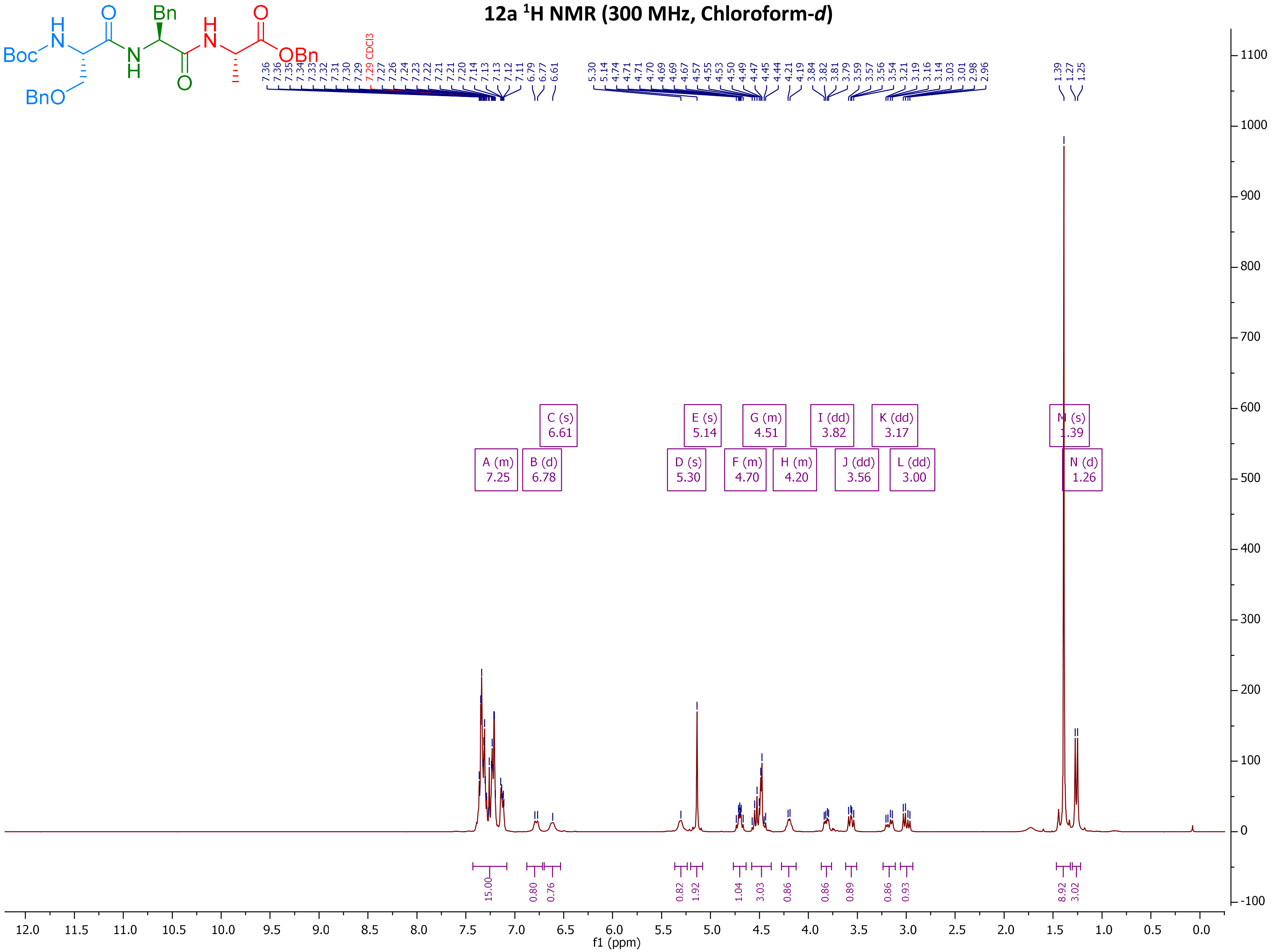

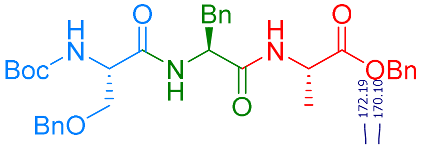

12a $^{13}C\{^{1}H\}$ NMR (75 MHz, Chloroform-*d*)

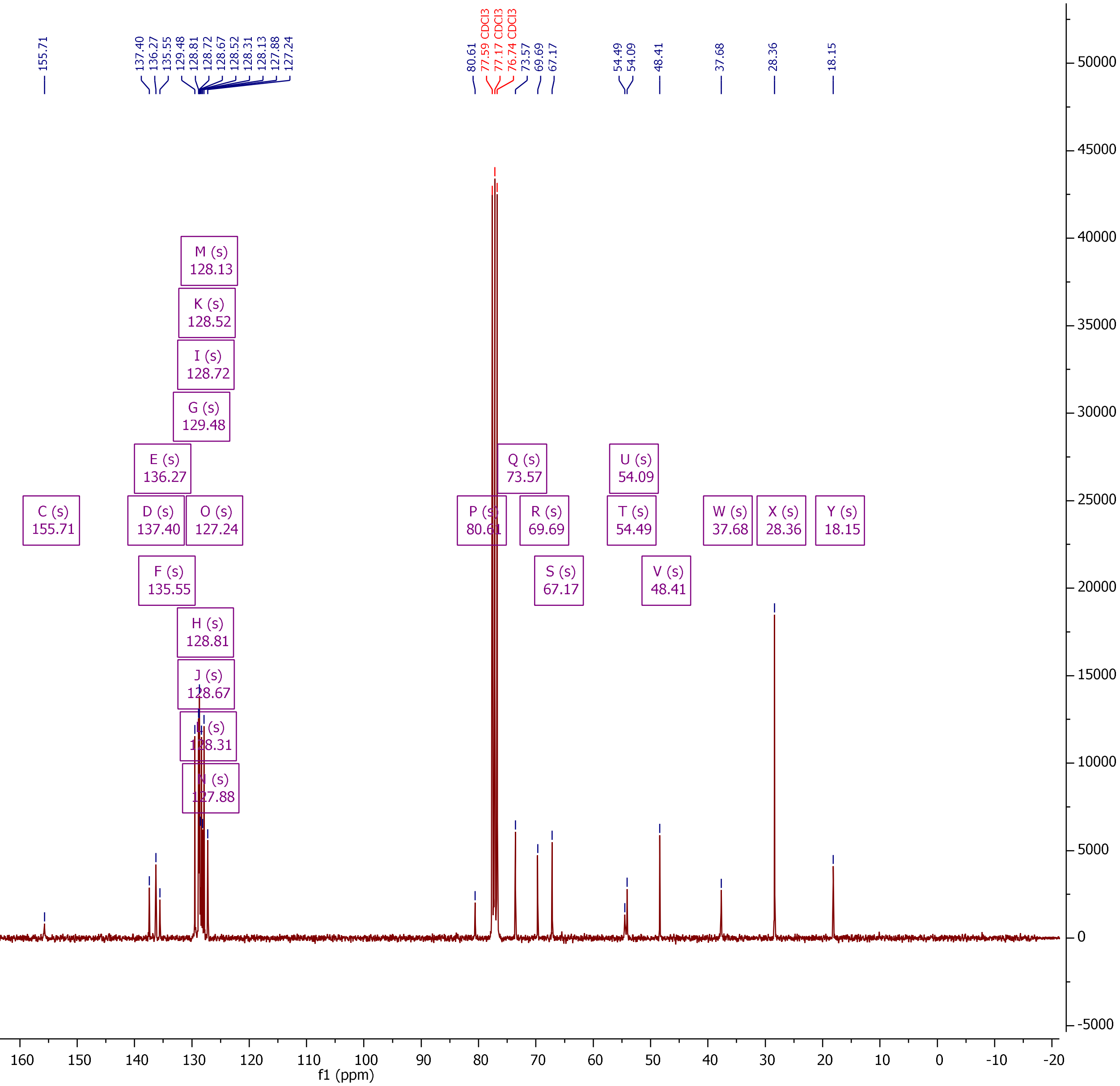

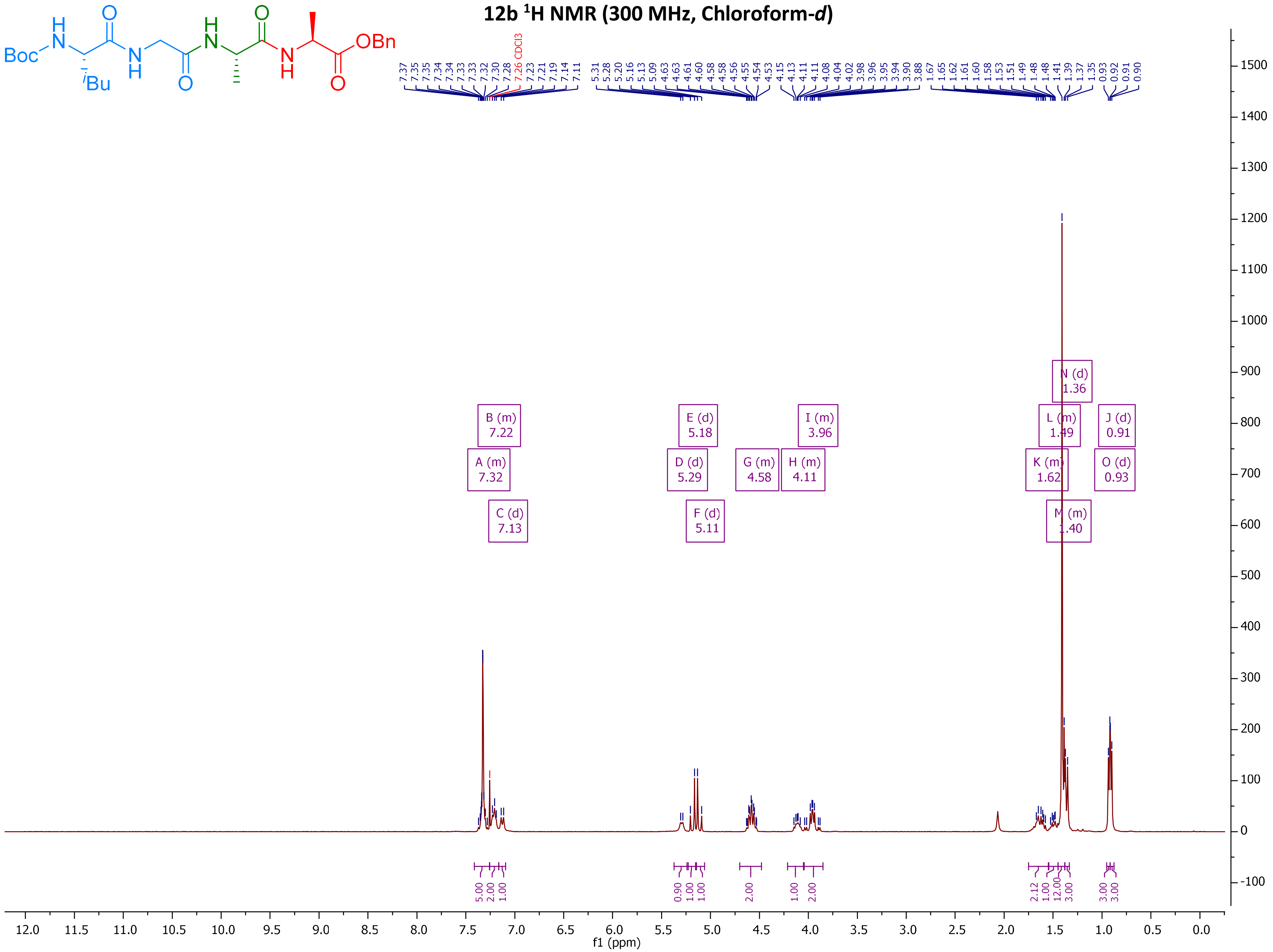

12b ¹H NMR (300 MHz, Chloroform-d)
Boc
OBn
ⁱBu
A (m) 7.32
B (m) 7.22
C (d) 7.13
D (d) 5.29
E (d) 5.18
F (d) 5.11
G (m) 4.58
H (m) 4.11
I (m) 3.96
J (d) 0.91
K (m) 1.62
L (m) 1.49
M (m) 1.40
N (d) 1.36
O (d) 0.93
f1 (ppm)

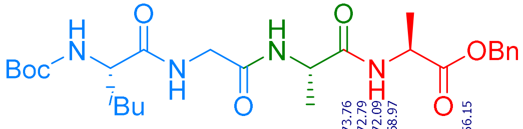

12b $^{13}C\{^{1}H\}$ NMR (75 MHz, Chloroform-*d*)

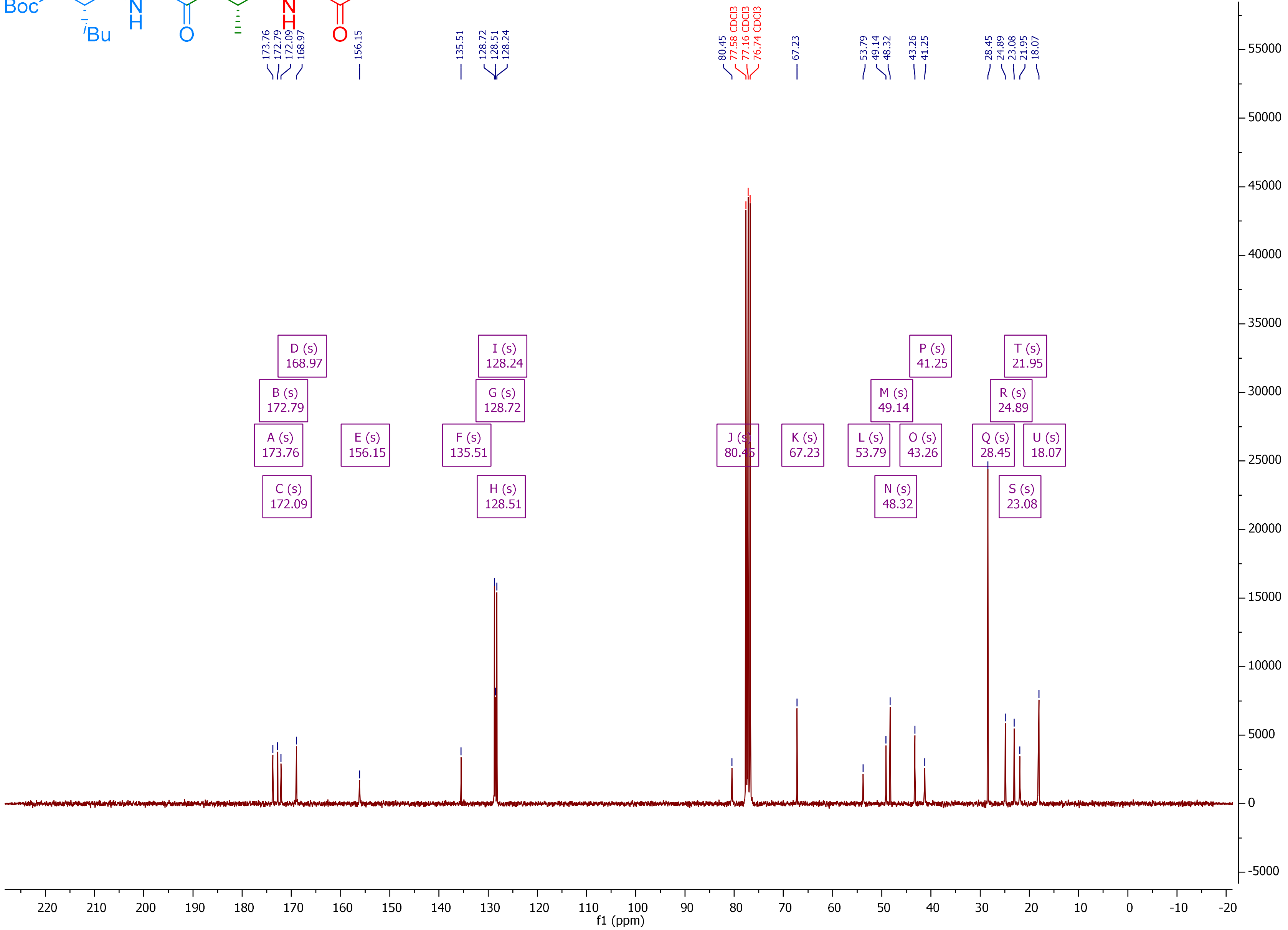

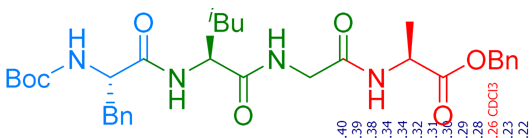

12c ^{1}H NMR (300 MHz, Chloroform-*d*)

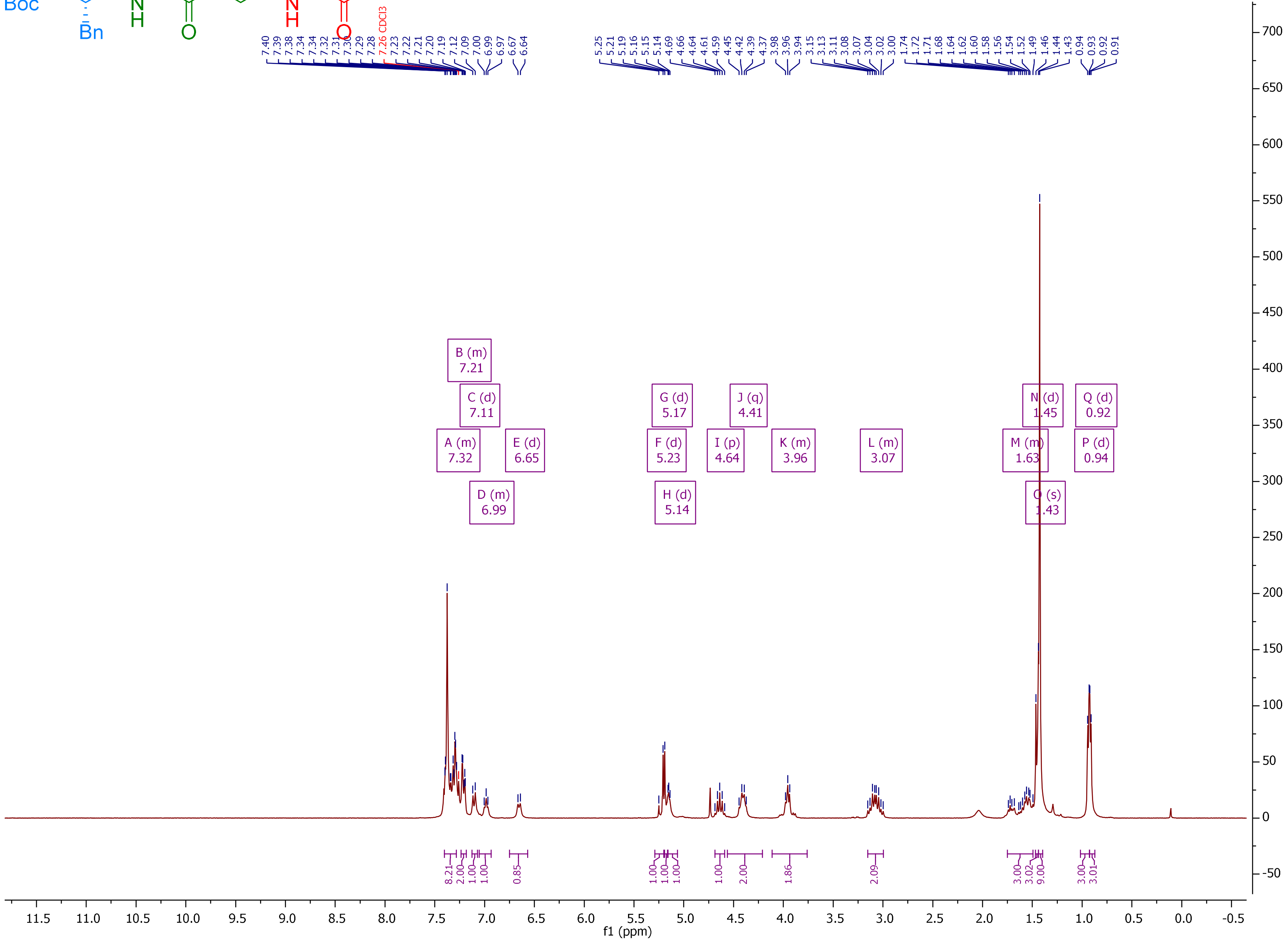

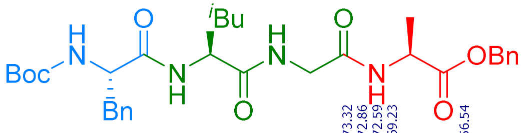

12c $^{13}C\{^{1}H\}$ NMR (75 MHz, Chloroform-*d*)

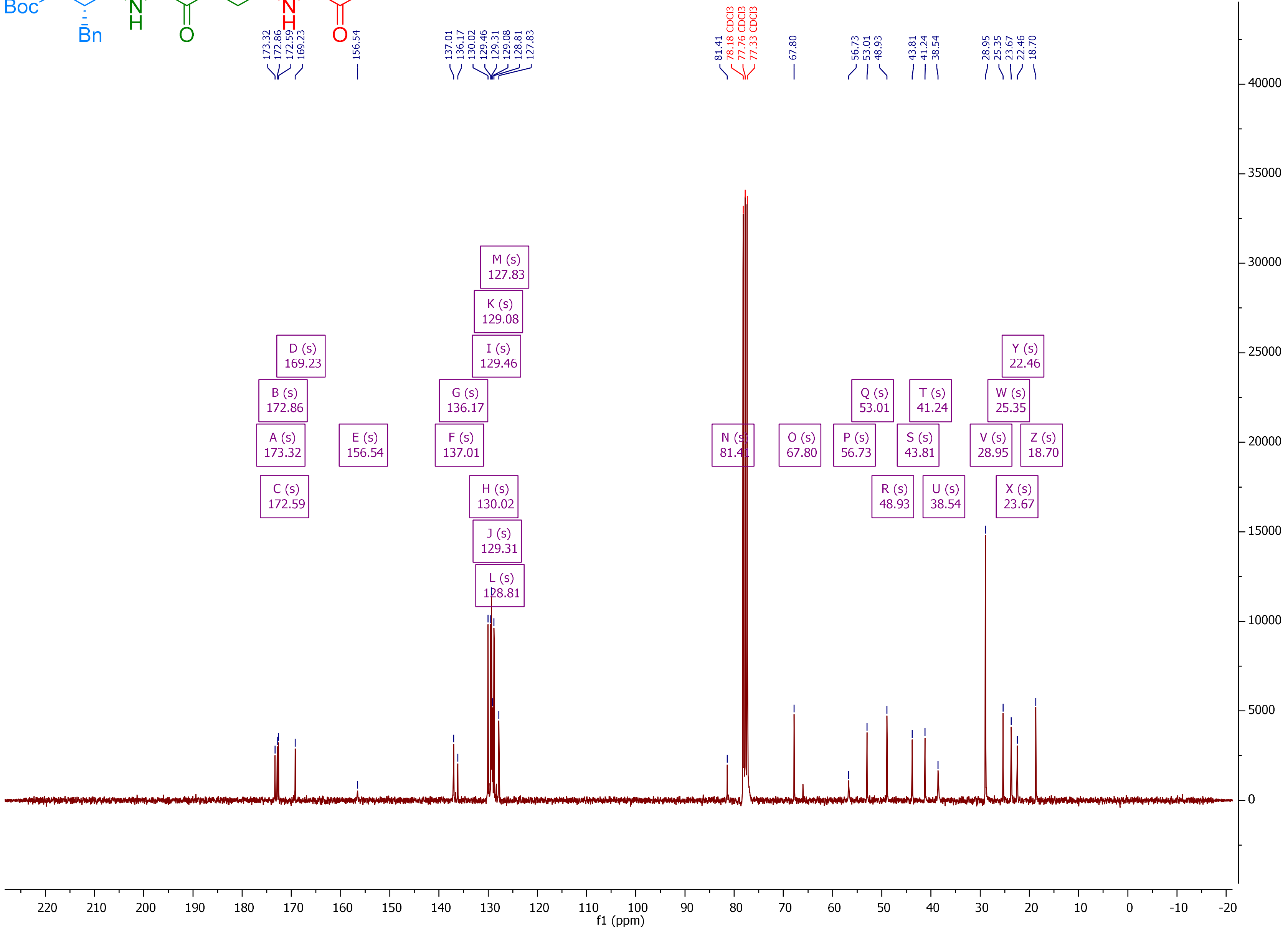

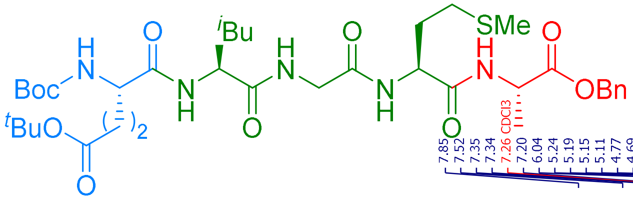

12d ¹H NMR (300 MHz, Chloroform-*d*)

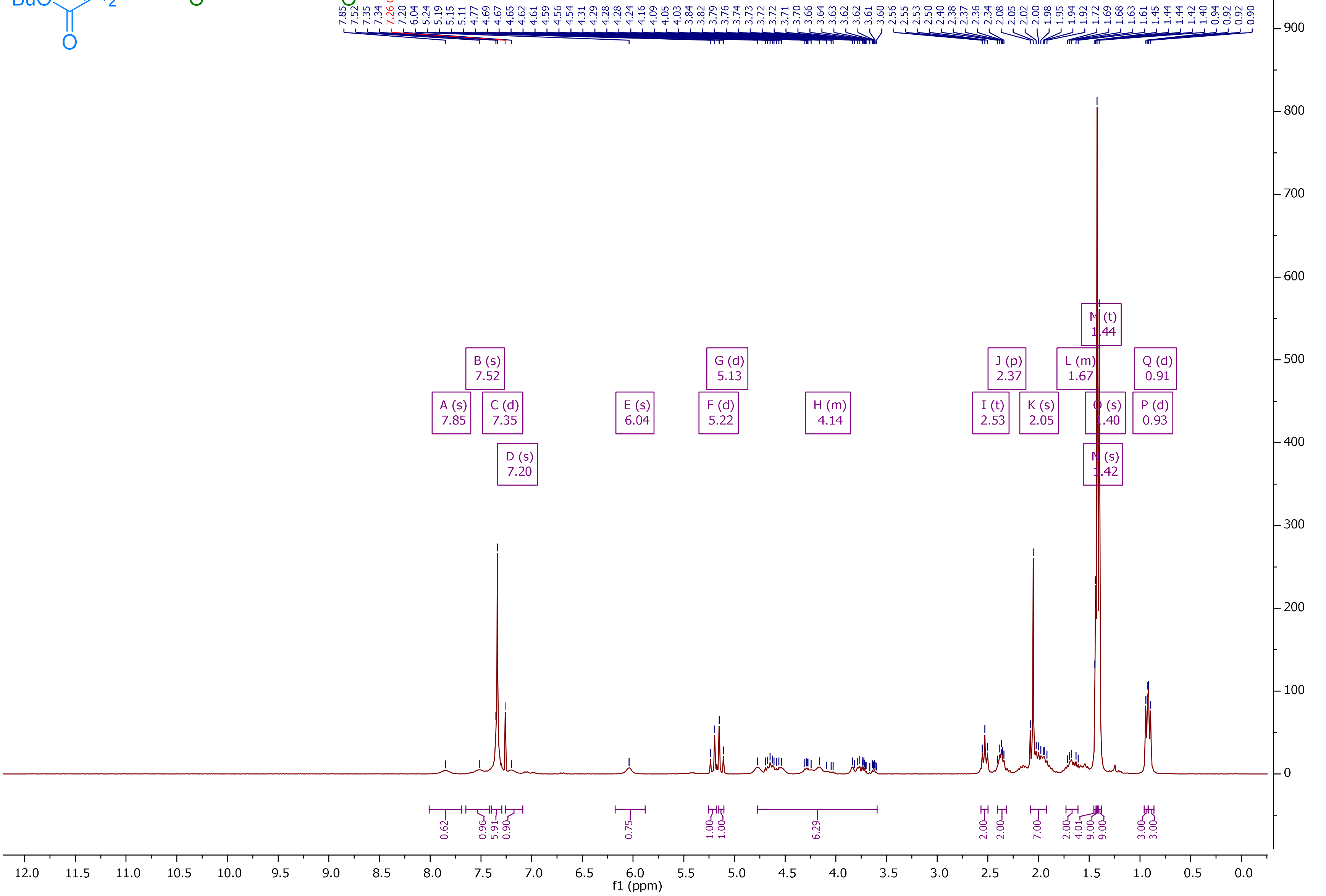

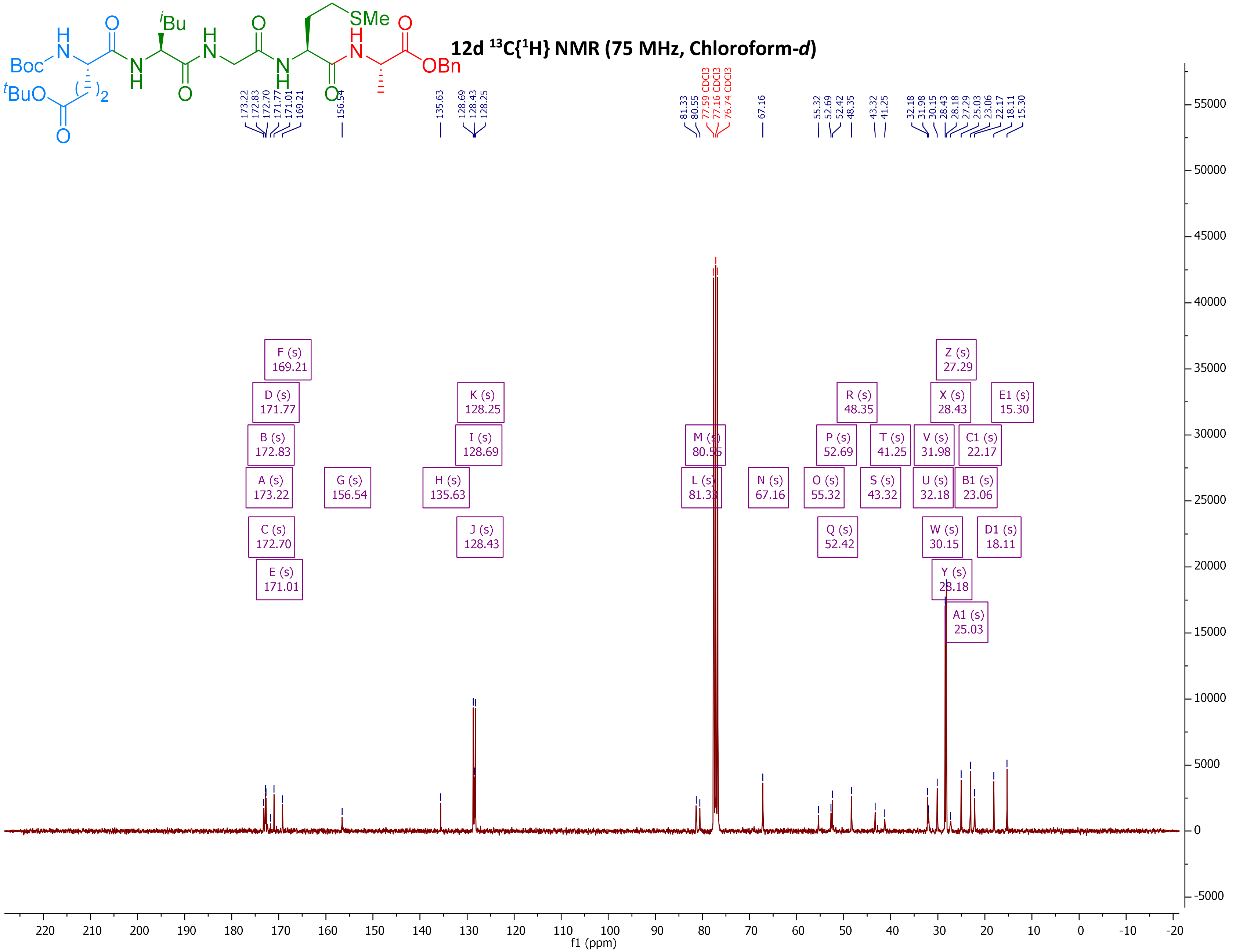

12d $^{13}C\{^1H\}$ NMR (75 MHz, Chloroform-*d*)
Boc
^{t}BuO
^{i}Bu
SMe
OBn
173.22
172.83
172.70
171.77
171.01
169.21
156.54
135.63
128.69
128.43
128.25
81.33
80.55
77.59 CDCl3
77.16 CDCl3
76.74 CDCl3
67.16
55.32
52.69
52.42
48.35
43.32
41.25
32.18
31.98
30.15
28.43
28.18
27.29
25.03
23.06
22.17
18.11
15.30
f1 (ppm)

12e ^{1}H NMR (300 MHz, Chloroform-*d*)

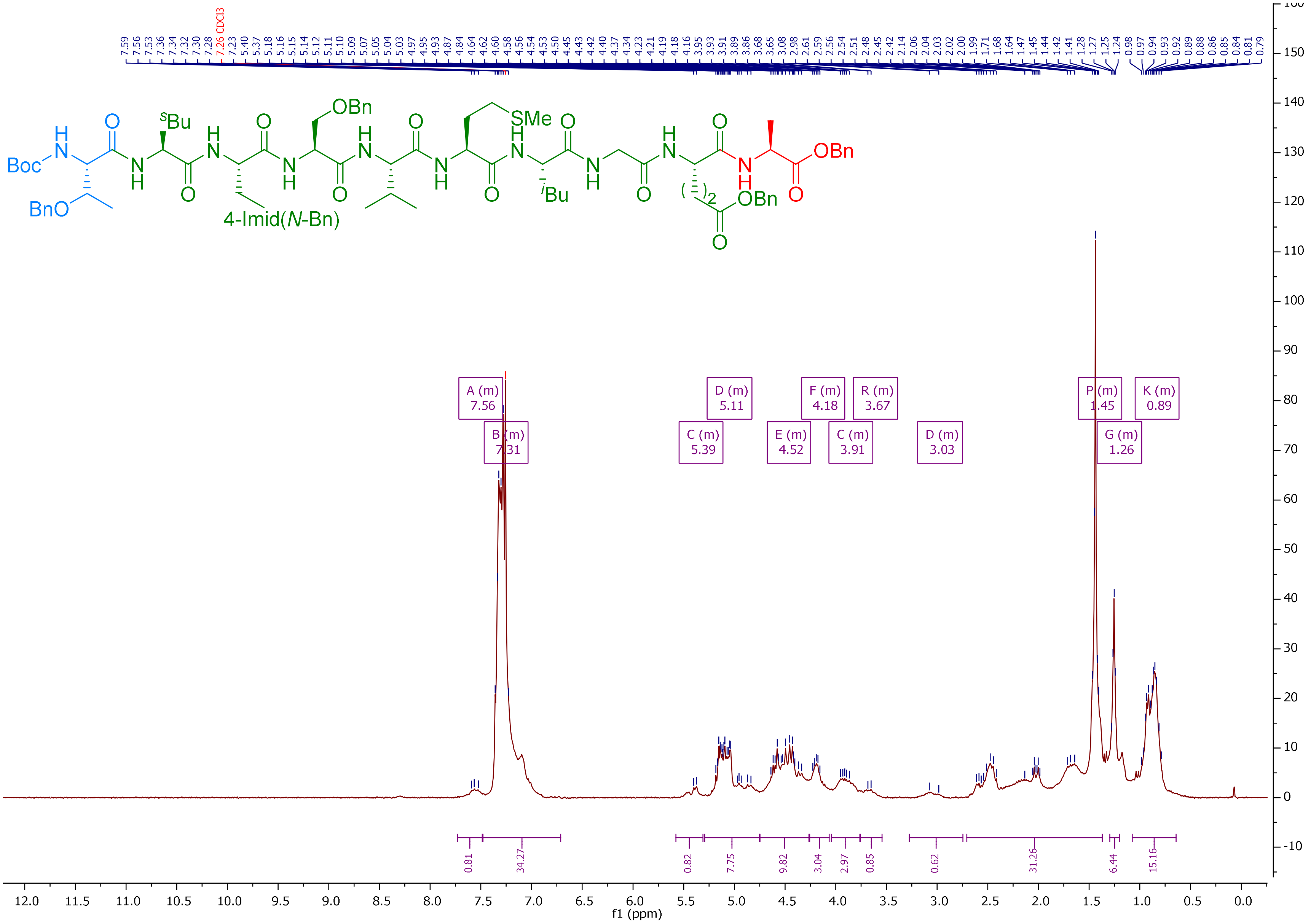

12e $^{13}C\{^{1}H\}$ NMR (75 MHz, Chloroform-*d*)

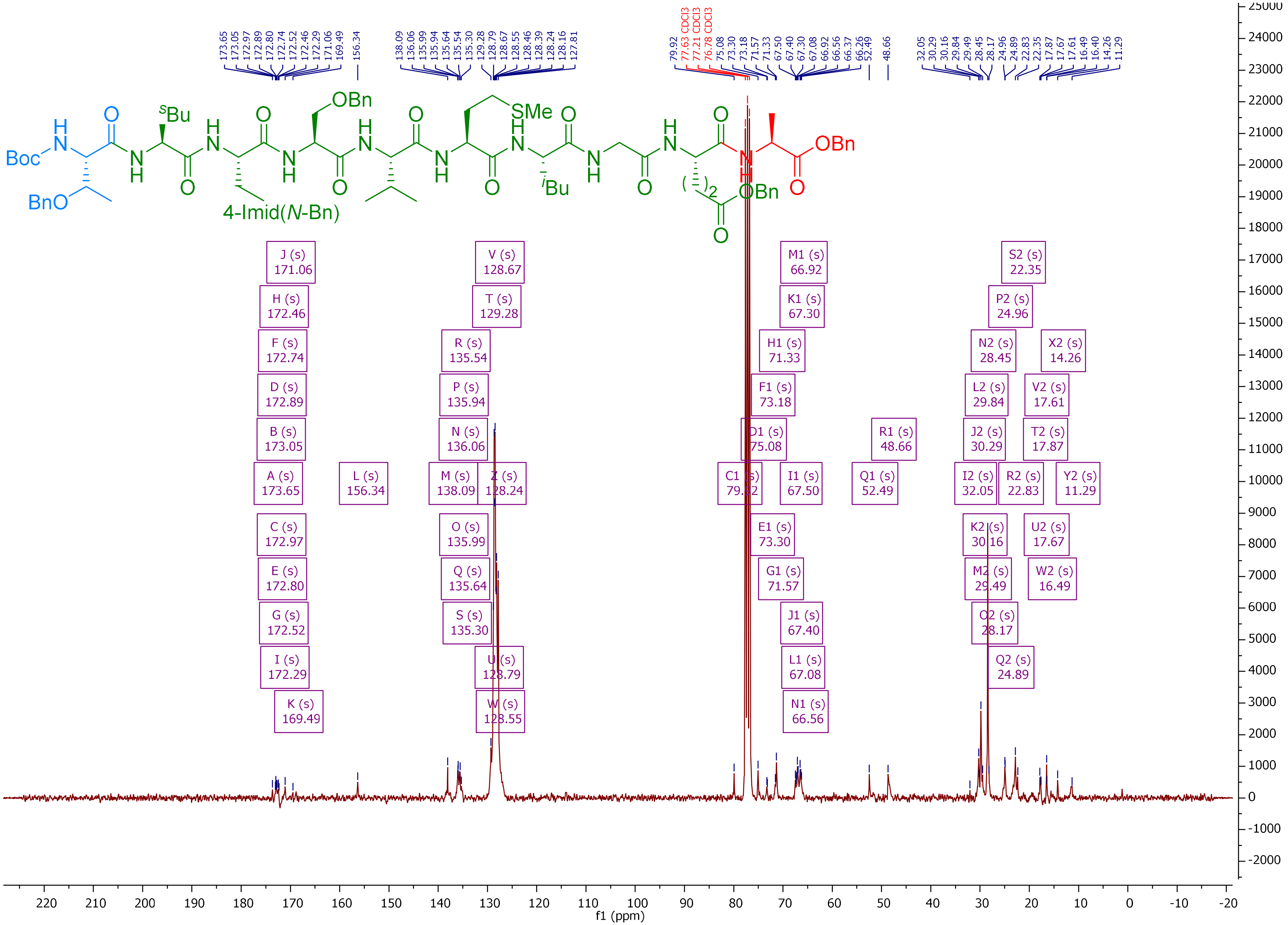

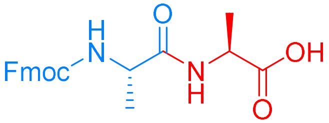

13a ^{1}H NMR (300 MHz, DMSO-d)

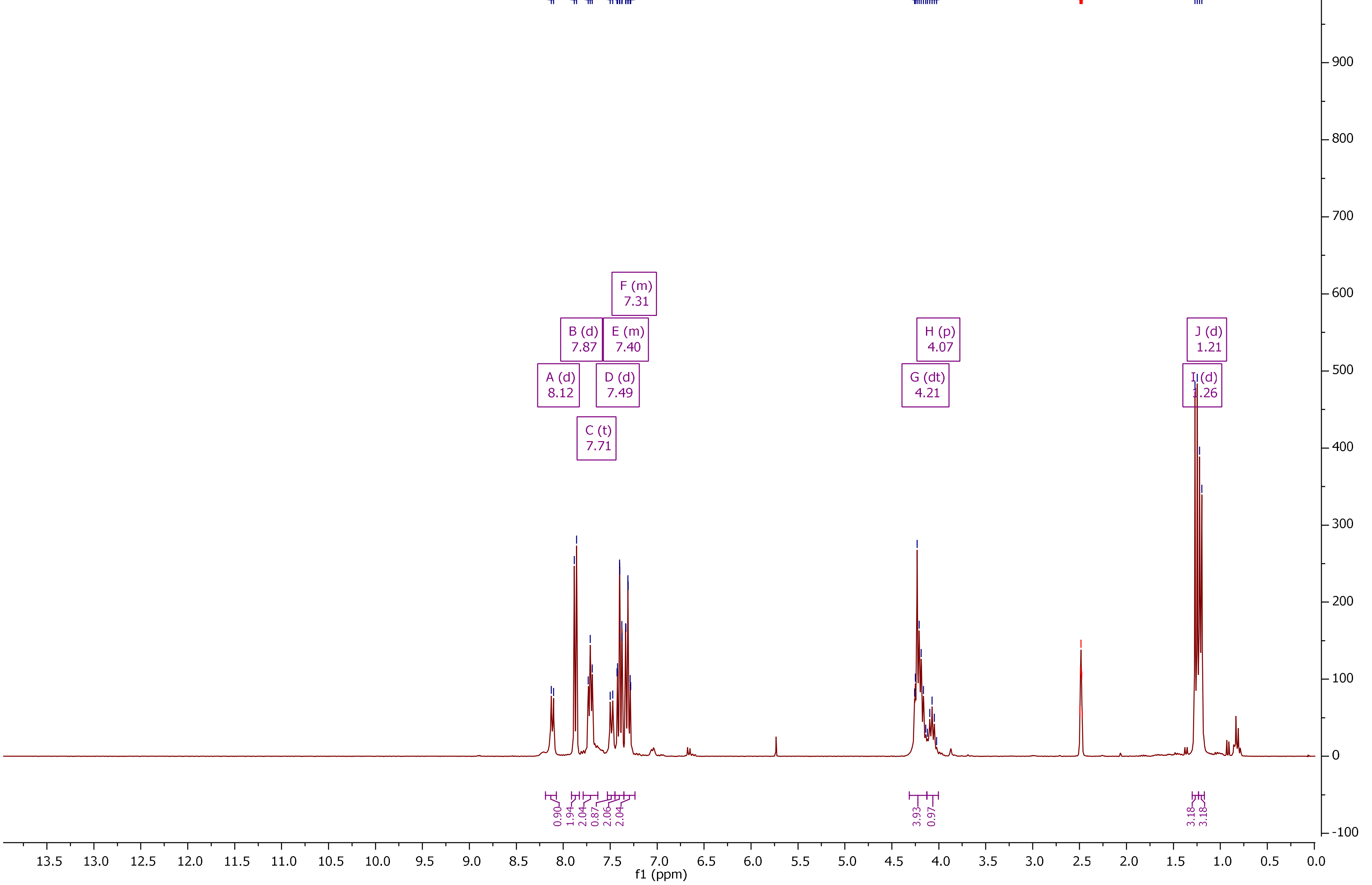

13a $^{13}C\{^1H\}$ NMR (75 MHz, DMSO-*d*)

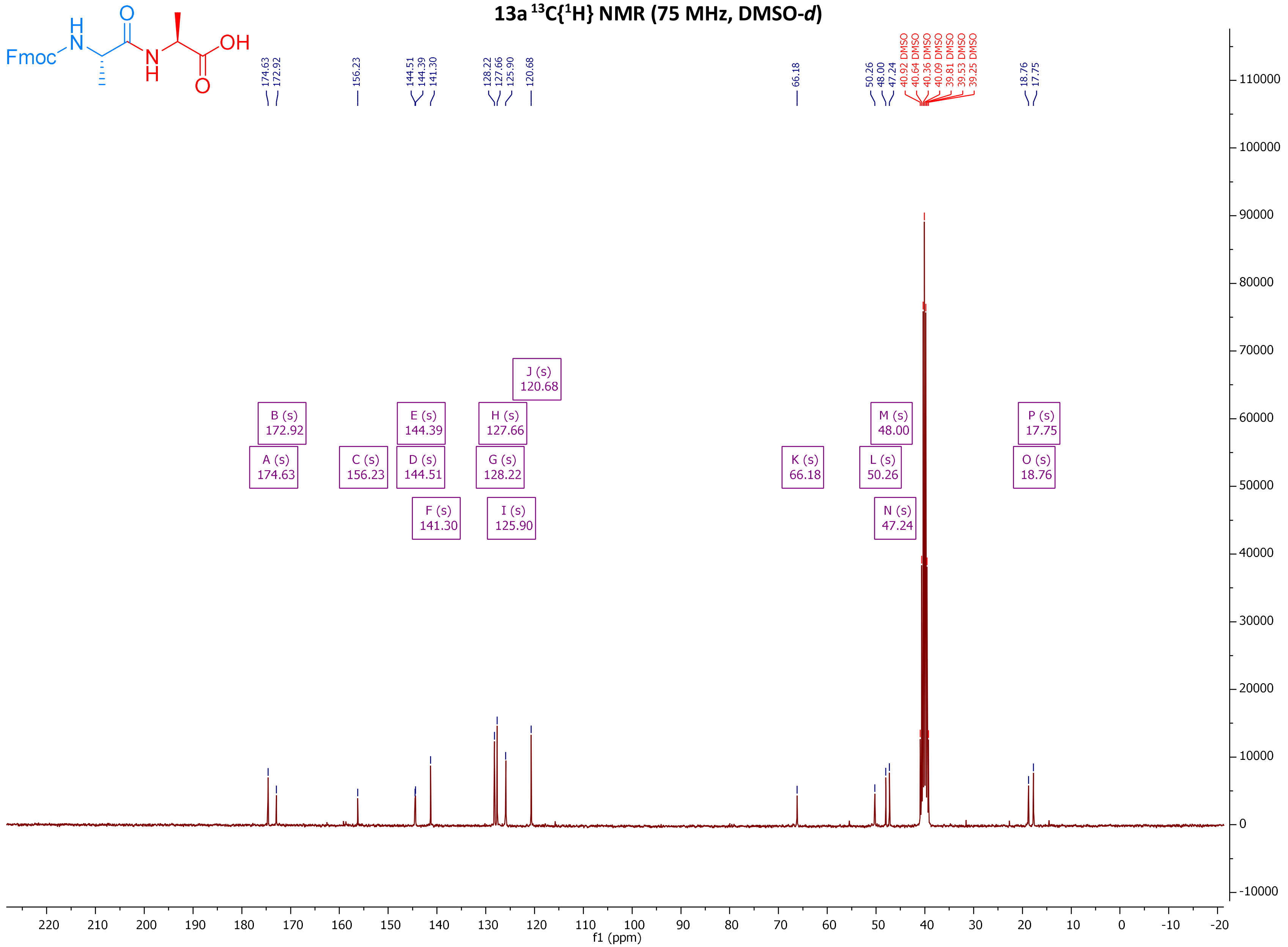

13b ^{1}H NMR (300 MHz, DMSO-*d*)

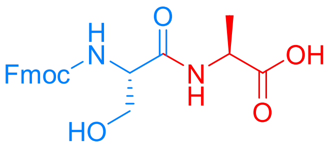

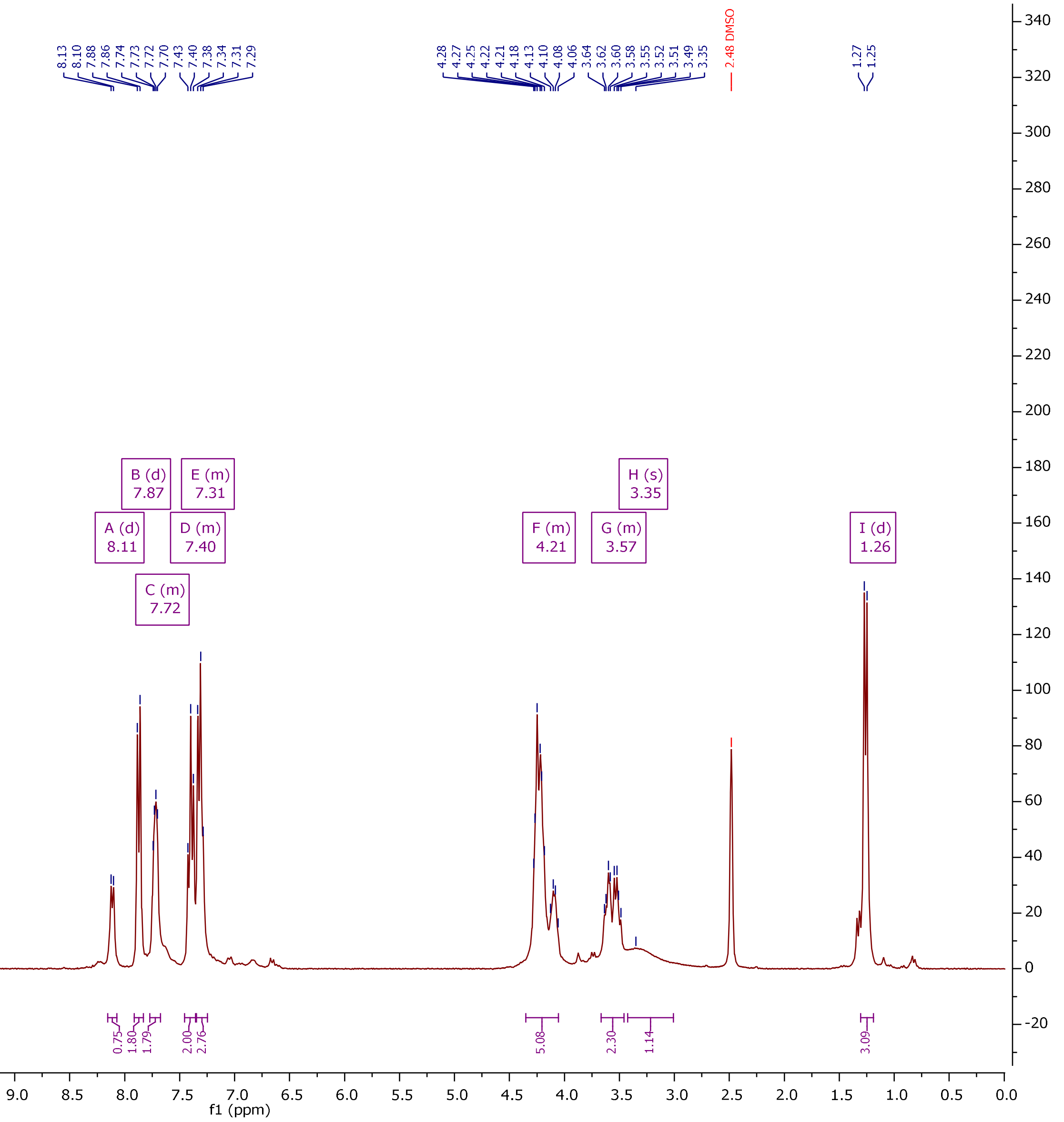

13b $^{13}C\{^1H\}$ NMR (75 MHz, DMSO-*d*)

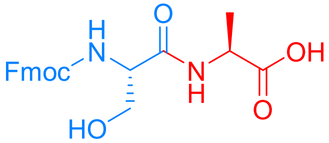

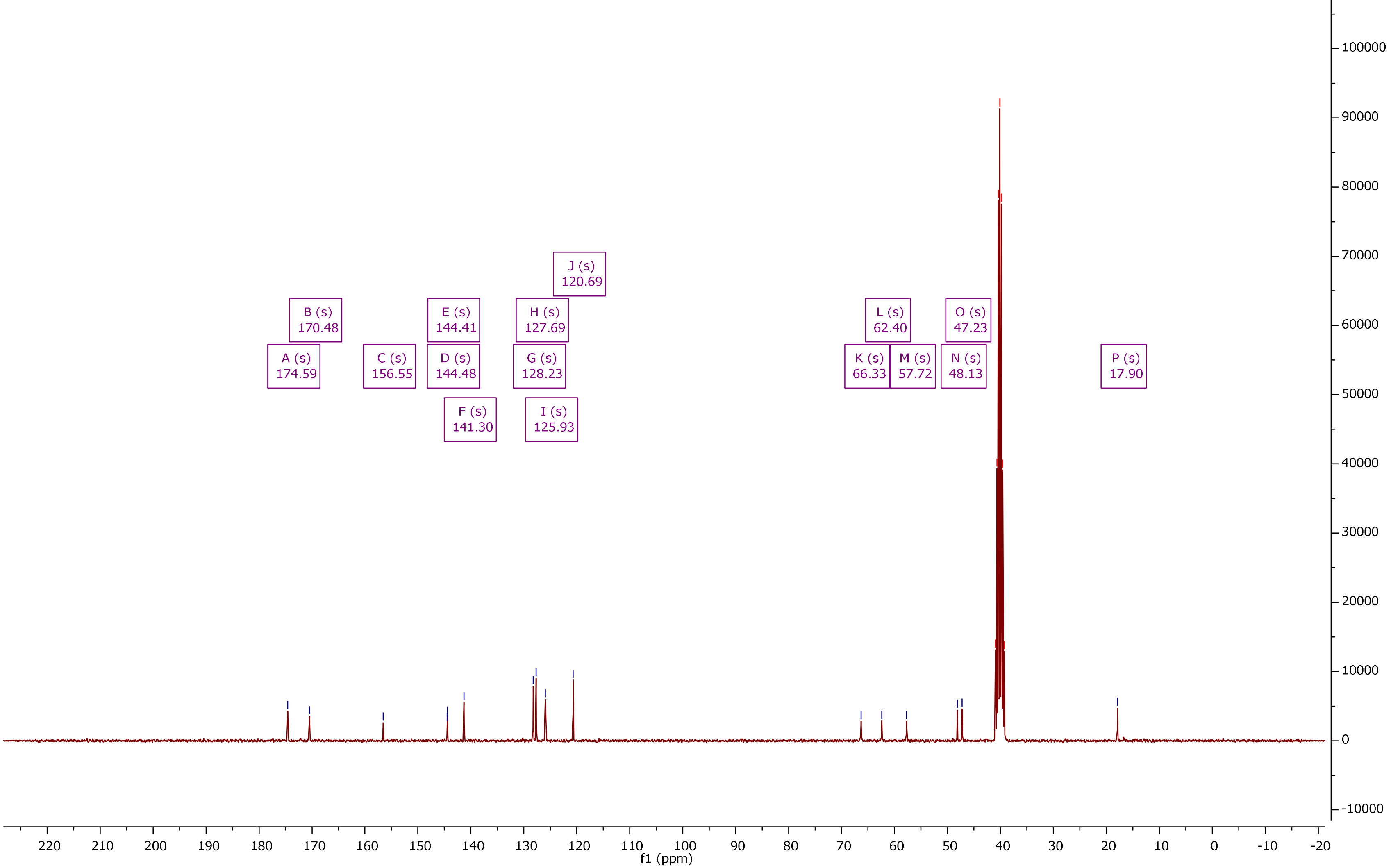

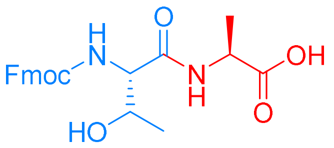

13c ¹H NMR (300 MHz, DMSO-*d*)

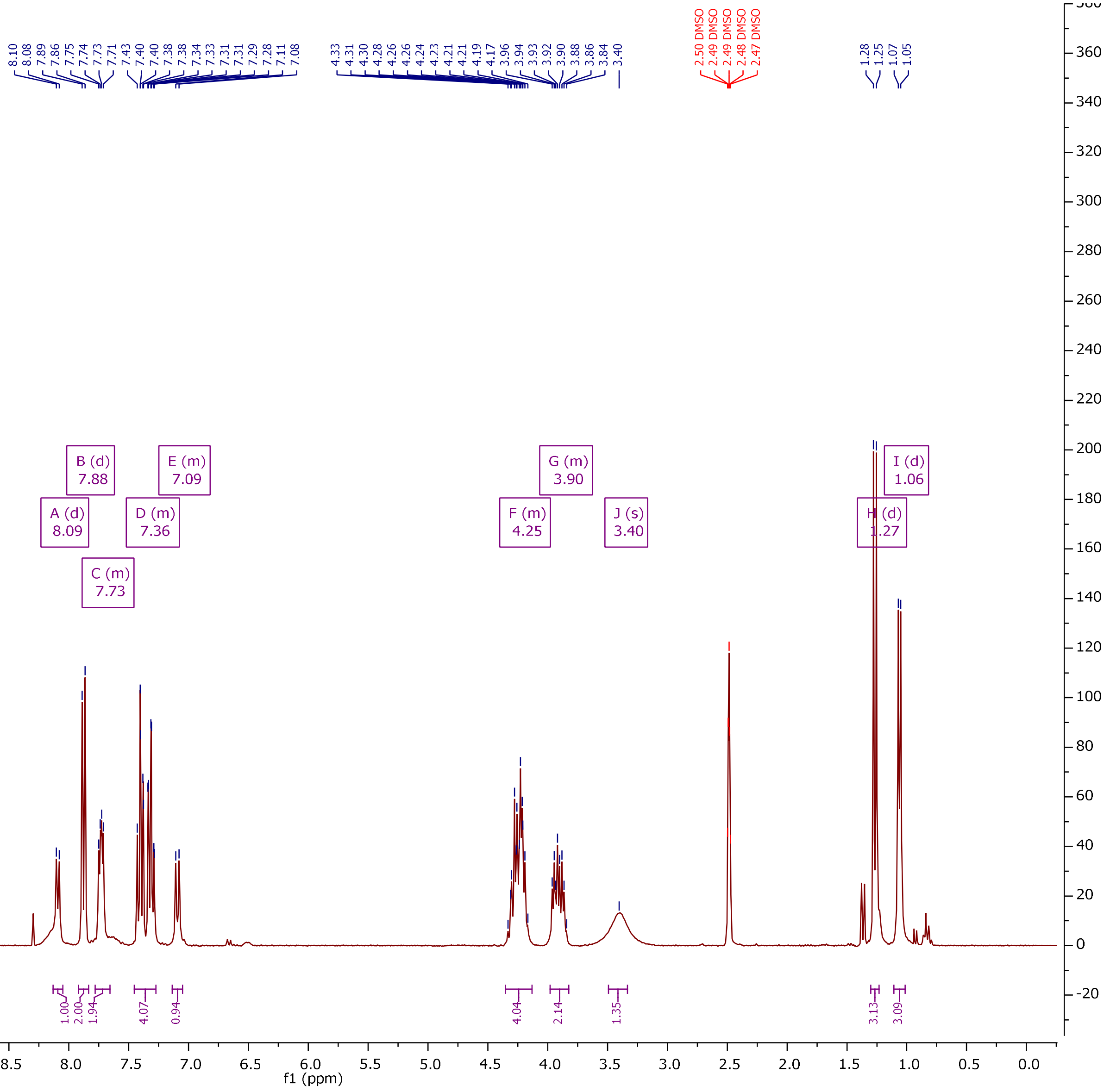

13c $^{13}C\{^{1}H\}$ NMR (75 MHz, DMSO-*d*)

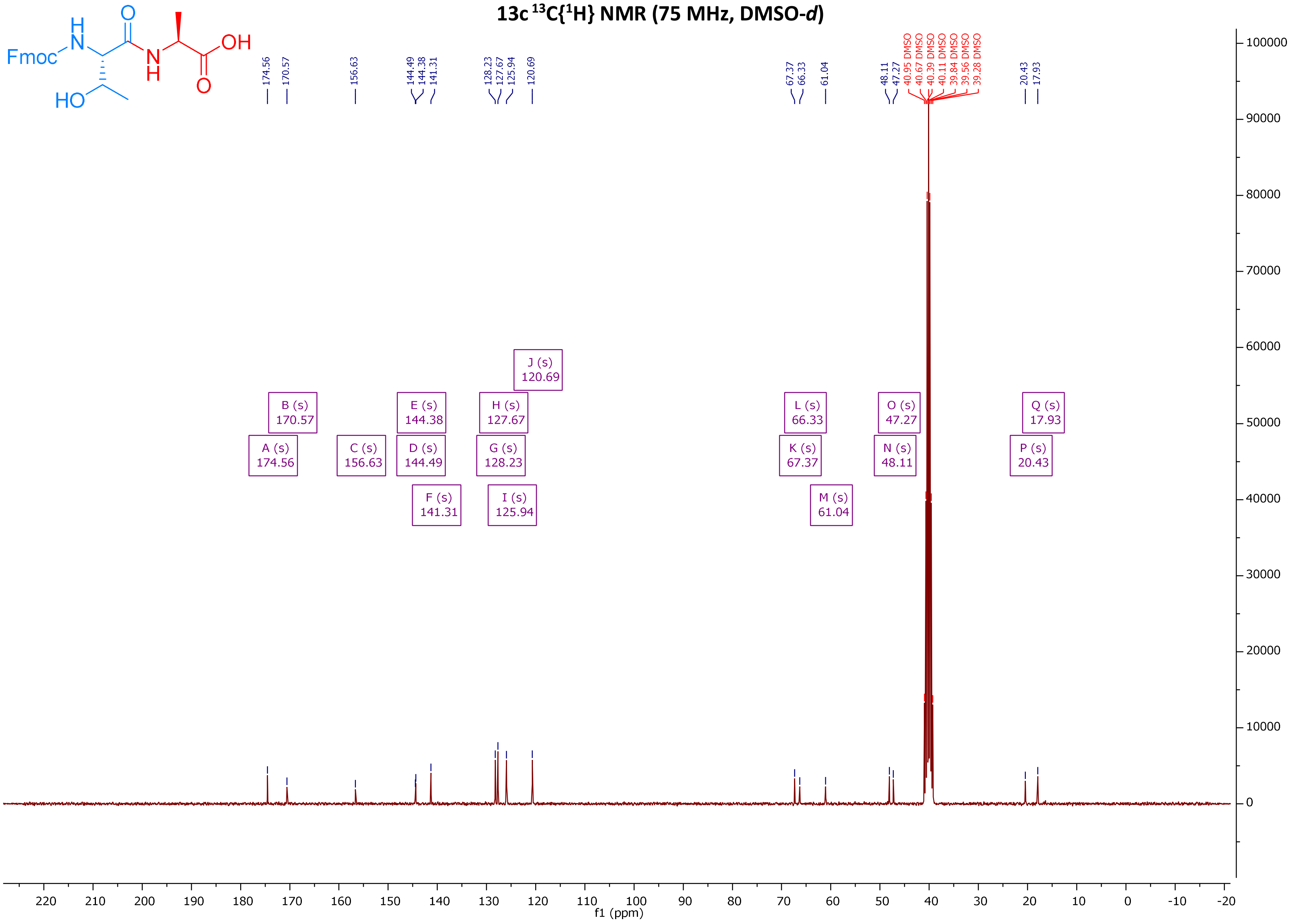

13d ¹H NMR (300 MHz, DMSO-*d*)

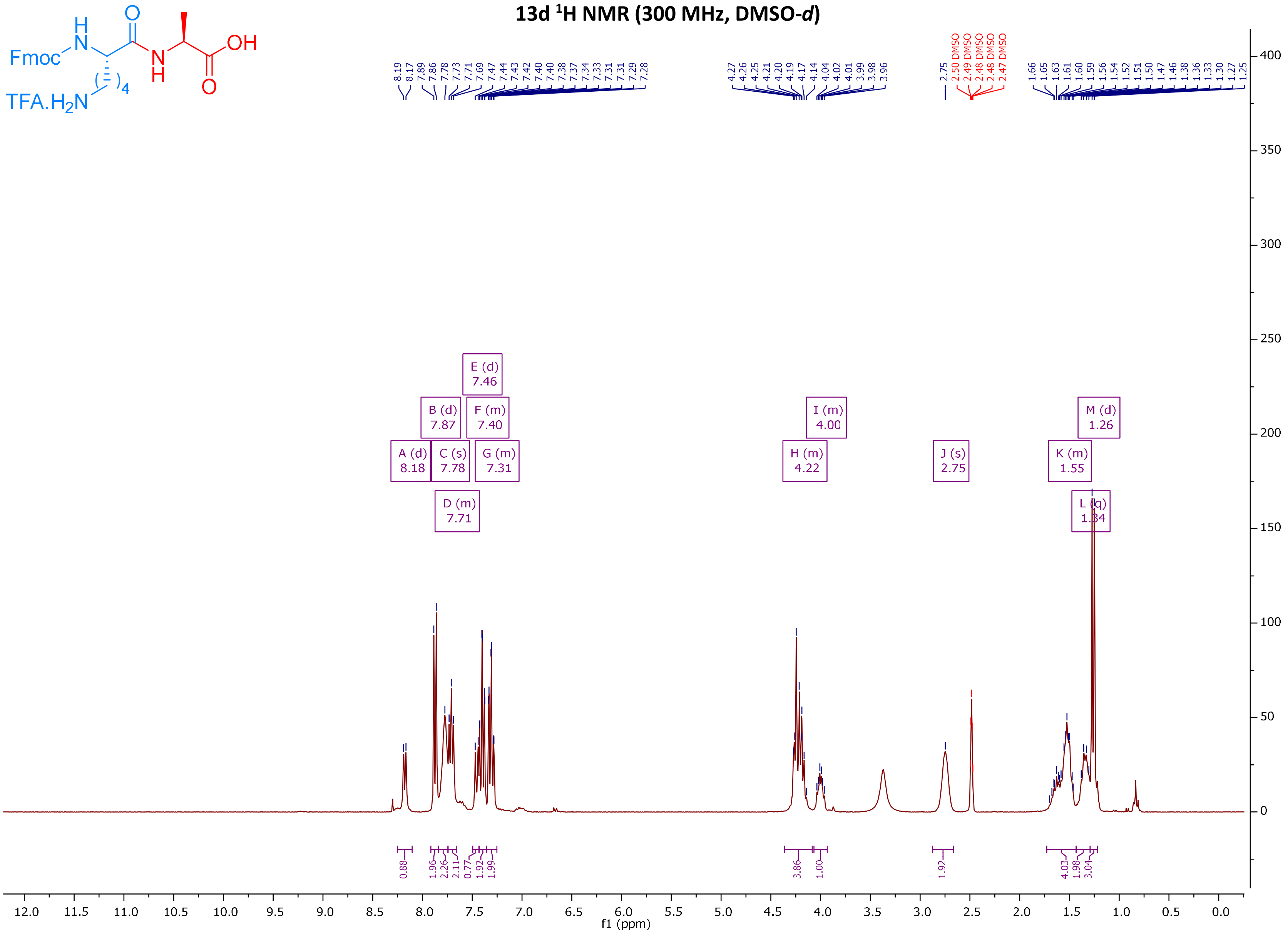

13d $^{13}C\{^1H\}$ NMR (75 MHz, DMSO-*d*)

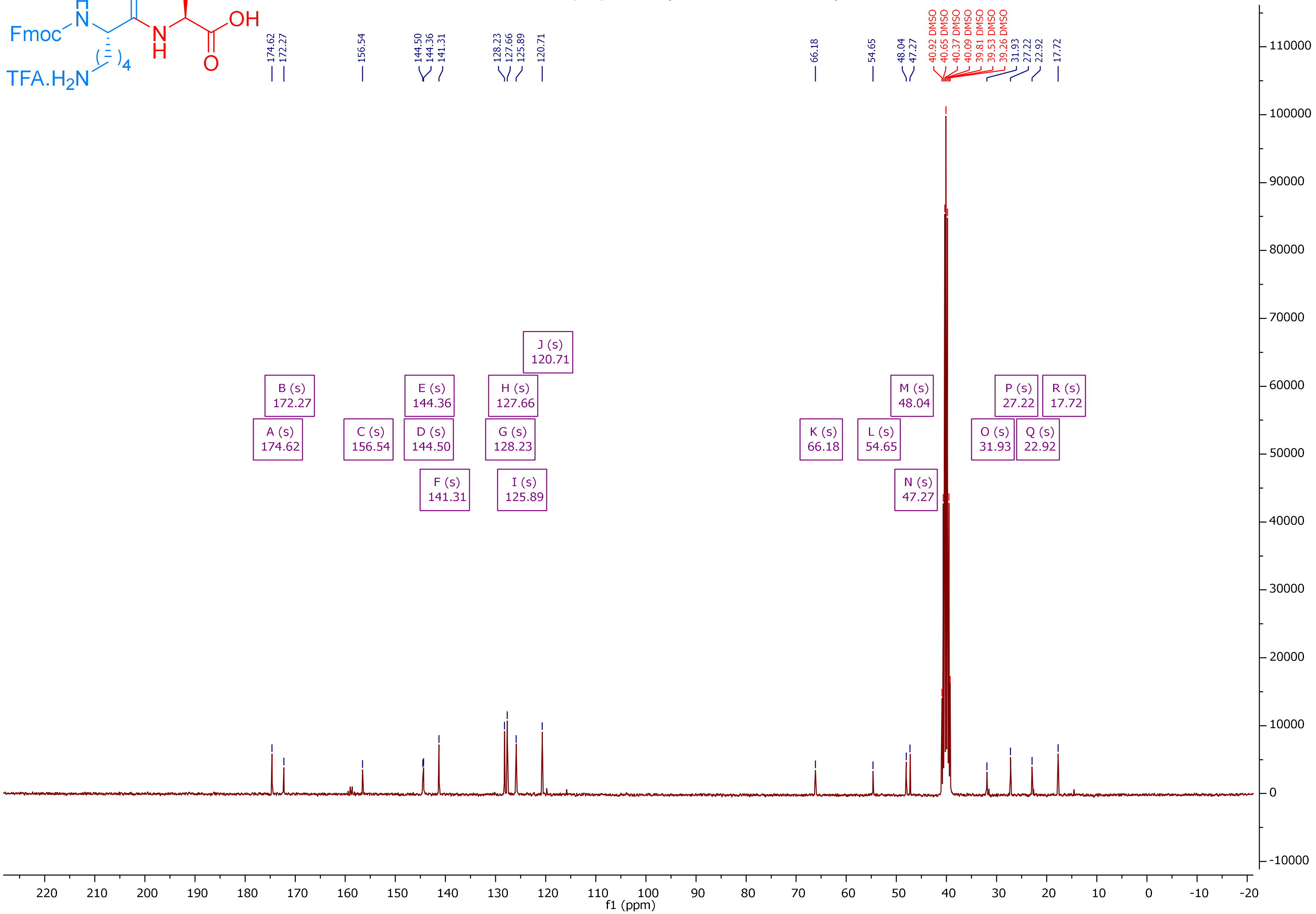

13e ^{1}H NMR (300 MHz, DMSO-*d*)

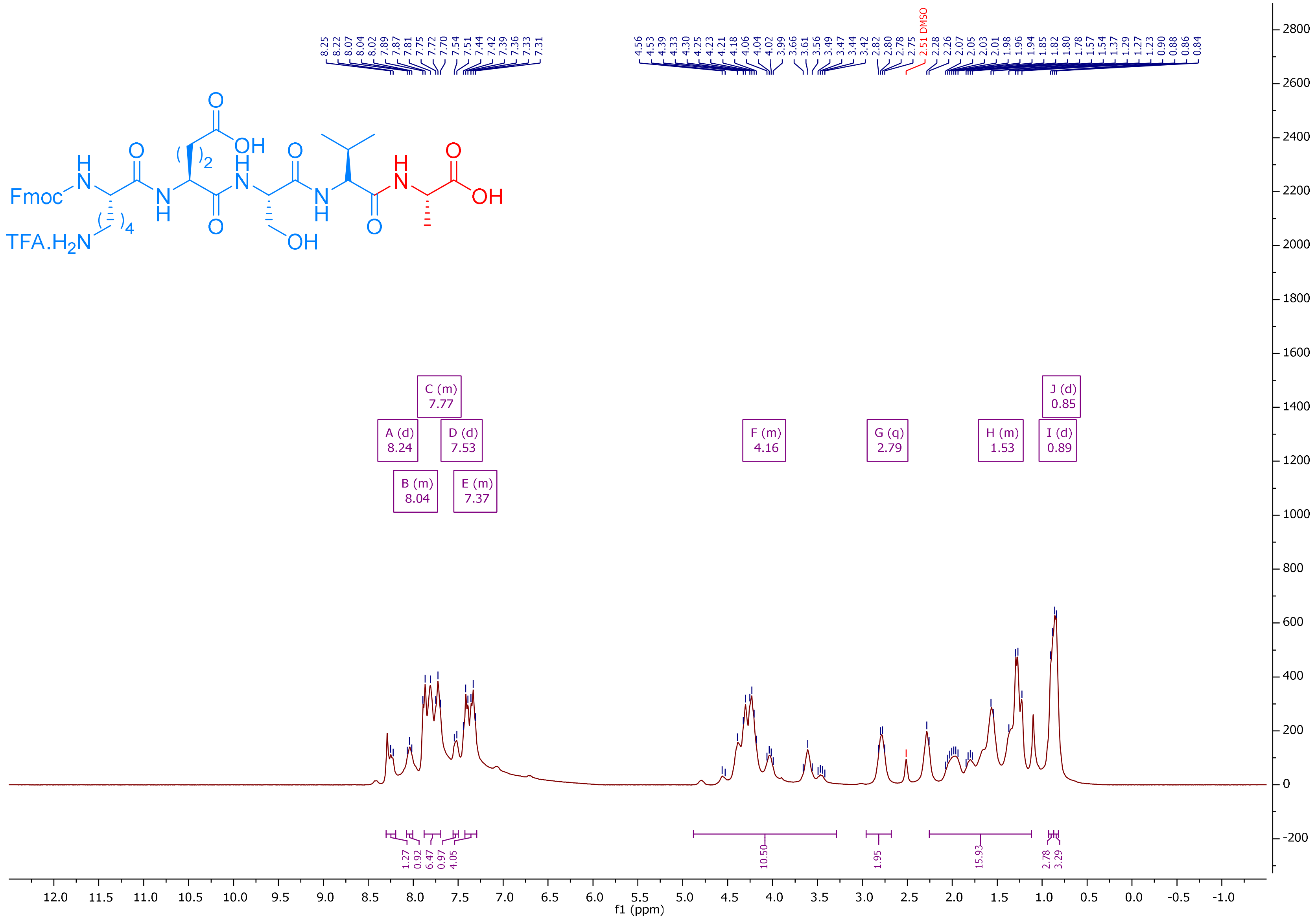

13e $^{13}C\{^{1}H\}$ NMR (75 MHz, DMSO-*d*)

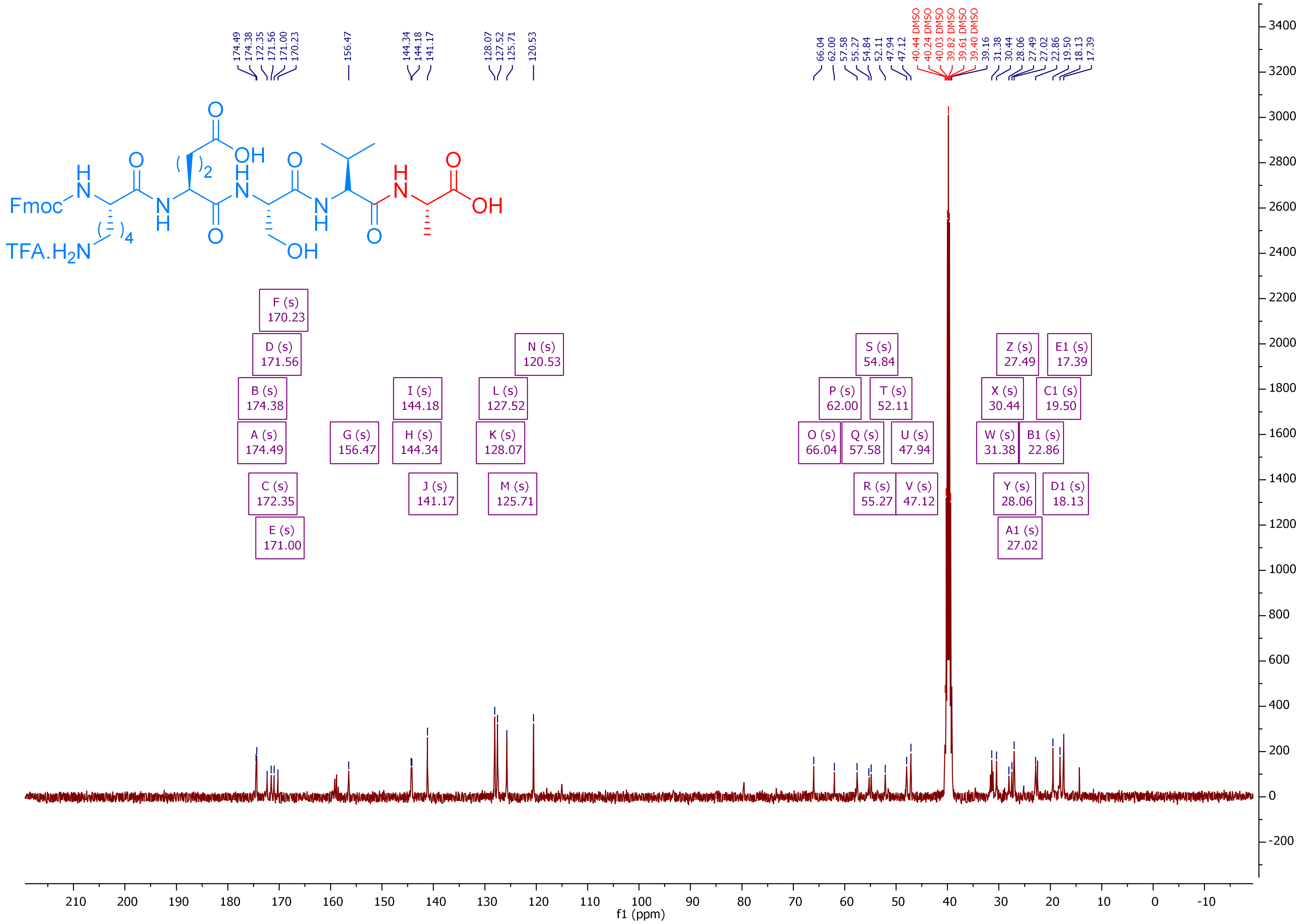

Supplement: SC-013-D1SC05316G-s001 [file SC-013-D1SC05316G-s001.pdf]
